# Synthesis and Anti-HBV Activity of Novel 3′-*N*-phenylsulfonyl Docetaxel Analogs

**DOI:** 10.3390/molecules180910189

**Published:** 2013-08-22

**Authors:** Jun Chang, Yun-Peng Hao, Xiao-Dong Hao, Hong-Fu Lu, Jian-Ming Yu, Xun Sun

**Affiliations:** Department of Natural Products Chemistry, School of Pharmacy, Fudan University, Shanghai 201203, China

**Keywords:** 3′-*N*-phenylsulfonyl, docetaxel analogs, synthesis, anti-HBV

## Abstract

Nine new 3′-*N*-phenylsulfonyl docetaxel analogs were synthesized in good yields from the key intermediate *N*-phenylsulfonyl oxazolidine via a six-step route. These analogs were tested for anti-hepatitis B virus (HBV) activity in vitro. Compounds **3e**, **3g** and **3j** showed more potent inhibitory activity against HBeAg secretion than the positive control lamivudine. Further extensive SAR and mechanistic studies will be reported in due course.

## 1. Introduction

Hepatitis B virus (HBV) is a major cause of acute and chronic hepatitis which can lead to liver cirrhosis, liver failure and hepatocellular carcinoma [1]. It was estimated there are 350 million chronic carriers and that about 2 billion people have been infected, and an estimated 600,000 to 1.2 million people die each year from HBV-associated illnesses [2,3]. Currently, therapies for HBV infection on market include immuno-modulators, interferons (interferon-alpha and pegylated interferon) and nucleoside analogues. However, these drugs still have their drawbacks. For example, immune-modulators (IFN-a and pegIFN-a) and polymerase inhibitors (lamivudine, entecavir, telbivudine and adefovir) are associated with a low cure rate, viral resistance, poor tolerability, and inefficiency in eradicating HBV [4,5,6,7]. Therefore, there is a need to search for new anti-HBV agents with novel antiviral targets and mechanisms of action.

**Figure 1:**
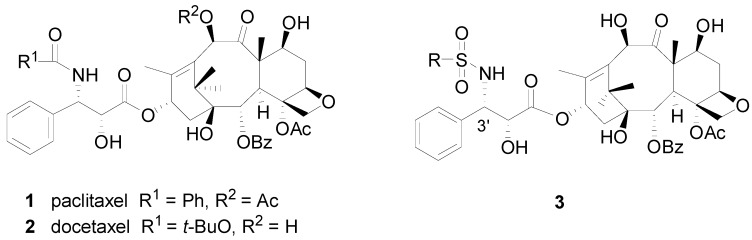
Structure of paclitaxel, docetaxel and 3′-*N*-phenylsulfonyl docetaxel analogs.

Natural products and their derivatives have always played a pivotal role as leads in drug discovery. Paclitaxel (**1**, Figure 1) and its semi-synthetic derivative docetaxel (Taxotere, **2**), are currently considered to be the most important and promising anticancer agents in the treatment of refractory breast and ovarian cancers due to their unique mechanism of action by binding tubulin and stabilizing microtubule formation, which ultimately disrupts mitosis and causes cell death [8]. In our studies of docetaxel derivatives as anticancer agents [9,10], many analogs were synthesized, including one 3′-*N*-phenylsulfonyl docetaxel analog. It was found that this 3′-*N*-phenylsulfonyl docetaxel analog didn’t show any potent antitumor activity [11,12]. However, it has been reported that a large number of structurally novel sulfonamide derivatives have shown substantial antiviral activity both *in vitro* and *in vivo* [13,14]. Accordingly, a series of novel 3′-*N*-phenylsulfonyl docetaxel analogs were designed, synthesized and investigated for their HBV inhibitory activity. It was hoped that a promising lead could be emerged from this type of structure.

## 2. Results and Discussion

### 2.1. Chemistry

**Scheme 1:**
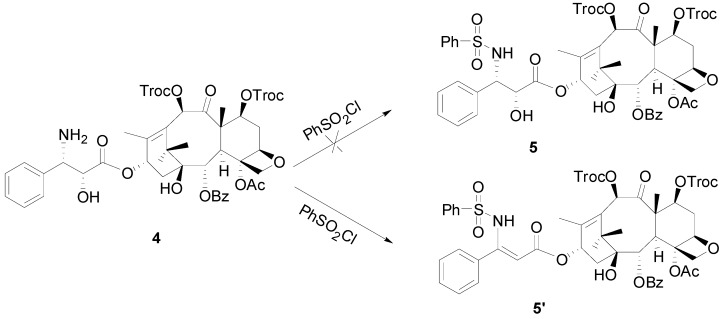
Attempted synthesis of compound **5**.

In order to synthesize these 3′-*N*-phenylsulfonyl docetaxel analogues, several synthetic routes were approached. The first attempt was to couple the intermediate *N*-de-*tert*-butoxy-carbonyl-7,10-ditroc-docetaxel (**4**) with phenylsulfonyl chloride directly (Scheme 1).

However, ESIMS of the product showed a quasimolecular ion peak at m/z 852 [M+Na]^+^, indicating a molecular weight of 829, or 18 Da lower than that of **5**. Since the H-3′ peak had disappeared in ^1^H-NMR spectrum, it was suggested that the dehydrated product **5′** was obtained rather than **5**. Obviously the elimation occurred quickly since the benzenesulfonate is a very good leaving group formed by phenylsulfonyl and hydroxyl groups.

**Scheme 2:**
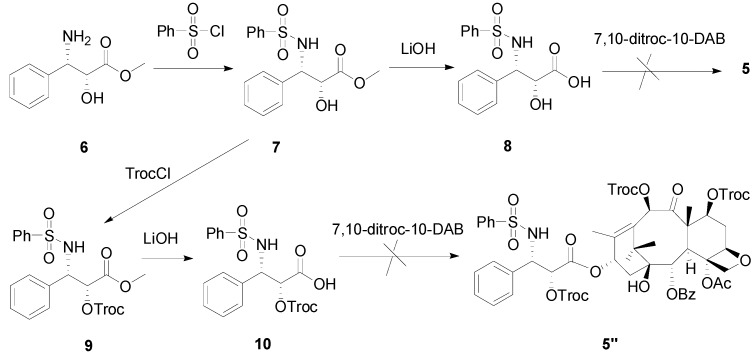
Another attempted synthesis of compound **5**.

The second attempt was to synthesize the side chain from (2*R*,3*S*)-3-amino-2-hydroxy-3-phenyl-propionic acid methyl ester (**6**), as illustrated in Scheme 2. Compound **6** was transformed into phenylsulfonamide **7** with phenylsulfonyl chloride, followed by saponification to afford acid **8**. However, the coupling reaction between **8** and the intermediate 7,10-ditroc-10-deacetylbaccatin failed to yield **5**, partially because **8** was more prone to undergo self-condensation due to the steric hindrance of the hydroxyl group in 10-DAB. Since the free hydroxyl group in **7** interfered with the reaction, we decided to protect it first. Thus, after protecting the hydroxyl group of **7** with 2,2,2-trichloroethyl chloroformate (TrocCl) followed by saponification, the carboxylic acid **10** was obtained as expected. However, the desired product **5′′** was still not formed when **10** reacted with 7,10-ditroc-10-DAB.

**Scheme 3:**
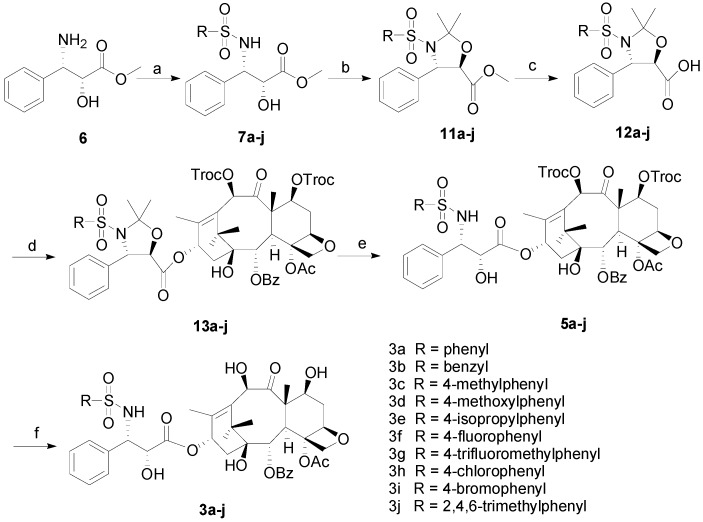
Synthesis of compounds **3a**–**j**.

Ke *et al.* reported the synthesis of novel 3′-*N*-*tert*-butylsulfonyl analogs of docetaxel by asymmetric synthesis of the side chain oxazolidine and condensing this oxazolidine with 7,10-ditroc-10-DAB [12]. Unfortunately, the oxazolidine is only obtained as diastereomeric mixtures via five steps from (*R*)-*tert*-butylsulfinylimine. We previously reported the synthesis of fluorinated docetaxel derivatives from the enantiopure *N*-Boc oxazolidine side chain [9,10]. Therefore, *N*-phenylsulfonyl oxazolidine could be used as an important intermediate for this condensation reaction. Finally, compounds **3a**–**j** were synthesized via a new six-step route in good yields from (2*R*,3*S*)-3-amino-2-hydroxy-3-phenyl-propionic acid methyl ester (**6**), a commercially available starting material, as illustrated in Scheme 3. Compound **6** was first transformed into phenylsulfonamides **7a**–**j** with phenylsulfonyl chloride. Cyclic protection using methoxypropene in the presence of a catalytic amount of pyridinium *para*-toluenesulfonate (PPTS) followed by saponification of the formed intermediates **11a**–**j** afforded acids **12a**–**j** in 80%–95% yields. Then, key intermediates **12a**–**j** were coupled with 7,10-ditroc-10-DAB in the presence of dicyclohexylcarbodiimide (DCC) and 4-dimethylaminopyridine (DMAP) to provide the corresponding intermediates **13a**–**j** in 85%–95% yields. After removing the acetonide protecting group of **13a**–**j** with 98% formic acid at room temperature**,** intermediates **5a**–**j** were obtained in 57%–85% yields. After further deprotection of the 7,10-ditroc protecting groups on **5a**–**j** with zinc in acetic acid, 3′-*N*-phenylsulfonyl docetaxel analogs **3a**–**j** were synthesized in 53%–80% yields. 

*Reagents and conditions:* (a) RSO_2_Cl/Et_3_N, CH_2_Cl_2_, 0 °C-r.t., 1 h; (b) CH_2_ = C(OCH_3_)CH_3_, PPTs, toluene, 85 °C, 2 h; (c) LiOH, THF/H_2_O, r.t., 1 h; (d) 7,10-ditroc-10-DAB, DCC, DMAP, toluene; 85 °C, 3 h; (e) HCOOH, r.t., 1.5 h; (f) Zn/HOAc, MeOH, 50 °C, 1 h.

### 2.2. Anti-HBV Activity

With compounds **3a**–**j** in hand, they were tested for their antiviral activity against hepatitis B virus (HBV) *in vitro* [15], and the results are summarized in Table 1.

**Table 1:** Anti-HBsAg and anti-HBeAg effects of compounds **3a**–**j** in HepG2 2.2.15 cell line.

	MNTC *^b^* (in µM or µg/mL)	HBsAg inhibition (%)	HBeAg inhibition (%)
**3a**	7.4 (6.25)	10.7	19.2
**3b**	14.5 (12.5)	10.0	34.1
**3c**	14.5 (12.5)	0	11.4
**3d**	28.5 (25)	11.8	14.8
**3e**	14.1 (12.5)	0	**44.4**
**3f**	28.9 (25)	0	3.0
**3g**	13.7 (12.5)	0	**47.0**
**3h**	14.2 (12.5)	5.0	25.8
**3i**	13.5 (12.5)	0	26.0
**3j**	14.1 (12.5)	0	**40.2**
**Docetaxel**	7.7 (6.25)	0	**8.2**
**Paclitaxel**	7.3 (6.25)	0	**5.3**
3TC *^a^*	873.4 (200)	10.9	12.5

*^a^* Positive control (3TC = Lamivudine); *^b^* Maximum non-toxic concentration. Cell damage was assessed by means of the MTT assay; cell growth inhibition ≥25% was considered as cytotoxic.

Overall, the compounds showed only weak or no inhibitory activity on HBsAg secretion, except for compounds **3c** and **3f**, **3a**–**b**, **3d**–**e** and **3g**–**j** that exhibited more potency against HBeAg secretion than the positive control lamivudine (12.5% at 873.4 µM). Especially, **3e**, **3g** and **3j** showed inhibitory activity on HBeAg secretion by 44.4%, 47.0% and 40.2% at the maximum non-toxic concentration (12.5 µg/mL), respectively. 

Preliminary structure-activity relationships (SAR) did not show a clear trend in terms of electron-withdrawing or electron-donating substituents on the phenyl ring. Only phenyl (**3a**), 4-methoxyl (**3d**), 4-chloro (**3h**) and benzyl (**3b**) analogs exihibited weaker inhibitory activity against both HBsAg secretion and HBeAg secretion. However, 4-isopropyl (**3e**), 4-trifluoromethyl (**3g**) and 2,4,6-trimethyl (**3j**) analogs demonstrated the most potent inhibitory activity against HBeAg secretion. Meanwhile, they were all inactive against HBsAg secretion. Accordingly, further extensive SAR study on the substituent pattern on phenyl ring and modification of the linker (based on the result of **3b**) will be continued in the near future.

## 3. Experimental

### 3.1. General

Reagents were purchased from the Aldrich (Shanghai, China) and TCI Chemical (Shanghai, China) companies. All solvents are purified and dried in accordance with standard procedures, unless otherwise indicated. Reactions were monitored by TLC using Yantai (Yantai, China) GF254 silica gel plates (5 × 10 cm). Silica gel column chromatography was performed on silica gel (300–400 mesh) from Yantai. Melting points (mp) were determined using an X-4 microscope melting point apparatus and were uncorrected. All NMR spectra were recorded on a Bruker DRX-400 (400/100 MHz) spectrometer. Low-resolution mass spectra (ESI) were performed on a Shimadzu LCMS-2010EV and high-resolution mass spectra on a Bruker Daltonics, Inc. APEXIII7.0 TESLA FMS (ESI). Elemental analysis was carried out on an Elementar Vario EL instrument.

### 3.2. Synthesis

#### 3.2.1. General Procedure for the Synthesis of **7a**–**j**

To a round-bottomed flask (2*R*,3S)-*3*-amino-2-hydroxy-3-phenyl-propionic acid methyl ester (**6**, 0.39 g, 2 mmol) was added into THF (15 mL), which was cooled to 0 ^o^C. To this suspension was added Et_3_N (1.11 mL, 8 mmol), followed by the dropwise addition of phenylsulfonyl chloride (2.2 mmol). After further stirred at this temperature for 1 h, it was diluted with DCM (30 mL). The organic layer was washed with brine, dried over anhydrous Na_2_SO_4_ and filtered. Then the filtrate was evaporated and the residue was purified by column chromatography using petroleum ether/EtOAc (2/1) to afford the products **7a**–**j** as white solids. 

*2-Hydroxy-3-benzenesulfonylamino-3-phenyl-propionic acid methyl ester* (**7a**). Yield 59% (395 mg); ^1^H-NMR (CD_3_COCD_3_): *δ* 3.60 (s, 3H, OCH_3_), 4.35 (d, 1H, *J* = 3.2 Hz, 2-CH), 4.88 (d, 1H, *J* = 3.2 Hz, 3-CH), 7.14 (m, 3H, 3-Ph), 7.27 (m, 2H, 3-Ph), 7.35 (t, 2H, *J* = 7.6 Hz, *m*-PhSO_2_), 7.46 (t, 1H, *J* = 7.6 Hz, *p*-PhSO_2_), 7.66 (t, 2H, *J* = 7.6 Hz, PhSO_2_); ^13^C-NMR (CD_3_COCD_3_): *δ* 172.72, 142.67, 139.31, 132.76, 129.44, 128.72, 128.26, 128.07, 127.63, 75.45, 60.92, 52.52; ESI-MS (*m/z*): 336 [M + H]^+^. HRMS (ESI) *m/z* calcd. for C_16_H_17_NO_5_SNa [M + Na]^+^: 358.0725, found 358.0704.

*2-Hydroxy-3-phenylmethane sulfonylamino-3-phenyl-propionic acid methyl ester* (**7b**). Yield 59% (412 mg); ^1^H-NMR (CD_3_COCD_3_): *δ* 3.69 (s, 3H, OCH_3_), 4.01 (d, 1H, *J* = 13.2 Hz, CH_2_ in Bn), 4.13 (d, 1H, *J* = 14.0 Hz, CH_2_ in Bn), 4.44 (d, 1H, *J* = 4.0 Hz, 2-CH), 4.93 (d, 1H, *J* = 3.6 Hz, 3-CH), 7.19 (m, 2H, 3-Ph), 7.27 (m, 3H, 3-Ph), 7.39 (m, 3H, Ph in Bn), 7.52 (m, 2H, Ph in Bn); ^13^C-NMR (CD_3_COCD_3_): 172.92, 140.33, 131.73, 130.88, 129.21, 129.03, 128.85, 128.66, 128.61, 75.63, 61.10, 60.41, 52.56; ESI-MS (*m/z*): 350 [M + H]^+^. HRMS (ESI) *m/z* calcd. for C_17_H_19_NO_5_SNa [M + Na]^+^: 372.0882, found 372.0859.

*2-Hydroxy-3-(4-methyl benzenesulfonylamino)-3-phenyl-propionic acid methyl ester* (**7c**). Yield 64% (448 mg); ^1^H-NMR (CD_3_COCD_3_): *δ* 2.31 (s, 3H, CH_3_ in Tosyl), 3.60 (s, 3H, OCH_3_), 4.34 (d, 1H, *J* = 2.8 Hz, 2-CH), 4.84 (d, 1H, *J* = 3.6 Hz, 3-CH), 7.27 (m, 5H, 3′-Ph), 7.26 (m, 2H, Ph in Tosyl), 7.53 (d, 2H, *J* = 8.0 Hz, Ph in Tosyl); ^13^C-NMR (CD_3_COCD_3_): 172.73, 143.39, 139.45, 129.92, 128.70, 128.29, 127.96, 127.73, 75.45, 60.87, 52.50, 21.29; ESI-MS (*m/z*): 350 [M + H]^+^. HRMS (ESI) *m/z* calcd. for C_17_H_19_NO_5_SNa [M + Na]^+^: 372.0882, found 372.0869.

*2-Hydroxy-3-(4-methoxy benzenesulfonylamino)-3-phenyl-propionic acid methyl ester* (**7d**). Yield 59% (432 mg); ^1^H-NMR (CD_3_COCD_3_): *δ* 3.63 (s, 3H, 3′-CH_3_), 3.81 (s, 3H, OCH_3_), 4.33 (d, 1H, *J* = 4.0 Hz, 2-CH), 4.83 (d, 1H, *J* = 3.2 Hz, 3-CH), 6.85 (m, 2H, PhSO_2_), 7.15 (m, 3H, 3-Ph), 7.26 (m, 2H, 3-Ph), 7.57 (m, 2H, PhSO_2_); ^13^C-NMR (CD_3_COCD_3_): 172.74, 163.31, 139.38, 134.30, 129.78, 128.94, 128.70, 128.29, 114.52, 75.50, 60.86, 55.98, 52.53; ESI-MS (*m/z*): 366 [M + H]^+^. HRMS (ESI) *m/z* calcd. for C_17_H_19_NO_6_SNa [M + Na]^+^: 388.0831, found 388.0837.

*2-Hydroxy-3-(4-isopropyl benzenesulfonylamino)-3-phenyl-propionic acid methyl ester* (**7e**). Yield 59% (445 mg); ^1^H-NMR (CD_3_COCD_3_): *δ* 1.19 (d, 6H, *J* = 3.2 Hz, 2CH_3_ in *i*-PrPh), 2.89 (m, 1H, CH in *i*-PrPh), 3.61 (s, 3H, OCH_3_), 4.34 (d, 1H, *J* = 3.2 Hz, 2-CH), 4.85 (d, 1H, *J* = 2.8 Hz, 3-CH), 7.12 (m, 3H, 3-Ph), 7.19 (d, 2H, *J* = 8.4 Hz, PhSO_2_), 7.23 (m, 2H, 3-Ph), 7.40 (m, 2H, PhSO_2_); ^13^C-NMR (CD_3_COCD_3_): 172.74, 154.05, 139.99, 139.20, 128.66, 128.31, 127.97, 127.87, 127.38, 75.44, 60.91, 52.51, 34.74, 23.93; ESI-MS (*m/z*): 378 [M + H]^+^. HRMS (ESI) *m/z* calcd. for C_19_H_23_NO_5_SNa [M + Na]^+^: 400.1195, found 400.1180.

*2-Hydroxy-3-(4-fluoro benzenesulfonylamino)-3-phenyl-propionic acid methyl ester* (**7f**). Yield 49% (346 mg); ^1^H-NMR (CD_3_COCD_3_): *δ* 3.66 (s, 3H, OCH_3_), 4.35 (d, 1H, *J* = 2.8 Hz, 2-CH), 4.88 (d, 1H, *J* = 3.2 Hz, 3-CH), 7.08 (t, 2H, *J* = 8.8 Hz, PhSO_2_), 7.15 (m, 3H, 3-Ph), 7.26 (m, 2H, 3-Ph), 7.68 (m, 2H, PhSO_2_); ^13^C-NMR (CD_3_COCD_3_): 172.68, 166.54, 164.06, 138.94, 130.66, 130.57, 128.73, 128.37, 128.10, 116.41, 116.18, 75.41, 61.01, 52.53; ESI-MS (*m/z*): 354 [M + H]^+^. HRMS (ESI) *m/z* calcd. for C_16_H_16_FNO_5_SNa [M + Na]^+^: 376.0631, found 376.0618.

*2-Hydroxy-3-(4-trifluoromethyl benzenesulfonylamino)-3-phenyl-propionic acid methyl ester* (**7g**). Yield 19% (153 mg); ^1^H-NMR (CD_3_COCD_3_): *δ* 3.65 (s, 3H, OCH_3_), 4.37 (d, 1H, *J* = 3.2 Hz, 2-CH), 4.91 (d, 1H, *J* = 3.2 Hz, 3-CH), 7.11 (m, 3H, 3-Ph), 7.23 (m, 2H, 3-Ph), 7.66 (d, 2H, *J* = 8.4 Hz, PhSO_2_), 7.82 (d, 2H, *J* = 8.0 Hz, PhSO_2_); ^13^C-NMR (CD_3_COCD_3_): 172.62, 146.30, 138.65, 133.69, 133.37, 128.73, 128.54, 128.43, 128.16, 126.52, 126.48, 126.44, 125.90, 123.20, 75.33, 61.19, 52.51; ESI-MS (*m/z*): 404 [M + H]^+^. HRMS (ESI) *m/z* calcd. for C_17_H_16_F_3_NO_5_SNa [M + Na]^+^: 426.0599, found 426.0606.

*2-Hydroxy-3-(4-chloro benzenesulfonylamino)-3-phenyl-propionic acid methyl ester* (**7h**). Yield 32% (236 mg); ^1^H-NMR (CD_3_COCD_3_): *δ* 3.65 (s, 3H, OCH_3_), 4.36 (d, 1H, *J* = 3.2 Hz, 2-CH), 4.88 (d, 1H, *J* = 3.2 Hz, 3-CH), 7.15 (m, 3H, 3-Ph), 7.26 (m, 2H, 3-Ph), 7.35 (d, 2H, *J* = 8.8 Hz, PhSO_2_), 7.62 (d, 2H, *J* = 8.8 Hz, PhSO_2_); ^13^C-NMR (CD_3_COCD_3_): 172.65, 141.42, 138.98, 138.36, 129.49, 128.74, 128.39, 128.10, 75.38, 61.05, 52.51; ESI-MS (*m/z*): 370 [M + H]^+^. HRMS (ESI) *m/z* calcd. for C_16_H_16_ClNO_5_SNa [M + Na]^+^: 392.0335, found 392.0342.

*2-Hydroxy-3-(4-bromo benzenesulfonylamino)-3-phenyl-propionic acid methyl ester* (**7i**). Yield 35% (290 mg); ^1^H-NMR (CD_3_COCD_3_): *δ* 3.64 (s, 3H, OCH_3_), 4.36 (d, 1H, *J* = 3.2 Hz, 2-CH), 4.88 (d, 1H, *J* = 3.2 Hz, 3-CH), 7.16 (m, 3H, 3-Ph), 7.26 (m, 2H, 3-Ph), 7.51 (m, 4H, PhSO_2_); ^13^C-NMR (CD_3_COCD_3_): 172.65, 141.88, 138.98, 132.51, 129.60, 128.75, 128.40, 128.09, 126.87, 75.37, 61.06, 52.51; ESI-MS (*m/z*): 413 [M + H]^+^. HRMS (ESI) *m/z* calcd. For C_16_H_16_BrNO_5_SNa [M + Na]^+^: 435.9830, found 435.9807.

*2-Hydroxy-3-(2,4,6-trimethyl benzenesulfonylamino)-3-phenyl-propionic acid methyl ester* (**7j**). Yield 50% (377 mg); ^1^H-NMR (CD_3_COCD_3_): *δ* 2.23 (s, 3H, CH_3_ in PhSO_2_), 2.53 (s, 6H, CH_3_ in PhSO_2_), 3.45 (s, 3H, OCH_3_), 4.32 (d, 1H, *J* = 3.2 Hz, 2-CH), 4.72 (d, 1H, *J* = 3.2 Hz, 3-CH), 6.90 (s, 2H, PhSO_2_), 7.20 (m, 3H, 3-Ph), 7.31 (m, 2H, 3-Ph); ^13^C-NMR (CD_3_COCD_3_): 172.67, 142.56, 139.92, 139.49, 136.23, 132.43, 128.70, 128.11, 128.05, 75.18, 60.45, 52.37, 23.09, 20.74; ESI-MS (*m/z*): 378 [M + H]^+^. HRMS (ESI) *m/z* calcd. for C_19_H_23_NO_5_SNa [M + Na]^+^: 400.1195, found 400.1180.

#### 3.2.2. General Procedure for the Synthesis of **11a**–**j**

To a stirred solution of **7a**–**j** (0.45 mmol) and PPTs (113 mg, 0.045 mmol) in anhydrous toluene (8 mL) was added 2-methoxypropene (0.129 mL, 1.35 mmol). The reaction mixture was warmed to 85 °C , and further stirred for 2 h at this temperature. After cooled down to room temperature, the mixture was diluted with EtOAc (50 mL). The organic layer was washed with brine, dried over anhydrous Na_2_SO_4_ and filtered. The filtrate was evaporated and the residue was purified by silica gel column chromatography using petroleum ether/EtOAc (2/1) to obtain products **11a**–**j** as colorless liquids.

*2,2-Dimethyl-3-benzenesulfonyl-4-phenyl-oxazolidine-5-carboxylic acid methyl ester* (**11a**). Yield 97% (164 mg); ^1^H-NMR (CD_3_COCD_3_): *δ* 1.69 (s, 3H, *i*-Pr), 1.81 (s, 3H, *i*-Pr), 3.66 (s, 3H, OCH_3_), 4.62 (d, 1H, *J* = 4.8 Hz, 2-CH), 5.27 (d, 1H, *J* = 4.8 Hz, 3-CH), 7.22 (m, 3H, 3′-Ph), 7.32 (m, 2H, 3′-Ph), 7.42 (t, 2H, *J* = 7.6 Hz, *m*-PhSO_2_), 7.55 (t, 1H, *J* = 7.6 Hz, *p*-PhSO_2_), 7.62 (t, 2H, *J* = 7.6 Hz, *o*-PhSO_2_); ^13^C-NMR (CD_3_COCD_3_): 171.03, 142.02, 139.51, 133.41, 129.60, 129.14, 128.76, 128.46, 128.32, 100.78, 82.50, 66.19, 52.76, 27.31; ESI-MS (*m/z*): 376 [M + H]^+^. HRMS (ESI) *m/z* calcd. for C_19_H_21_NO_5_SNa [M + Na]^+^: 398.1038, found 398.1024.

*2,2-Dimethyl-3-phenylmethane sulfonyl-4-phenyl-oxazolidine-5-carboxylic acid methyl ester* (**11b**). Yield 92% (161 mg); ^1^H-NMR (CD_3_COCD_3_): *δ* 1.60 (s, 3H, *i*-Pr), 1.73 (s, 3H, *i*-Pr), 3.79 (s, 3H, OCH_3_), 3.79 (d, 1H, *J* = 14.0 Hz, CH_2_ in Bn), 4.10 (d, 1H, *J* = 13.6 Hz, CH_2_ in Bn), 4.79 (d, 1H, *J* = 4.0 Hz, 2-CH), 5.30 (d, 1H, *J* = 4.4 Hz, 3-CH), 7.25 (m, 2H, 3-Ph), 7.33 (m, 3H, 3-Ph), 7.40 (m, 1H, Ph in Bn), 7.48 (m, 2H, Ph in Bn), 7.58 (m, 2H, Ph in Bn); ^13^C-NMR (CD_3_COCD_3_): 171.57, 140.54, 131.98, 130.12, 129.58, 129.30, 129.14, 128.97, 100.28, 82.18, 65.65, 61.03, 52.88, 28.95, 27.74; ESI-MS (*m/z*): 390 [M + H]^+^; Anal. calcd. for C_20_H_23_NO_5_S: C, 61.68; H, 5.95; N, 3.60. Found: C, 61.73; H, 6.05; N, 3.52.

*2,2-Dimethyl-3-(4-methyl)benzenesulfonyl-4-phenyl-oxazolidine-5-carboxylic acid methyl ester* (**11c**). Yield 80% (140 mg); ^1^H-NMR (CD_3_COCD_3_): *δ* 1.68 (s, 3H, *i*-Pr), 1.79 (s, 3H, *i*-Pr), 2.36 (s, 3H, CH_3_ in tosyl), 3.66 (s, 3H, OCH_3_), 4.60 (d, 1H, *J* = 4.8 Hz, 2-CH), 5.24 (d, 1H, *J* = 4.8 Hz, 3-CH), 7.23 (m, 5H, 3′-Ph), 7.32 (m, 2H, Ph in tosyl), 7.50 (d, 2H, *J* = 8.4 Hz, Ph in tosyl); ^13^C-NMR (CD_3_COCD_3_): 171.04, 144.23, 139.71, 139.12, 130.07, 129.10, 128.62, 128.43, 128.42, 100.68, 82.48, 66.20, 52.75, 27.22, 21.37; ESI-MS (*m/z*): 390 [M + H]^+^. HRMS (ESI) *m/z* calcd. for C_20_H_23_NO_5_SNa [M + Na]^+^: 412.1195, found 412.1196. 

*2,2-Dimethyl-3-(4-methoxy)benzenesulfonyl-4-phenyl-oxazolidine-5-carboxylic acid methyl ester* (**11d**). Yield 99% (180 mg); ^1^H-NMR (CD_3_COCD_3_): *δ* 1.69 (s, 3H, *i*-Pr), 1.80 (s, 3H, *i*-Pr), 3.67 (s, 3H, 3-CH_3_), 3.85 (s, 3H, OCH_3_), 4.58 (d, 1H, *J* = 4.8 Hz, 2-CH), 5.21 (d, 1H, *J* = 4.8 Hz, 3-CH), 6.89 (m, 2H, PhSO_2_), 7.22 (m, 3H, 3-Ph), 7.29 (m, 2H, 3-Ph), 7.53 (m, 2H, PhSO_2_); ^13^C-NMR (CD_3_COCD_3_): 171.06, 163.73, 139.67, 133.52, 130.57, 129.08, 128.63, 128.38, 114.63, 100.65, 82.51, 66.12, 60.57, 56.09, 52.73, 27.15; ESI-MS (*m/z*): 406 [M + H]^+^; Anal. calcd. for C_20_H_23_NO_6_S: C, 59.24; H, 5.72; N, 3.45. Found: C, 59.21; H, 5.70; N, 3.49.

*2,2-Dimethyl-3-(4-isopropyl)benzenesulfonyl-4-phenyl-oxazolidine-5-carboxylic acid methyl ester* (**11e**). Yield 97% (182 mg); ^1^H-NMR (CD_3_COCD_3_): *δ* 1.22 (d, 6H, *J* = 7.4 Hz, 2CH_3_ in *i*-PrPh), 1.71 (s, 3H, *i*-Pr), 1.83 (s, 3H, *i*-Pr), 2.94 (m, 1H, CH in *i*-PrPh), 3.66 (s, 3H, OCH_3_), 4.59 (d, 1H, *J* = 4.8 Hz, 2-CH), 5.21 (d, 1H, *J* = 5.2 Hz, 3-CH), 7.19 (m, 3H, 3-Ph), 7.24 (d, 2H, *J* = 8.8 Hz, PhSO_2_), 7.26 (m, 2H, 3-Ph), 7.49 (d, 2H, *J* = 8.8 Hz, PhSO_2_); ^13^C-NMR (CD_3_COCD_3_): 170.14, 153.83, 138.44, 138.35, 128.18, 127.91, 127.68, 127.57, 126.60, 99.98, 81.68, 78.33, 65.24, 51.82, 33.87, 26.42, 23.03, 22.98; ESI-MS (*m/z*): 418 [M + H]^+^. HRMS (ESI) *m/z* calcd. for C_22_H_27_NO_5_SNa [M + Na]^+^: 440.1508, found 440.1501.

*2,2-Dimethyl-3-(4-fluoro)benzenesulfonyl-4-phenyl-oxazolidine-5-carboxylic acid methyl ester* (**11f**). Yield 81% (143 mg); ^1^H-NMR (CD_3_COCD_3_): *δ* 1.71 (s, 3H, *i*-Pr), 1.83 (s, 3H, *i*-Pr), 3.69 (s, 3H, OCH_3_), 4.62 (d, 1H, *J* = 4.8 Hz, 2-CH), 5.23 (d, 1H, *J* = 4.8 Hz, 3-CH), 7.14 (t, 2H, *J* = 8.8 Hz, PhSO_2_), 7.22 (m, 3H, 3-Ph), 7.29 (m, 2H, 3-Ph), 7.63 (m, 2H, PhSO_2_); ^13^C-NMR (CD_3_COCD_3_): 171.02, 166.85, 164.35, 139.09, 138.26, 131.43, 131.33, 129.17, 128.82, 128.60, 116.63, 116.40, 100.97, 82.52, 66.07, 52.77, 27.38; ESI-MS (*m/z*): 394 [M + H]^+^. HRMS (ESI) *m/z* calcd. for C_19_H_20_FNO_5_SNa [M + Na]^+^: 416.0944, found 416.0936.

*2,2-Dimethyl-3-(4-trifluoromethyl)benzenesulfonyl-4-phenyl-oxazolidine-5-carboxylic acid methyl ester* (**11g**). Yield 99% (197 mg); ^1^H-NMR (CD_3_COCD_3_): *δ* 1.74 (s, 3H, *i*-Pr), 1.87 (s, 3H, *i*-Pr), 3.69 (s, 3H, OCH_3_), 4.65 (d, 1H, *J* = 5.2 Hz, 2-CH), 5.26 (d, 1H, *J* = 4.8 Hz, 3-CH), 7.19 (m, 3H, 3-Ph), 7.26 (m, 2H, 3-Ph), 7.70 (d, 2H, *J* = 8.4 Hz, PhSO_2_), 7.76 (d, 2H, *J* = 8.0 Hz, PhSO_2_); ^13^C-NMR (CD_3_COCD_3_): 170.90, 145.58, 138.51, 134.14, 129.21, 129.17, 128.95, 128.78, 126.71, 126.68, 126.64, 126.60, 101.24, 82.49, 66.11, 52.79, 27.67; ESI-MS (*m/z*): 444 [M + H]^+^; Anal. calcd. for C_20_H_20_F_3_NO_5_S: C, 54.17; H, 4.55; N, 3.16. Found: C, 54.31; H, 4.51; N, 3.22.

*2,2-Dimethyl-3-(4-chloro)benzenesulfonyl-4-phenyl-oxazolidine-5-carboxylic acid methyl ester* (**11h**). Yield 93% (171 mg); ^1^H-NMR (CD_3_COCD_3_): *δ* 1.71 (s, 3H, *i*-Pr), 1.83 (s, 3H, *i*-Pr), 3.69 (s, 3H, OCH_3_), 4.63 (d, 1H, *J* = 5.2 Hz, 2-CH), 5.24 (d, 1H, *J* = 5.2 Hz, 3-CH), 7.24 (m, 3H, 3-Ph), 7.29 (m, 2H, 3-Ph), 7.41 (d, 2H, *J* = 8.8 Hz, PhSO_2_), 7.56 (d, 2H, *J* = 8.0 Hz, PhSO_2_); ^13^C-NMR (CD_3_COCD_3_): 170.99, 140.73, 139.10, 139.02, 130.15, 129.69, 129.21, 128.83, 128.66, 101.01, 82.50, 66.11, 52.79, 27.46; ESI-MS (*m/z*): 410 [M + H]^+^. HRMS (ESI) *m/z* calcd for C_19_H_20_ClNO_5_SNa [M + Na]^+^: 432.0648, found 432.0653.

*2,2-Dimethyl-3-(4-bromo)benzenesulfonyl-4-phenyl-oxazolidine-5-carboxylic acid methyl ester* (**11i**). Yield 99% (202 mg); ^1^H-NMR (CD_3_COCD_3_): *δ* 1.71 (s, 3H, *i*-Pr), 1.83 (s, 3H, *i*-Pr), 3.69 (s, 3H, OCH_3_), 4.63 (d, 1H, *J* = 5.2 Hz, 2-CH), 5.23 (d, 1H, *J* = 4.8 Hz, 3-CH), 7.24 (m, 3H, 3-Ph), 7.29 (m, 2H, 3-Ph), 7.49 (d, 2H, *J* = 8.8 Hz, PhSO_2_), 7.57 (d, 2H, *J* = 8.8 Hz, PhSO_2_); ^13^C-NMR (CD_3_COCD_3_): 170.96, 141.17, 138.99, 132.70, 130.20, 129.21, 128.81, 128.65, 127.67, 101.00, 82.48, 66.10, 52.78, 27.46; ESI-MS (*m/z*): 455 [M + H]^+^. HRMS (ESI) *m/z* calcd. for C_19_H_20_BrNO_5_SNa [M + Na]^+^: 476.0143, found 476.0156.

*2,2-Dimethyl-3-(2,4,6-trimethyl)benzenesulfonyl-4-phenyl-oxazolidine-5-carboxylic acid methyl ester* (**11j**). Yield 81% (152 mg); ^1^H-NMR (CD_3_COCD_3_): *δ* 1.82 (s, 3H, *i*-Pr), 1.92 (s, 3H, *i*-Pr), 2.10 (s, 3H, CH_3_ in PhSO_2_), 2.51 (s, 6H, CH_3_ in PhSO_2_), 3.74 (s, 3H, OCH_3_), 4.47 (d, 1H, *J* = 5.6 Hz, 2-CH), 4.99 (d, 1H, *J* = 6.0 Hz, 3-CH), 6.63 (s, 2H, PhSO_2_), 7.01 (m, 5H, 3-Ph); ^13^C-NMR (CD_3_COCD_3_): 171.11, 144.03, 140.69, 138.39, 134.00, 132.53, 128.67, 128.25, 127.46, 102.10, 83.12, 66.06, 52.72, 27.12, 23.21, 20.67; ESI-MS (*m/z*): 418 [M + H]^+^; Anal. calcd. for C_22_H_27_NO_5_S: C, 63.29; H, 6.52; N, 3.35. Found: C, 63.42; H, 6.48; N, 3.39. 

#### 3.2.3. General Procedure for the Synthesis of **12a**–**j**

To a stirred solution of **11a**–**j** (0.437 mmol) in a mixture of solvent (THF:H_2_O = 6 mL:2 mL) at 0 °C was added LiOH•H_2_O (37 mg, 0.87 mmol). The resulting mixture was warmed to room temperature and further stirred for 1 h. Then the pH of the mixture was adjusted to 2~3 by adding 1N HCl solution. After extracted with CH_2_Cl_2_ three times, the combined organic phase was dried over anhydrous Na_2_SO_4_ and evaporated to afford the colorless liquid products **12a**–**j**.

*2,2-Dimethyl-3-benzenesulfonyl-4-phenyl-oxazolidine-5-carboxylic acid* (**12a**). Yield 97% (154 mg); ^1^H-NMR (CD_3_COCD_3_): *δ* 1.71 (s, 3H, *i*-Pr), 1.83 (s, 3H, *i*-Pr), 4.56 (d, 1H, *J* = 4.8 Hz, 2-CH), 5.25 (d, 1H, *J* = 4.4 Hz, 3-CH), 7.21 (m, 3H, 3′-Ph), 7.31 (m, 2H, 3′-Ph), 7.39 (t, 2H, *J* = 7.6 Hz, *m*-PhSO_2_), 7.53 (t, 1H, *J* = 7.6 Hz, *p*-PhSO_2_), 7.60 (t, 2H, *J* = 7.6 Hz, *o*-PhSO_2_); ^13^C-NMR (CD_3_COCD_3_) *δ* 171.48, 142.04, 139.68, 133.34, 129.55, 129.12, 128.69, 128.50, 128.305, 100.72, 82.50, 66.24, 27.41; ESI-MS (*m/z*): 362 [M + H]^+^. HRMS (ESI) *m/z* calcd. for C_18_H_19_NO_5_SNa [M + Na]^+^: 384.0882, found 384.0888.

*2,2-Dimethyl-3-phenylmethane sulfonyl-4-phenyl-oxazolidine-5-carboxylic acid* (**12b**). Yield 97% (160 mg); ^1^H-NMR (CD_3_COCD_3_): *δ* 1.61 (s, 3H, *i*-Pr), 1.76 (s, 3H, *i*-Pr), 3.79 (d, 1H, *J* = 14.0 Hz, CH_2_ in Bn), 4.10 (d, 1H, *J* = 14.0 Hz, CH_2_ in Bn), 4.76 (d, 1H, *J* = 4.0 Hz, 2-CH), 5.30 (d, 1H, *J* = 4.0 Hz, 3-CH), 7.24 (m, 3H, 3-Ph), 7.33 (m, 2H, 3-Ph), 7.40 (m, 2H, Ph in Bn), 7.59 (m, 3H, Ph in Bn); ^13^C-NMR (CD_3_COCD_3_) *δ* 172.03, 140.75, 131.99, 131.74, 130.93, 130.15, 129.56, 129.24, 129.15, 129.04, 129.00, 128.83, 128.62, 128.55, 100.23, 82.12, 75.05, 65.72, 27.81; ESI-MS (*m/z*): 376 [M + H]^+^. HRMS (ESI) *m/z* calcd. for C_19_H_21_NO_5_SNa [M + Na]^+^: 398.1038, found 398.1031.

*2,2-Dimethyl-3-(4-methyl)benzenesulfonyl-4-phenyl-oxazolidine-5-carboxylic acid* (**12c**). Yield 96% (158 mg); ^1^H-NMR (CD_3_COCD_3_): *δ* 1.70 (s, 3H, *i*-Pr), 1.82 (s, 3H, *i*-Pr), 2.35 (s, 3H, CH_3_ in Tosyl), 4.55 (d, 1H, *J* = 5.2 Hz, 2-CH), 5.22 (d, 1H, *J* = 5.2 Hz, 3-CH), 7.22 (m, 5H, Ph in Tosyl), 7.30 (m, 2H, Ph in Tosyl), 7.48 (d, 2H, Ph in Tosyl); ^13^C-NMR (CD_3_COCD_3_) *δ* 171.47, 144.15, 139.91, 139.12, 130.04, 129.09, 128.55, 128.45, 128.44, 100.63, 82.49, 66.23, 27.31, 21.37; ESI-MS (*m/z*): 376 [M + H]^+^. HRMS (ESI) *m/z* calcd. for C_19_H_21_NO_5_SNa [M + Na]^+^: 398.1038, found 398.1031.

*2,2-Dimethyl-3-(4-methoxy)benzenesulfonyl-4-phenyl-oxazolidine-5-carboxylic acid* (**12d**). Yield 99% (170 mg); ^1^H-NMR (CD_3_COCD_3_): *δ* 1.74 (s, 3H, *i*-Pr), 1.88 (s, 3H, *i*-Pr), 3.70 (s, 3H, 3-CH_3_), 4.67 (d, 1H, *J* = 4.8 Hz, 2-CH), 5.29 (d, 1H, *J* = 5.2 Hz, 3-CH), 7.21 (m, 3H, 3-Ph), 7.29 (m, 2H, 3-Ph), 7.80 (d, 2H, *J* = 9.2 Hz, PhSO_2_), 8.19 (d, 2H, *J* = 8.8 Hz, PhSO_2_); ^13^C-NMR (CD_3_COCD_3_) *δ* 170.87, 150.65, 147.26, 138.47, 129.81, 129.30, 129.06, 128.9, 124.68, 101.32, 82.43, 66.12, 52.85, 27.78; ESI-MS (*m/z*): 392 [M + H]^+^. HRMS (ESI) *m/z* calcd. for C_19_H_21_NO_6_SNa [M + Na]^+^: 414.0987, found 414.0998.

*2,2-Dimethyl-3-(4-isopropyl)benzenesulfonyl-4-phenyl-oxazolidine-5-carboxylic acid* (**12e**). Yield 95% (168 mg); ^1^H-NMR (CD_3_COCD_3_): *δ* 1.21 (d, 3H, *J* = 1.6 Hz, CH_3_ in *i*-PrPh), 1.22 (d, 3H, *J* = 1.2 Hz, CH_3_ in *i*-PrPh), 1.74 (s, 3H, CH_3_ in *i*-Pr), 1.85 (s, 3H, CH_3_ in *i*-Pr), 2.92 (m, 1H, CH in *i*-PrPh), 4.53 (d, 1H, *J* = 5.2 Hz, 2-CH), 5.19 (d, 1H, *J* = 5.2 Hz, 3-CH), 7.16 (m, 3H, 3-Ph), 7.20 (d, 2H, *J* = 8.4 Hz, PhSO_2_), 7.23 (m, 2H, 3-Ph), 7.46 (m, 2H, PhSO_2_); ^13^C-NMR (CD_3_COCD_3_) *δ* 171.48, 154.64, 139.36, 139.33, 129.06, 128.64, 128.55, 128.54, 127.45, 100.83, 82.59, 66.20, 34.76, 27.46, 23.95, 23.86; ESI-MS (*m/z*): 404 [M + H]^+^. HRMS (ESI) *m/z* calcd. for C_21_H_25_NO_5_SNa [M + Na]^+^: 426.1351, found 426.1339.

*2,2-Dimethyl-3-(4-fluoro)benzenesulfonyl-4-phenyl-oxazolidine-5-carboxylic acid* (**12f**). Yield 81% (135 mg); ^1^H-NMR (CD_3_COCD_3_): *δ* 1.73 (s, 3H, *i*-Pr), 1.84 (s, 3H, *i*-Pr), 4.58 (d, 1H, *J* = 5.2 Hz, 2-CH), 5.23 (d, 1H, *J* = 5.2 Hz, 3-CH), 7.12 (t, 2H, *J* = 8.8 Hz, PhSO_2_), 7.22 (m, 3H, 3-Ph), 7.28 (m, 2H, 3-Ph), 7.63 (m, 2H, PhSO_2_); ^13^C-NMR (CD_3_COCD_3_) *δ* 171.45, 166.83, 164.32, 139.32, 138.29, 138.26, 131.43, 131.33, 129.16, 128.76, 128.62, 116.60, 116.38, 100.93, 82.50, 66.12, 27.47; ESI-MS (*m/z*): 380 [M + H]^+^. HRMS (ESI) *m/z* calcd. for C_18_H_18_FNO_5_SNa [M + Na]^+^: 402.0787, found 402.0782.

*2,2-Dimethyl-3-(4-trifluoromethyl)benzenesulfonyl-4-phenyl-oxazolidine-5-carboxylic acid* (**12g**). Yield 78% (147 mg); ^1^H-NMR (CD_3_COCD_3_): *δ* 1.77 (s, 3H, *i*-Pr), 1.88 (s, 3H, *i*-Pr), 4.64 (d, 1H, *J* = 5.2 Hz, 2-CH), 5.29 (d, 1H, *J* = 4.8 Hz, 3-CH), 7.21 (m, 3H, 3-Ph), 7.30 (m, 2H, 3-Ph), 7.81 (d, 2H, *J* = 8.8 Hz, PhSO_2_), 8.18 (d, 2H, *J* = 9.2 Hz, PhSO_2_); ^13^C-NMR (CD_3_COCD_3_) *δ* 171.32, 150.62, 147.27, 138.73, 129.82, 129.28, 128.99, 128.89, 124.68, 101.26, 82.38, 66.18, 27.83; ESI-MS (*m/z*): 430 [M + H]^+^. HRMS (ESI) *m/z* calcd. for C_19_H_18_F_3_NO_5_SNa [M + Na]^+^: 452.0755, found 452.0737.

*2,2-Dimethyl-3-(4-chloro)benzenesulfonyl-4-phenyl-oxazolidine-5-carboxylic acid* (**12h**). Yield 100% (173 mg); ^1^H-NMR (CD_3_COCD_3_): *δ* 1.73 (s, 3H, *i*-Pr), 1.84 (s, 3H, *i*-Pr), 4.59 (d, 1H, *J* = 5.2 Hz, 2-CH), 5.24 (d, 1H, *J* = 4.8 Hz, 3-CH), 7.23 (m, 3H, 3-Ph), 7.29 (m, 2H, 3-Ph), 7.39 (d, 2H, *J* = 8.8 Hz, PhSO_2_), 7.56 (d, 2H, *J* = 8.0 Hz, PhSO_2_); ^13^C-NMR (CD_3_COCD_3_) *δ* 171.40, 140.74, 139.26, 139.04, 130.14, 129.66, 129.19, 128.76, 128.67, 100.95, 82.46, 66.14, 27.54; ESI-MS (*m/z*): 396 [M + H]^+^. HRMS (ESI) *m/z* calcd. for C_18_H_18_ClNO_5_SNa [M + Na]^+^: 418.0492, found 418.0504.

*2,2-Dimethyl-3-(4-bromo)benzenesulfonyl-4-phenyl-oxazolidine-5-carboxylic acid* (**12i**). Yield 99% (191 mg); ^1^H-NMR (CD_3_COCD_3_): *δ* 1.73 (s, 3H, *i*-Pr), 1.84 (s, 3H, *i*-Pr), 4.59 (d, 1H, *J* = 4.8 Hz, 2-CH), 5.23 (d, 1H, *J* = 5.2 Hz, 3-CH), 7.23 (m, 3H, 3-Ph), 7.29 (m, 2H, 3-Ph), 7.48 (d, 2H, *J* = 8.8 Hz, PhSO_2_), 7.55 (d, 2H, *J* = 8.8 Hz, PhSO_2_); ^13^C-NMR (CD_3_COCD_3_) *δ* 171.40, 141.20, 139.22, 132.67, 130.20, 129.20, 128.73, 128.70, 127.62, 100.93, 82.45, 66.14, 27.54; ESI-MS (*m/z*): 439 [M + H]^+^. HRMS (ESI) *m/z* calcd. for C_18_H_18_BrNO_5_SNa [M + Na]^+^: 461.9987, found 461.9997.

*2,2-Dimethyl-3-(2,4,6-trimethyl)benzenesulfonyl-4-phenyl-oxazolidine-5-carboxylic acid* (**12j**). Yield 99% (175 mg); ^1^H-NMR (CD_3_COCD_3_): *δ* 1.85 (s, 3H, *i*-Pr), 1.93 (s, 3H, *i*-Pr), 2.09 (s, 3H, CH_3_ in PhSO_2_), 2.52 (s, 6H, CH_3_ in PhSO_2_), 4.44 (d, 1H, *J* = 5.6 Hz, 2-CH), 5.02 (d, 1H, *J* = 5.6 Hz, 3-CH), 6.63 (s, 2H, PhSO_2_), 7.01 (m, 5H, 3-Ph); ^13^C-NMR (CD_3_COCD_3_) *δ* 171.58, 143.99, 140.68, 138.84, 134.01, 132.52, 128.65, 128.15, 127.39, 102.05, 83.10, 66.04, 27.20, 23.21, 20.68. ESI-MS (*m/z*): 404 [M + H]^+^. HRMS (ESI) *m/z* calcd for C_21_H_25_NO_5_SNa [M + Na]^+^: 426.1351, found 426.1345.

#### 3.2.4. General Procedure for the Synthesis of **13a**–**j**

To a solution of anhydrous toluene (60 mL) were added **12a**–**j** (0.49 mmol), 7,10-ditroc-10-DAB (0.23 g, 0.24 mmol), DCC (0.15 g, 0.75 mmol) and DMAP (15 mg, 0.13 mmol). The resulting mixture was stirred at 85 °C for 3 h. After the completion, the reaction mixture was washed with brine (30 mL × 3), dried over Na_2_SO_4_, filtered and concentrated *in vacuo*. The obtained residue was purified by silica gel flash chromatography column (petroleum ether/ethyl acetate: 4/1) to afford **13a**–**j** as white solids.

*7,10-Di(2,2,2-trichloroethyloxycarbonyl)-10-deacetylbaccatin III-13-O-[2,2-dimethyl-3-benzene sulfonyl-4-phenyl-oxazolidine-5-carboxylate]* (**13a**). Yield 96% (285 mg); mp 155–157 °C; ^1^H-NMR (CDCl_3_): *δ* 1.17 (s, 3H, 17-CH_3_), 1.24 (s, 3H, 16-CH_3_), 1.82 and 1.87 (2s, 6H, *i*-Pr), 1.95 (s, 3H, 19-CH_3_), 1.97 (s, 3H, 18-CH_3_), 1.99 (s, 3H, OAc), 2.13 (m, 2H, 14-CH_2_), 2.03 and 2.59 (2m, 2H, 6-CH_2_), 3.87 (d, 1H, *J* = 7.2 Hz, 3-CH), 4.11 and 4.27 (2d, 2H, *J* = 8.6 Hz, 20-CH_2_), 4.50 (d, 1H, *J* = 6.0 Hz, 2′-CH), 4.60 and 4.91 (2d, 2H, *J* = 12.0 Hz, Troc), 4.78 (s, 2H, Troc), 4.90 (d, 1H, *J* = 7.6 Hz, 5-CH), 5.23 (d, 1H, *J* = 6.4 Hz, 3′-CH), 5.56 (dd, 1H, *J* = 10.8, 7.2 Hz, 7-CH), 5.65 (d, 1H, *J* = 7.2 Hz, 2-CH), 6.21 (t, 1H, *J* = 8.4 Hz, 13-CH), 6.23 (s, 1H, 10-CH), 7.15 (m, 2H, 3′-Ph), 7.21 (m, 3H, 3′-Ph), 7.26 (t, 2H, *J* = 7.6 Hz, *m*-PhSO_2_), 7.41 (t, 1H, *J* = 7.6 Hz, *p*-PhSO_2_), 7.46 (d, 2H, *J* = 7.2 Hz, *o*-PhSO_2_), 7.47 (t, 2H, *J* = 7.6 Hz, *m*-OBz), 7.62 (t, 1H, *J* = 7.6 Hz, *p*-OBz), 8.01 (d, 2H, *J* = 7.2 Hz, *o*-OBz). ^13^C-NMR (CDCl_3_) *δ* 200.65, 170.14, 169.78, 166.84, 153.21, 142.48, 140.58, 136.95, 133.87, 132.41, 132.02, 130.05, 129.00, 128.67, 128.58, 128.46, 128.33, 128.00, 127.50, 100.95, 94.19, 83.68, 81.99, 80.43, 79.01, 78.88, 74.23, 71.49, 65.27, 58.46, 56.09, 49.21, 46.90, 43.07, 35.38, 33.91, 33.17, 28.73, 27.18, 26.23, 25.60, 24.92, 21.58, 21.03, 18.43, 14.81, 10.72. Anal. calcd. for C_53_H_55_Cl_6_NO_18_S: C, 51.39; H, 4.48; N, 1.13. Found: C, 51.52; H, 4.51; N, 1.15.

*7,10-Di(2,2,2-trichloroethyloxycarbonyl)-10-deacetylbaccatin III-13-O-[2,2-dimethyl-3-phenyl methane sulfonyl-4-phenyl-oxazolidine-5-carboxylate]* (**13b**). Yield 94% (282 mg); mp 154–156 °C; ^1^H-NMR (CDCl_3_): *δ* 1.20 (s, 3H, 17-CH_3_), 1.28 (s, 3H, 16-CH_3_), 1.61 (s, 3H, 19-CH_3_), 1.84 and 1.85 (2s, 6H, *i*-Pr), 2.08 (s, 3H, 18-CH_3_), 2.10 (s, 3H, OAc), 2.22 (m, 2H, 14-CH_2_), 2.04 and 2.62 (2m, 2H, 6-CH_2_), 3.73 (2d, 2H, *J* = 13.6 Hz, 3″-CH_2_), 3.93 (d, 1H, *J* = 7.2 Hz, 3-CH), 4.15 and 4.32 (2d, 2H, *J* = 8.4 Hz, 20-CH_2_), 4.70 (d, 1H, *J* = 5.2 Hz, 2′-CH), 4.62 and 4.94 (2d, 2H, *J* = 12.0 Hz, Troc), 4.80 (dd, 2H, *J* = 12.8, 12.0 Hz, Troc), 4.94 (d, 1H, *J* = 9.2 Hz, 5-CH), 5.29 (d, 1H, *J* = 5.2 Hz, 3′-CH), 5.61 (dd, 1H, *J* = 10.6, 7.0 Hz, 2-CH), 5.69 (d, 1H, *J* = 7.2 Hz, 2-CH), 6.27 (t, 1H, *J* = 8.6 Hz, 13-CH), 6.27 (s, 1H, 10-CH), 7.21 (m, 2H, 3′-Ph), 7.35 (m, 3H, 3′-Ph), 751 (t, 2H, *J* = 8.0 Hz, *m*-OBz), 7.51 (m, 2H, 3″-Ph), 7.56 (m, 2H, 3″-Ph), 7.65 (t, 1H, *J* = 7.6 Hz, *p*-OBz), 8.05 (d, 2H, *J* = 7.6 Hz, *o*-OBz). ^13^C-NMR (CDCl_3_) *δ* 200.67, 171.18, 170.18, 170.04, 166.86, 153.22, 153.19, 142.43, 138.48, 133.91, 132.07, 130.99, 130.07, 129.21, 129.14, 128.99, 128.80, 128.71, 128.59, 128.50, 128.17, 100.50, 94.19, 83.70, 81.67, 80.48, 79.02, 78.91, 74.24, 71.66, 64.89, 61.36, 60.41, 56.10, 49.31, 46.94, 43.10, 35.45, 33.83, 33.19, 29.70, 27.98, 27.77, 26.23, 25.57, 24.89, 2, 21.65, 21.06, 21.03, 14.96, 14.21, 10.74. Anal. calcd. for C_54_H_57_Cl_6_NO_18_S: C, 51.77; H, 4.59; N, 1.12. Found: C, 51.88; H, 4.65; N, 1.18.

*7,10-Di(2,2,2-trichloroethyloxycarbonyl)-10-deacetylbaccatin III-13-O-[2,2-dimethyl-3-(4-methyl) benzene sulfonyl-4-phenyl-oxazolidine-5-carboxylate]* (**13c**). Yield 85% (255 mg); mp 154–156 °C; ^1^H-NMR (CDCl_3_): *δ* 1.19 (s, 3H, 17-CH_3_), 1.27 (s, 3H, 16-CH_3_), 1.84 and 1,87 (2s, 6H, *i*-Pr), 1.94 (s, 3H, 19-CH_3_), 1.99 (s, 3H, 18-CH_3_), 2.00 (s, 3H, OAc), 2.16 (m, 2H, 14-CH_2_), 2.36 (s, 3H, 4″-CH_3_), 2.05 and 2.61 (2m, 2H, 6-CH_2_), 3.89 (d, 1H, *J* = 6.8 Hz, 3-CH), 4.13 and 4.29 (2d, 2H, *J* = 8.4 Hz, 20-CH_2_), 4.52 (d, 1H, *J* = 6.8 Hz, 2′-CH), 4.62 and 4.93 (2d, 2H, *J* = 12.0 Hz, Troc), 4.80 (dd, 2H, *J* = 14.4, 12.0 Hz, Troc), 4.92 (d, 1H, *J* = 7.6 Hz, 5-CH), 5.21 (d, 1H, *J* = 6.8 Hz, 3′-CH), 5.58 (dd, 1H, *J* = 10.8, 7.2 Hz, 7-CH), 5.67 (d, 1H, *J* = 7.2 Hz, 2-CH), 6.22 (t, 1H, *J* = 8.8 Hz, 13-CH), 6.25 (s, 1H, 10-CH), 7.07 (d, 2H, *J* = 8.4 Hz, *m*-PhSO_2_), 7.20 (m, 2H, 3′-Ph), 7.26 (m, 3H, 3′-Ph), 7.39 (d, 2H, *J* = 8.4 Hz, *o*-PhSO_2_), 7.50 (t, 2H, *J* = 7.6 Hz, *m*-OBz), 7.65 (t, 1H, *J* = 7.4 Hz, *p*-OBz), 8.04 (d, 2H, *J* = 7.6 Hz, *o*-OBz). ^13^C-NMR (CDCl_3_) *δ* 200.65, 171.15, 169.81, 166.85, 153.21, 153.20, 143.32, 142.51, 137.57, 133.88, 131.99, 130.06, 129.10, 128.99, 130.06, 129.10, 128.99, 128.68, 128.53, 128.16, 127.92, 100.81, 94.21, 94.19, 83.67, 81.94, 80.42, 79.02, 78.90, 74.23, 71.45, 65.33, 60.39, 56.08, 46.90, 43.07, 35.37, 33.10, 33.17, 29.70, 28.90, 26.99, 26.23, 25.51, 24.82, 21.57, 21.44, 21.03, 14.78, 14.20, 10.72. Anal. calcd. for C_54_H_57_Cl_6_NO_18_S: C, 51.77; H, 4.59; N, 1.12. Found: C, 51.93; H, 4.63; N, 1.16.

*7,10-Di(2,2,2-trichloroethyloxycarbonyl)-10-deacetylbaccatin III-13-O-[2,2-dimethyl-3-(4-methoxy) benzene sulfonyl-4-phenyl-oxazolidine-5-carboxylate]* (**13d**). Yield 95% (288 mg); mp 159–161 °C; ^1^H-NMR (CDCl_3_): *δ* 1.19 (s, 3H, 17-CH_3_), 1.26 (s, 3H, 16-CH_3_), 1.83 and 1.87 (2s, 6H, *i*-Pr), 1.94 (s, 3H, 19-CH_3_), 1.99 (s, 3H, 18-CH_3_), 2.01 (s, 3H, OAc), 2.15 (m, 2H, 14-CH_2_), 2.05 and 2.61 (2m, 2H, 6-CH_2_), 3.82 (s, 3H, OCH_3_), 3.89 (d, 1H, *J* = 6.8 Hz, 3-CH), 4.13 and 4.28 (2d, 2H, *J* = 8.4 Hz, 20-CH_2_), 4.51 (d, 1H, *J* = 6.8 Hz, 2′-CH), 4.62 and 4.93 (2d, 2H, *J* = 11.6 Hz, Troc), 4.80 (dd, 2H, *J* = 14.0, 12.0 Hz, Troc), 4.92 (d, 1H, *J* = 8.0 Hz, 5-CH), 5.20 (d, 1H, *J* = 6.4 Hz, 3′-CH), 5.58 (dd, *J* = 10.8, 7.2 Hz, 7-CH), 5.67 (d, 1H, *J* = 7.6 Hz, 2-CH), 6.22 (t, 1H, *J* = 9.2 Hz, 13-CH), 6.25 (s, 1H, 10-CH), 6.72 (d, 2H, *J* = 8.8 Hz, *o*-3″-PhOCH_3_), 7.23 (m, 5H, 3′-Ph), 7.41 (d, *J* = 9.2 Hz, *m*-3″-PhOCH_3_), 7.50 (t, 2H, *J* = 7.6 Hz, *m*-OBz), 7.64 (t, 1H, *J* = 7.6 Hz, *p*-OBz), 8.03 (d, 2H, *J* = 7.6 Hz, *o*-OBz). ^13^C-NMR (CDCl_3_) *δ* 200.66, 171.15, 170.16, 169.86, 166.82, 162.70, 153.21, 153.19, 142.51, 137.31, 133.87, 132.00, 132.19, 132.00, 130.05, 129.75, 129.01, 128.67, 128.55, 128.20, 127.93, 113.64, 100.84, 94.21, 94.19, 83.67, 81.98, 80.41, 79.02, 78.88, 74.24, 71.45, 65.28, 60.39, 56.08, 55.58, 49.23, 46.90, 43.07, 35.39, 33.86, 33.17, 28.89, 26.96, 26.23, 25.59, 24.90, 21.57, 21.04, 14.80, 14.20, 10.72. Anal. calcd. for C_54_H_57_Cl_6_NO_19_S: C, 51.12; H, 4.53; N, 1.10. Found: C, 51.33; H, 4.55; N, 1.14. 

*7,10-Di(2,2,2-trichloroethyloxycarbonyl)-10-deacetylbaccatin III-13-O-[2,2-dimethyl-3-(4-isopropyl) benzene sulfonyl-4-phenyl-oxazolidine-5-carboxylate]* (**13e**). Yield 87% (267 mg); mp 165–167 °C; ^1^H-NMR (CDCl_3_): *δ* 1.18 (s, 3H, 17-CH_3_), 1.22 (d, 3H, *J* = 1.6 Hz, 2″-CH_3_), 1.24 (d, 3H, *J* = 1.6 Hz, 2″-CH_3_), 1.26 (s, 3H, 16-CH_3_), 1.83 and 1.89 (2s, 6H, *i*-Pr), 1.96 (s, 3H, 19-CH_3_), 1.99 (s, 3H, 18-CH_3_), 2.00 (s, 3H, OAc), 2.15 (m, 2H, 14-CH_2_), 2.05 and 2.61 (2m, 2H, 6-CH_2_), 2.89 (m, 1H, 2″-CH), 3.89 (d, 1H, *J* = 7.2 Hz, 3-CH), 4.12 and 4.28 (2d, 2H, *J* = 8.4 Hz, 20-CH_2_), 4.51 (d, 1H, *J* = 6.4 Hz, 2′-CH), 4.62 and 4.93 (2d, 2H, *J* = 12.0 Hz, Troc), 4.80 (dd, 2H, *J* = 14.4, 12.0 Hz, Troc), 4.92 (d, 1H, *J* = 8.0 Hz, 5-CH), 5.24 (m, 1H, 3′-CH), 5.58 (dd, 1H, *J* = 10.8, 7.2 Hz, 7-CH), 5.66 (d, 1H, *J* = 7.2 Hz, 2-CH), 6.22 (t, 1H, *J* = 9.2 Hz, 13-CH), 6.24 (s, 1H, 10-CH), 7.08 (d, 2H, *J* = 8.0 Hz, 3″-Ph), 7.14 (m, 2H, 3′-Ph), 7.20 (m, 3H, 3′-Ph), 7.39 (d, 2H, *J* = 8.8 Hz, 3″-Ph), 7.49 (t, 2H, *J* = 7.6 Hz, *m*-OBz), 7.64 (t, 1H, *J* = 7.6 Hz, *p*-OBz), 8.03 (d, 2H, *J* = 7.6 Hz, *o*-OBz). ^13^C-NMR (CDCl_3_) *δ* 200.66, 171.15, 170.15, 169.88, 166.82, 153.91, 153.20, 153.19, 142.51, 137.01, 133.87, 131.99, 130.05, 129.01, 128.67, 128.47, 128.18, 127.97, 127.73, 126.52, 100.94, 94.22, 94.20, 83.67, 82.01, 80.41, 79.01, 78.88, 74.24, 71.46, 65.30, 60.39, 56.07, 53.43, 46.89, 43.07, 35.39, 34.09, 33.16, 28.82, 27.06, 26.23, 23.67, 23.58, 21.55, 21.03, 14.81, 14.20, 10.72. Anal. calcd. for C_56_H_61_Cl_6_NO_18_S: C, 52.51; H, 4.80; N, 1.09. Found: C, 52.75; H, 4.88; N, 1.15.

*7,10-Di(2,2,2-trichloroethyloxycarbonyl)-10-deacetylbaccatin III-13-O-[2,2-dimethyl-3-(4-fluoro) benzene sulfonyl-4-phenyl-oxazolidine-5-carboxylate]* (**13f**). Yield 89% (268 mg); mp 166–168 °C; ^1^H-NMR (CDCl_3_): *δ* 1.19 (s, 3H, 17-CH_3_), 1.27 (s, 3H, 16-CH_3_), 1.84 and 1.90 (2s, 6H, *i*-Pr), 1.97 (s, 3H, 19-CH_3_), 2.01 (s, 3H, 18-CH_3_), 2.04 (s, 3H, OAc), 2.16 (m, 2H, 14-CH_2_), 2.05 and 2.62 (2m, 2H, 6-CH_2_), 3.90 (d, 1H, *J* = 6.8 Hz, 3-CH), 4.13 and 4.29 (2d, 2H, *J* = 8.8 Hz, 20-CH_2_), 4.52 (d, 1H, *J* = 6.4 Hz, 2′-CH), 4.62 and 4.94 (2d, 2H, *J* = 11.6 Hz, Troc), 4.80 (dd, 2H, *J* = 13.0, 11.8 Hz, Troc), 4.93 (d, 1H, *J* = 9.2 Hz, 5-CH), 5.25 (d, 1H, *J* = 6.4 Hz, 3′-CH), 5.59 (dd, 1H, *J* = 10.8, 7.2 Hz, 5-CH), 5.67 (d, 1H, *J* = 7.2 Hz, 2-CH), 6.24 (t, 1H, *J* = 8.4 Hz, 13-CH), 6.25 (s, 1H, 10-CH), 6.90 (t, 2H, *J* = 8.4 Hz, -PhF), 7.20 (m, 4H, 3′-Ph), 7.26 (m, 1H, 3′-Ph), 7.45 (m, 2H, -PhF), 7.49 (t, 2H, *J* = 7.6 Hz, *m*-OBz), 7.64 (t, 1H, *J* = 7.6 Hz, *p*-OBz), 8.03 (d, 2H, *J* = 7.6 Hz, *o*-OBz). ^13^C-NMR (CDCl_3_) *δ* 200.65, 171.17, 170.13, 169.77, 166.84, 166.02, 163.49, 153.21, 142.42, 136.71, 133.90, 132.05, 130.30, 130.20, 130.05, 128.97, 128.68, 128.65, 128.46, 128.11, 115.70, 115.47, 101.12, 94.18, 83.68, 82.03, 80.44, 79.00, 78.89, 74.21, 71.55, 65.15, 60.40, 56.08, 46.91, 43.07, 35.36, 33.17, 28.59, 27.26, 26.22, 21.59, 21.06, 21.02, 14.85, 14.21, 10.73. Anal. calcd. for C_53_H_54_Cl_6_FNO_18_S: C, 50.65; H, 4.33; N, 1.11. Found: C, 50.82; H, 4.37; N, 1.18.

*7,10-Di(2,2,2-trichloroethyloxycarbonyl)-10-deacetylbaccatin III-13-O-[2,2-dimethyl-3-(4-trifluoro methyl) benzene sulfonyl-4-phenyl-oxazolidine-5-carboxylate]* (**13g**). Yield 100% (312 mg); mp 165–167 °C; ^1^H-NMR (CDCl_3_): *δ* 1.19 (s, 3H, 17-CH_3_), 1.27 (s, 3H, 16-CH_3_), 1.84 and 1.92 (2s, 6H, *i*-Pr), 1.99 (s, 3H, 19-CH_3_), 2.02 (s, 3H, 18-CH_3_), 2.03 (s, 3H, OAc), 2.16 (m, 2H, 14-CH_2_), 2.05 and 2.62 (2m, 2H, 6-CH_2_), 3.90 (d, 1H, *J* = 6.8 Hz, 3-CH), 4.13 and 4.29 (2d, 2H, *J* = 8.0 Hz, 20-CH_2_), 4.54 (d, 1H, *J* = 6.4 Hz, 2′-CH), 4.63 and 4.94 (2d, 2H, *J* = 12.0 Hz, Troc), 4.80 (dd, 2H, *J* = 13.6, 12.0 Hz, Troc), 4.93 (d, 1H, *J* = 7.6 Hz, 5-CH), 5.29 (dd, 1H, *J* = 6.0 Hz, 3′-CH), 5.59 (dd, 1H, *J* = 10.8, 6.8 Hz, 7-CH), 5.67 (d, 1H, *J* = 6.8 Hz, 2-CH), 6.24 (t, 1H, *J* = 9.2 Hz, 13-CH), 6.26 (s, 1H, 10-CH), 7.14 (m, 1H, 3′-Ph), 7.23 (m, 4H, 3′-Ph), 7.47 (d, 2H, *J* = 8.4 Hz, -PhCF_3_), 7.53 (d, 2H, *J* = 8.4 Hz, -PhCF_3_), 7.49 (t, 2H, *J* = 8.0 Hz, *m*-OBz), 7.37 (t, 1H, *J* = 7.6 Hz, *p*-OBz), 8.02 (d, 2H, *J* = 7.6 Hz, *o*-OBz). ^13^C-NMR (CDCl_3_) *δ* 200.64, 171.17, 170.10, 169.66, 166.84, 153.21, 144.07, 142.32, 136.19, 134.04, 133.90, 133.71, 132.11, 130.05, 128.96, 128.68, 128.60, 127.94, 125.49, 125.45, 124.47, 121.76, 101.32, 94.18, 83.67, 82.00, 80.46, 78.98, 74.20, 71.62, 65.16, 60.40, 56.08, 46.92, 43.08, 35.36, 33.17, 28.42, 27.55, 26.22, 21.60, 21.06, 21.00, 14.85, 14.20, 10.72. Anal. calcd. for C_54_H_54_Cl_6_F_3_NO_18_S: C, 49.63; H, 4.17; N, 1.07. Found: C, 49.85; H, 4.22; N, 1.13.

*7,10-Di(2,2,2-trichloroethyloxycarbonyl)-10-deacetylbaccatin III-13-O-[2,2-dimethyl-3-(4-chloro) benzene sulfonyl-4-phenyl-oxazolidine-5-carboxylate]* (**13h**). Yield 91% (277 mg); mp 169–171 °C; ^1^H-NMR (CDCl_3_): *δ* 1.19 (s, 3H, 17-CH_3_), 1.27 (s, 3H, 16-CH_3_), 1.84 and 1.89 (2s, 6H, *i*-Pr), 1.96 (s, 3H, 19-CH_3_), 2.01 (s, 3H, 18-CH_3_), 2.03 (s, 3H, OAc), 2.16 (m, 2H, 14-CH_2_), 2.05 and 2.61 (2m, 2H, 6-CH_2_), 3.90 (d, 1H, *J* = 7.2 Hz, 3-CH), 413 and 4.29 (2d, 2H, *J* = 8.4 Hz, 20-CH_2_), 4.53 (d, 1H, *J* = 6.4 Hz, 2′-CH), 4.62 and 4.94 (2d, 2H, *J* = 11.6 Hz, Troc), 4.80 (dd, 2H, *J* = 13.6, 11.6 Hz, Troc), 4.93 (d, 1H, *J* = 8.0 Hz, 5-CH), 5.25 (d, 1H, *J* = 6.4 Hz, 3′-CH), 5.58 (dd, 1H, *J* = 10.8, 7.2 Hz, 2-CH), 5.67 (d, 1H, *J* = 7.2 Hz, 2-CH), 6.24 (t, 1H, *J* = 9.2 Hz, 13-CH), 6.25 (s, 1H, 10-CH), 7.20 (d, 2H, *J* = 9.2 Hz, -PhCl), 7.20 (m, 4H, 3′-Ph), 7.28 (m, 1H, 3′-Ph), 7.36 (d, 2H, *J* = 8.8 Hz, -PhCl), 7.49 (t, 2H, *J* = 7.6 Hz, *m*-OBz), 7.64 (t, 1H, *J* = 7.6 Hz, *p*-OBz), 8.03 (d, 2H, *J* = 7.6 Hz, *o*-OBz). ^13^C-NMR (CDCl_3_) *δ* 200.65, 171.18, 170.13, 169.73, 166.84, 153.21, 142.40, 139.11, 138.95, 136.66, 133.90, 138.95, 136.66, 133.90, 132.06, 130.05, 128.97, 128.92, 128.68, 128.46, 128.14, 101.10, 94.20, 94.18, 83.67, 81.98, 80.44, 78.99, 78.89, 74.21, 71.55, 65.18, 60.41, 56.08, 46.91, 43.07, 35.37, 33.17, 28.58, 27.31, 26.23, 21.60, 21.06, 21.02, 14.84, 14.21, 10.73. Anal. calcd. for C_53_H_54_Cl_7_NO_18_S: C, 50.00; H, 4.27; N, 1.10. Found: C, 50.23; H, 4.34; N, 1.15.

*7,10-Di(2,2,2-trichloroethyloxycarbonyl)-10-deacetylbaccatin III-13-O-[2,2-dimethyl-3-(4-bromo) benzene sulfonyl-4-phenyl-oxazolidine-5-carboxylate]* (**13i**). Yield 98% (309 mg); mp 166–168 °C; ^1^H-NMR (CDCl_3_): *δ* 1.19 (s, 3H, 17-CH_3_), 1.27 (s, 3H, 16-CH_3_), 1.84 and 1.89 (2s, 6H, *i*-Pr), 1.96 (s, 3H, 19-CH_3_), 2.01 (s, 3H, 18-CH_3_), 2.03 (s, 3H, OAc), 2.16 (m, 2H, 14-CH_2_), 2.06 and 2.62 (2m, 2H, 6-CH_2_), 3.90 (d, 1H, *J* = 6.8 Hz, 3-CH), 4.13 and 4.29 (2d, 2H, *J* = 8.4 Hz, 20-CH_2_), 4.53 (d, 1H, *J* = 6.0 Hz, 2′-CH), 4.62 and 4.94 (2d, 2H, *J* = 11.8 Hz, Troc), 4.80 (dd, 2H, *J* = 13.8, 11.8 Hz, Troc), 4.93 (d, 1H, *J* = 8.0 Hz, 5-CH), 5.25 (d, 1H, *J* = 6.0 Hz, 3′-CH), 5.59 (dd, 1H, *J* = 10.8, 7.2 Hz, 7-CH), 5.67 (d, 1H, *J* = 7.2 Hz, 2-CH), 6.24 (t, 1H, *J* = 9.2 Hz, 13-CH), 6.25 (s, 1H, 10-CH), 7.20 (m, 4H, 3′-Ph), 7.28 (m, 1H, 3′-Ph), 7.29 (d, 2H, *J* = 8.8 Hz, -PhBr), 7.37 (d, 2H, *J* = 8.8 Hz, -PhBr), 7.50 (t, 2H, *J* = 7.8 Hz, *m*-OBz), 7.64 (t, 1H, *J* = 7.4 Hz, *p*-OBz), 8.03 (d, 2H, *J* = 7.6 Hz, *o*-OBz). ^13^C-NMR (CDCl_3_) *δ* 200.65, 170.12, 169.73, 166.85, 153.21, 142.39, 139.64, 136.63, 133.90, 132.06, 131.66, 130.06, 129.00, 128.96, 128.71, 128.46, 128.13, 127.49, 101.10, 94.18, 83.67, 81.97, 80.44, 78.99, 78.90, 74.20, 71.56, 65.20, 60.41, 56.08, 46.91, 43.08, 35.36, 33.17, 28.58, 27.32, 26.23, 21.60, 21.01, 14.84, 14.21, 10.73. Anal. calcd. for C_53_H_54_BrCl_6_NO_18_S: C, 48.31; H, 4.13; N, 1.06. Found: C, 48.53; H, 4.23; N, 1.14.

*7,10-Di(2,2,2-trichloroethyloxycarbonyl)-10-deacetylbaccatin III-13-O-[2,2-dimethyl-3-(2,4,6-trimethyl) benzene sulfonyl-4-phenyl-oxazolidine-5-carboxylate]* (**13j**). Yield 81% (248 mg); mp 167–169 °C; ^1^H-NMR (CDCl_3_): *δ* 1.19 (s, 3H, 17-CH_3_), 1.28 (s, 3H, 16-CH_3_), 1.84 and 1.96 (2s, 6H, *i*-Pr), 1.98 (s, 3H, 19-CH_3_), 2.03 (s, 3H, 18-CH_3_), 2.08 (s, 3H, OAc), 2.12 (s, 3H, *p*-3″-CH_3_), 2.14 (m, 2H, 14-CH_2_), 2.05 and 2.62 (2m, 2H, 6-CH_2_), 2.54 (s, 6H, *o*-3″-CH_3_), 3.91 (d, 1H, *J* = 7.2 Hz, 3-CH), 4.13 and 4.28 (2d, 2H, *J* = 8.4 Hz, 20-CH_2_), 4.44 (d, 1H, *J* = 6.8 Hz, 2′-CH), 4.63 and 4.94 (2d, 2H, *J* = 11.8 Hz, Troc), 4.80 (s, 2H, Troc), 4.93 (d, 1H, *J* = 8.0 Hz, 5-CH), 5.15 (d, 1H, *J* = 6.8 Hz, 3′-CH), 5.59 (dd, 1H, *J* = 10.8, 7.2 Hz, 7-CH), 5.67 (d, 1H, *J* = 7.2 Hz, 2-CH), 6.27 (t, 1H, *J* = 8.2 Hz, 13-CH), 6.27 (s, 1H, 10-CH), 6.55 (s, 2H, 3″-Ph), 6.95–7.09 (m, 5H, 3′-Ph), 7.49 (t, 2H, *J* = 7.8 Hz, *m*-OBz), 7.63 (t, 1H, *J* = 7.6 Hz, *p*-OBz), 8.02 (d, 2H, *J* = 7.6 Hz, *o*-OBz). ^13^C-NMR (CDCl_3_) *δ* 200.72, 171.18, 170.17, 170.09, 166.83, 153.21, 153.19, 143.12, 142.62, 140.07, 136.70, 133.85, 132.93, 131.91, 131.69, 130.07, 129.00, 128.65, 128.00, 127.66, 126.91, 102.19, 94.20, 83.70, 82.46, 80.38, 79.02, 78.94, 74.23, 71.45, 65.32, 60.41, 56.06, 46.90, 43.06, 35.38, 33.17, 29.70, 29.05, 26.89, 26.33, 23.04, 21.53, 21.04, 20.72, 14.90, 14.21, 10.73. Anal. calcd. for C_56_H_61_Cl_6_NO_18_S: C, 52.51; H, 4.80; N, 1.09. Found: C, 52.75; H, 4.88; N, 1.17.

#### 3.2.5. General Procedure for the Synthesis of **5a**–**j**

To a round-bottomed flask (25 mL) were added **13a**–**j** (0.218 mmol) and HCOOH (>98%, 5 mL). The reaction mixture was stirred at room temperature for 4 h. Then the resulting solution was neutralized by addition of saturated NaHCO_3_. After extracted with EtOAc three times, the combined organic phase was washed with brine, dried over Na_2_SO_4_, filtered, and concentrated *in vacuo*. The obtained residue was purified by silica gel flash chromatography column (acetone/petroleum ether: 1/3) to afford **5a**–**j** as white solids.

*N-De-tert-butoxycarbonyl-N-phenylsulfonyl 7,10-di(2,2,2-trichloroethyloxycarbonyl)-docetaxel* (**5a**). Yield 72% (188 mg); mp 156–158 °C; ^1^H-NMR (CDCl_3_): *δ* 1.19 (s, 3H, 17-CH_3_), 1.25 (s, 3H, 16-CH_3_), 1.86 (s, 3H, 19-CH_3_), 1.90 (s, 3H, 18-CH_3_), 2.25 (m, 2H, 14-CH_2_), 2.34 (s, 3H, OAc), 2.06 and 2.61 (2m, 2H, 6-CH_2_), 3.86 (d, 1H, *J* = 6.8 Hz, 3-CH), 4.20 and 4.31 (2d, 2H, *J* = 8.4 Hz, 20-CH_2_), 4.54 (d, 1H, *J* = 3.2 Hz, 2′-CH), 4.60 and 4.91 (2d, 2H, *J* = 12.0 Hz, Troc), 4.78 (s, 2H, Troc), 4.93 (d, 1H, *J* = 9.6 Hz, 5-CH), 4.93 (m, 1H, 3′-CH), 5.52 (m, 1H, 7-CH), 5.67 (d, 1H, *J* = 6.8 Hz, 2-CH), 5.79 (m, 1H, -CONH-), 6.16 (t, 1H, *J* = 8.8 Hz, 13-CH), 6.21 (s, 1H, 10-CH), 7.09 (m, 2H, 3′-Ph), 7.18 (m, 3H, 3′-Ph), 7.28 (t, 2H, *J* = 8.0 Hz, *m*-PhSO_2_), 7.42 (t, 1H, *J* = 7.6 Hz, *p*-PhSO_2_), 7.48 (t, 2H, *J* = 7.6 Hz, *m*-OBz), 7.61 (t, 1H, *J* = 7.6 Hz, *p*-OBz), 7.62 (d, 2H, *J* = 7.6 Hz, *o*-PhSO_2_), 8.08 (d, 2H, *J* = 7.6 Hz, *o*-OBz). ^13^C-NMR (CDCl_3_) *δ* 200.62, 170.61, 166.78, 153.22, 142.02, 140.18, 136.31, 133.88, 132.62, 132.30, 130.13, 129.04, 128.81, 128.72, 128.65, 128.36, 127.05, 126.91, 94.17, 83.60, 80.95, 79.12, 78.61, 74.62, 74.12, 72.10, 60.42, 59.50, 56.27, 46.92, 43.02, 35.53, 33.28, 26.50, 22.46, 20.73, 14.91, 14.20, 10.73. Anal. calcd. for C_50_H_51_Cl_6_NO_18_S: C, 50.10; H, 4.29; N, 1.17. Found: C, 50.33; H, 4.43; N, 1.23.

*N-De-tert-butoxycarbonyl-N-phenylmethylsulfonyl 7,10-di(2,2,2-trichloroethyloxycarbonyl)-docetaxel* (**5b**). Yield 79% (208 mg); mp 149–151 °C; ^1^H-NMR (CDCl_3_): *δ* 1.20 (s, 3H, 17-CH_3_), 1.27 (s, 3H, 16-CH_3_), 1.76 (s, 3H, 19-CH_3_), 1.91 (s, 3H, 18-CH_3_), 2.29 (m, 2H, 14-CH_2_), 2.35 (s, 3H, OAc), 2.07 and 2.62 (2m, 2H, 6-CH_2_), 3.89 (d, 1H, *J* = 7.2 Hz, 3-CH), 4.04 (s, 2H, 3″-CH_2_), 4.22 and 4.32 (2d, 2H, *J* = 8.6 Hz, 20-CH_2_), 4.54 (br s, 1H, 2′-CH), 4.61 and 4.92 (2d, 2H, *J* = 11.6 Hz, Troc), 4.79 (s, 2H, Troc), 4.88 (m, 1H, 3′-CH), 4.95 (d, 1H, *J* = 9.2 Hz, 5-CH), 5.53 (dd, 1H, *J* = 10.8, 7.2 Hz, 2-CH), 5.69 (d, 1H, *J* = 7.2 Hz, 2-CH), 6.22 (s, 1H, 10-CH), 6.25 (t, 1H, *J* = 8.0 Hz, 13-CH), 7.07 (d, 2H, *J* = 7.2 Hz, 3″-Ph), 7.22 (t, 2H, *J* = 7.2 Hz, 3′-Ph), 7.35 (d, 2H, *J* = 7.6 Hz, 3″-Ph), 7.42 (m, 3H, 3′-Ph), 7.48 (t, 2H, *J* = 8.0 Hz, *m*-OBz), 7.63 (t, 1H, *J* = 7.4 Hz, *p*-OBz), 8.10 (d, 2H, *J* = 7.6 Hz, *o*-OBz). ^13^C-NMR (CDCl_3_) *δ* 200.65, 171.53, 170.52, 166.75, 153.22, 153.17, 142.17, 137.52, 133.82, 132.20, 130.71, 130.16, 129.08, 129.02, 128.83, 128.68, 128.59, 128.44, 127.48, 94.18, 83.62, 80.89, 79.12, 78.61, 74.68, 74.19, 72.15, 60.41, 60.25, 59.57, 58.47, 56.24, 46.87, 43.04, 35.54, 33.81, 33.26, 29.69, 26.45, 25.57, 24.89, 22.47, 21.05, 20.84, 18.40, 14.79, 14.20, 10.73. Anal. calcd. for C_51_H_53_Cl_6_NO_18_S: C, 50.51; H, 4.40; N, 1.15. Found: C, 50.73; H, 4.53; N, 1.16.

*N-De-tert-butoxycarbonyl-N-(4-methyl)-phenylsulfonyl 7,10-di(2,2,2-trichloroethyloxycarbonyl)-docetaxel* (**5c**). Yield 85% (224 mg); mp 157–159 °C; ^1^H-NMR (CDCl_3_): *δ* 1.20 (s, 3H, 17-CH_3_), 1.26 (s, 3H, 16-CH_3_), 1.88 (s, 3H, 19-CH_3_), 1.91 (s, 3H, 18-CH_3_), 2.26 (m, 2H, 14-CH_2_), 2.34 (s, 3H, OAc), 2.35 (s, 3H, CH_3_ in 4-methylphenyl), 2.08 and 2.63 (2m, 2H, 6-CH_2_), 3.88 (d, 1H, *J* = 6.8 Hz, 3-CH), 4.22 and 4.32 (2d, 2H, *J* = 8.4 Hz, 20-CH_2_), 4.56 (d, 1H, *J* = 3.2 Hz, 2′-CH), 4.62 and 4.93 (2d, 2H, *J* = 12.0 Hz, Troc), 4.80 (s, 2H, Troc), 4.91 (m, 1H, 3′-CH), 4.94 (d, 1H, *J* = 9.2 Hz, 5-CH), 5.54 (m, 1H, 7-CH), 5.69 (d, 1H, *J* = 6.8 Hz, 2-CH), 5.84 (m, 1H, -CONH-), 6.18 (t, 1H, *J* = 9.0 Hz, 13-CH), 6.22 (s, 1H, 10-CH), 7.08 (d, 2H, *J* = 8.4 Hz, *m*-PhSO_2_), 7.13 (m, 2H, 3′-Ph), 7.21 (m, 3H, 3′-Ph), 7.50 (t, 2H, *J* = 7.6 Hz, *m*-OBz), 7.53 (d, 2H, *J* = 8.4 Hz, *o*-PhSO_2_), 7.64 (t, 1H, *J* = 7.4 Hz, *p*-OBz), 8.10 (d, 2H, *J* = 7.6 Hz, *o*-OBz). ^13^C-NMR (CDCl_3_) *δ* 200.64, 170.58, 166.78, 153.22, 153.20, 143.51, 142.10, 137.25, 136.58, 133.87, 132.22, 130.14, 129.40, 129.07, 128.72, 128.62, 128.24, 127.08, 126.96, 94.17, 83.61, 80.90, 79.11, 78.63, 74.60, 74.17, 72.06, 59.51, 56.24, 46.90, 43.02, 35.50, 26.44, 22.46, 21.45, 20.79, 14.85, 10.73. Anal. calcd. for C_51_H_53_Cl_6_NO_18_S: C, 50.51; H, 4.40; N, 1.15. Found: C, 50.75; H, 4.51; N, 1.19.

*N-De-tert-butoxycarbonyl-N-(4-methoxyl)-phenylsulfonyl 7,10-di(2,2,2-trichloroethyloxycarbonyl)-docetaxel* (**5d**). Yield 79% (210 mg); mp 152–154 °C; ^1^H-NMR (CDCl_3_): *δ* 1.21 (s, 3H, 17-CH_3_), 1.27 (s, 3H, 16-CH_3_), 1.86 (s, 3H, 19-CH_3_), 1.92 (s, 3H, 18-CH_3_), 2.22 (m, 2H, 14-CH_2_), 2.36 (s, 3H, OAc), 2.08 and 2.64 (2m, 2H, 6-CH_2_), 3.80 (s, 3H, OCH_3_), 3.89 (d, 1H, *J* = 7.2 Hz, 3-CH), 4.22 and 4.34 (2d, 2H, *J* = 8.4 Hz, 20-CH_2_), 4.55 (d, 1H, *J* = 3.2 Hz, 2′-CH), 4.62 and 4.93 (2d, 2H, *J* = 12.0 Hz, Troc), 4.80 (s, 2H, Troc), 4.91 (m, 1H, 3′-CH), 4.96 (d, 1H, *J* = 9.2 Hz, 5-CH), 5.54 (dd, *J* = 10.8, 7.2 Hz, 7-CH), 5.69 (d, 1H, *J* = 6.8 Hz, 2-CH), 5.74 (m, 1H, -CONH-), 6.16 (t, 1H, *J* = 8.8 Hz, 13-CH), 6.23 (s, 1H, 10-CH), 6.76 (m, 2H, *o*-PhOCH_3_), 7.15 (m, 2H, 3′-Ph), 7.23 (m, 3H, 3′-Ph), 7.51 (t, 2H, *J* = 7.6 Hz, *m*-OBz), 7.58 (d, 2H, *J* = 8.8 Hz, *m*-PhOCH_3_), 7.64 (t, 1H, *J* = 7.2 Hz, *p*-OBz), 8.10 (d, 2H, *J* = 7.6 Hz, *o*-OBz). ^13^C-NMR (CDCl_3_) *δ* 200.64, 171.18, 170.57, 166.77, 162.83, 153.22, 136.72, 133.87, 132.22, 131.87, 130.13, 129.12, 128.72, 128.66, 128.27, 127.09, 113.94, 94.18, 83.60, 80.92, 79.12, 78.62, 74.13, 72.00, 60.40, 56.27, 55.57, 46.91, 43.02, 35.53, 33.80, 33.29, 26.43, 25.56, 24.88, 22.44, 21.05, 20.74, 14.90, 14.20, 10.72. Anal. calcd. for C_51_H_53_Cl_6_NO_19_S: C, 49.85; H, 4.35; N, 1.14. Found: C, 49.99, H, 4.43, N, 1.21.

*N-De-tert-butoxycarbonyl-N-(4-isopropyl)-phenylsulfonyl 7,10-di(2,2,2-trichloroethyloxycarbonyl)-docetaxel* (**5e**). Yield 77% (208 mg); mp 165–167 °C; ^1^H-NMR (CDCl_3_): *δ* 1.20 (br s, 6H, 2CH_3_ in *i*-Pr), 1.21 (s, 3H, 17-CH_3_), 1.27 (s, 3H, 16-CH_3_), 1.88 (s, 3H, 19-CH_3_), 1.92 (s, 3H, 18-CH_3_), 2.29 (m, 2H, 14-CH_2_), 2.37 (s, 3H, OAc), 2.08 and 2.64 (2m, 2H, 6-CH_2_), 2.88 (m, 1H, CH in *i*-Pr), 3.89 (d, 1H, *J* = 6.4 Hz, 3-CH), 4.23 and 4.32 (2d, 2H, *J* = 8.4 Hz, 20-CH_2_), 4.56 (d, 1H, *J* = 3.6 Hz, 2′-CH), 4.62 and 4.93 (2d, 2H, *J* = 11.6 Hz, Troc), 4.80 (s, 2H, Troc), 4.93 (m, 1H, 3′-CH), 4.94 (d, 1H, *J* = 9.2 Hz, 5-CH), 5.54 (dd, 1H, *J* = 10.6, 7.4 Hz, 7-CH), 5.70 (d, 1H, *J* = 6.8 Hz, 2-CH), 6.23 (s, 1H, 10-CH), 6.23 (t, 1H, *J* = 9.0 Hz, 13-CH), 7.01-7.15 (m, 8H, 3′-Ph and *i*-PrPh), 7.52 (m, 2H, *i*-PrPh), 7.52 (t, 2H, *J* = 7.6 Hz, *m*-OBz), 7.63 (t, 1H, *J* = 7.2 Hz, *p*-OBz), 8.11 (d, 2H, *J* = 7.6 Hz, *o*-OBz). ^13^C-NMR (100 MHz, CDCl_3_) *δ* 200.65, 171.23, 170.63, 166.74, 154.17, 153.22, 142.15, 137.36, 136.21, 133.82, 132.22, 130.15, 129.13, 128.71, 128.51, 128.21, 127.09, 126.84, 94.18, 83.62, 80.88, 79.12, 78.62, 74.66, 74.21, 72.06, 60.41, 59.63, 56.22, 46.91, 43.02, 35.62, 34.08, 33.27, 26.45, 23.65, 23.60, 22.47, 21.05, 20.87, 14.83, 14.20, 10.75. Anal. calcd. for C_53_H_57_Cl_6_NO_18_S: C, 51.30; H, 4.63; N, 1.13. Found: C, 51.53; H, 4.72; N, 1.18.

*N-De-tert-butoxycarbonyl-N-(4-fluoro)-phenylsulfonyl 7,10-di(2,2,2-trichloroethyloxycarbonyl)-docetaxel* (**5f**). Yield 50% (132 mg); mp 162–164 °C;^1^H-NMR (CDCl_3_): *δ* 1.21 (s, 3H, 17-CH_3_), 1.26 (s, 3H, 16-CH_3_), 1.88 (s, 3H, 19-CH_3_), 1.92 (s, 3H, 18-CH_3_), 2.27 (m, 2H, 14-CH_2_), 2.36 (s, 3H, OAc), 2.08 and 2.63 (2m, 2H, 6-CH_2_), 3.88 (d, 1H, *J* = 6.8 Hz, 3-CH), 4.22 and 4.33 (2d, 2H, *J* = 8.6 Hz, 20-CH_2_), 4.56 (d, 1H, *J* = 3.6 Hz, 2′-CH), 4.62 and 4.93 (2d, 2H, *J* = 11.6 Hz, Troc), 4.80 (s, 2H, Troc), 4.95 (d, 1H, *J* = 9.6 Hz, 3′-CH), 4.96 (d, 1H, *J* = 9.2 Hz, 5-CH), 5.53 (dd, 1H, *J* = 10.6, 7.4 Hz, 5-CH), 5.69 (d, 1H, *J* = 7.2 Hz, 2-CH), 5.92 (d, 1H, *J* = 9.2 Hz, -CONH-), 6.20 (t, 1H, *J* = 8.0 Hz, 13-CH), 6.23 (s, 1H, 10-CH), 6.94 (t, 2H, *J* = 8.4 Hz, -PhF), 7.11 (m, 2H, 3′-Ph), 7.21 (m, 3H, 3′-Ph), 7.50 (t, 2H, *J* = 7.6 Hz, *m*-OBz), 7.62 (m, 2H, -PhF), 7.63 (t, 1H, *J* = 7.6 Hz, *p*-OBz), 8.10 (d, 2H, *J* = 8.0 Hz, *o*-OBz). ^13^C-NMR (CDCl_3_) *δ* 200.58, 171.21, 171.08, 170.66, 166.77, 166.16, 163.63, 153.22, 141.91, 136.30, 136.12, 133.90, 132.40, 130.12, 129.71, 129.62, 129.02, 128.71, 128.69, 128.47, 127.13, 126.89, 116.05, 115.82, 94.16, 83.58, 81.01, 79.10, 78.58, 74.62, 74.10, 72.11, 60.42, 59.57, 58.48, 56.29, 46.94, 43.02, 35.51, 33.28, 26.47, 22.45, 21.05, 20.67, 18.41, 14.94, 14.20, 10.72. Anal. calcd. for C_50_H_50_Cl_6_FNO_18_S: C, 49.36; H, 4.14; N, 1.15. Found: C, 49.58; H, 4.22; N, 1.16.

*N-De-tert-butoxycarbonyl-N-(4-trifluoromethyl)-phenylsulfonyl 7,10-di(2,2,2-trichloroethyloxy carbonyl)-docetaxel* (**5g**). Yield 56% (154 mg); mp 159–161 °C; ^1^H-NMR (CDCl_3_): *δ* 1.21 (s, 3H, 17-CH_3_), 1.27 (s, 3H, 16-CH_3_), 1.89 (s, 3H, 19-CH_3_), 1.90 (s, 3H, 18-CH_3_), 2.29 (m, 2H, 14-CH_2_), 2.36 (s, 3H, OAc), 2.09 and 2.64 (2m, 2H, 6-CH_2_), 3.88 (d, 1H, *J* = 6.8 Hz, 3-CH), 4.23 and 4.33 (2d, 2H, *J* = 8.6 Hz, 20-CH_2_), 4.58 (d, 1H, *J* = 2.4 Hz, 2′-CH), 4.62 and 4.93 (2d, 2H, *J* = 11.6 Hz, Troc), 4.80 (s, 2H, Troc), 4.96 (d, 1H, *J* = 9.6 Hz, 5-CH), 5.01 (dd, 1H, *J* = 9.2, 7.2 Hz, 3′-CH), 5.53 (dd, 1H, *J* = 10.6, 7.4 Hz, 7-CH), 5.69 (d, 1H, *J* = 7.2 Hz, 2-CH), 6.00 (d, 1H, *J* = 9.6 Hz, -CONH-), 6.22 (t, 1H, *J* = 8.2 Hz, 13-CH), 6.23 (s, 1H, 10-CH), 7.07 (m, 2H, 3′-Ph), 7.17 (m, 3H, 3′-Ph), 7.50 (d, 2H, *J* = 8.4 Hz, -PhCF_3_), 7.50 (t, 2H, *J* = 7.6 Hz, *m*-OBz), 7.64 (t, 1H, *J* = 7.6 Hz, *p*-OBz), 7.70 (d, 2H, *J* = 8.4 Hz, -PhCF_3_), 8.09 (d, 2H, *J* = 7.2 Hz, *o*-OBz). ^13^C-NMR (CDCl_3_) *δ* 200.54, 170.86, 170.76, 166.78, 153.24, 143.76, 141.74, 135.70, 133.93, 132.53, 130.11, 128.98, 128.72, 128.68, 128.54, 127.41, 127.17, 125.79, 94.15, 83.55, 81.10, 79.10, 78.54, 74.57, 74.03, 72.12, 59.60, 58.49, 56.33, 46.97, 43.02, 35.50, 33.30, 26.52, 22.46, 21.05, 20.56, 18.42, 15.00, 14.20, 10.71. Anal. calcd. for C_51_H_50_Cl_6_F_3_NO_18_S: C, 48.36; H, 3.98; N, 1.11. Found: C, 48.59; H, 4.11; N, 1.15.

*N-De-tert-butoxycarbonyl-N-(4-chloro)-phenylsulfonyl 7,10-di(2,2,2-trichloroethyloxycarbonyl)-docetaxel* (**5h**). Yield 67% (180 mg); mp 162–164 °C; ^1^H-NMR (CDCl_3_): *δ* 1.21 (s, 3H, 17-CH_3_), 1.27 (s, 3H, 16-CH_3_), 1.88 (s, 3H, 19-CH_3_), 1.90 (s, 3H, 18-CH_3_), 2.27 (m, 2H, 14-CH_2_), 2.35 (s, 3H, OAc), 2.08 and 2.64 (2m, 2H, 6-CH_2_), 3.88 (d, 1H, *J* = 6.8 Hz, 3-CH), 4.22 and 4.33 (2d, 2H, *J* = 8.8 Hz, 20-CH_2_), 4.57 (br s, 1H, 2′-CH), 4.62 and 4.93 (2d, 2H, *J* = 12.0 Hz, Troc), 4.80 (s, 2H, Troc), 4.96 (d, 1H, *J* = 9.6 Hz, 5-CH), 4.97 (d, 1H, *J* = 9.2 Hz, 3′-CH), 5.53 (dd, 1H, *J* = 10.4, 7.0 Hz, 2-CH), 5.69 (d, 1H, *J* = 7.2 Hz, 2-CH), 5.92 (d, 1H, *J* = 9.2 Hz, -CONH-), 6.19 (t, 1H, *J* = 8.8 Hz, 13-CH), 6.23 (s, 1H, 10-CH), 7.12 (m, 2H, -PhCl), 7.23 (m, 5H, 3′-Ph), 7.50 (t, 2H, *J* = 7.6 Hz, *m*-OBz), 7.53 (d, 2H, *J* = 8.8 Hz, -PhCl), 7.64 (t, 1H, *J* = 7.6 Hz, *p*-OBz), 8.09 (d, 2H, *J* = 7.6 Hz, *o*-OBz). ^13^C-NMR (CDCl_3_) *δ* 200.57, 171.01, 170.68, 166.77, 153.22, 141.85, 139.09, 138.78, 136.12, 133.92, 132.43, 130.12, 129.00, 128.72, 128.48, 128.36, 127.14, 94.17, 83.57, 81.03, 79.10, 78.57, 74.58, 74.07, 72.12, 60.42, 59.55, 56.30, 46.95, 43.02, 35.51, 33.29, 26.49, 22.46, 20.64, 14.95, 14.20, 10.72. Anal. calcd. for C_50_H_50_Cl_7_NO_18_S: C, 48.70; H, 4.09; N, 1.14. Found: C, 48.95; H, 4.11; N, 1.18.

*N-De-tert-butoxycarbonyl-N-(4-bromo)-phenylsulfonyl 7,10-di(2,2,2-trichloroethyloxycarbonyl)-docetaxel* (**5i**). Yield 57% (158 mg); mp 155–157 °C; ^1^H-NMR (CDCl_3_): *δ* 1.21 (s, 3H, 17-CH_3_), 1.27 (s, 3H, 16-CH_3_), 1.88 (s, 3H, 19-CH_3_), 1.91 (s, 3H, 18-CH_3_), 2.27 (m, 2H, 14-CH_2_), 2.35 (s, 3H, OAc), 2.08 and 2.63 (2m, 2H, 6-CH_2_), 3.88 (d, 1H, *J* = 7.2 Hz, 3-CH), 4.22 and 4.33 (2d, 2H, *J* = 8.6 Hz, 20-CH_2_), 4.60 (t, 1H, *J* = 3.6 Hz, 2′-CH), 4.62 and 4.93 (2d, 2H, *J* = 11.6 Hz, Troc), 4.80 (s, 2H, Troc), 4.96 (d, 1H, *J* = 9.6 Hz, 5-CH), 4.97 (d, 1H, *J* = 9.2 Hz, 3′-CH), 5.53 (dd, 1H, *J* = 10.8, 7.2 Hz, 7-CH), 5.69 (d, 1H, *J* = 7.2 Hz, 2-CH), 5.92 (d, 1H, *J* = 9.2 Hz, -CONH-), 6.20 (t, 1H, *J* = 8.8 Hz, 13-CH), 6.23 (s, 1H, 10-CH), 7.11 (m, 2H, 3′-Ph), 7.24 (m, 3H, 3′-Ph), 7.40 (m, 2H, -PhBr), 7.46 (m, 2H, -PhBr), 7.50 (t, 2H, *J* = 7.8 Hz, *m*-OBz), 7.64 (t, 1H, *J* = 7.6 Hz, *p*-OBz), 8.09 (d, 2H, *J* = 7.6 Hz, *o*-OBz). ^13^C-NMR (CDCl_3_) *δ* 200.57, 171.21, 171.02, 170.67, 166.77, 153.22, 141.85, 139.33, 136.12, 133.91, 132.43, 131.98, 130.12, 129.01, 128.72, 128.43, 127.55, 127.15, 126.96, 94.16, 83.57, 81.03, 79.10, 78.57, 74.57, 74.08, 72.11, 60.42, 59.57, 58.48, 56.30, 53.43, 46.95, 43.03, 35.52, 33.29, 26.51, 22.46, 21.05, 20.64, 18.42, 14.95, 14.20, 10.72. Anal. calcd. for C_50_H_50_BrCl_6_NO_18_S: C, 47.00; H, 3.94; N, 1.10. Found: C, 47.30; H, 4.03; N, 1.17.

*N-De-tert-butoxycarbonyl-N-(2,4,6-trimethyl)-phenylsulfonyl 7,10-di(2,2,2-trichloroethyloxy carbonyl)-docetaxel* (**5j**). Yield 68% (183 mg); mp 151–153 °C; ^1^H-NMR (CDCl_3_): *δ* 1.21 (s, 3H, 17-CH_3_), 1.26 (s, 3H, 16-CH_3_), 1.87 (s, 3H, 19-CH_3_), 1.92 (s, 3H, 18-CH_3_), 2.29 (m, 2H, 14-CH_2_), 2.23 (s, 3H, *p*-3″-CH_3_), 2.32 (s, 3H, OAc), 2.08 and 2.63 (2m, 2H, 6-CH_2_), 2.50 (s, 6H, *o*-3″-CH_3_), 3.88 (d, 1H, *J* = 6.8 Hz, 3-CH), 4.21 and 4.33 (2d, 2H, *J* = 8.6 Hz, 20-CH_2_), 4.54 (d, 1H, *J* = 3.6 Hz, 2′-CH), 4.62 and 4.93 (2d, 2H, *J* = 11.6 Hz, Troc), 4.75 (m, 1H, 3′-CH), 4.80 (s, 2H, Troc), 4.96 (d, 1H, *J* = 9.6 Hz, 5-CH), 5.53 (dd, 1H, *J* = 10.4, 7.2 Hz, 7-CH), 5.66 (d, 1H, *J* = 8.8 Hz, -CONH-), 5.70 (d, 1H, *J* = 6.8 Hz, 2-CH), 6.12 (t, 1H, *J* = 8.8 Hz, 13-CH), 6.23 (s, 1H, 10-CH), 6.78 (s, 2H, 3″-Ph), 7.10-7.22 (m, 5H, 3′-Ph), 7.53 (t, 2H, *J* = 7.8 Hz, *m*-OBz), 7.67 (t, 1H, *J* = 7.2 Hz, *p*-OBz), 8.10 (d, 2H, *J* = 7.2 Hz, *o*-OBz). ^13^C-NMR (CDCl_3_) *δ* 200.63, 171.51, 170.31, 166.77, 153.22, 153.18, 142.38, 142.09, 138.60, 136.74, 134.20, 133.90, 132.26, 131.83, 130.08, 129.05, 128.71, 128.54, 128.35, 126.94, 94.17, 83.61, 80.90, 79.11, 78.59, 74.34, 74.10, 72.24, 59.39, 58.48, 56.24, 46.86, 43.05, 35.26, 33.27, 31.92, 29.68, 26.43, 22.87, 22.48, 20.85, 18.42, 14.73, 10.71. Anal. calcd. for C_53_H_57_Cl_6_NO_18_S: C, 51.30; H, 4.63; N, 1.13. Found: C, 51.53; H, 4.65; N, 1.17.

#### 3.2.6. General Procedure for the Synthesis of **3a**–**j**

To a solution of **5a**–**j** (0.19 mmol) in methanol (10 mL) were added glacial acetic acid (4.60 mL) and zinc powder (0.46 g, 7.08 mmol). After stirred at 50 °C for 1 h, the reaction mixture was filtered to remove the zinc and solid formed. The filtrate was evaporated by distillation to give a white solid. The obtained solid was then dissolved in ethyl acetate (60 mL), which was washed with saturated NaHCO_3_, brine, dried over Na_2_SO_4_, and concentrated *in vacuo*. The obtained residue was further purified by silica gel flash chromatography column (petroleum ether/acetone: 2/1) to give **3a**–**j**.

*N-De-tert-butoxycarbonyl-N-phenylsulfonyl docetaxel* (**3a**). White powder; Yield 75% (121 mg); mp 174–175 °C; ^1^H-NMR (CDCl_3_): *δ* 1.09 (s, 3H, 17-CH_3_), 1.19 (s, 3H, 16-CH_3_), 1.73 (s, 3H, 19-CH_3_), 1.81 (s, 3H, 18-CH_3_), 2.15 (m, 2H, 14-CH_2_), 2.30 (s, 3H, OAc), 1.82 and 2.51 (2m, 2H, 6-CH_2_), 3.84 (d, 1H, *J* = 7.2 Hz, 3-CH), 4.20 and 4.26 (2d, 2H, *J* = 8.4 Hz, 20-CH_2_), 4.23 (m, 1H, 7-CH), 4.50 (d, 1H, *J* = 4.0 Hz, 2′-CH), 4.89 (d, 1H, *J* = 9.6 Hz, 5-CH), 4.89 (m, 1H, 3′-CH), 5.22 (s, 1H, 10-CH), 5.63 (d, 1H, *J* = 7.2 Hz, 2-CH), 6.14 (t, 1H, *J* = 8.8 Hz, 13-CH), 6.35 (d, 1H, *J* = 6.0 Hz, -CONH-), 7.09 (m, 2H, 3′-Ph), 7.14 (m, 3H, 3′-Ph), 7.24 (t, 2H, *J* = 8.0 Hz, *m*-PhSO_2_), 7.40 (t, 1H, *J* = 7.6 Hz, *p*-PhSO_2_), 7.46 (t, 2H, *J* = 7.6 Hz, *m*-OBz), 7.58 (t, 1H, *J* = 7.6 Hz, *p*-OBz), 7.59 (d, 2H, *J* = 7.6 Hz, *o*-PhSO_2_), 8.07 (d, 2H, *J* = 7.6 Hz, *o*-OBz); ^13^C-NMR (CD_3_COCD_3_) *δ* 210.54, 171.85, 170.02, 165.73, 141.70, 137.67, 137.40, 136.71, 133.15, 131.98, 130.50, 130.00, 128.56, 128.48, 128.03, 127.57, 127.54, 126.77, 84.16, 80.86, 77.87, 77.76, 75.97, 75.23, 74.92, 74.28, 71.52, 71.26, 60.51, 57.63, 54.08, 46.52, 46.52, 43.25, 36.68, 36.10, 26.27, 22.17, 20.62, 13.55, 9.52; HRMS (ESI) *m/z* calcd. for C_44_H_49_NO_14_SNa^+^ [M+Na^+^]: 870.2771, found 870.2802.

*N-De-tert-butoxycarbonyl-N-phenylmethylsulfonyl docetaxel* (**3b**). White powder; Yield 74% (121 mg); mp 168–170 °C; ^1^H-NMR (CDCOCD_3_): *δ* 1.14 (s, 3H, 17-CH_3_), 1.21 (s, 3H, 16-CH_3_), 1.74 (s, 3H, 19-CH_3_), 1.89 (s, 3H, 18-CH_3_), 2.17 (m, 2H, 14-CH_2_), 2.41 (s, 3H, OAc), 1.85 and 2.46 (2m, 2H, 6-CH_2_), 3.89 (s, 1H, 2′-OH), 3.92 (d, 1H, *J* = 7.2 Hz, 3-CH), 4.09 and 4.22 (2d, 2H, *J* = 14.0 Hz, CH_2_Ph), 4.16 and 4.19 (2d, 2H, *J* = 8.4 Hz, 20-CH_2_), 4.32 (m, 1H, 7-CH), 4.36 (br s, 1H, 10-OH), 4.64 (t, 1H, *J* = 5.2 Hz, 2′-CH), 4.97 (d, 1H, *J* = 9.2 Hz, 5-CH), 5.00 (d, 1H, *J* = 5.6 Hz, 3′-CH), 5.12 (m, 1H, -CONH-), 5.24 (d, 1H, *J* = 2.0 Hz,10-CH), 5.67 (d, 1H, *J* = 7.6 Hz, 2-CH), 6.22 (t, 1H, *J* = 8.8 Hz, 13-CH), 7.25-7.36 (m, 6H, 3′-Ph and 3″-Ph), 7.47 (t, 2H, *J* = 7.2 Hz, *m*-OBz), 7.55 (m, 4H, 3″-Ph), 7.66 (t, 1H, *J* = 7.2 Hz, *p*-OBz), 8.10 (d, 2H, *J* = 7.2 Hz, *o*-OBz); ^13^C-NMR (CD_3_COCD_3_) *δ* 210.54, 172.23, 170.07, 165.76, 138.92, 137.38, 136.73, 133.15, 130.94, 130.45, 130.05, 129.98, 128.55, 128.51, 128.19, 128.14, 128.00, 127.93, 84.18, 80.83, 77.83, 75.98, 75.20, 75.00, 74.28, 71.52, 71.16, 60.70, 59.68, 57.63, 54.09, 46.48, 43.24, 36.67, 35.98, 26.22, 22.29, 20.61, 18.00, 13.52, 9.52; HRMS (ESI) *m/z* calcd. for C_45_H_51_NO_14_SNa^+^ [M+Na^+^]: 884.2928, found 884.2944.

*N-De-tert-butoxycarbonyl-N-(4-methyl)-phenylsulfonyl docetaxel* (**3c**). White powder; Yield 80% (131 mg); mp 172–174 °C; ^1^H-NMR (CDCl_3_): *δ* 1.10 (s, 3H, 17-CH_3_), 1.19 (s, 3H, 16-CH_3_), 1.73 (s, 3H, 19-CH_3_), 1.78 (s, 3H, 18-CH_3_), 2.14 (m, 2H, 14-CH_2_), 2.30 (s, 3H, OAc), 2.30 (s, 3H, CH_3_ in 4-methylphenyl), 1.83 and 2.52 (2m, 2H, 6-CH_2_), 3.84 (d, 1H, *J* = 6.8 Hz, 3-CH), 4.20 and 4.27 (2d, 2H, *J* = 8.4 Hz, 20-CH_2_), 4.21 (m, 1H, 7-CH), 4.50 (d, 1H, *J* = 3.6 Hz, 2′-CH), 4.89 (d, 1H, *J* = 9.2 Hz, 5-CH), 4.89 (m, 1H, 3′-CH), 5.20 (s, 1H, 10-CH), 5.63 (d, 1H, *J* = 7.6 Hz, 2-CH), 6.12 (m, 1H,-CONH-), 6.13 (t, 1H, *J* = 9.2 Hz, 13-CH), 7.04 (d, 2H, *J* = 8.0 Hz, *m*-PhSO_2_), 7.11 (m, 2H, 3′-Ph), 7.16 (m, 3H, 3′-Ph), 7.47 (t, 2H, *J* = 8.0 Hz, *m*-OBz), 7.49 (d, 2H, *J* = 8.4 Hz, *o*-PhSO_2_), 7.59 (t, 1H, *J* = 7.6 Hz, *p*-OBz), 8.07 (d, 2H, *J* = 7.6 Hz, *o*-OBz); ^13^C-NMR (CD_3_COCD_3_) *δ* 210.55, 171.86, 170.05, 165.75, 142.66, 138.85, 137.93, 137.44, 136.66, 133.17, 130.48, 130.00, 129.08, 128.92, 128.50, 128.20, 128.03, 127.79, 127.59, 127.43, 126.92, 126.84, 84.17, 80.86, 77.88, 77.77, 75.98, 75.23, 74.92, 74.29, 71.53, 71.26, 60.44, 57.64, 46.52, 43.24, 36.67, 36.09, 26.25, 22.16, 20.61, 20.47, 17.99, 13.55, 9.53; HRMS (ESI) *m/z* calcd. for C_45_H_51_NO_14_SNa^+^ [M+Na^+^]: 884.2928, found 884.2942.

*N-De-tert-butoxycarbonyl-N-(4-methoxyl)-phenylsulfonyl docetaxel* (**3d**). White powder; Yield 73% (122 mg); mp 172–174 °C; ^1^H-NMR (CDCOCD_3_): *δ* 1.16 (s, 3H, 17-CH_3_), 1.23 (s, 3H, 16-CH_3_), 1.74 (s, 3H, 19-CH_3_), 1.89 (s, 3H, 18-CH_3_), 2.25 (m, 2H, 14-CH_2_), 2.40 (s, 3H, OAc), 1.86 and 2.45 (2m, 2H, 6-CH_2_), 3.82 (s, 3H, OCH_3_), 3.93 (d, 1H, *J* = 6.8 Hz, 3-CH), 3.93 (s, 1H, 2′-OH), 4.16 and 4.22 (2d, 2H, *J* = 8.2 Hz, 20-CH_2_), 4.31 (m, 1H, 7-CH), 4.37 (br s, 1H, 10-OH), 4.56 (d, 1H, *J* = 4.0 Hz, 2′-CH), 4.91 (m, 1H, 3′-CH), 4.87 (m, 1H, -CONH-), 4.97 (d, 1H, *J* = 9.2 Hz, 5-CH), 5.24 (s, 1H, 10-CH), 5.69 (d, 1H, *J* = 6.8 Hz, 2-CH), 6.17 (t, 1H, *J* = 9.0 Hz, 13-CH), 6.85 (d, 2H, *J* = 8.8 Hz, *o*-PhOCH_3_), 7.16 (t, 1H, *J* = 7.2 Hz, 3′-Ph), 7.22 (t, 2H, *J* = 7.2 Hz, 3′-Ph), 7.28 (d, 1H, *J* = 7.2 Hz, 3′-Ph), 7.57 (t, 2H, *J* = 7.2 Hz, *m*-OBz), 7.60 (d, 2H, *J* = 8.8 Hz, *m*-PhOCH_3_), 7.67 (t, 1H, *J* = 7.2 Hz, *p*-OBz), 8.13 (d, 2H, *J* = 7.6 Hz, *o*-OBz); ^13^C-NMR (CD_3_COCD_3_) *δ* 210.55, 171.88, 170.04, 165.75, 162.51, 137.88, 137.42, 136.69, 133.36, 133.16, 130.49, 130.01, 128.92, 128.50, 128.04, 127.61, 127.47, 113.69, 84.17, 80.85, 77.88, 75.97, 75.23, 74.95, 74.28, 71.53, 71.26, 60.44, 57.63, 55.12, 46.52, 43.24, 36.68, 36.11, 26.24, 22.17, 20.62, 13.57, 9.52; HRMS (ESI) *m/z* calcd. for C_45_H_51_NO_15_SNa^+^ [M+Na^+^]: 900.2877, found 900.2889.

*N-De-tert-butoxycarbonyl-N-(4-isopropyl)-phenylsulfonyl docetaxel* (**3e**). White powder; Yield 77% (130 mg); mp 171–173 °C; ^1^H-NMR (CDCOCD_3_): *δ* 1.17 (s, 3H, 17-CH_3_), 1.20 (s, 3H, 16-CH_3_), 1.22 (s, 3H, CH_3_ in *i*-Pr), 1.25 (s, 3H, CH_3_ in *i*-Pr), 1.75 (s, 3H, 19-CH_3_), 1.91 (s, 3H, 18-CH_3_), 2.29 (m, 2H, 14-CH_2_), 2.43 (s, 3H, OAc), 1.86 and 2.47 (2m, 2H, 6-CH_2_), 2.90 (m, 1H, CH in *i*-Pr), 3.95 (d, 1H, *J* = 7.2 Hz, 3-CH), 3.96 (s, 1H, 2′-OH), 4.17 and 4.24 (2d, 2H, *J* = 8.2 Hz, 20-CH_2_), 4.32 (m, 1H, 7-CH), 4.38 (br s, 1H, 10-OH), 4.57 (d, 1H, *J* = 4.8 Hz, 2′-CH), 4.93 (d, 1H, *J* = 4.4 Hz, 3′-CH), 4.98 (d, 1H, *J* = 9.2 Hz, 5-CH), 5.25 (s, 1H, 10-CH), 5.69 (d, 1H, *J* = 7.6 Hz, 2-CH), 6.25 (t, 1H, *J* = 9.0 Hz, 13-CH), 7.17 (m, 8H, 3′-Ph and *i*-PrPh), 7.55 (d, 2H, *J* = 8.0 Hz, *i*-PrPh), 7.56 (t, 2H, *J* = 7.6 Hz, *m*-OBz), 7.66 (t, 1H, *J* = 7.2 Hz, *p*-OBz), 8.14 (d, 2H, *J* = 7.2 Hz, *o*-OBz); ^13^C-NMR (CD_3_COCD_3_) *δ* 210.56, 171.93, 170.08, 165.79, 153.27, 138.97, 137.49, 137.40, 136.69, 133.15, 130.50, 130.03, 128.49, 128.21, 127.96, 127.76, 127.64, 127.45, 127.06, 127.00, 126.51, 126.39, 84.19, 80.87, 77.91, 77.80, 75.99, 75.27, 74.91, 74.29, 71.52, 71.30, 60.48, 57.64, 46.51, 43.27, 36.67, 36.13, 33.84, 26.30, 23.13, 23.05, 22.21, 20.68, 17.99, 15.37, 13.58, 9.56; HRMS (ESI) *m/z* calcd. for C_47_H_55_NO_14_SNa^+^ [M+Na^+^]: 912.3241, found 912.3239.

*N-De-tert-butoxycarbonyl-N-(4-fluoro)-phenylsulfonyl docetaxel* (**3f**). White powder; Yield 53% (87 mg); mp 173–175 °C; ^1^H-NMR (CDCOCD_3_): *δ* 1.16 (s, 3H, 17-CH_3_), 1.23 (s, 3H, 16-CH_3_), 1.74 (s, 3H, 19-CH_3_), 1.90 (s, 3H, 18-CH_3_), 2.24 (m, 2H, 14-CH_2_), 2.41 (s, 3H, OAc), 1.85 and 2.46 (2m, 2H, 6-CH_2_), 3.93 (d, 1H, *J* = 7.2 Hz, 3-CH), 3.93 (s, 1H, 2′-OH), 4.16 and 4.22 (2d, 2H, *J* = 8.0 Hz, 20-CH_2_), 4.31 (m, 1H, 7-CH), 4.37 (br s, 1H, 10-OH), 4.57 (d, 1H, *J* = 4.8 Hz, 2′-CH), 4.95 (d, 1H, *J* = 4.8 Hz, 3′-CH), 4.97 (d, 1H, *J* = 9.0 Hz, 5-CH), 5.24 (s, 1H, 10-CH), 5.69 (d, 1H, *J* = 7.6 Hz, 2-CH), 6.19 (t, 1H, *J* = 8.8 Hz, 13-CH), 7.09 (t, 2H, *J* = 7.2 Hz, 3′-Ph), 7.18 (d, 1H, *J* = 7.2 Hz, 3′-Ph), 7.21 (d, 2H, *J* = 6.8 Hz, -PhF), 7.27 (d, 2H, *J* = 6.8 Hz, 3′-Ph), 7.57 (t, 2H, *J* = 8.0 Hz, *m*-OBz), 7.67 (t, 1H, *J* = 7.6 Hz, *p*-OBz), 7.71 (dd, 2H, *J* = 8.8, 5.2 Hz, -PhF), 8.12 (d, 2H, *J* = 8.0 Hz, *o*-OBz); ^13^C-NMR (CD_3_COCD_3_) *δ* 210.53, 171.84, 170.05, 165.74, 165.71, 163.21, 138.03, 138.00, 137.45, 137.37, 136.74, 133.17, 130.48, 130.00, 129.79, 129.70, 128.49, 128.07, 127.68, 127.60, 115.59, 115.36, 84.17, 80.86, 77.87, 75.97, 75.23, 74.90, 74.27, 71.52, 71.23, 60.64, 57.63, 46.51, 43.25, 36.67, 36.12, 26.23, 22.18, 20.63, 13.56, 9.53; HRMS (ESI) *m/z* calcd. for C_44_H_48_FNO_14_SNa^+^ [M+Na^+^]: 888.2677, found 888.2700.

*N-De-tert-butoxycarbonyl-N-(4-trifluoromethyl)-phenylsulfonyl docetaxel* (**3g**). White powder; Yield 57% (99 mg); mp 168–170 °C; ^1^H-NMR (CDCOCD_3_): *δ* 1.16 (s, 3H, 17-CH_3_), 1.23 (s, 3H, 16-CH_3_), 1.74 (s, 3H, 19-CH_3_), 1.91 (s, 3H, 18-CH_3_), 2.24 (m, 2H, 14-CH_2_), 2.42 (s, 3H, OAc), 1.86 and 2.47 (2m, 2H, 6-CH_2_), 3.93 (s, 1H, 2′-OH), 3.93 (d, 1H, *J* = 6.0 Hz, 3-CH), 4.16 and 4.23 (2d, 2H, *J* = 8.0 Hz, 20-CH_2_), 4.32 (m, 1H, 7-CH), 4.37 (br s, 1H, 10-OH), 4.59 (d, 1H, *J* = 4.8 Hz, 2′-CH), 4.97 (d, 1H, *J* = 8.8 Hz, 5-CH), 4.98 (d, 1H, *J* = 4.4 Hz, 3′-CH), 5.24 (s, 1H, 10-CH), 5.69 (d, 1H, *J* = 6.8 Hz, 2-CH), 6.23 (t, 1H, *J* = 9.0 Hz, 13-CH), 7.16 (m, 3H, 3′-Ph), 7.24 (d, 1H, *J* = 6.8 Hz, 3′-Ph), 7.56 (t, 2H, *J* = 7.6 Hz, *m*-OBz), 7.66 (t, 1H, *J* = 7.6 Hz, *p*-OBz), 7.66 (d, 2H, *J* = 8.0 Hz, -PhCF_3_), 7.84 (d, 2H, *J* = 8.0 Hz, -PhCF_3_), 8.12 (d, 2H, *J* = 8.0 Hz, *o*-OBz); ^13^C-NMR (CD_3_COCD_3_) *δ* 210.51, 171.79, 170.06, 165.75, 145.38, 137.35, 137.03, 136.77, 133.16, 132.87, 132.55, 130.48, 130.00, 128.48, 128.06, 127.75, 127.66, 125.67, 125.63, 125.00, 122.30, 84.17, 80.87, 77.87, 75.97, 75.24, 74.82, 74.27, 71.52, 71.23, 60.81, 57.63, 46.51, 43.25, 36.66, 36.13, 26.24, 22.20, 20.65, 13.55, 9.53; HRMS (ESI) *m/z* calcd. for C_45_H_48_F_3_NO_14_SNa^+^ [M+Na^+^]: 938.2645, found 938.2651.

*N-De-tert-butoxycarbonyl-N-(4-chloro)-phenylsulfonyl docetaxel* (**3h**). White powder; Yield 65% (109 mg); mp 178–180 °C; ^1^H-NMR (CDCOCD_3_): *δ* 1.16 (s, 3H, 17-CH_3_), 1.23 (s, 3H, 16-CH_3_), 1.74 (s, 3H, 19-CH_3_), 1.90 (s, 3H, 18-CH_3_), 2.24 (m, 2H, 14-CH_2_), 2.41 (s, 3H, OAc), 1.86 and 2.47 (2m, 2H, 6-CH_2_), 3.93 (d, 1H, *J* = 6.0 Hz, 3-CH), 3.93 (s, 1H, 2′-OH), 4.16 and 4.22 (2d, 2H, *J* = 8.0 Hz, 20-CH_2_), 4.32 (m, 1H, 7-CH), 4.38 (br s, 1H, 10-OH), 4.58 (d, 1H, *J* = 4.8 Hz, 2′-CH), 4.95 (d, 1H, *J* = 4.8 Hz, 3′-CH), 4.97 (d, 1H, *J* = 10.2 Hz, 5-CH), 5.25 (s, 1H, 10-CH), 5.69 (d, 1H, *J* = 7.2 Hz, 2-CH), 6.20 (t, 1H, *J* = 9.0 Hz, 13-CH), 7.21 (m, 3H, 3′-Ph), 7.28 (d, 1H, *J* = 6.8 Hz, 3′-Ph), 7.36 (d, 2H, *J* = 8.4 Hz, -PhCl), 7.57 (t, 2H, *J* = 7.6 Hz, *m*-OBz), 7.65 (d, 2H, *J* = 8.8 Hz, -PhCl), 7.67 (t, 1H, *J* = 7.2 Hz, *p*-OBz), 8.12 (d, 2H, *J* = 7.2 Hz, *o*-OBz); ^13^C-NMR (CD_3_COCD_3_) *δ* 210.53, 171.83, 170.07, 165.76, 140.49, 137.60, 137.44, 137.39, 136.74, 133.19, 130.47, 130.00, 128.67, 128.62, 128.50, 128.09, 127.70, 127.60, 84.18, 80.86, 77.88, 75.98, 75.24, 74.86, 74.28, 71.53, 71.25, 60.67, 57.64, 46.51, 43.26, 36.66, 36.12, 26.27, 22.20, 20.65, 13.57, 9.55; HRMS (ESI) *m/z* calcd. for C_44_H_48_ClNO_14_SNa^+^ [M+Na^+^]: 904.2382, found 904.2359.

*N-De-tert-butoxycarbonyl-N-(4-bromo)-phenylsulfonyl docetaxel* (**3i**). White powder; Yield 61% (107 mg); mp 179–181 °C; ^1^H-NMR (CDCOCD_3_): *δ* 1.16 (s, 3H, 17-CH_3_), 1.24 (s, 3H, 16-CH_3_), 1.74 (s, 3H, 19-CH_3_), 1.90 (s, 3H, 18-CH_3_), 2.24 (m, 2H, 14-CH_2_), 2.41 (s, 3H, OAc), 1.86 and 2.46 (2m, 2H, 6-CH_2_), 3.93 (s, 1H, 2′-OH), 3.93 (d, 1H, *J* = 6.0 Hz, 3-CH), 4.16 and 4.22 (2d, 2H, *J* = 8.0 Hz, 20-CH_2_), 4.32 (m, 1H, 7-CH), 4.37 (br s, 1H, 10-OH), 4.58 (t, 1H, *J* = 5.0 Hz, 2′-CH), 4.95 (d, 1H, *J* = 5.2 Hz, 3′-CH), 4.95 (m, 1H, -CONH-), 4.97 (d, 1H, *J* = 7.6 Hz, 5-CH), 5.24 (d, 1H, *J* = 2.0 Hz,10-CH), 5.69 (d, 1H, *J* = 7.2 Hz, 2-CH), 6.20 (t, 1H, *J* = 9.0 Hz, 13-CH), 7.22 (m, 3H, 3′-Ph), 7.28 (d, 1H, *J* = 6.8 Hz, 3′-Ph), 7.53 (t, 2H, *J* = 7.6 Hz, *m*-OBz), 7.58 (d, 4H, *J* = 8.4 Hz, -PhBr), 7.67 (t, 1H, *J* = 7.2 Hz, *p*-OBz), 8.12 (d, 2H, *J* = 7.2 Hz, *o*-OBz); ^13^C-NMR (CD_3_COCD_3_) *δ* 210.52, 171.81, 170.04, 165.74, 140.96, 137.43, 137.34, 136.76, 133.17, 131.68, 130.48, 130.00, 128.71, 128.49, 128.10, 127.71, 127.58, 126.11, 84.16, 80.86, 77.87, 75.97, 75.23, 74.86, 74.27, 71.52, 71.24, 60.67, 57.64, 46.51, 43.26, 36.68, 36.13, 26.28, 22.19, 20.63, 13.55, 9.53; HRMS (ESI) *m/z* calcd. for C_44_H_48_BrNO_14_SNa^+^ [M+Na^+^]: 948.1877, found 948.1870.

*N-De-tert-butoxycarbonyl-N-(2,4,6-trimethyl)-phenylsulfonyl docetaxel* (**3j**). White powder; Yield 65% (110 mg); mp 165–167 °C; ^1^H-NMR (CDCOCD_3_): *δ* 1.13 (s, 3H, 17-CH_3_), 1.21 (s, 3H, 16-CH_3_), 1.73 (s, 3H, 19-CH_3_), 1.87 (s, 3H, 18-CH_3_), 2.16 (m, 2H, 14-CH_2_), 2.20 (s, 3H, *p*-3″-CH_3_), 2.36 (s, 3H, OAc), 1.84 and 2.45 (2m, 2H, 6-CH_2_), 2.55 (s, 6H, *o*-3″-CH_3_), 3.83 (s, 1H, 2′-OH), 3.90 (d, 1H, *J* = 7.2 Hz, 3-CH), 4.16 and 4.19 (2d, 2H, *J* = 8.0 Hz, 20-CH_2_), 4.30 (m, 1H, 7-CH), 4.35 (br s, 1H, 10-OH), 4.53 (t, 1H, *J* = 4.0 Hz, 2′-CH), 4.75 (d, 2H, *J* = 4.8 Hz, -CONH-), 4.96 (d, 1H, *J* = 9.6 Hz, 5-CH), 5.00 (m, 1H, 3′-CH), 5.23 (d, 1H, *J* = 2.0 Hz,10-CH), 5.68 (d, 1H, *J* = 7.2 Hz, 2-CH), 6.09 (t, 1H, *J* = 8.4 Hz, 13-CH), 6.85 (s, 2H, 3″-Ph), 7.17-7.28 (m, 5H, 3′-Ph), 7.61 (t, 2H, *J* = 8.0 Hz, *m*-OBz), 7.71 (t, 1H, *J* = 7.4 Hz, *p*-OBz), 8.11 (d, 2H, *J* = 7.6 Hz, *o*-OBz); ^13^C-NMR (CD_3_COCD_3_) *δ* 210.52, 171.94, 169.86, 165.72, 141.79, 138.47, 137.86, 137.33, 136.77, 135.17, 133.22, 131.57, 130.46, 129.91, 128.52, 127.97, 127.61, 127.36, 84.13, 80.86, 77.77, 77.67, 75.93, 75.13, 74.77, 74.29, 71.52, 71.32, 60.19, 57.63, 46.50, 43.24, 36.67, 35.82, 26.21, 22.36, 22.21, 20.49, 19.91, 13.46, 9.47. HRMS (ESI) *m/z* calcd. for C_47_H_55_NO_14_SNa^+^ [M+Na^+^]: 912.3241, found 912.3264.

### 3.3. Biological Assays: Anti-HBV Tests

Drug stock solutions were prepared in DMSO and stored at −70 °C. Upon dilution into culture medium, the final DMSO concentration was <1% DMSO (v/v), a concentration without effect on cell replication. Cell culture and other procedures were the same as those reported previously [15]. A HepG2-derived human hepatablastoma cell line, 2.2.15, was used in this study, which was transfected with cloned HBV DNA to produce HBV particles. All stock cultures were grown in T-25 flasks containing the DMEM supplemented with 10% (v/v) fetal bovine serum, 0.03% (v/v) L-glutamine, 100 mg/mL penicillin, 100 mg/mL streptomycin, and 380 mg/mL G418 at 37 °C in a humidified atmosphere containing 5% CO_2_. After the HepG2 2.2.15 cell suspensions seeded in 24-well microtiter plates were cultured for 48 h, they were incubated at 37 °C for 9 d in the presence of various concentrations of drugs (200, 100, 50, 25, 12.5 and 6.25 mg/mL respectively) from DMSO-diluted stock, and the medium was refreshed every 3 d. Then the culture supernatants were harvested to detect the HBsAg and HBeAg secretion using diagnostic ELISA kits (Shanghai SIIC KEHUA Biotech Co., Ltd., Shanghai, China) as described in triplicate, and the standard error of the mean (SEM) of inhibition values varied no more than 5%. Cell damage was assessed using the MTT assay.

## 4. Conclusions

In conclusion, we have provided a convenient route for the synthesis in high yields of 3′-*N*-phenylsulfonyl docetaxel analogs from the key intermediate *N*-phenylsulfonyl oxazolidine. Among them, compounds **3e**, **3g** and **3j** showed more potent inhibitory activity against HBeAg secretion than the positive control lamivudine.

## Supplementary Materials

Supplementary materials can be accessed at: http://www.mdpi.com/1420-3049/18/9/10189/s1.
