# Supplementary material for: Synthesis and Anti-HBV Activity of Novel 3′-N-phenylsulfonyl Docetaxel Analogs
Source: Molecules. 2013 Aug 22;18(9):10189–212. doi: 10.3390/molecules180910189 (PMC6270178; doi:10.3390/molecules180910189)
Supplement: Supplementary file 1 [file molecules-18-10189-s001.pdf]

Supplementary Materials

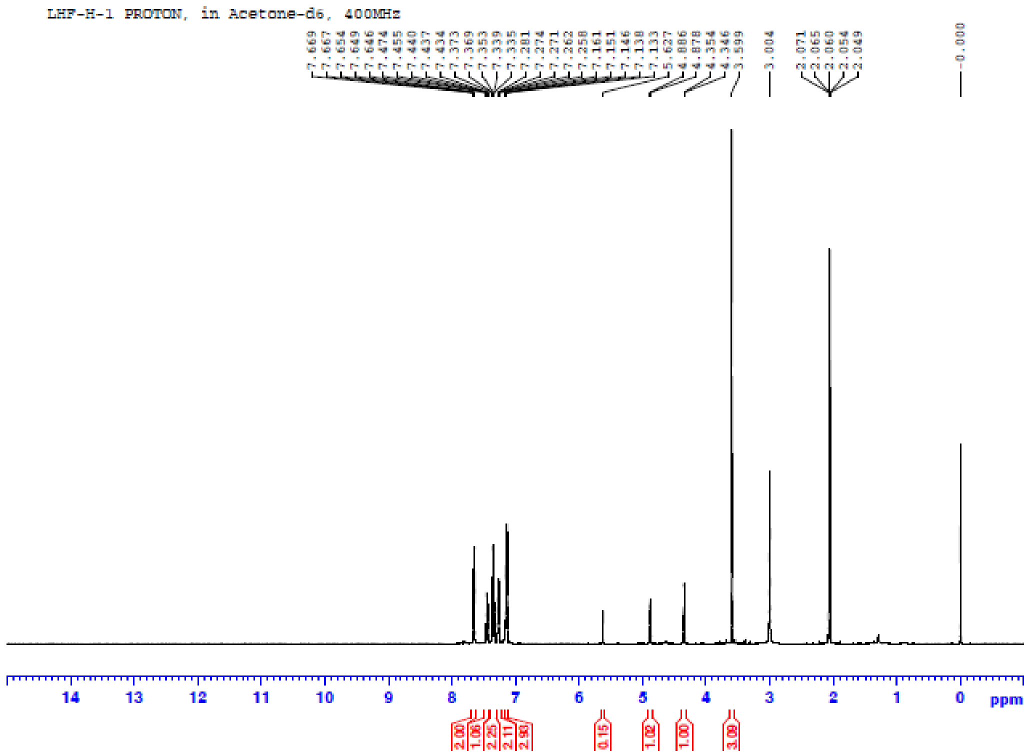

^{1}H-NMR of 7a

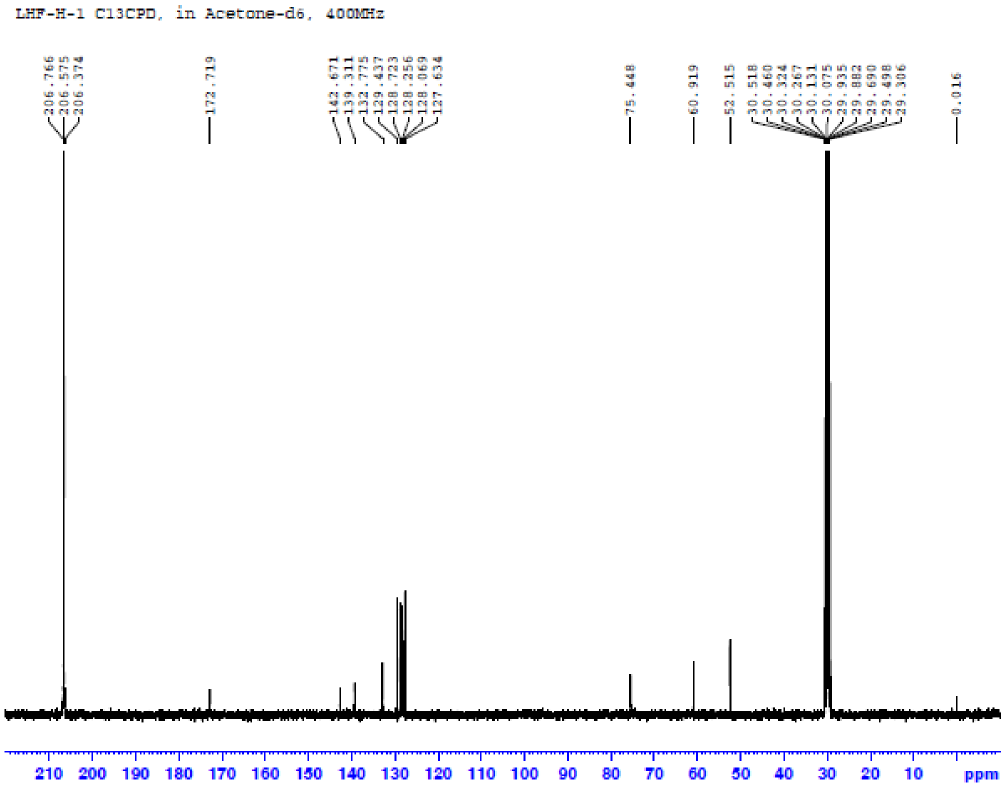

^{13}C-NMR of 7a

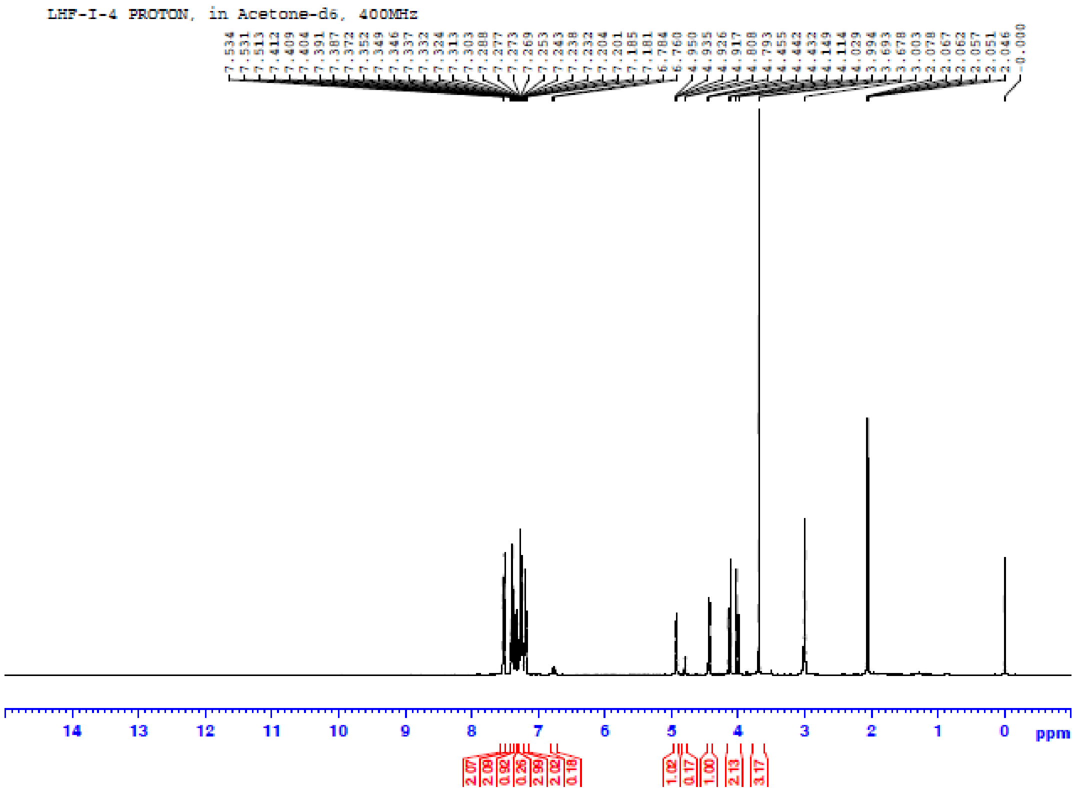

^{1}H-NMR of 7b

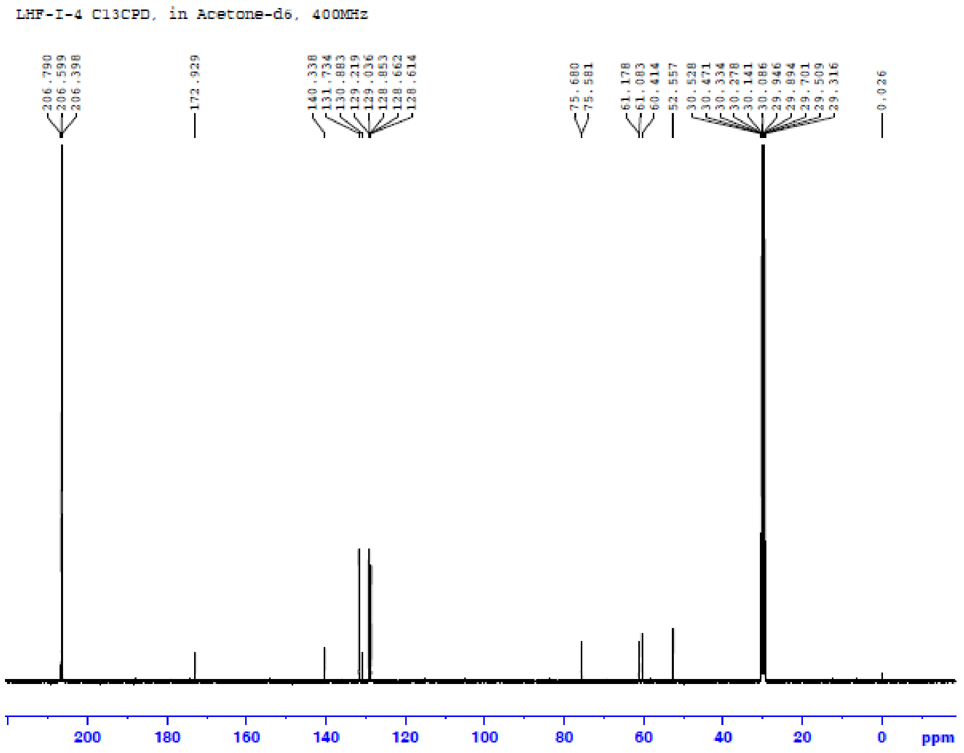

^{13}C-NMR of 7b

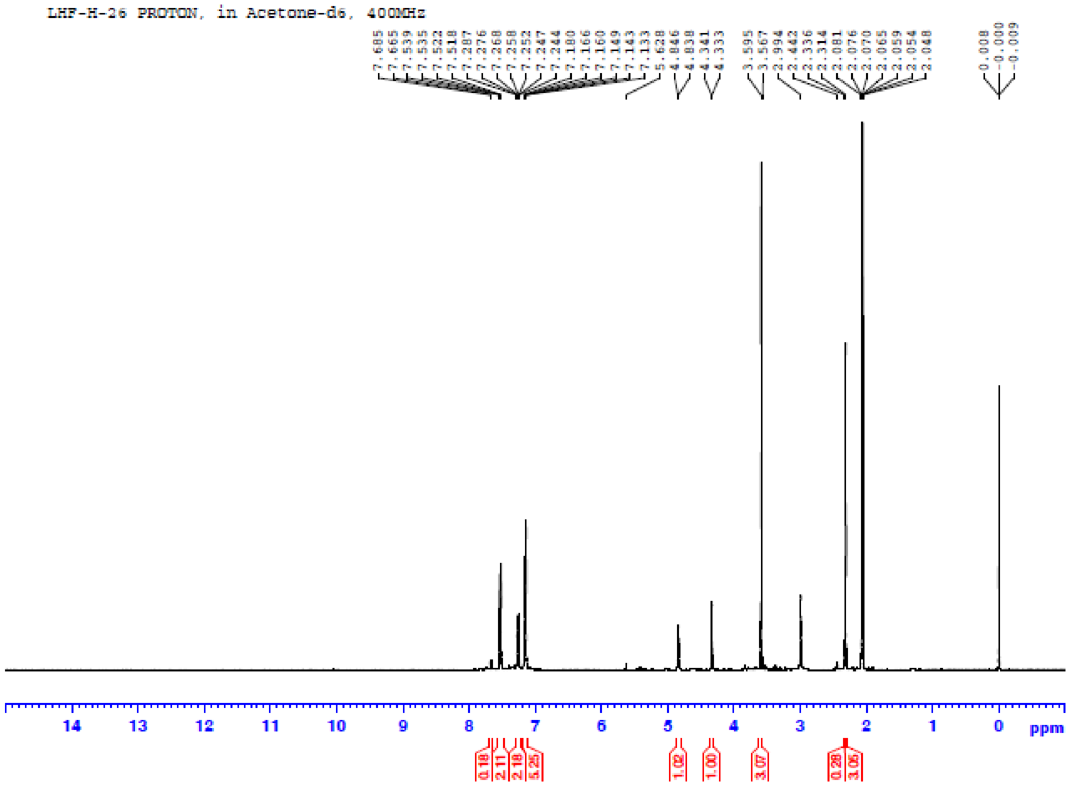

^{1}H-NMR of 7c

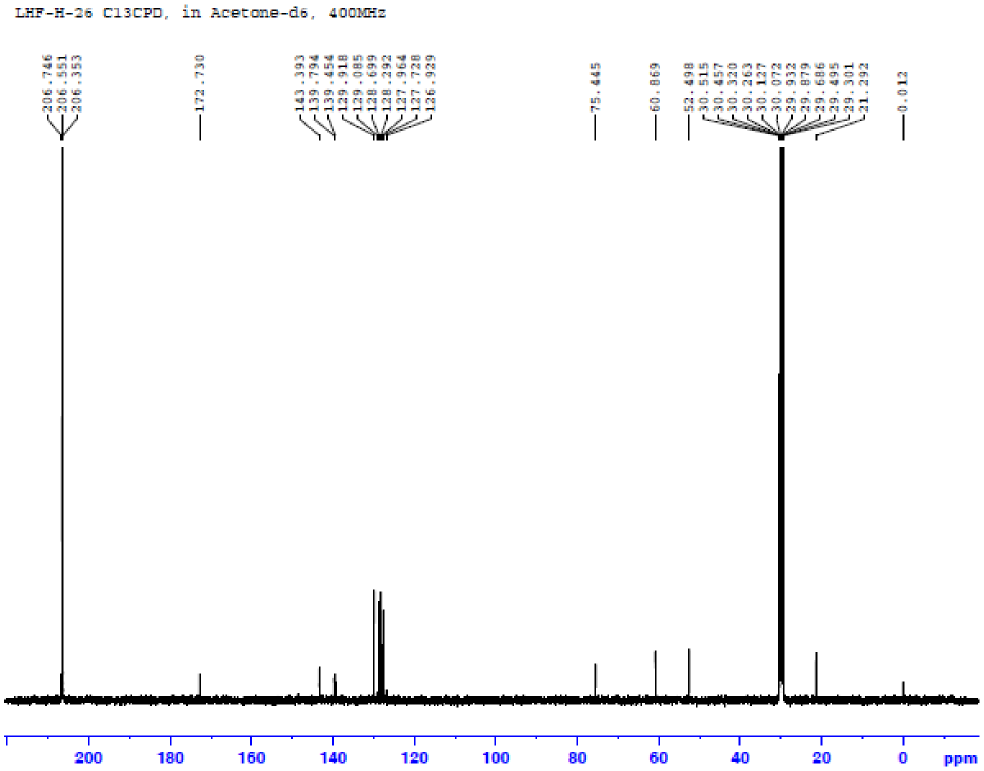

^{13}C-NMR of 7c

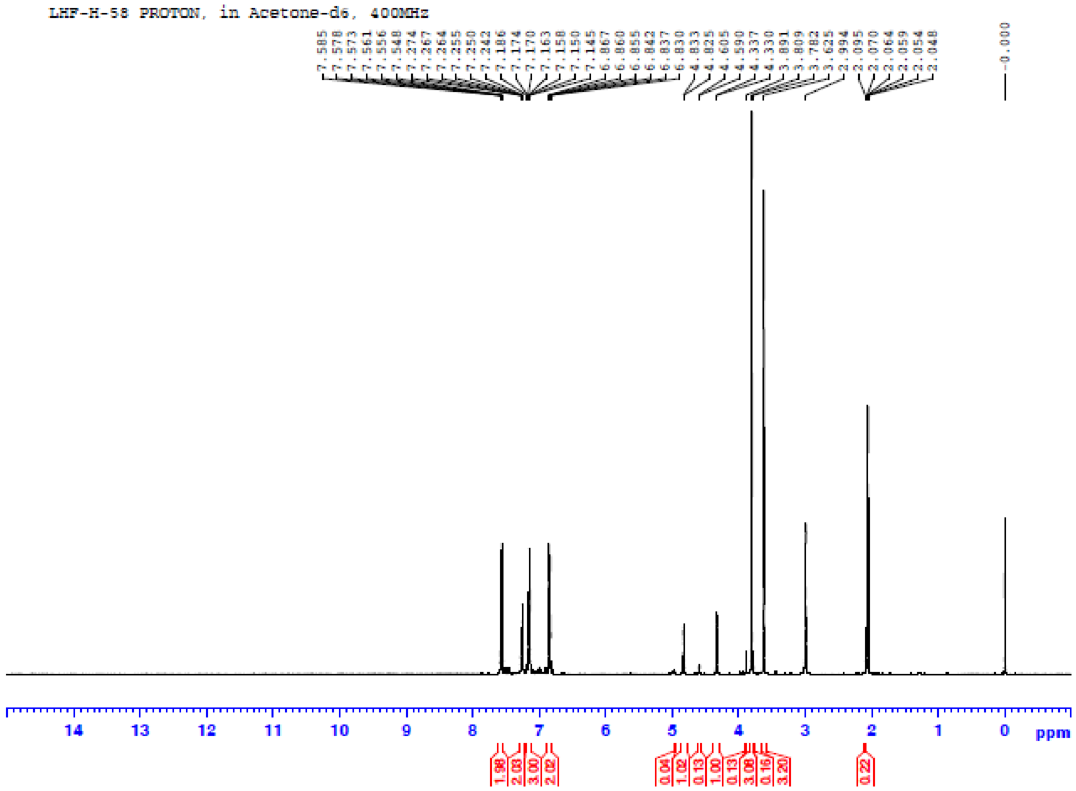

^{1}H-NMR of 7d

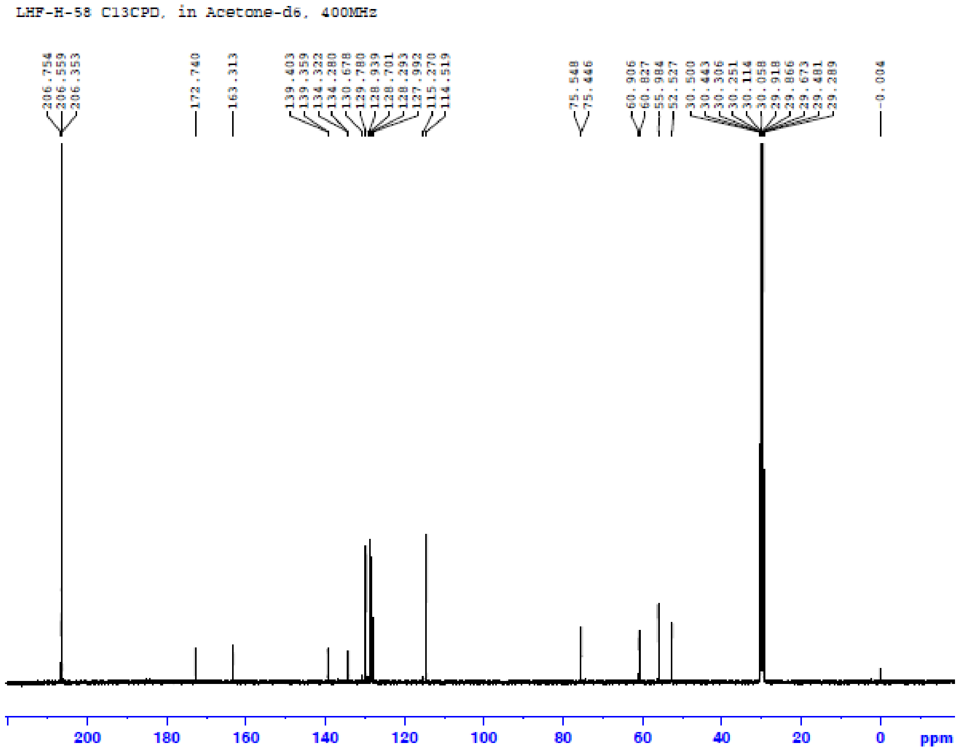

^{13}C-NMR of 7d

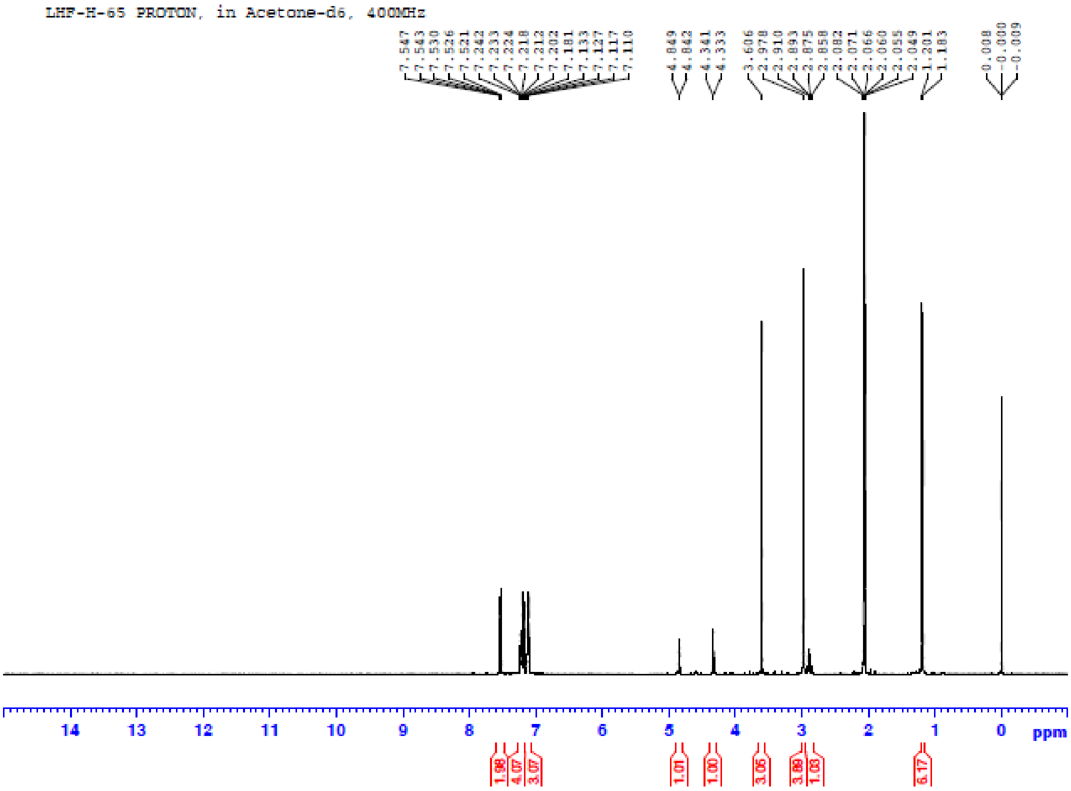

^{1}H-NMR of 7e

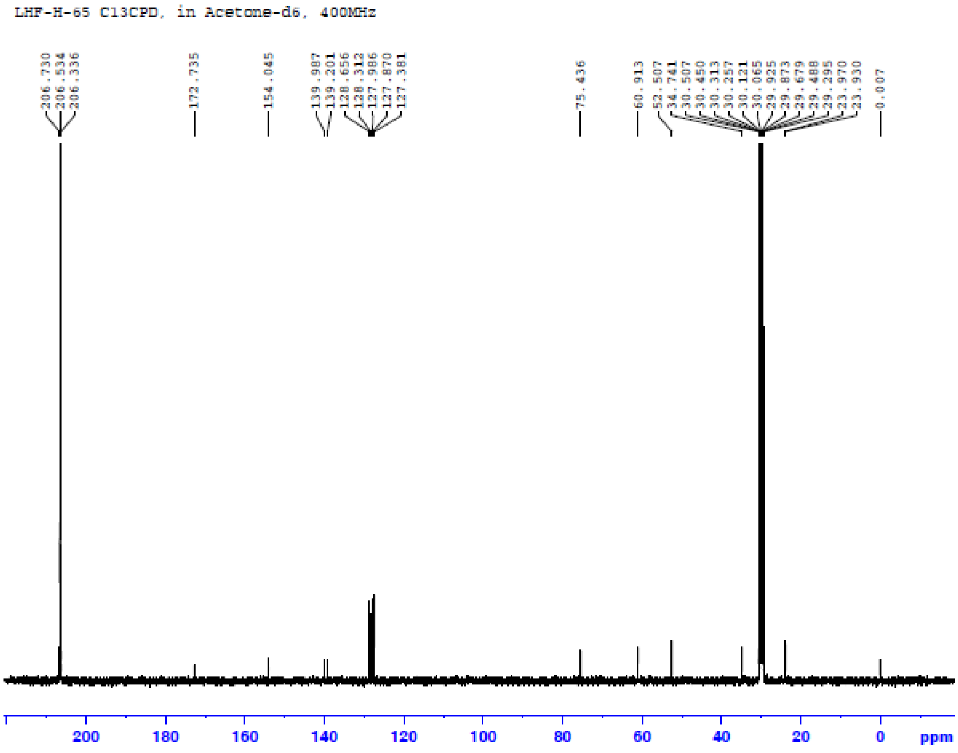

^{13}C-NMR of 7e

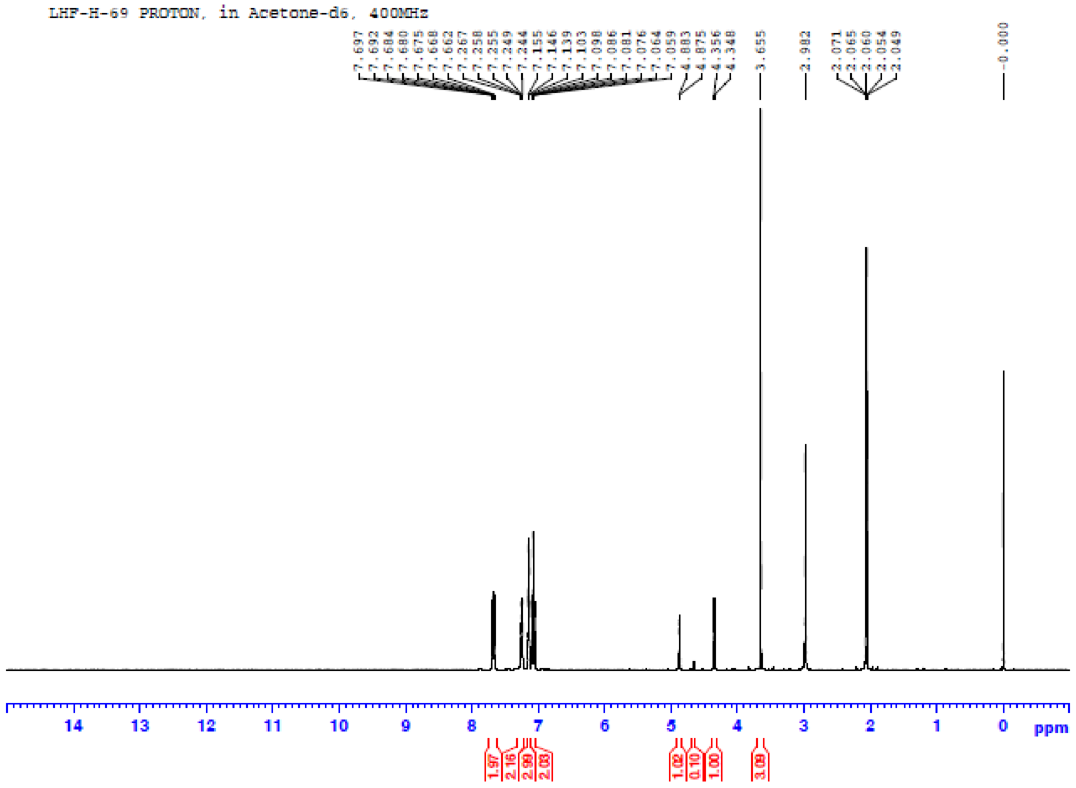

^{1}H-NMR of 7f

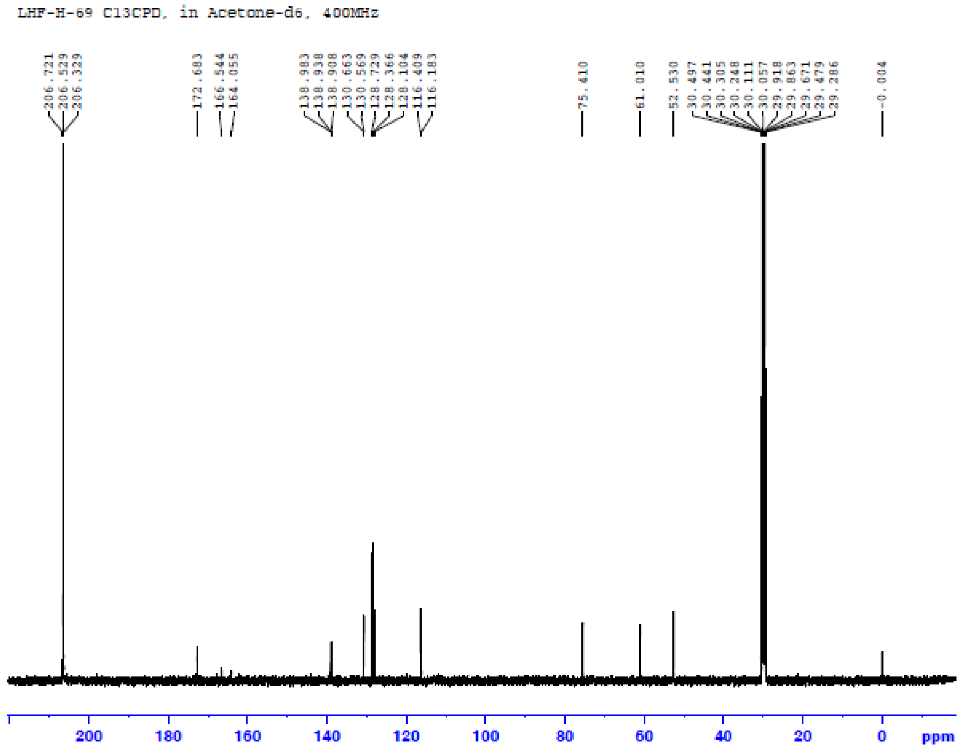

^{13}C-NMR of 7f

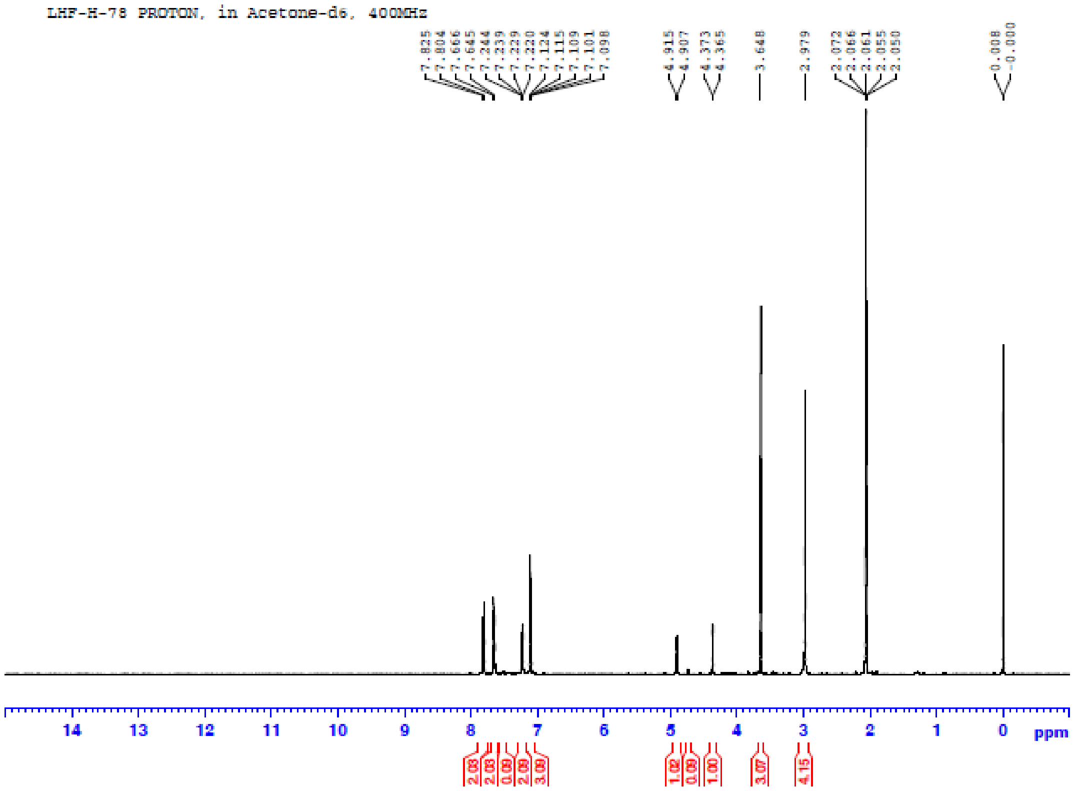

^{1}H-NMR of 7g

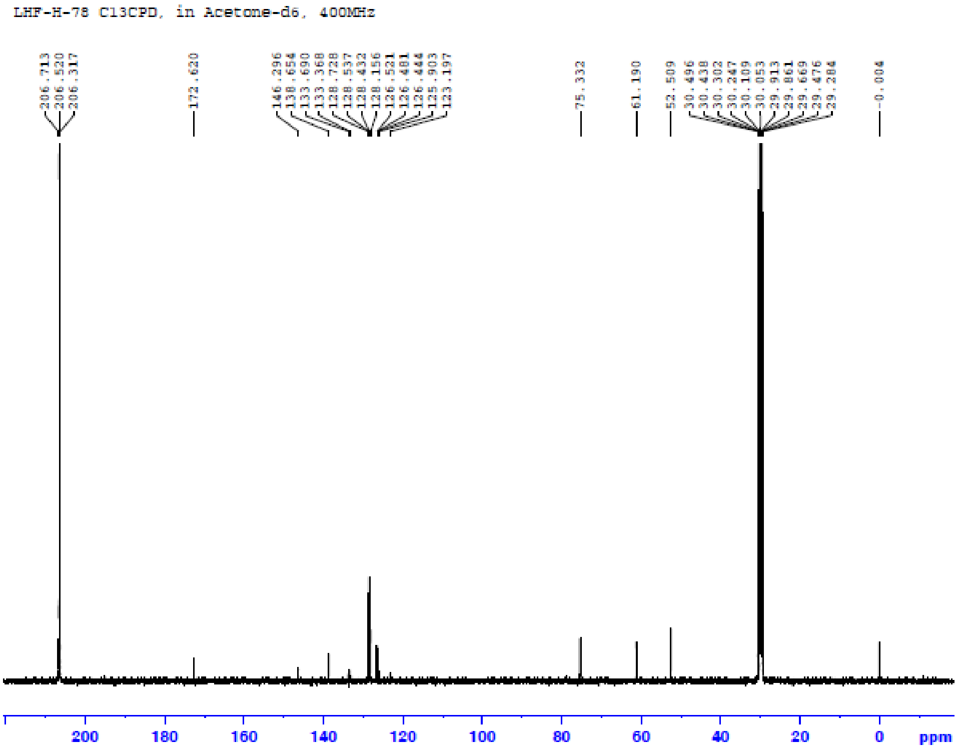

^{13}C-NMR of 7g

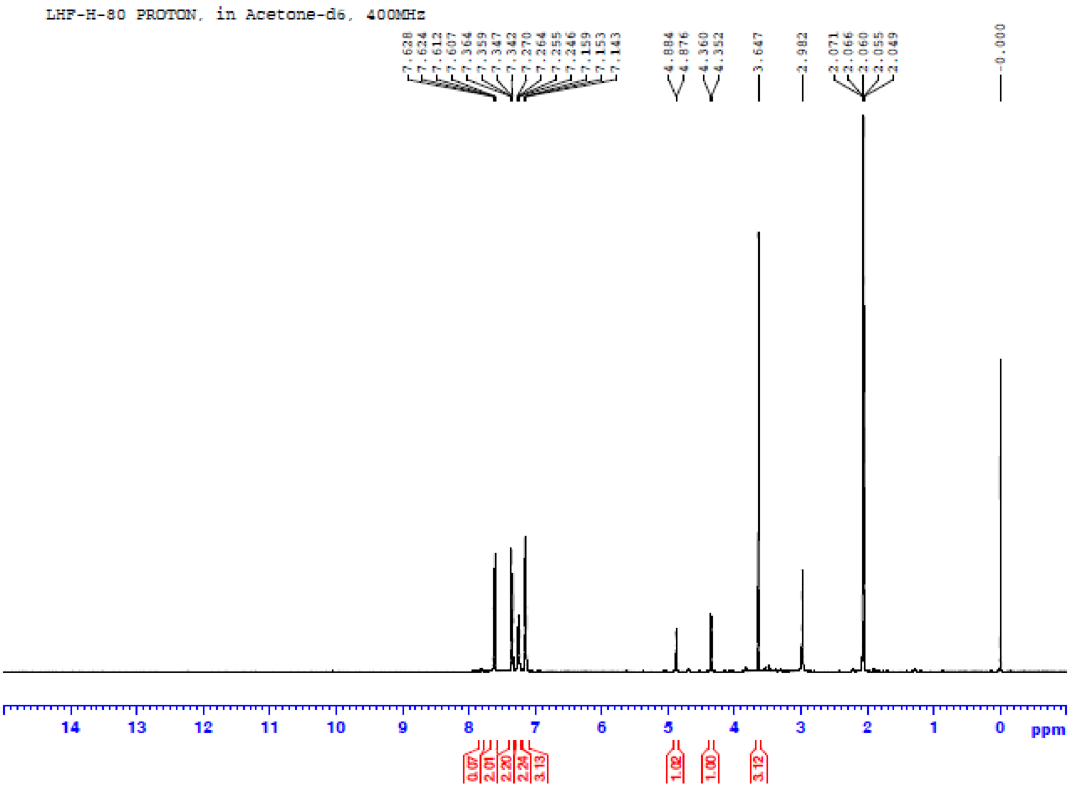

^{1}H-NMR of 7h

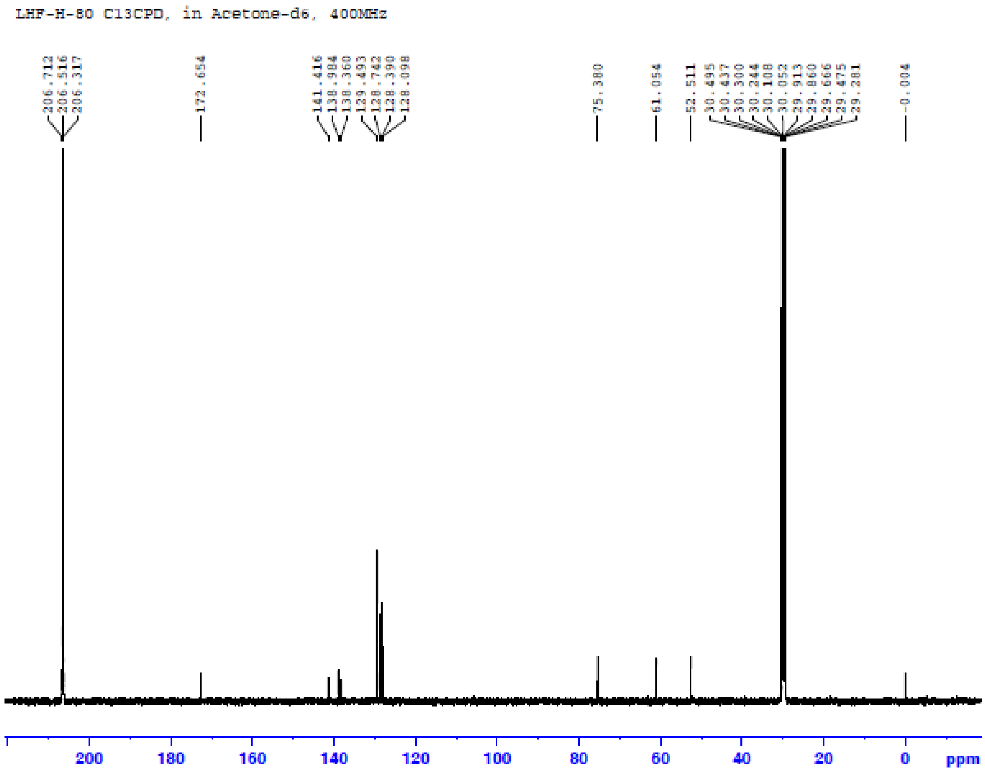

^{13}C-NMR of 7h

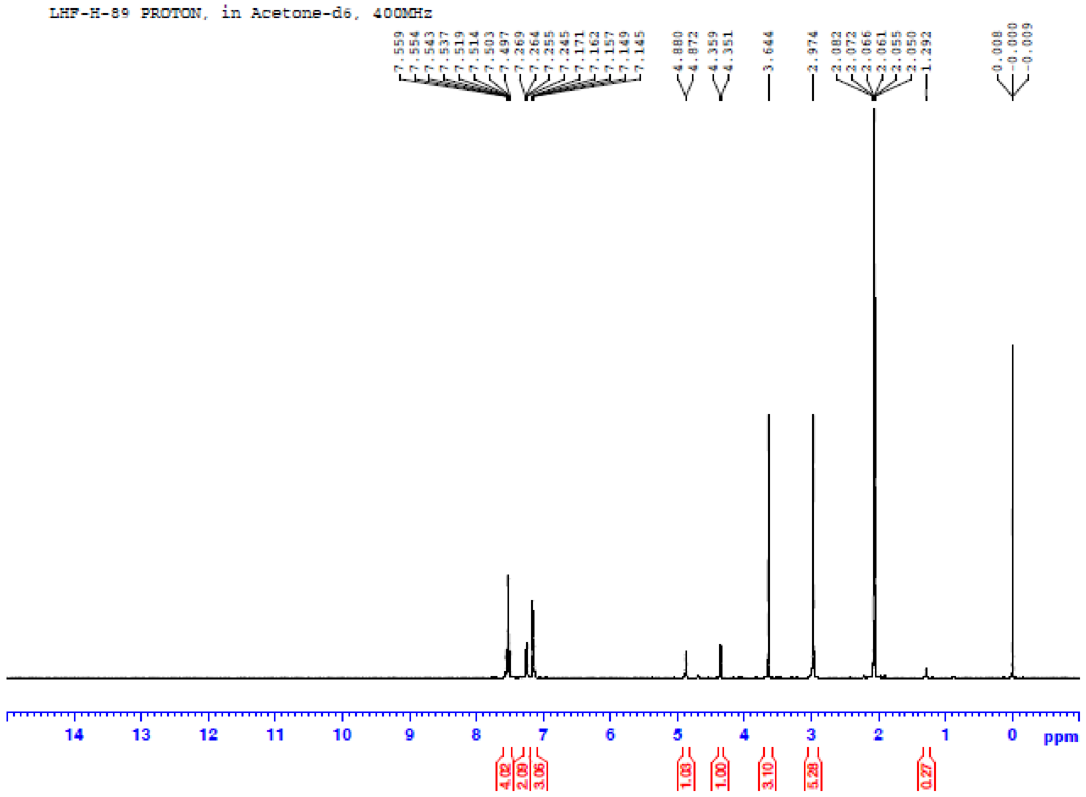

^{1}H-NMR of 7i

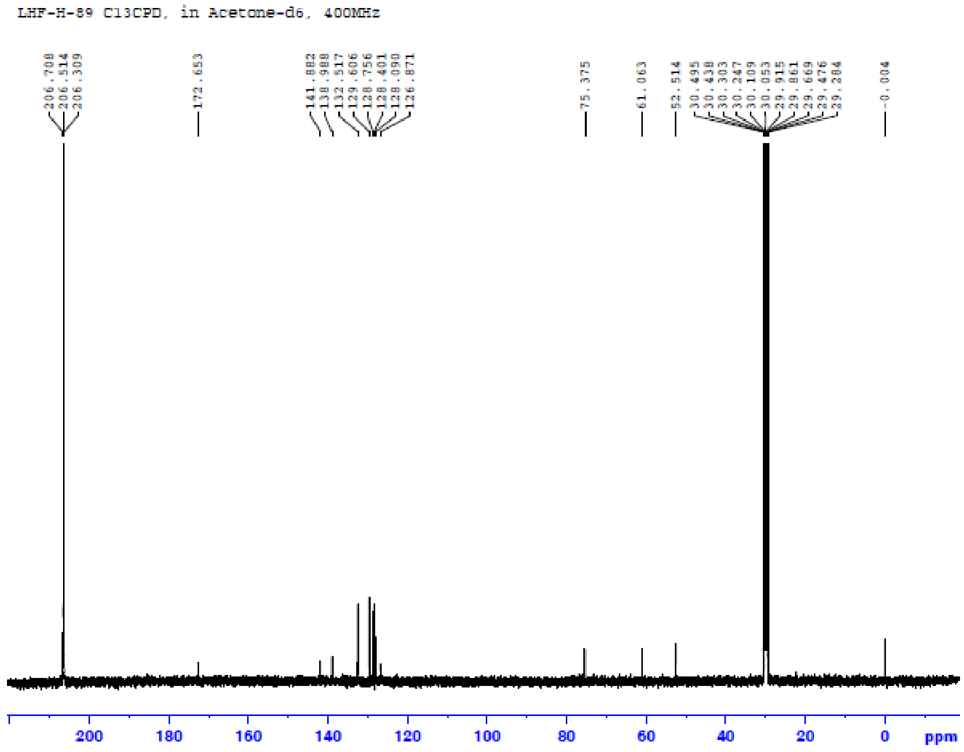

^{13}C-NMR of 7i

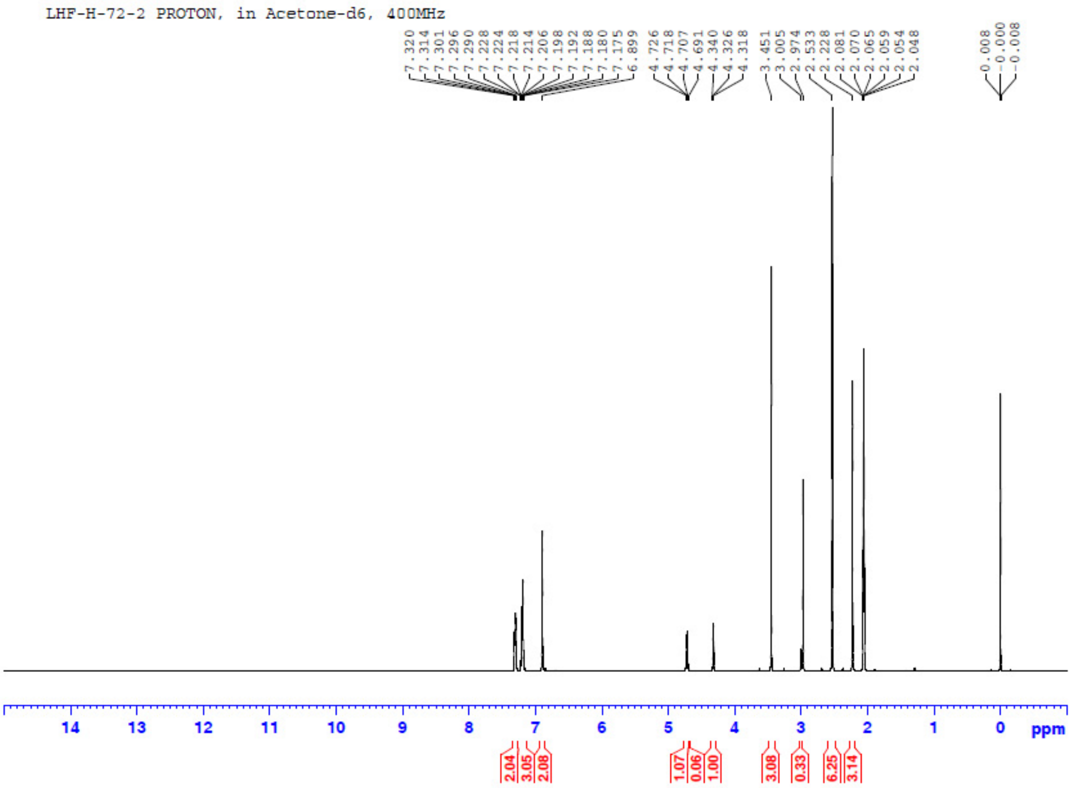

^{1}H-NMR of 7j

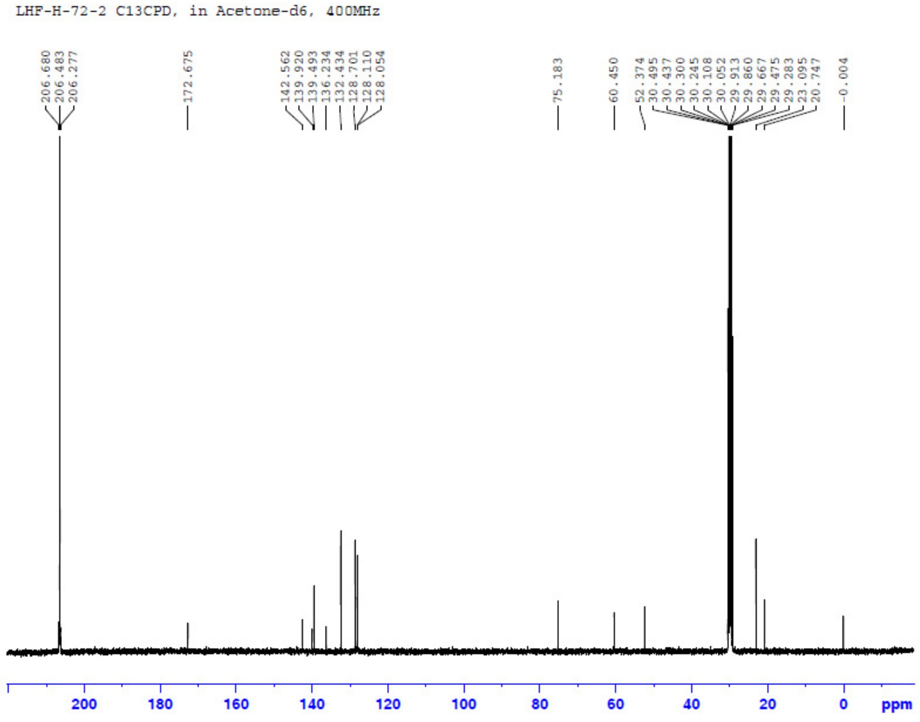

^{13}C-NMR of 7j

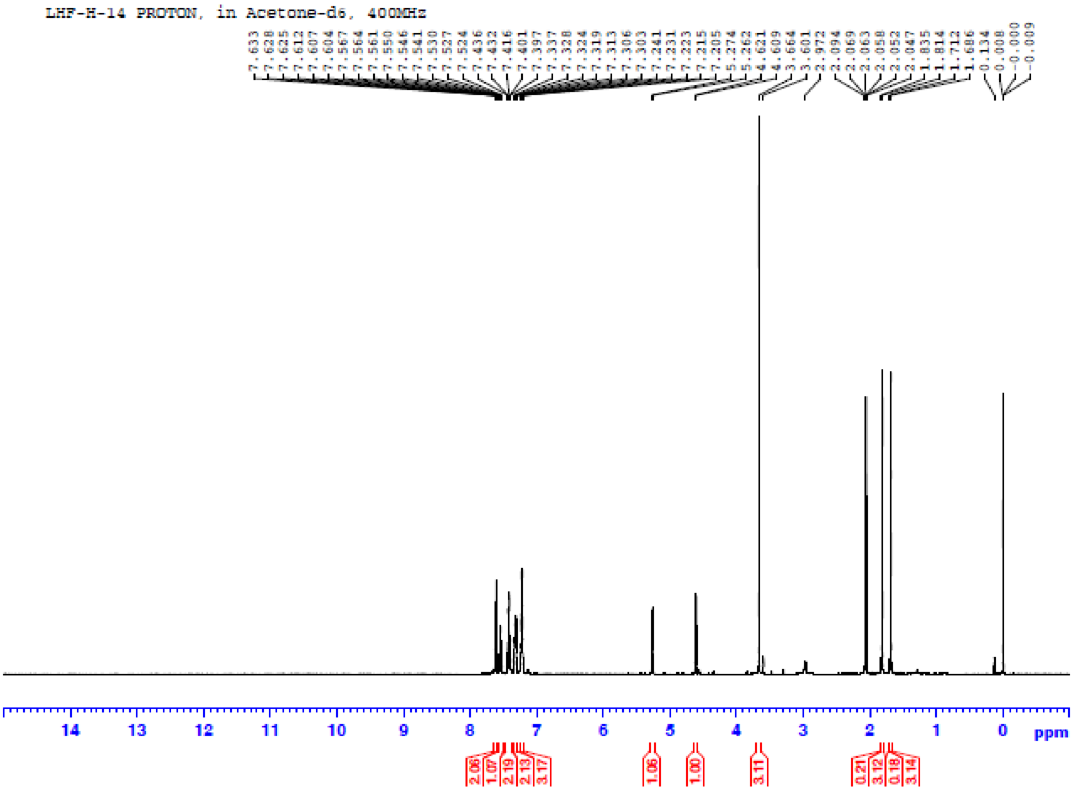

^{1}H-NMR of 11a

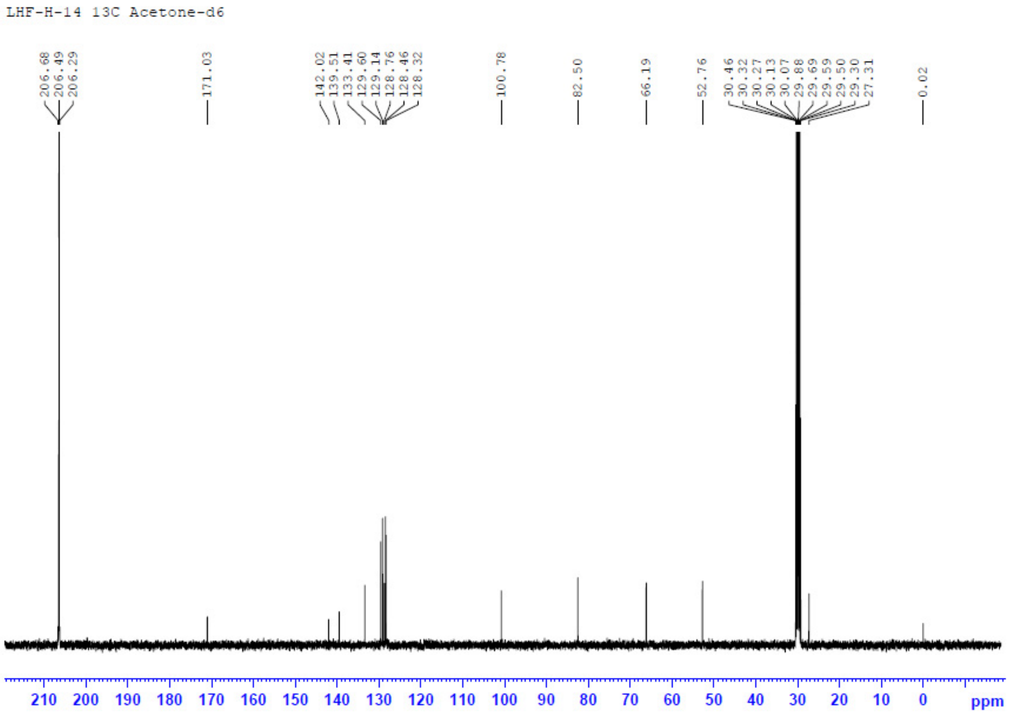

^{13}C-NMR of 11a

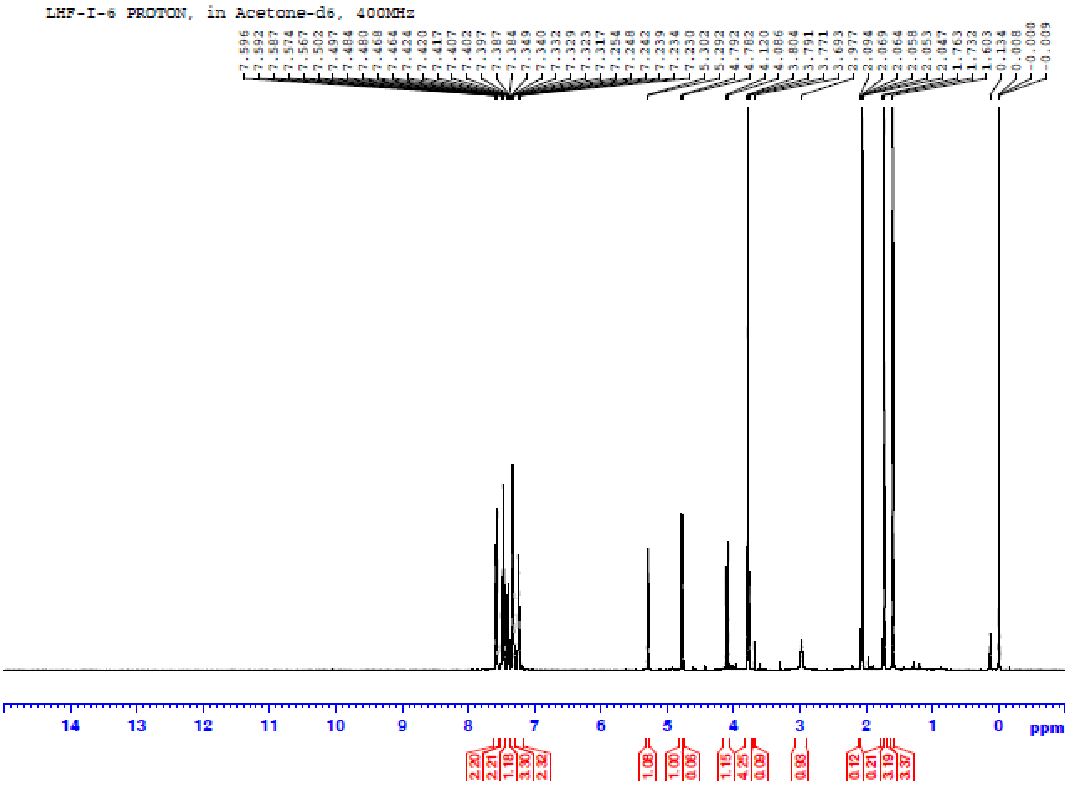

^{1}H-NMR of 11b

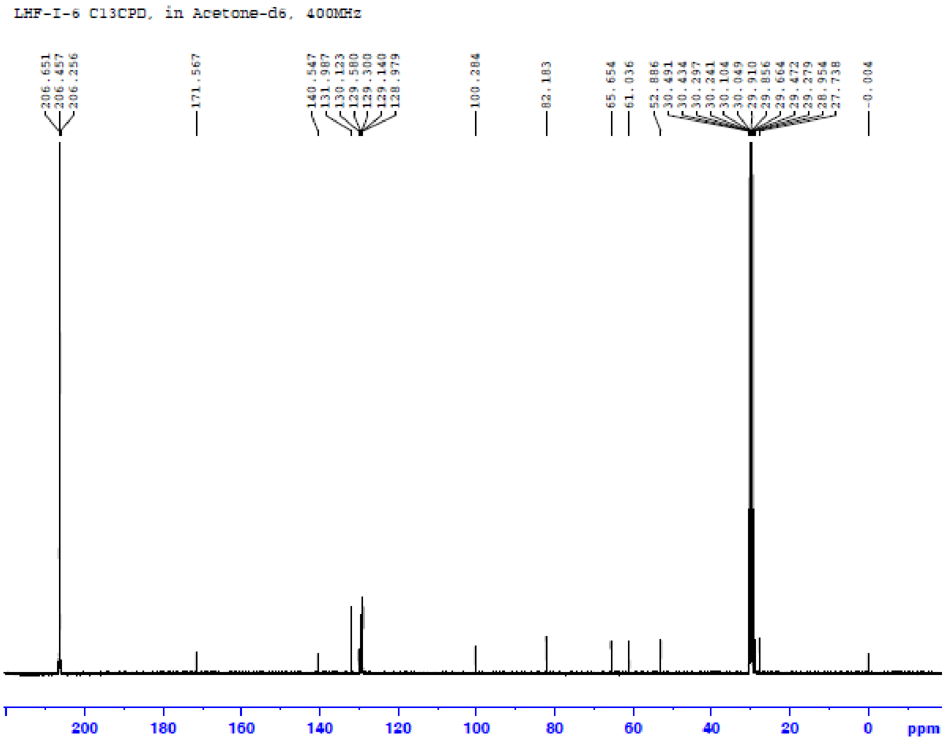

^{13}C-NMR of 11b

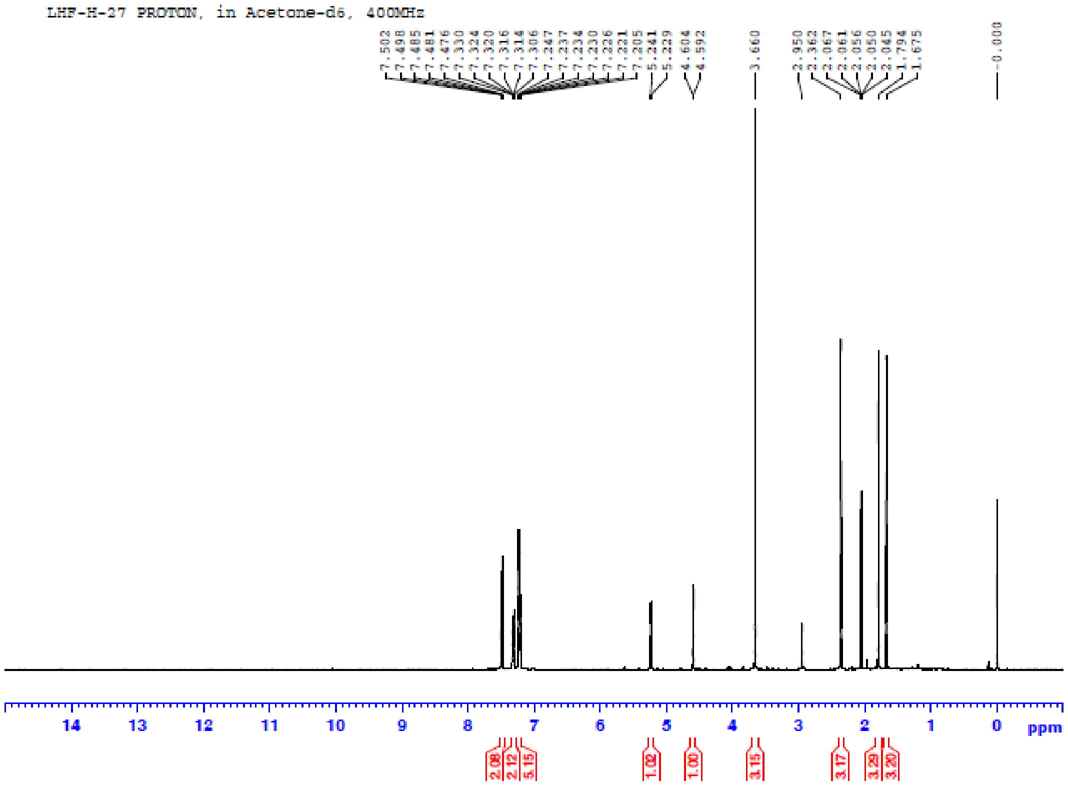

^{1}H-NMR of 11c

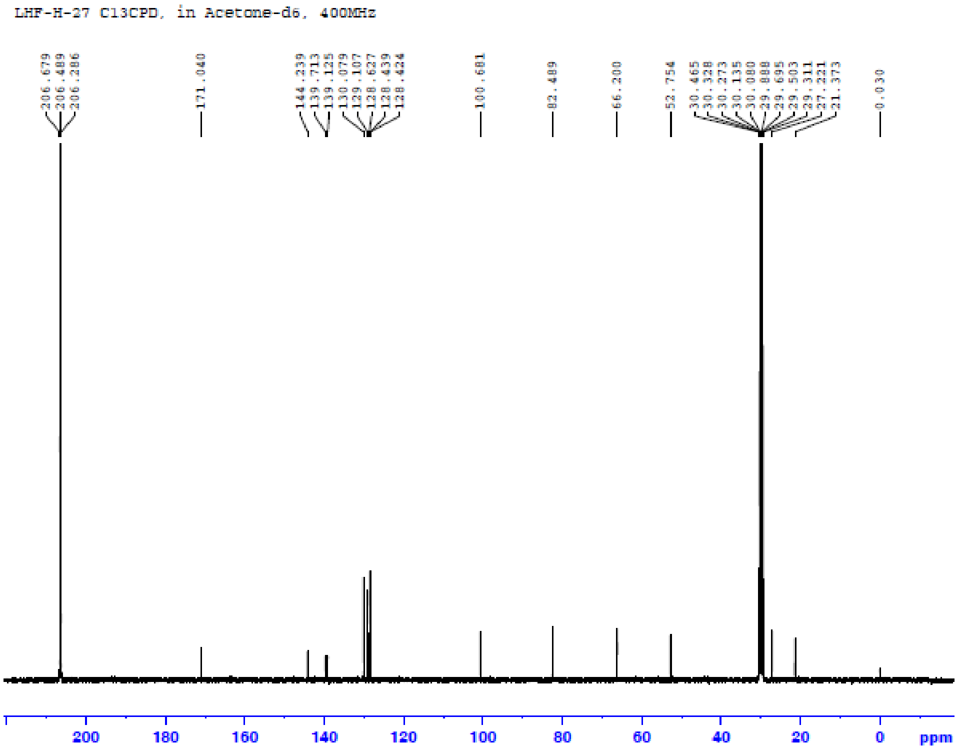

^{13}C-NMR of 11c

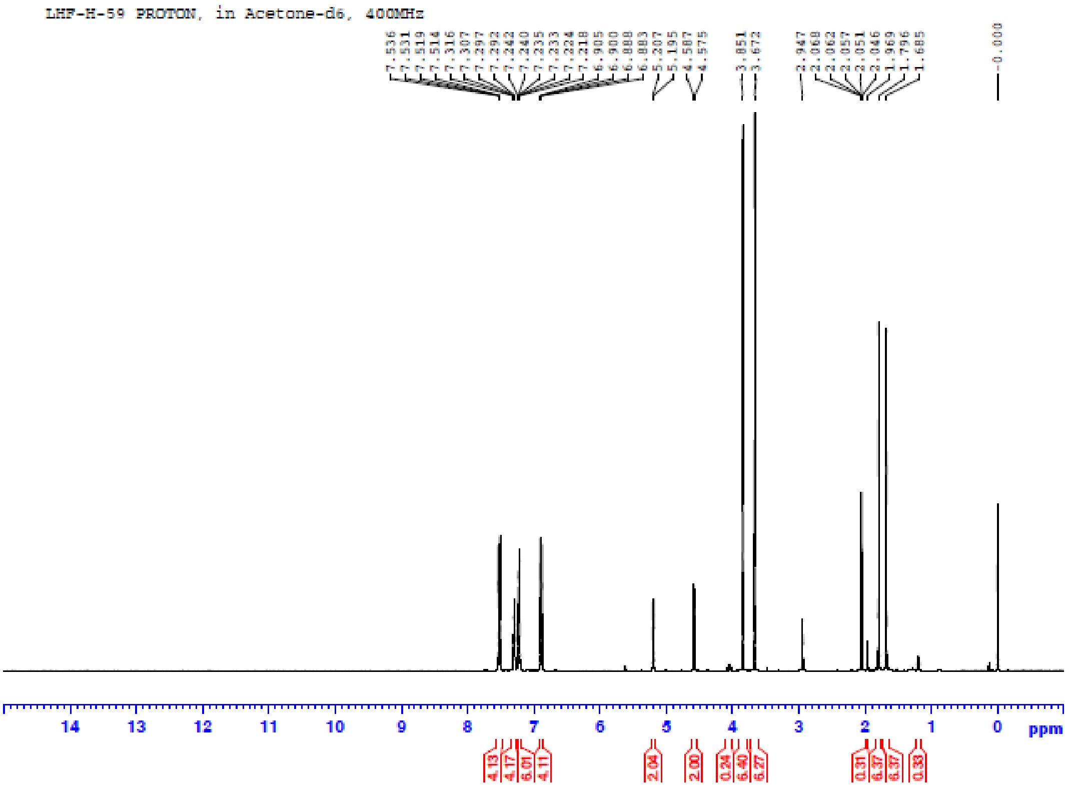

^{1}H-NMR of 11d

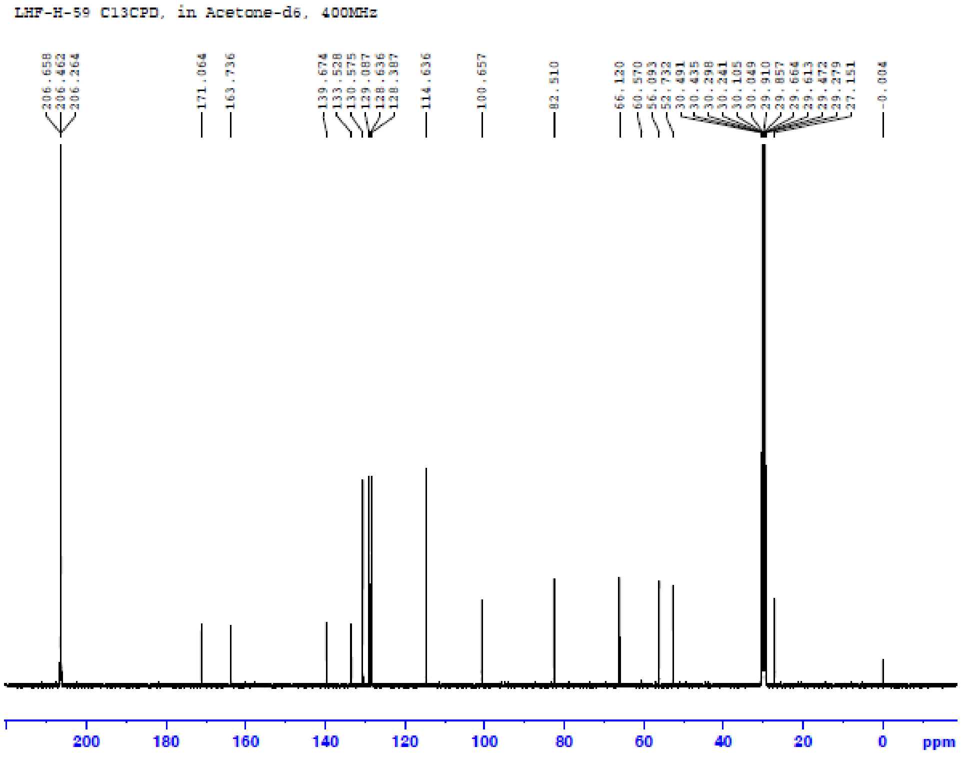

^{13}C-NMR of 11d

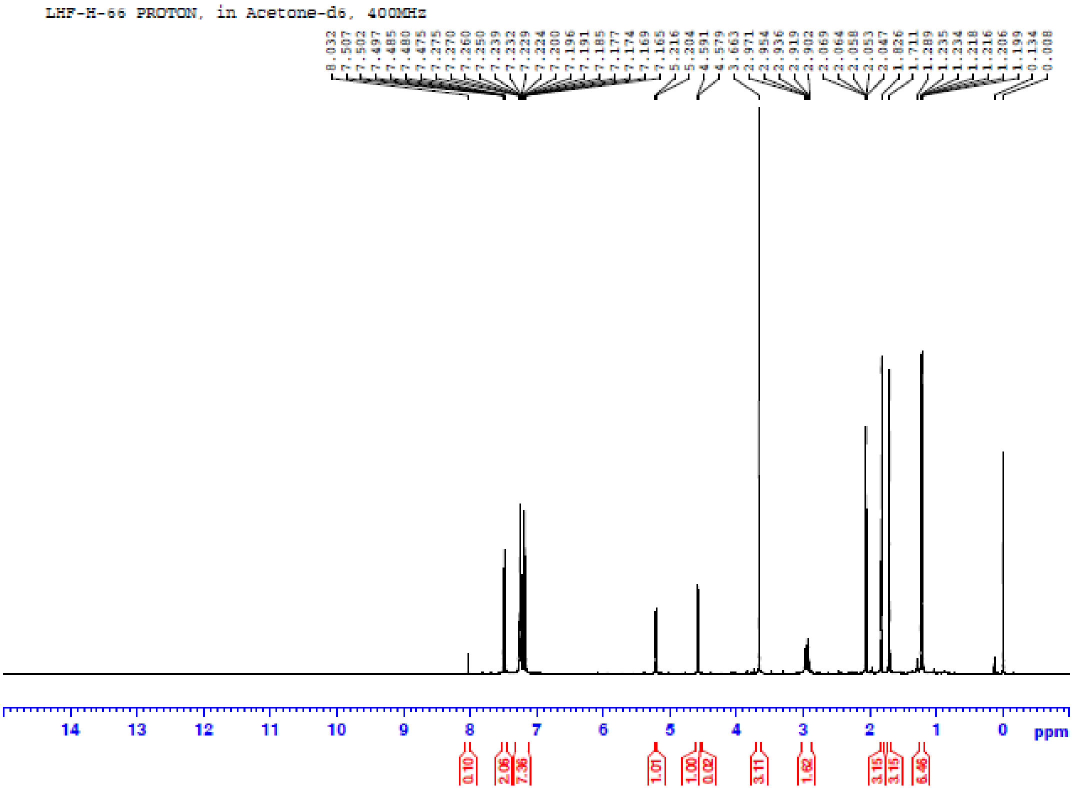

^{1}H-NMR of 11e

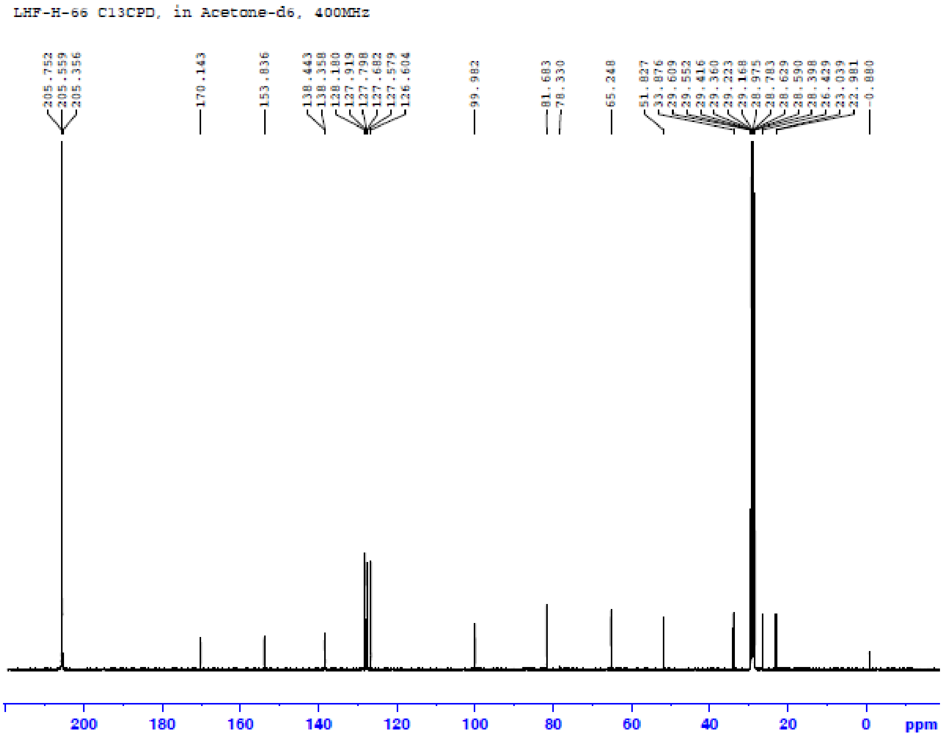

^{13}C-NMR of 11e

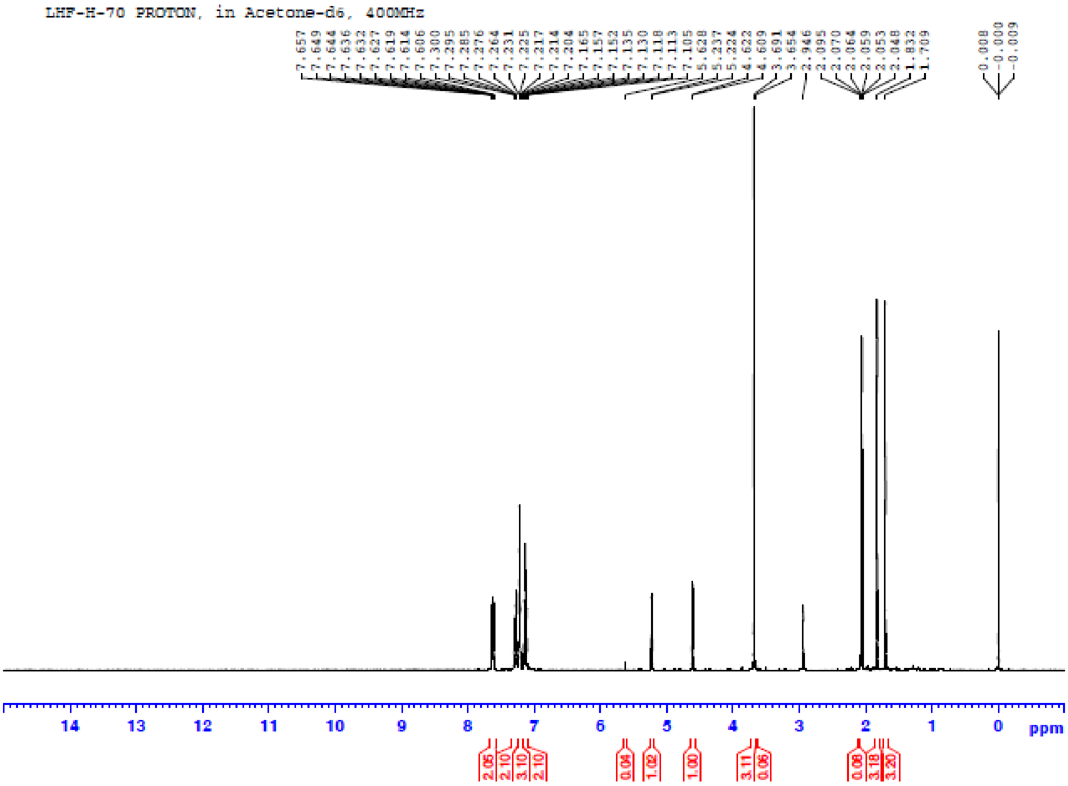

^{1}H-NMR of 11f

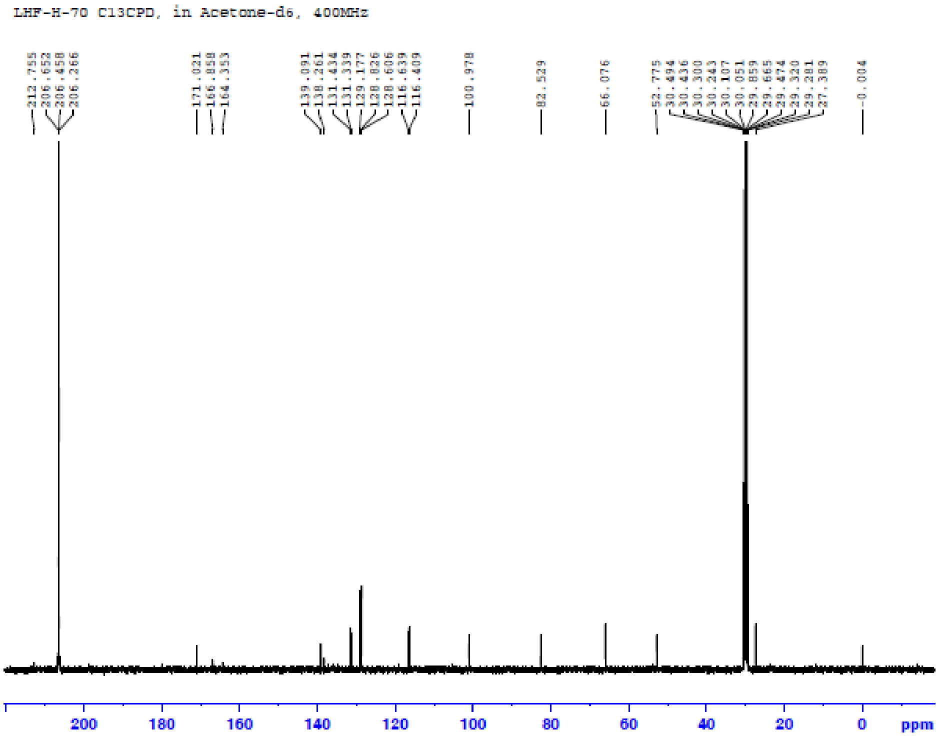

^{13}C-NMR of 11f

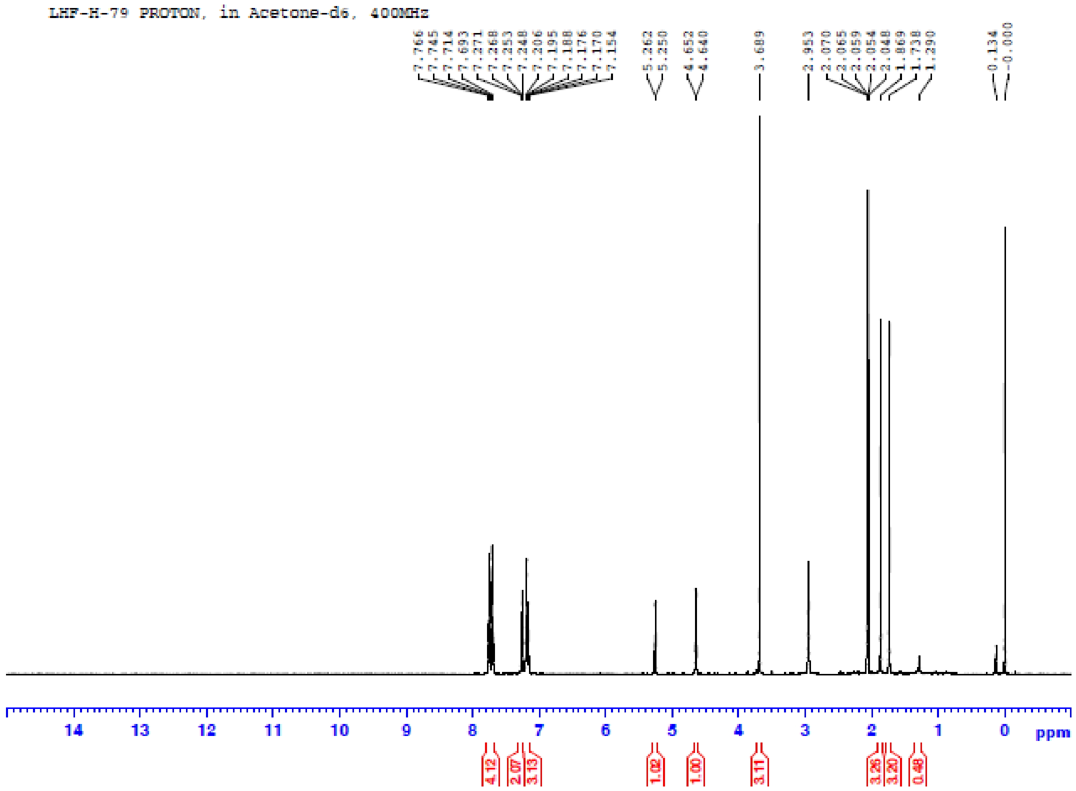

^{1}H-NMR of 11g

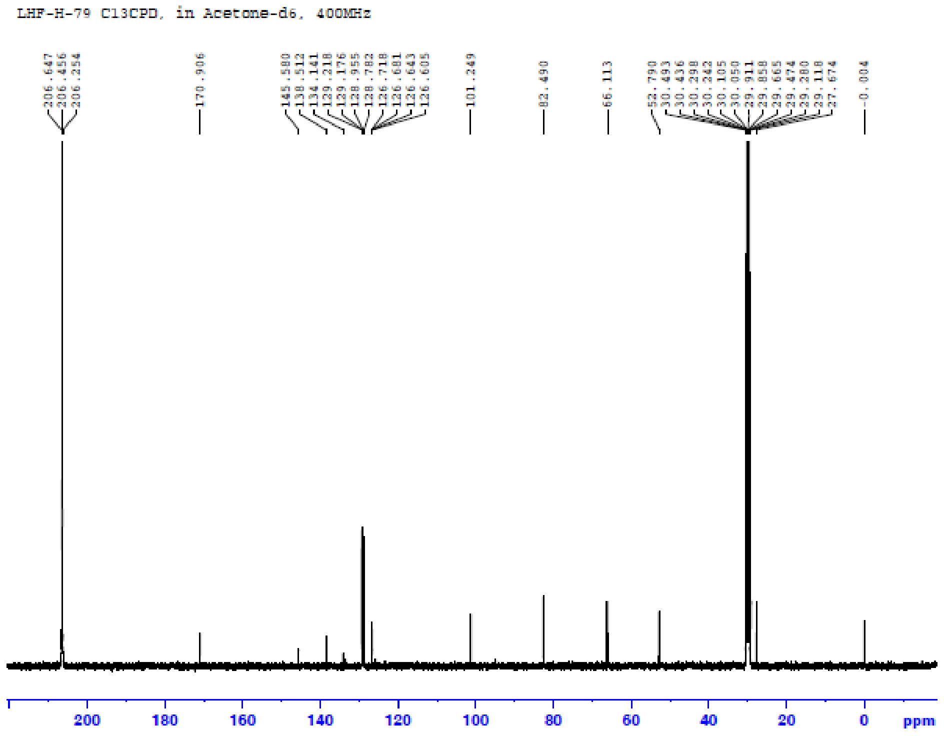

^{13}C-NMR of 11g

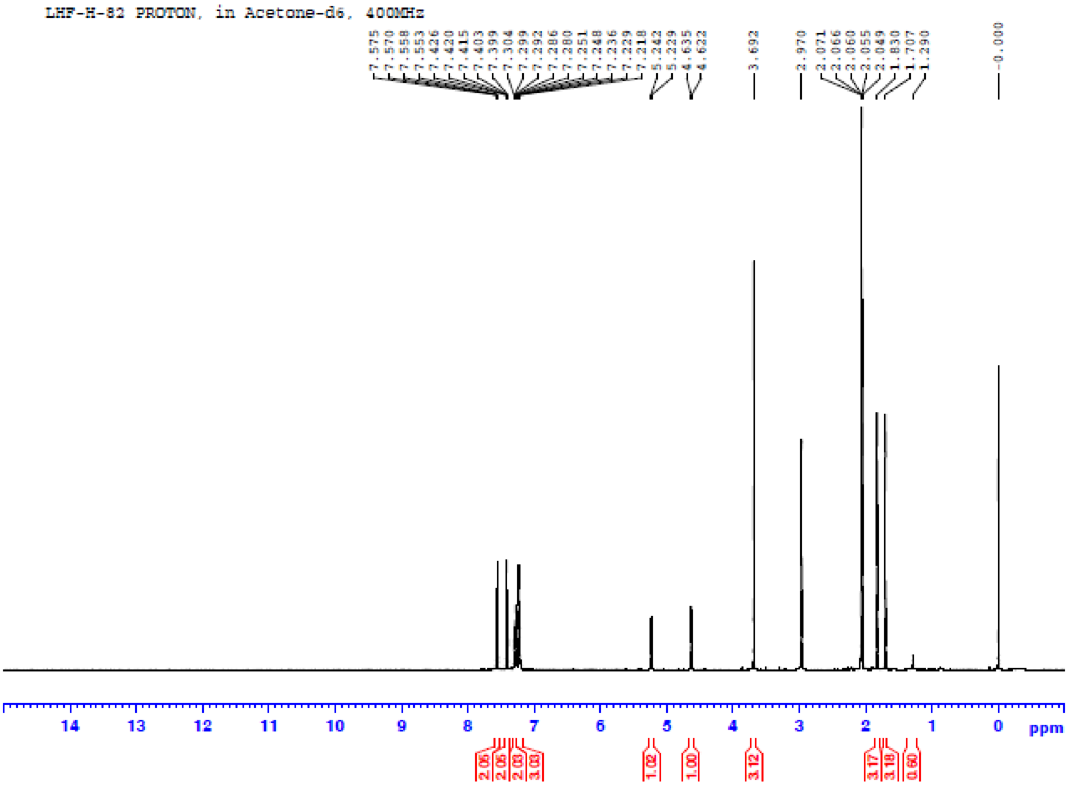

^{1}H-NMR of 11h

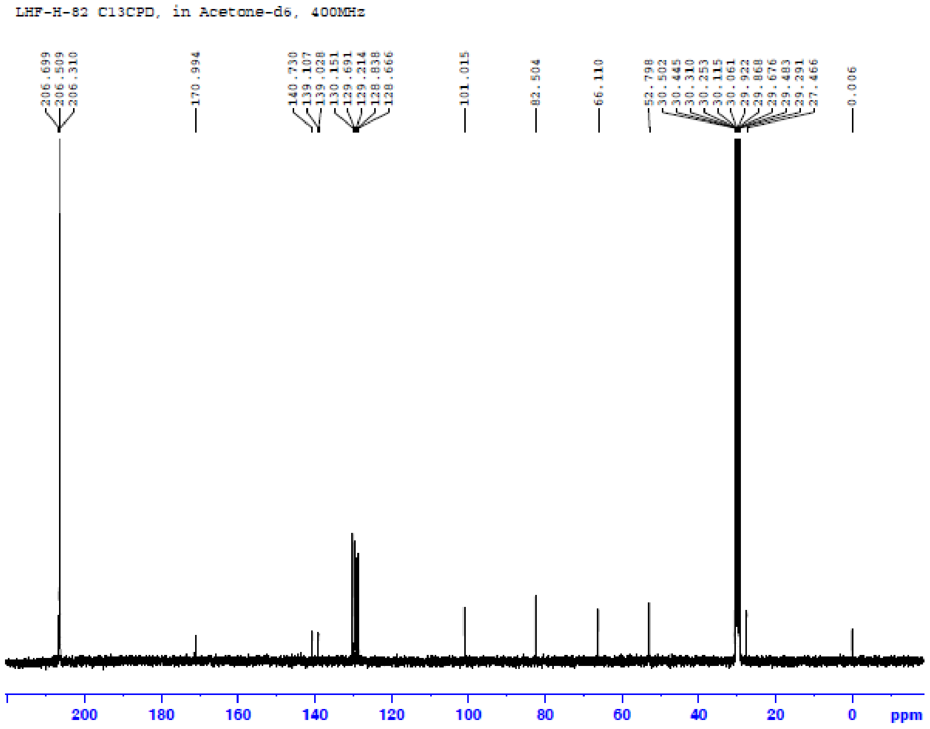

^{13}C-NMR of 11h

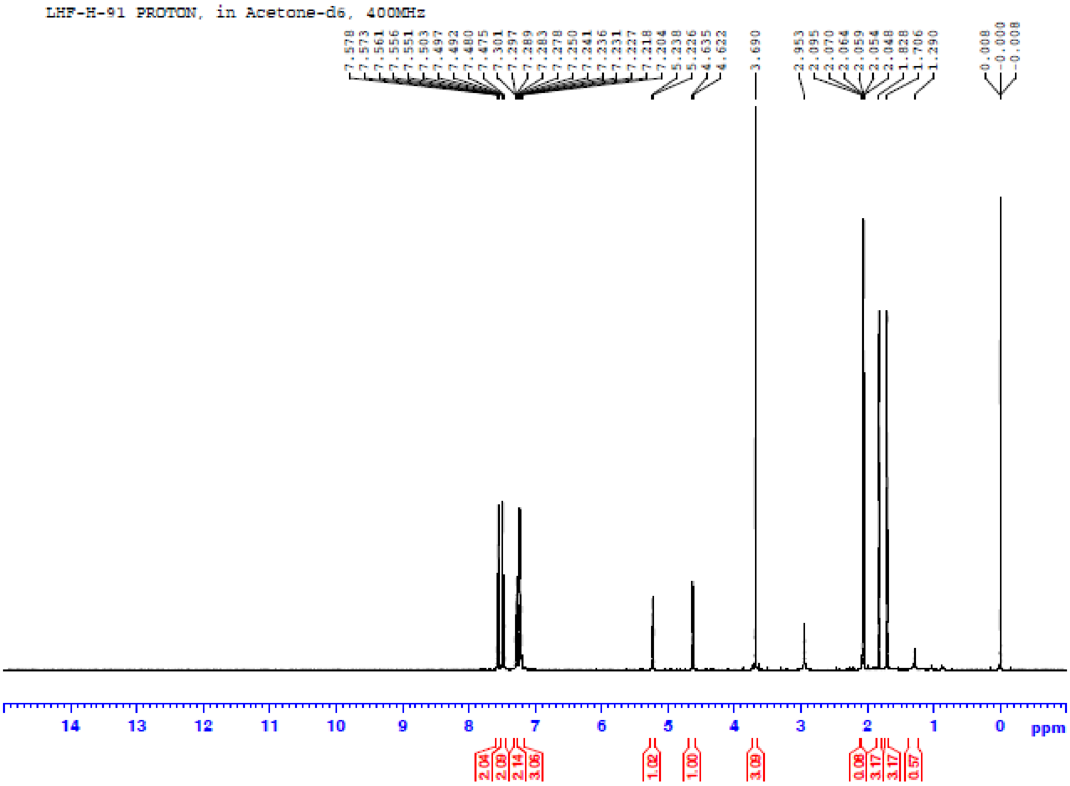

^{1}H-NMR of 11i

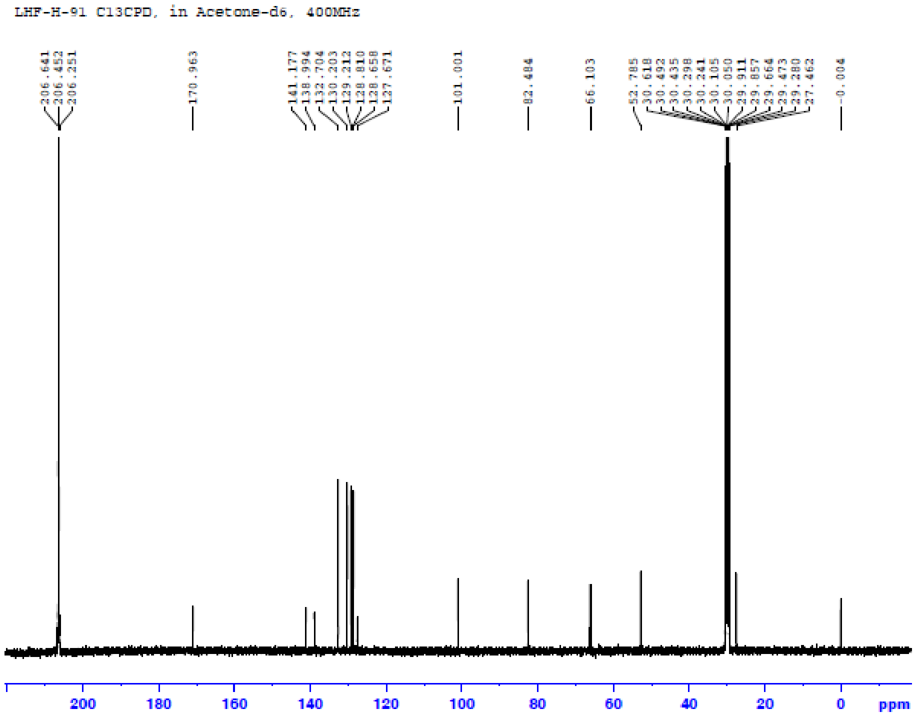

^{13}C-NMR of 11i

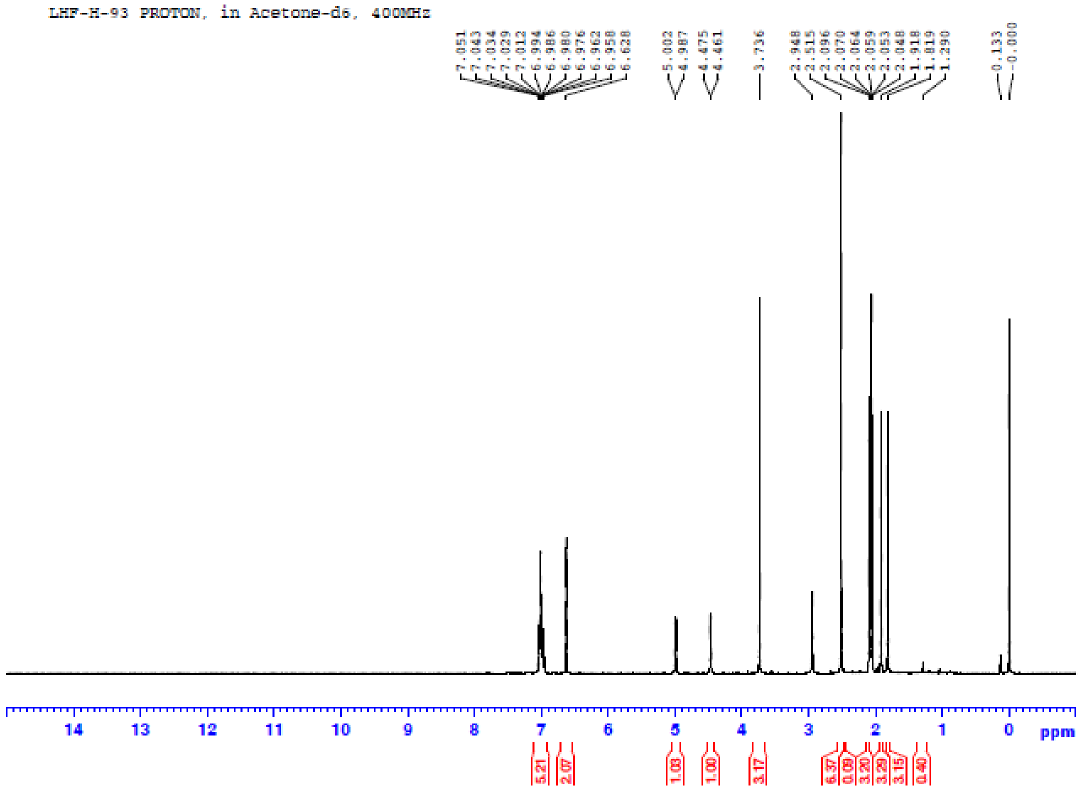

^{1}H-NMR of 11j

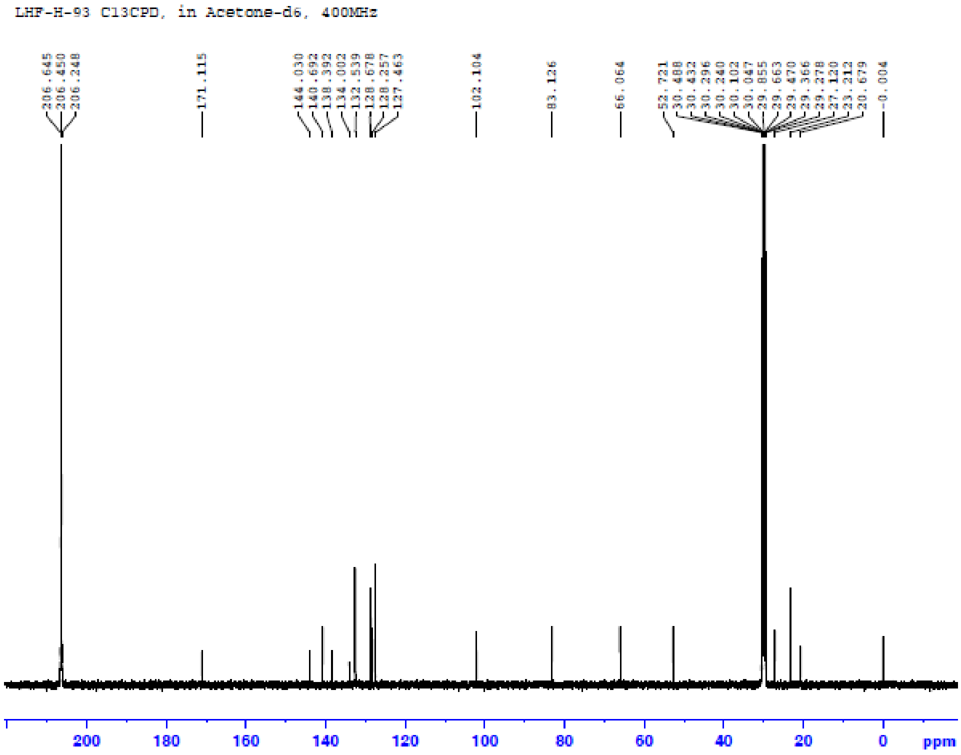

^{13}C-NMR of 11j

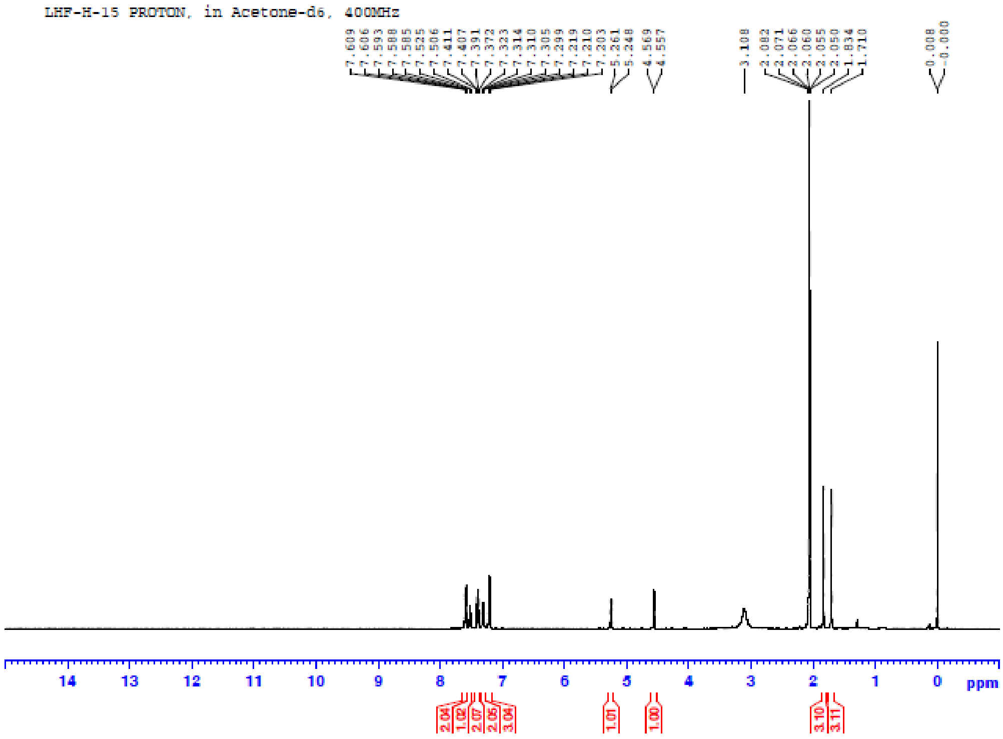

^{1}H-NMR of 12a

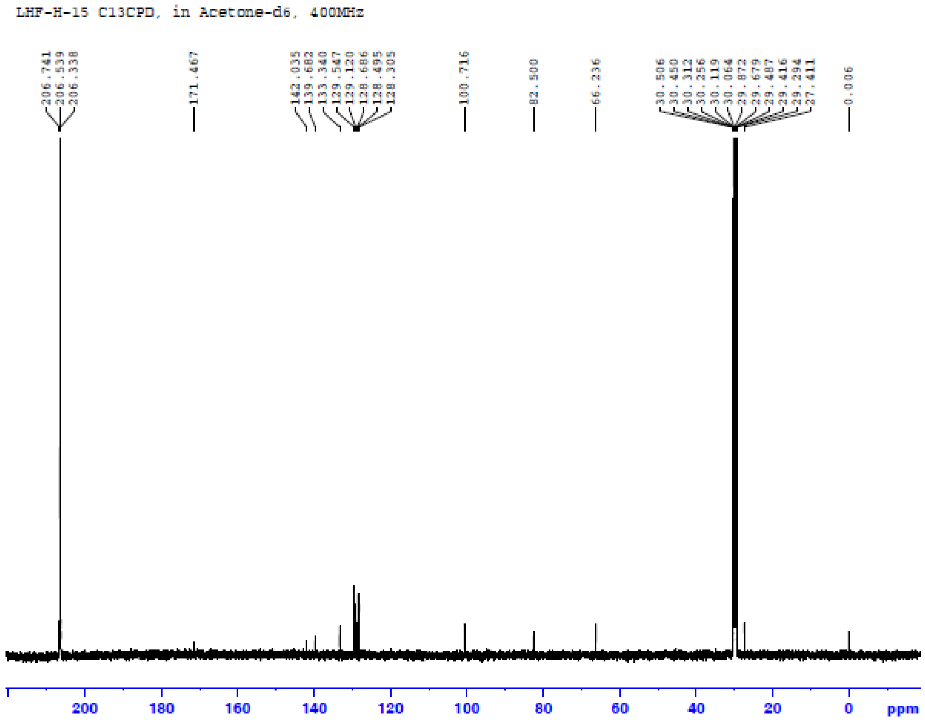

^{13}C-NMR of 12a

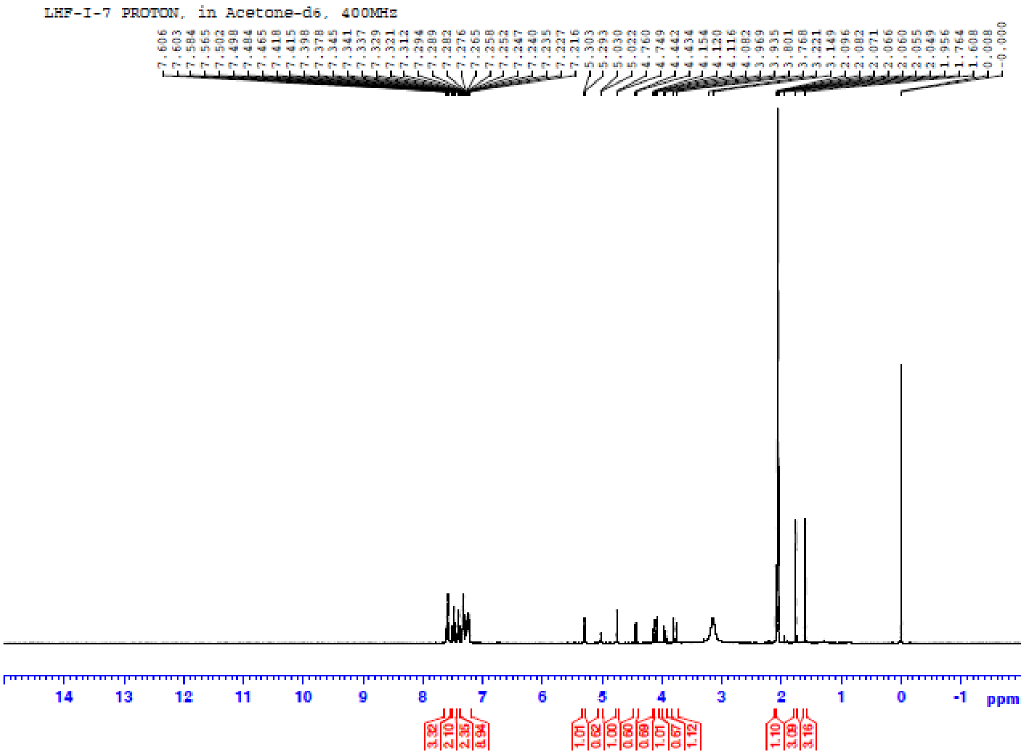

^{1}H-NMR of 12b

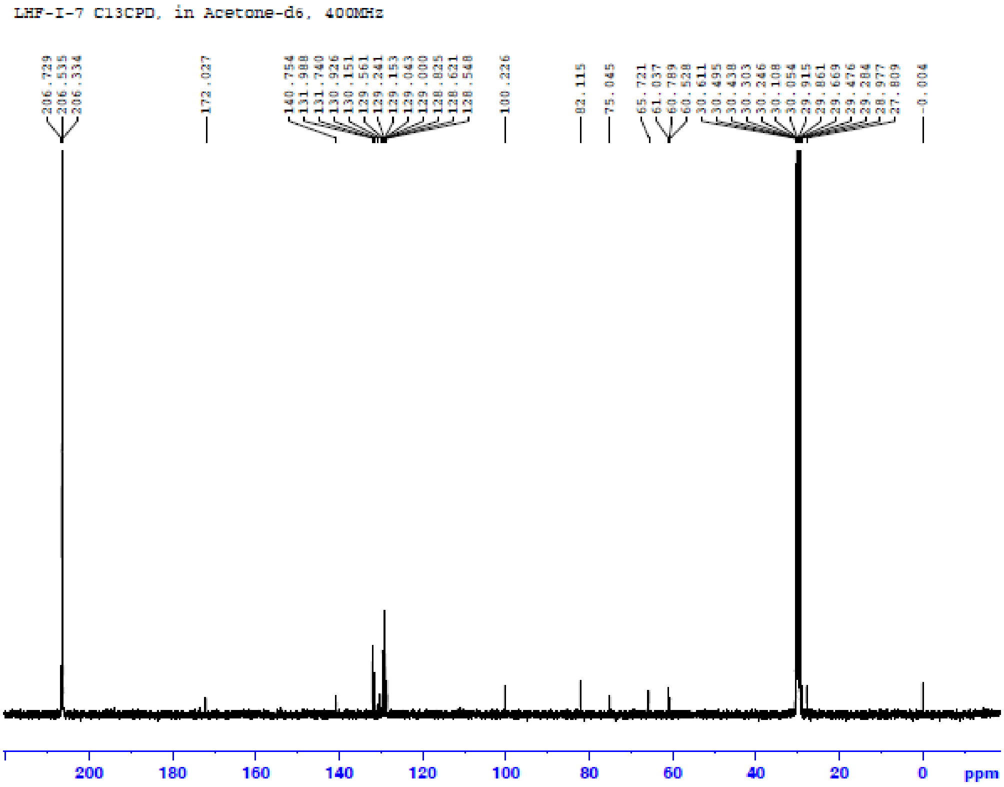

^{13}C-NMR of 12b

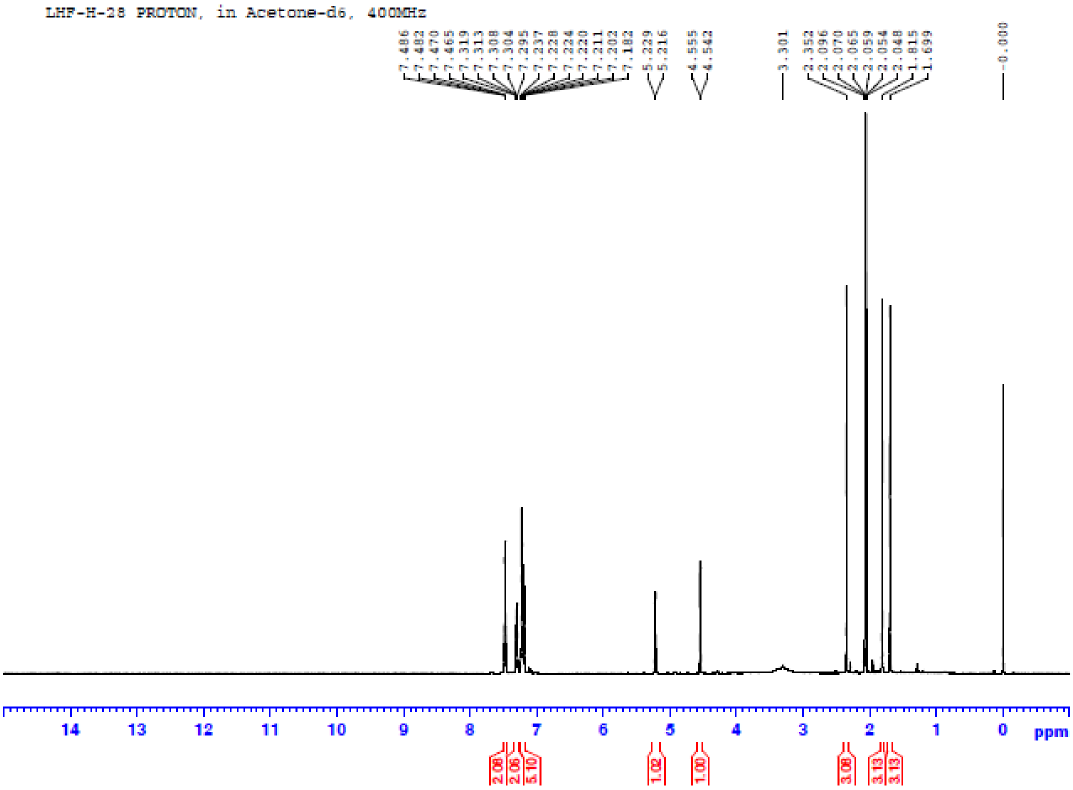

^{1}H-NMR of 12c

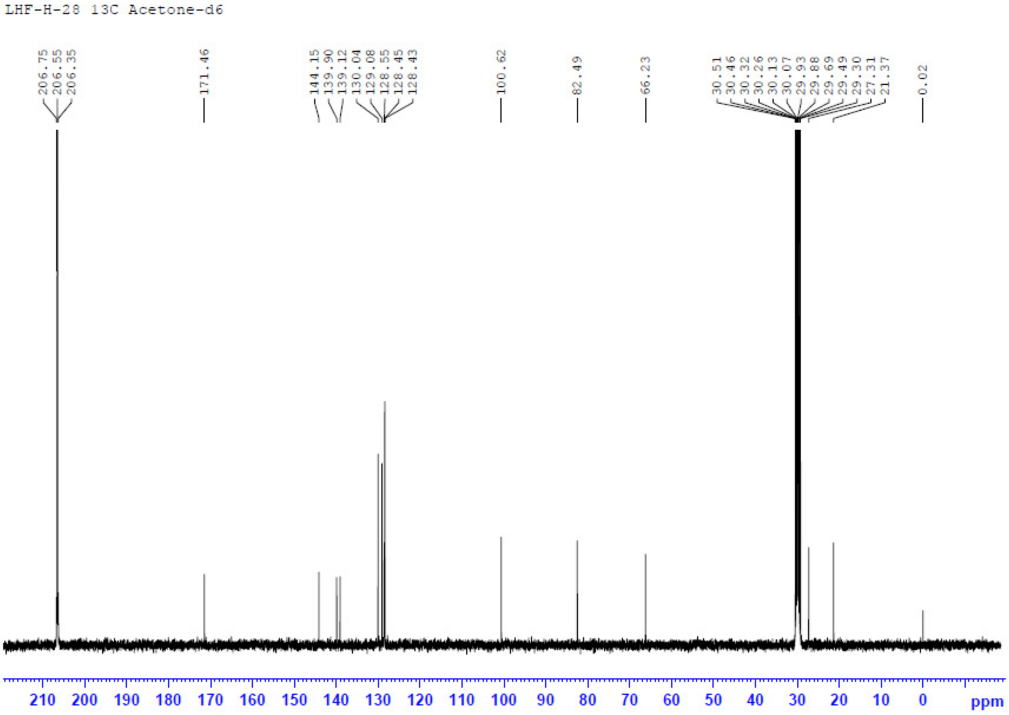

^{13}C-NMR of 12c

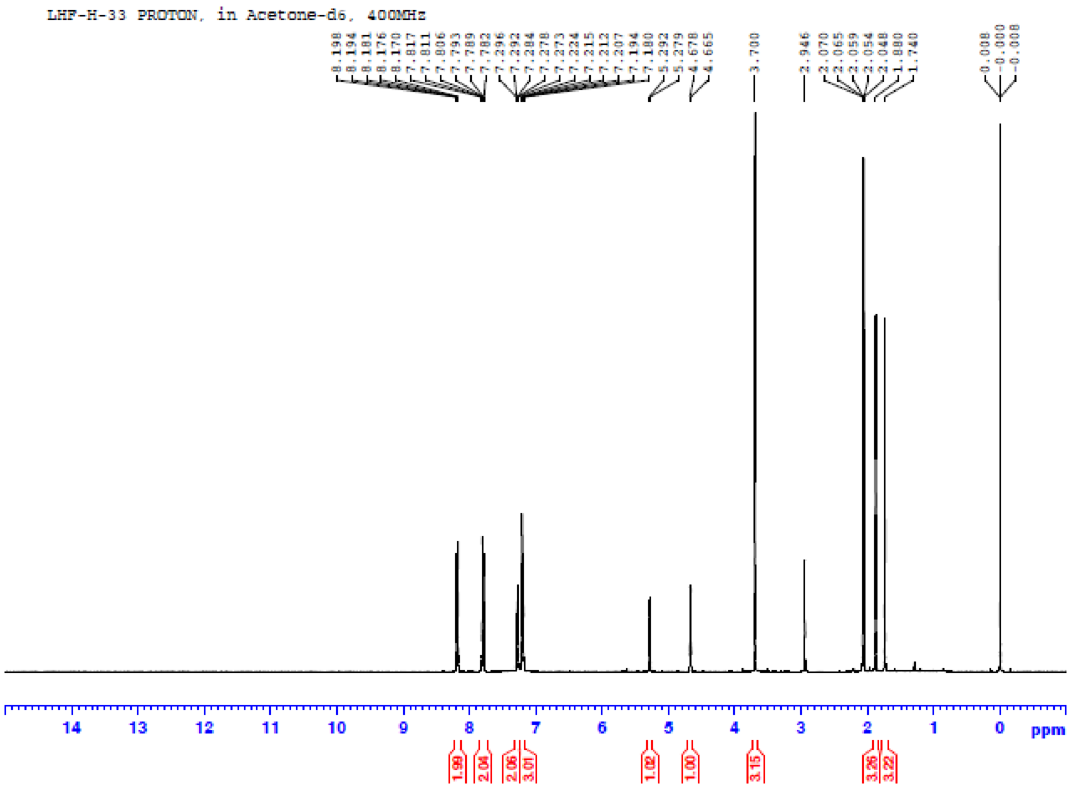

^{1}H-NMR of 12d

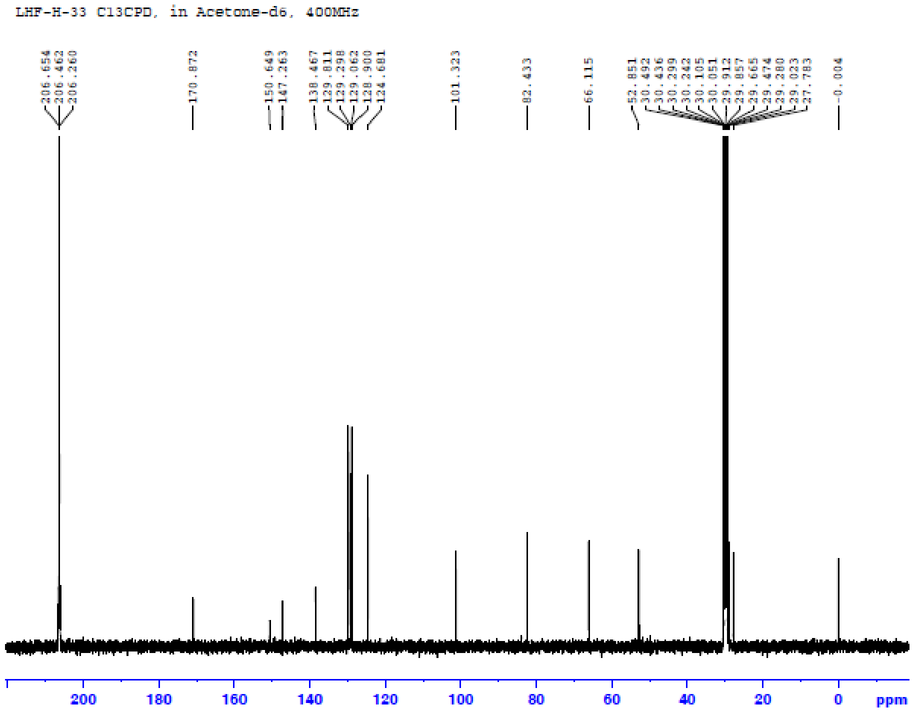

^{13}C-NMR of 12d

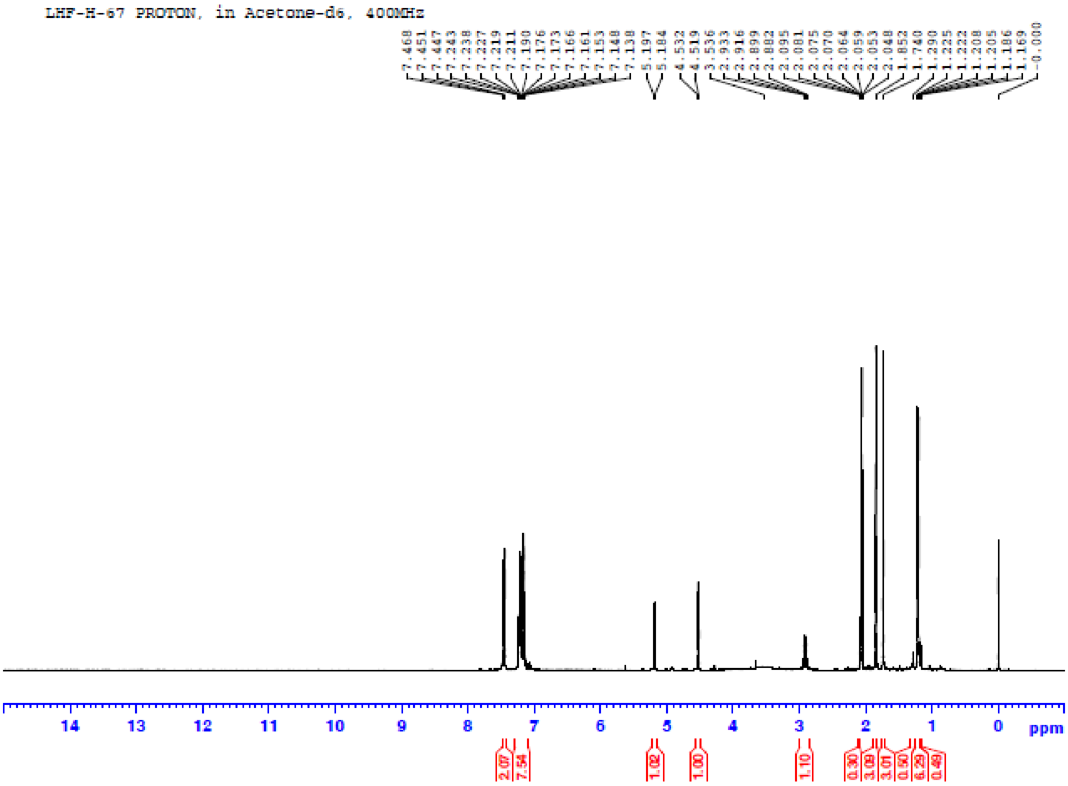

^{1}H-NMR of 12e

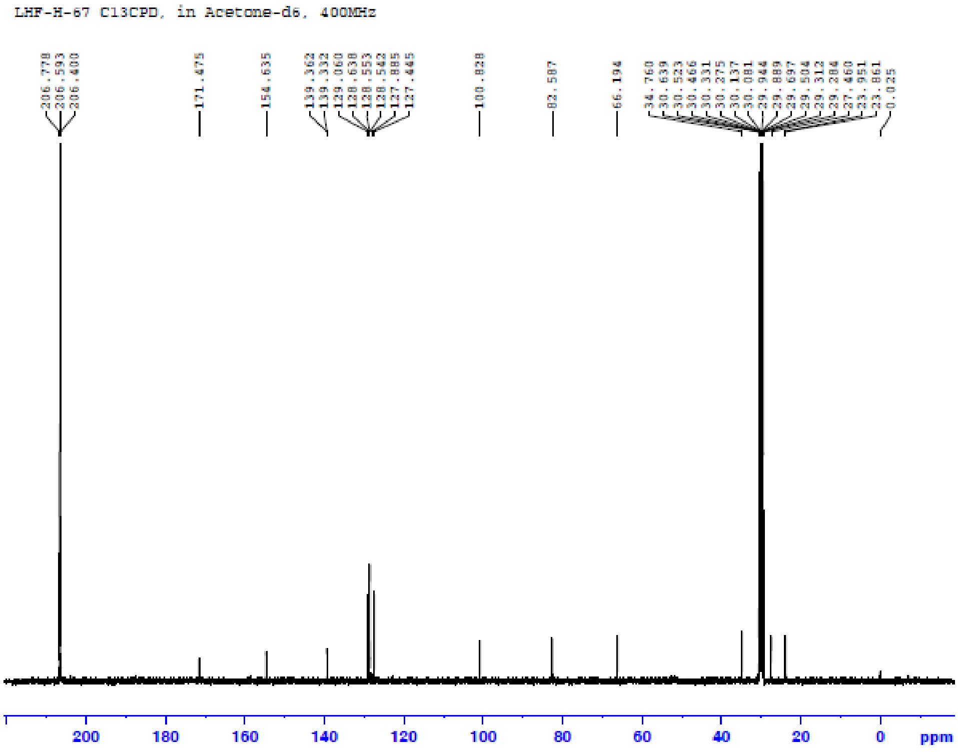

^{13}C-NMR of 12e

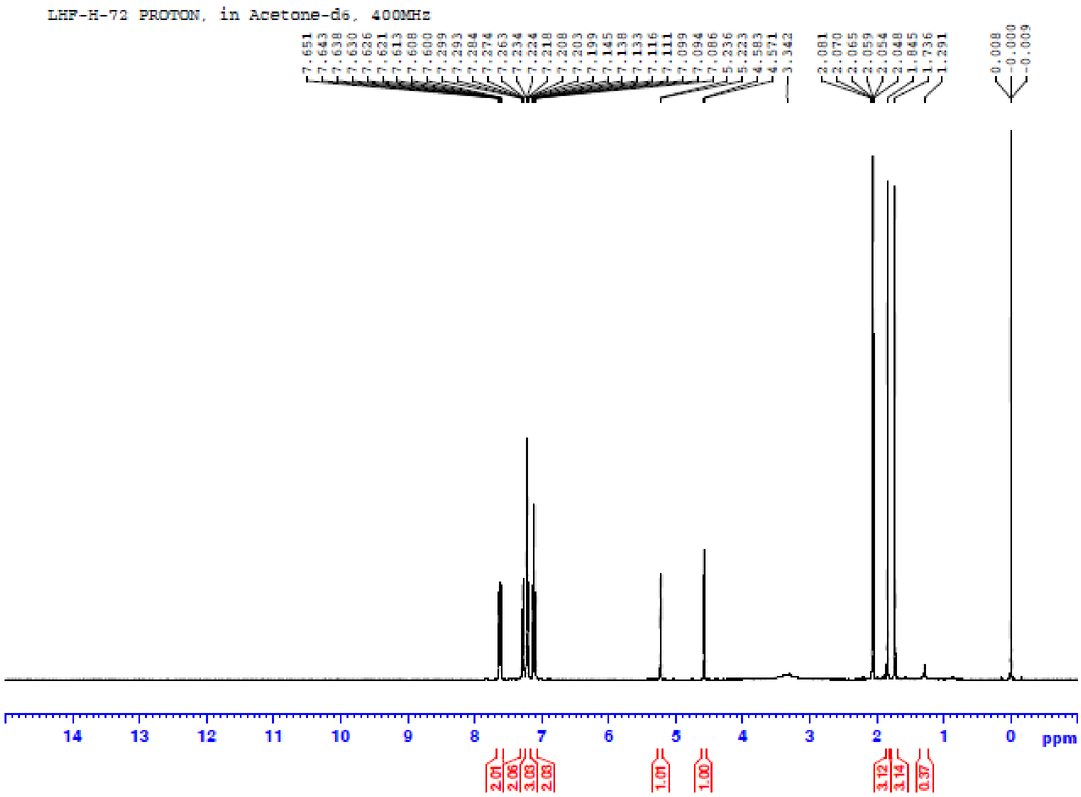

^{1}H-NMR of 12f

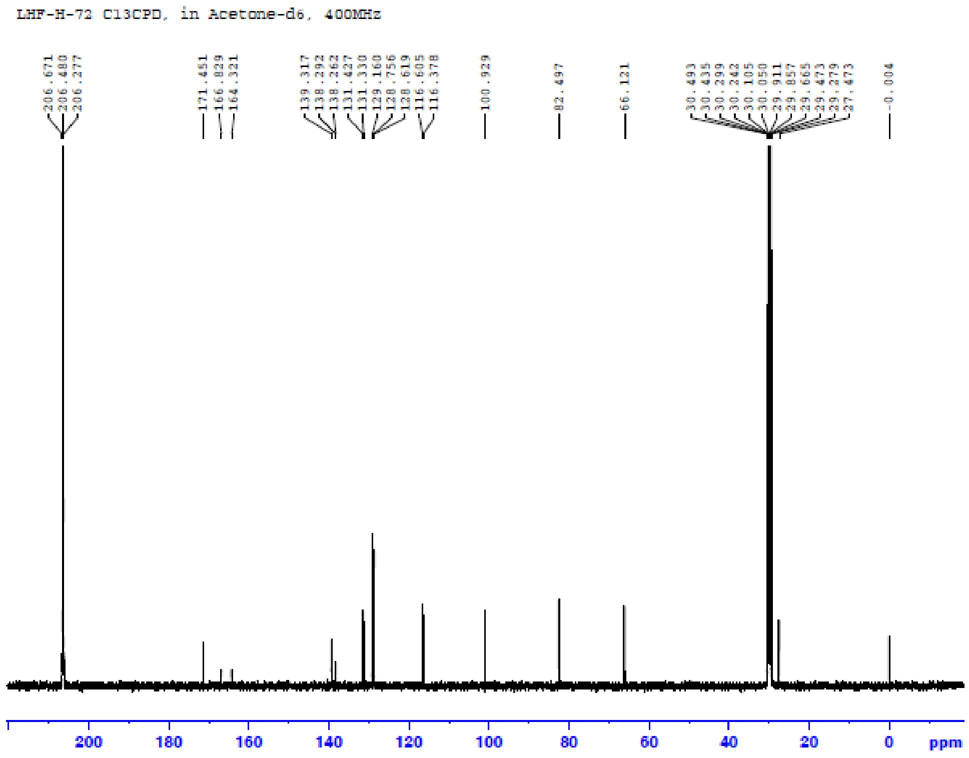

^{13}C-NMR of 12f

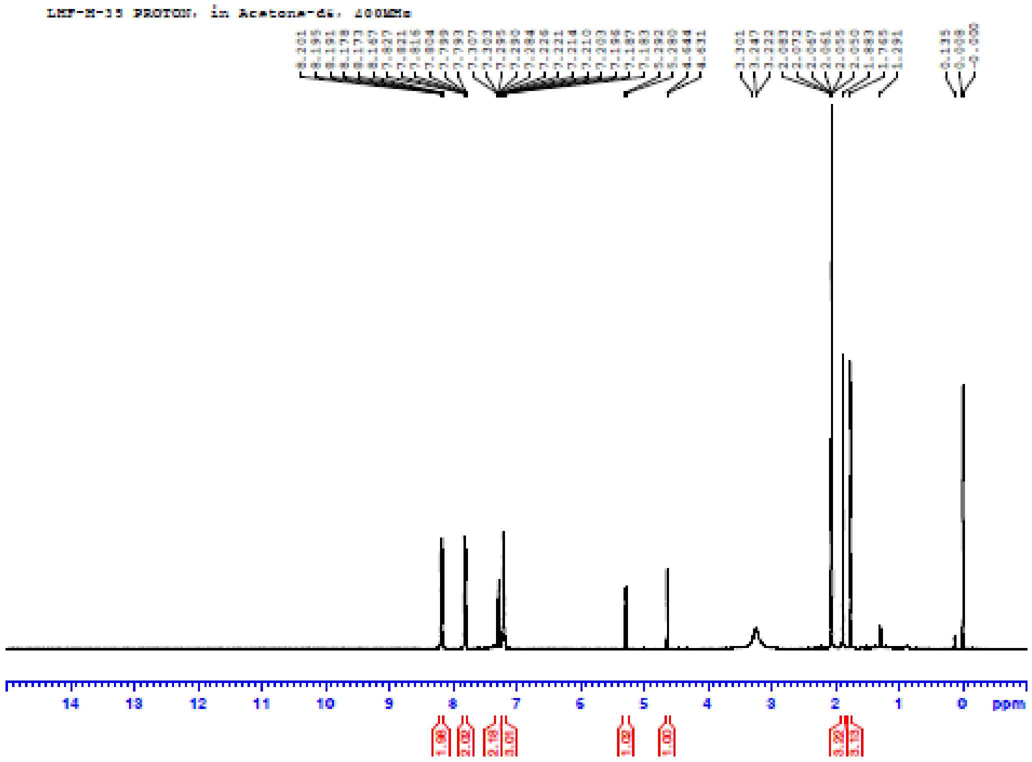

^{1}H-NMR of 12g

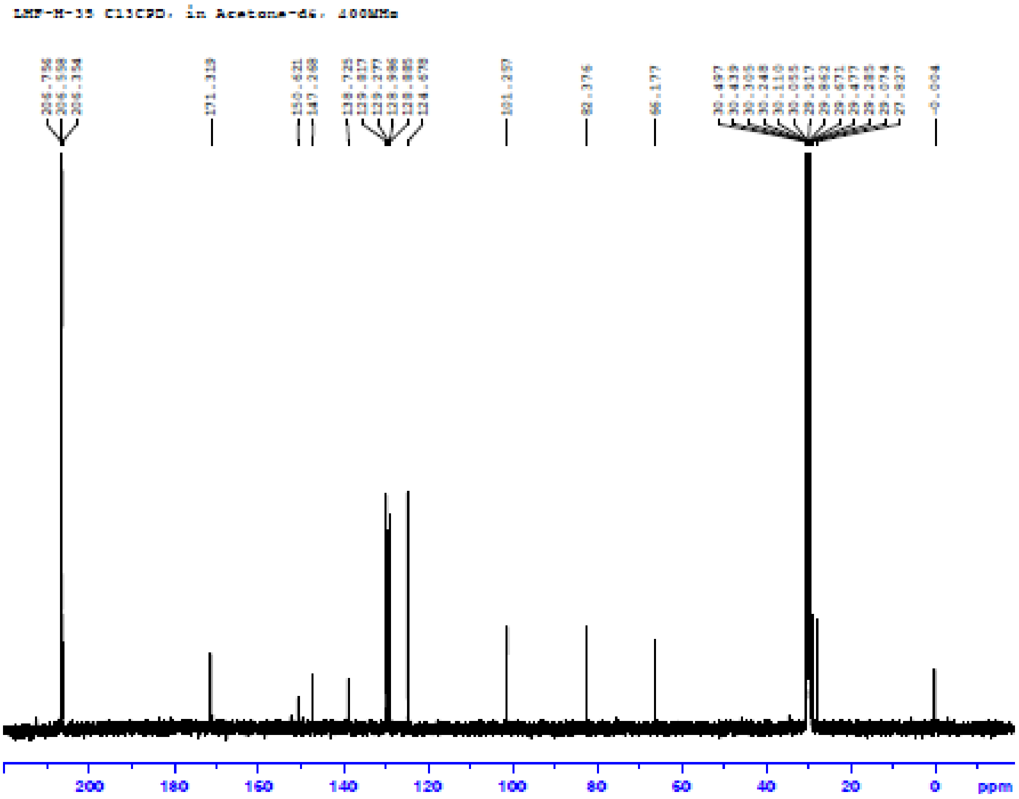

^{13}C-NMR of 12g

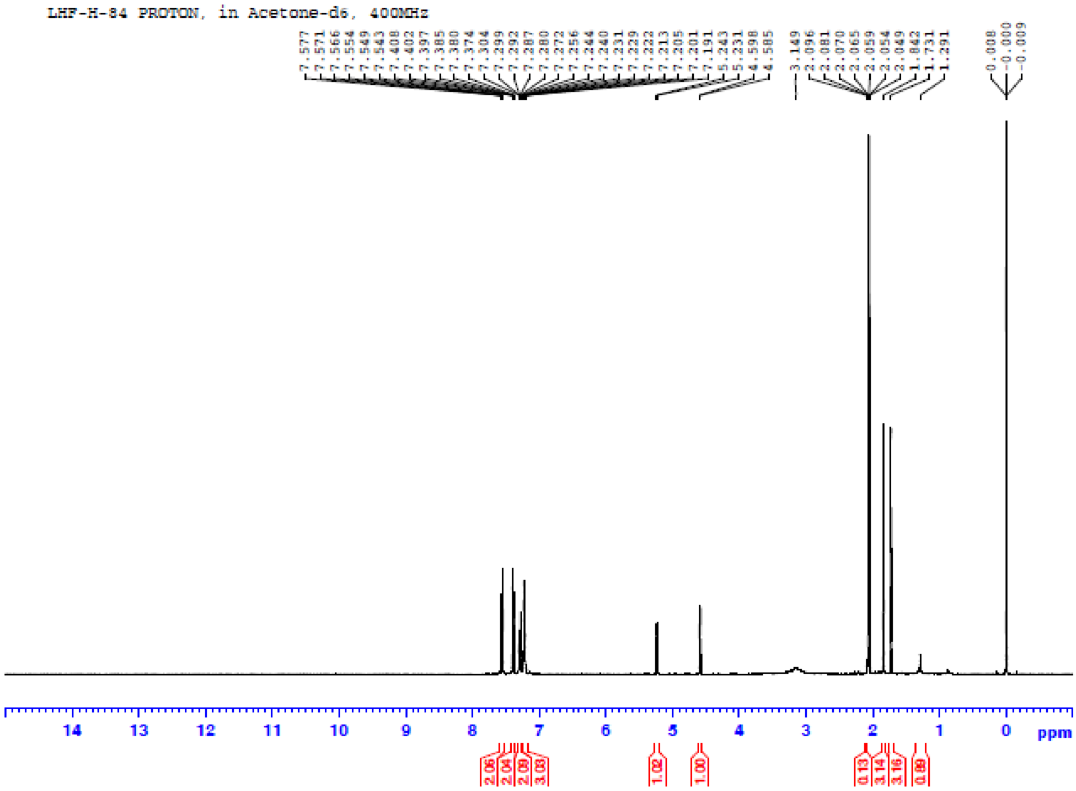

^{1}H-NMR of 12h

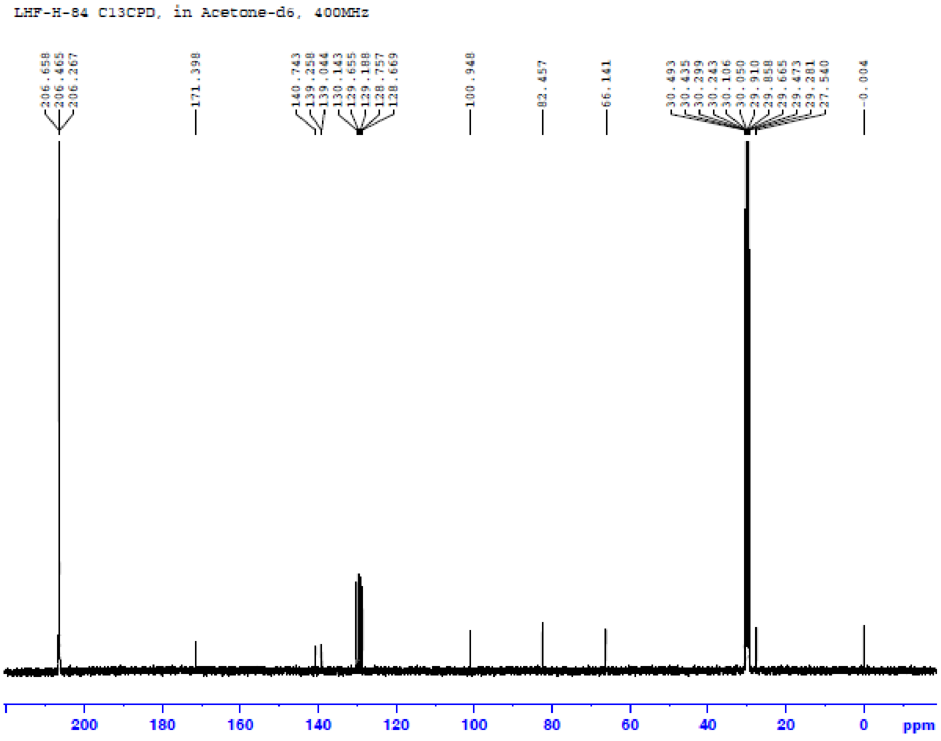

^{13}C-NMR of 12h

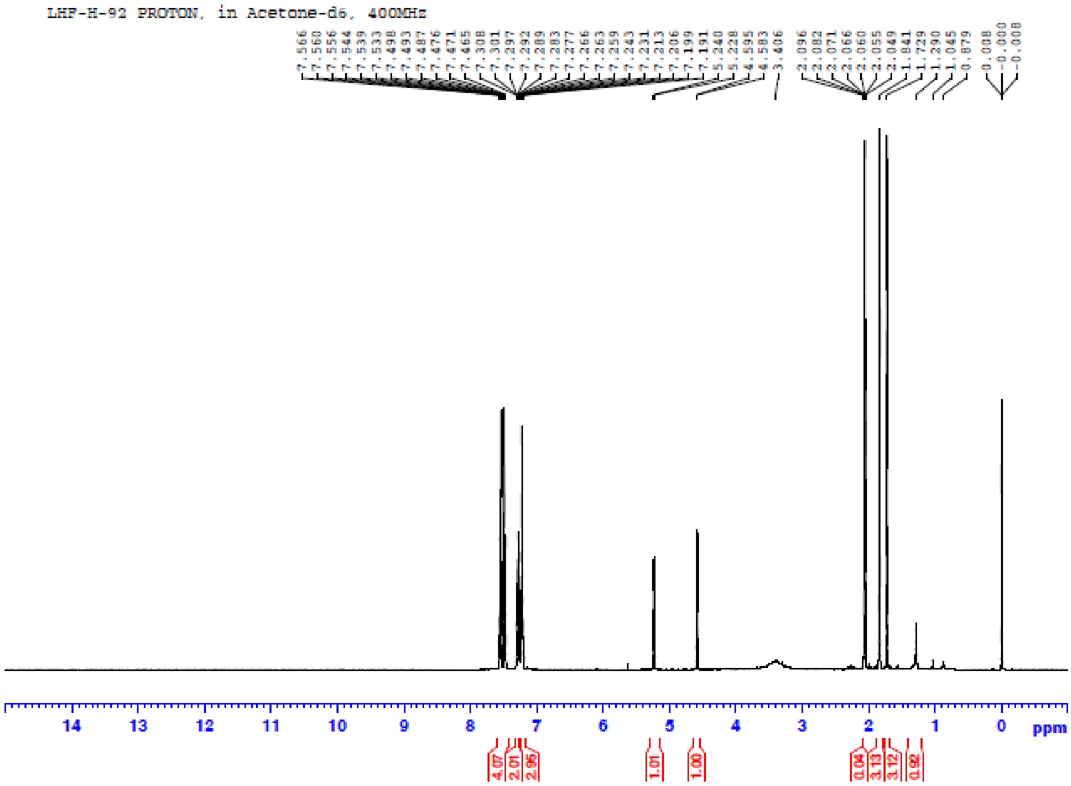

^{1}H-NMR of 12i

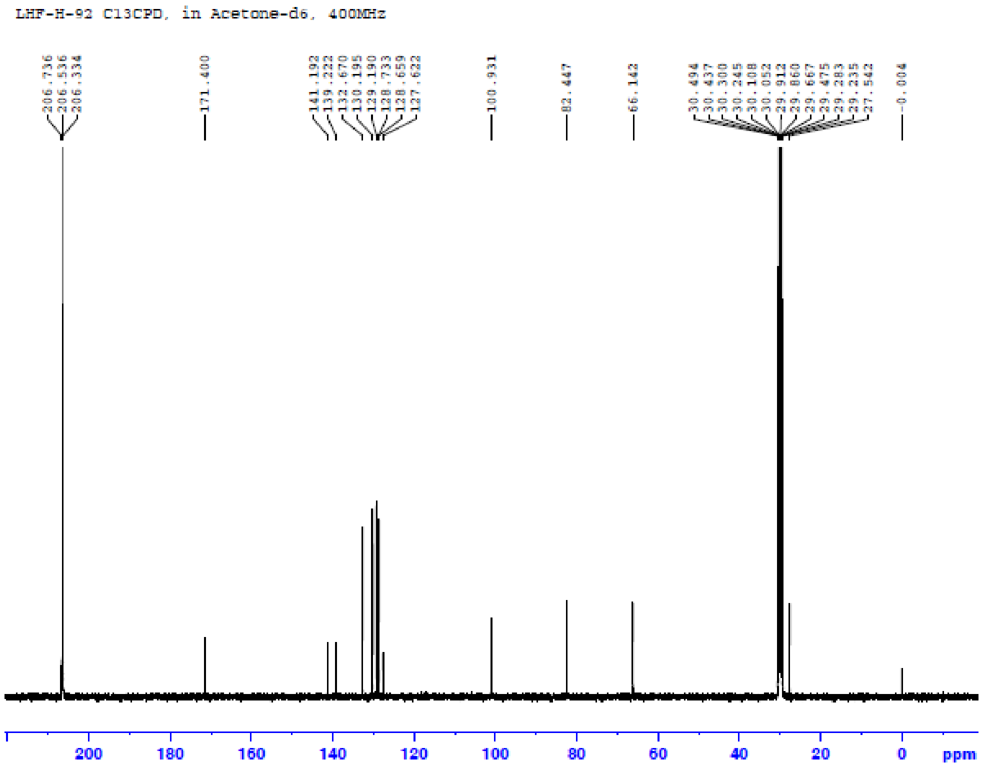

^{13}C-NMR of 12i

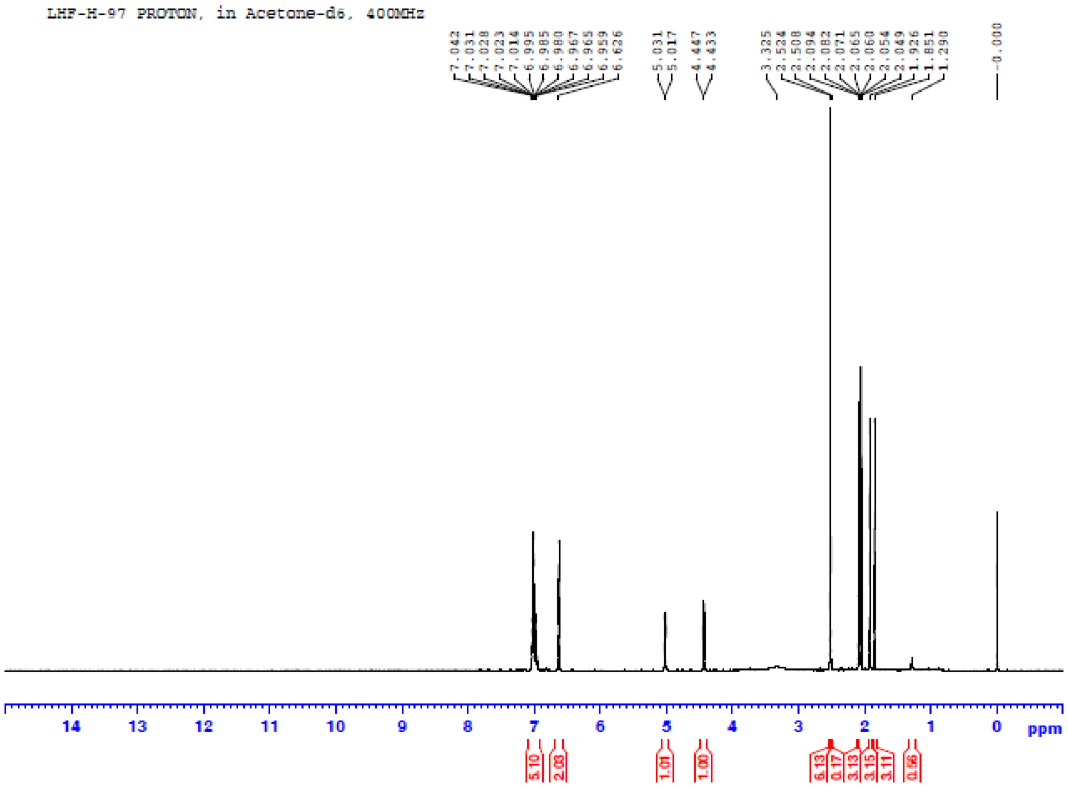

^{1}H-NMR of 12j

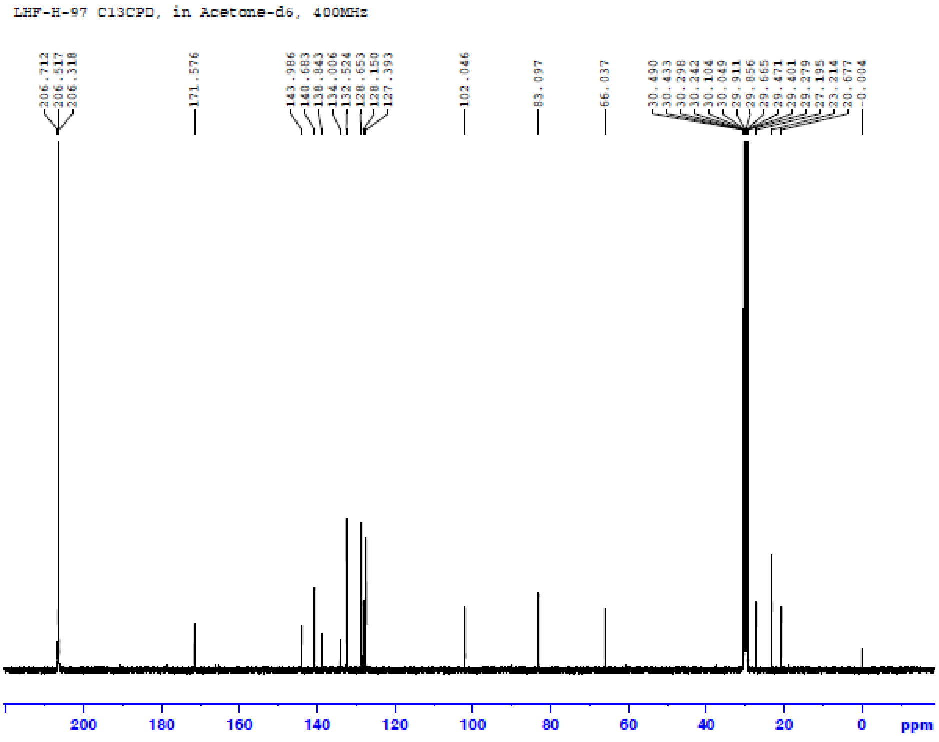

^{13}C-NMR of 12j

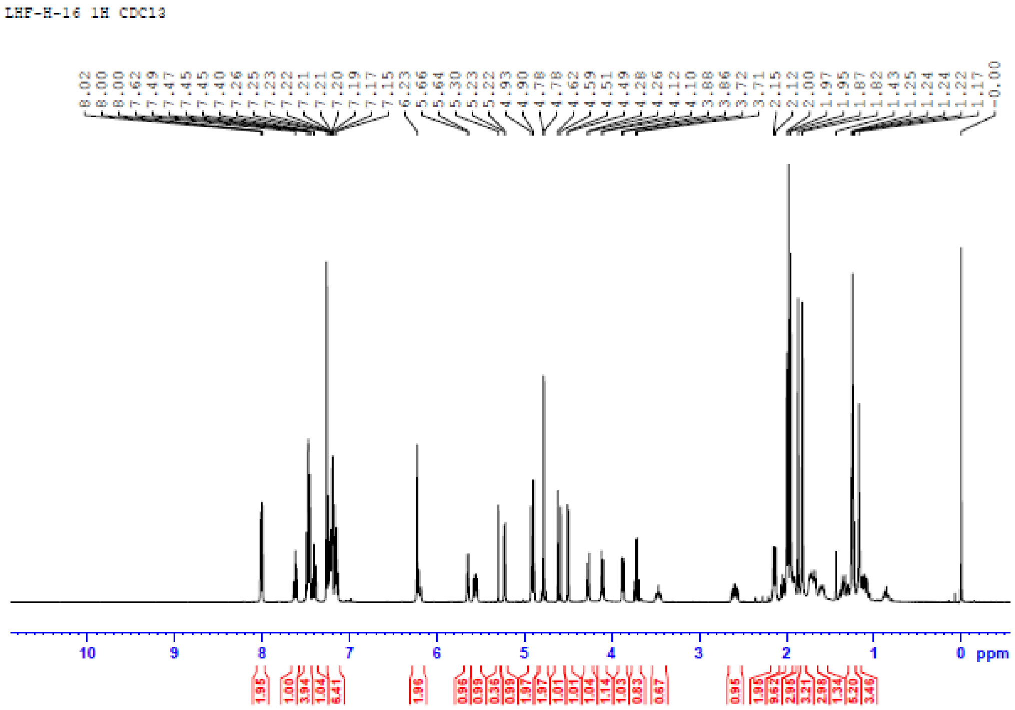

^{1}H-NMR of 13a

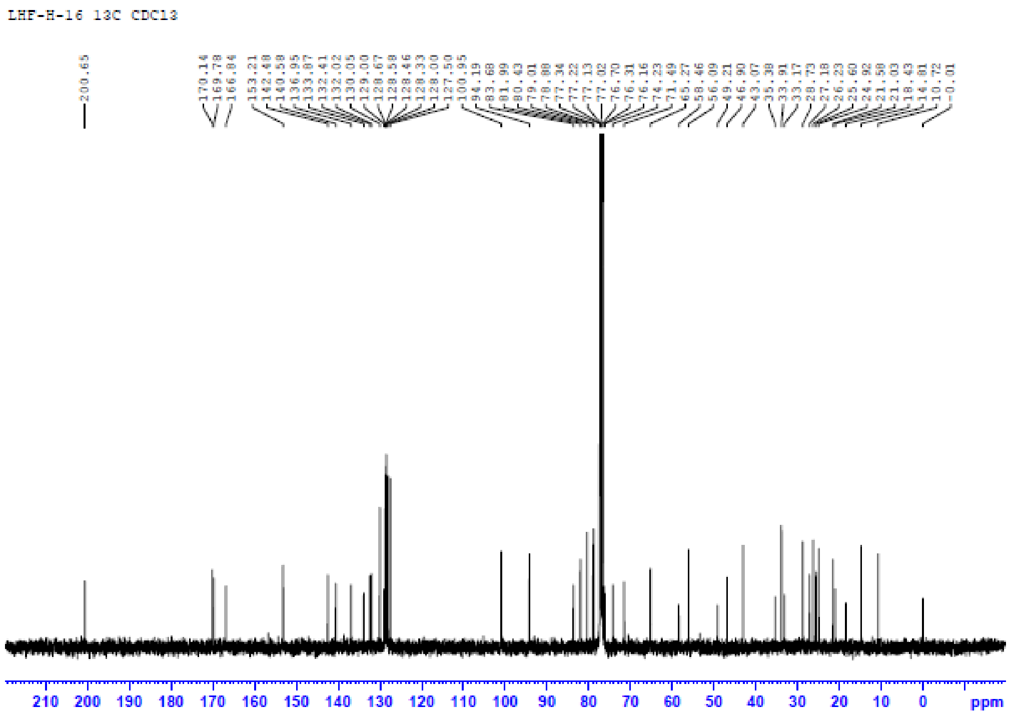

^{13}C-NMR of 13a

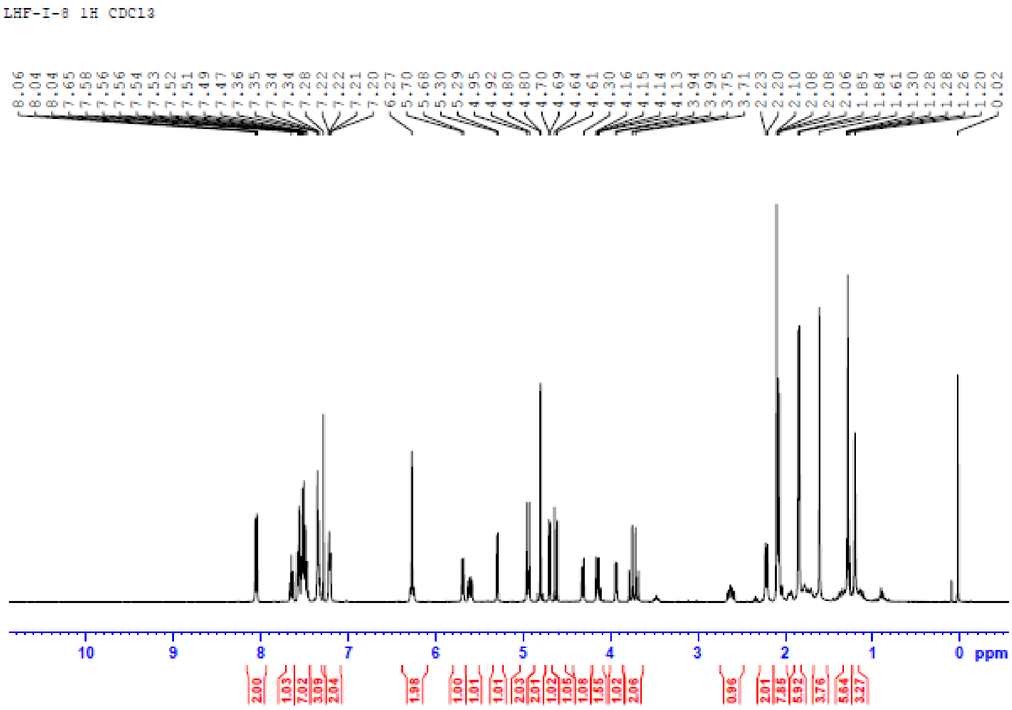

^{1}H-NMR of 13b

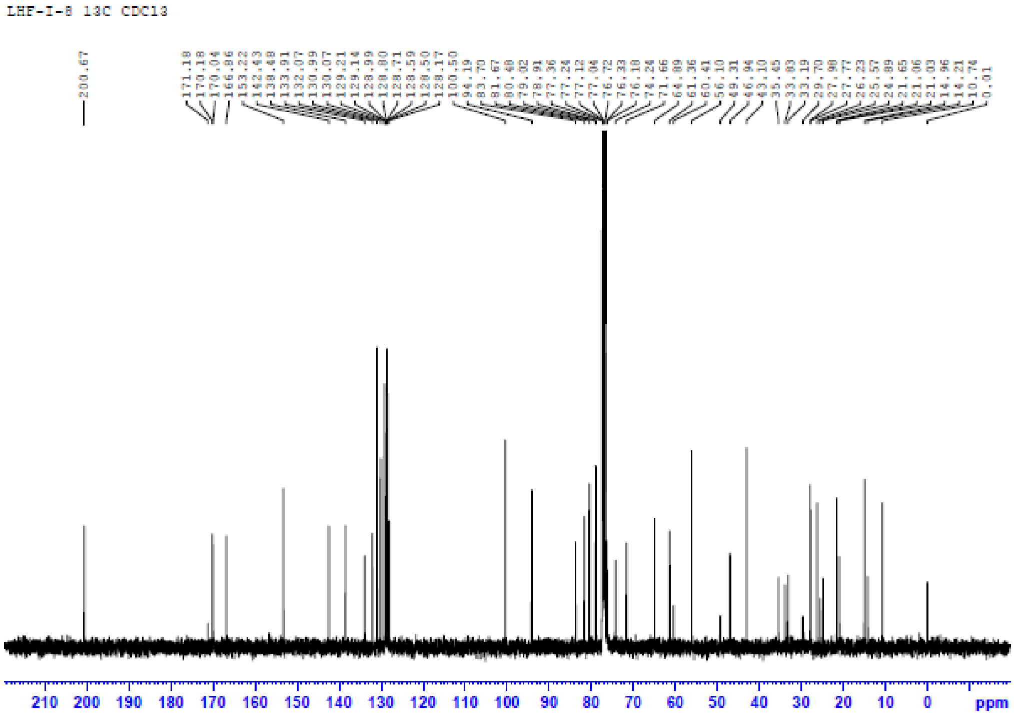

^{13}C-NMR of 13b

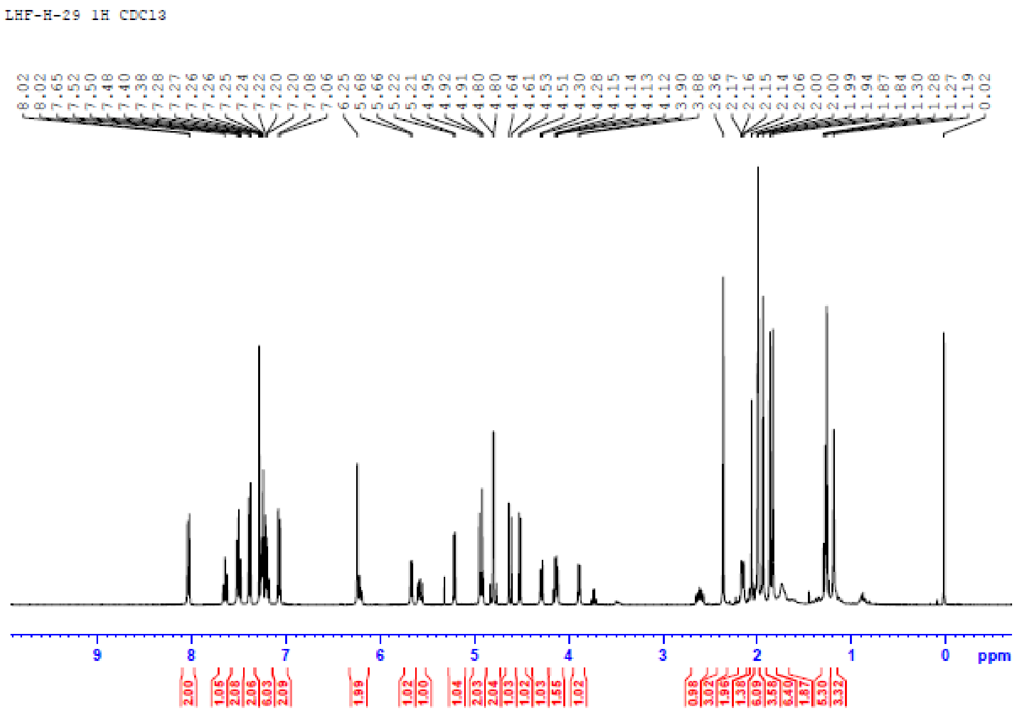

^{1}H-NMR of 13c

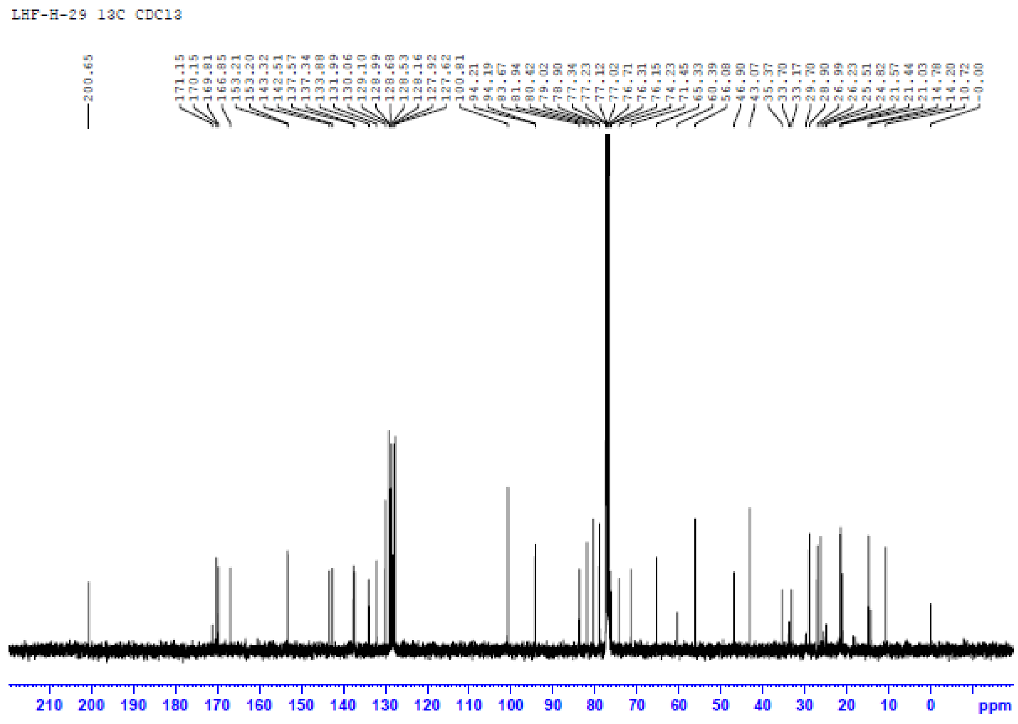

^{13}C-NMR of 13c

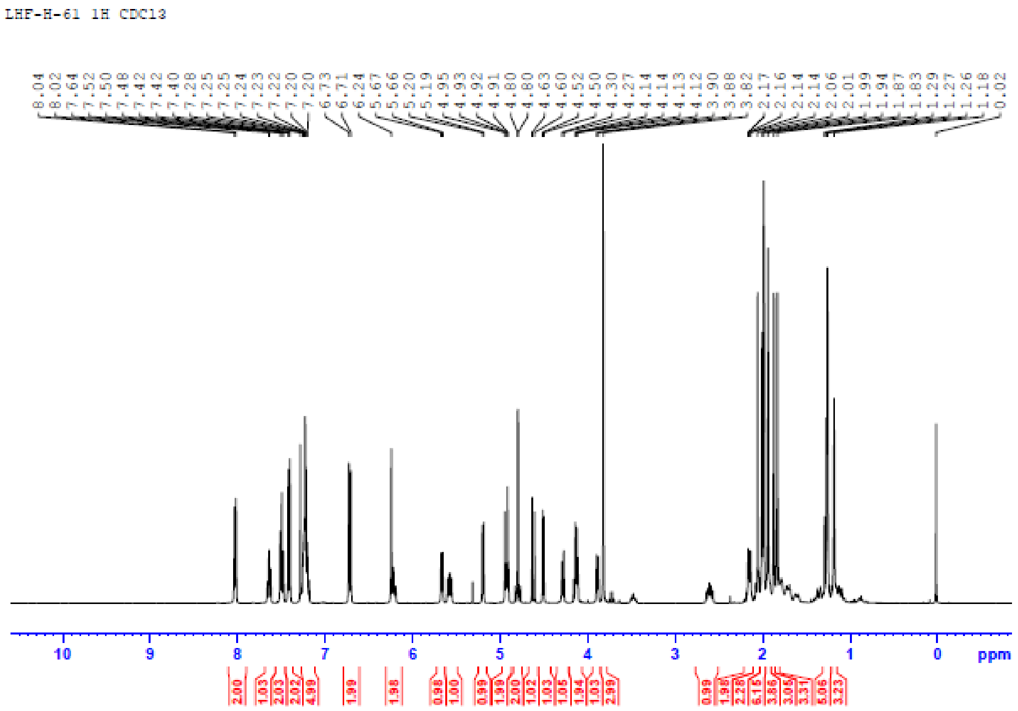

^{1}H-NMR of 13d

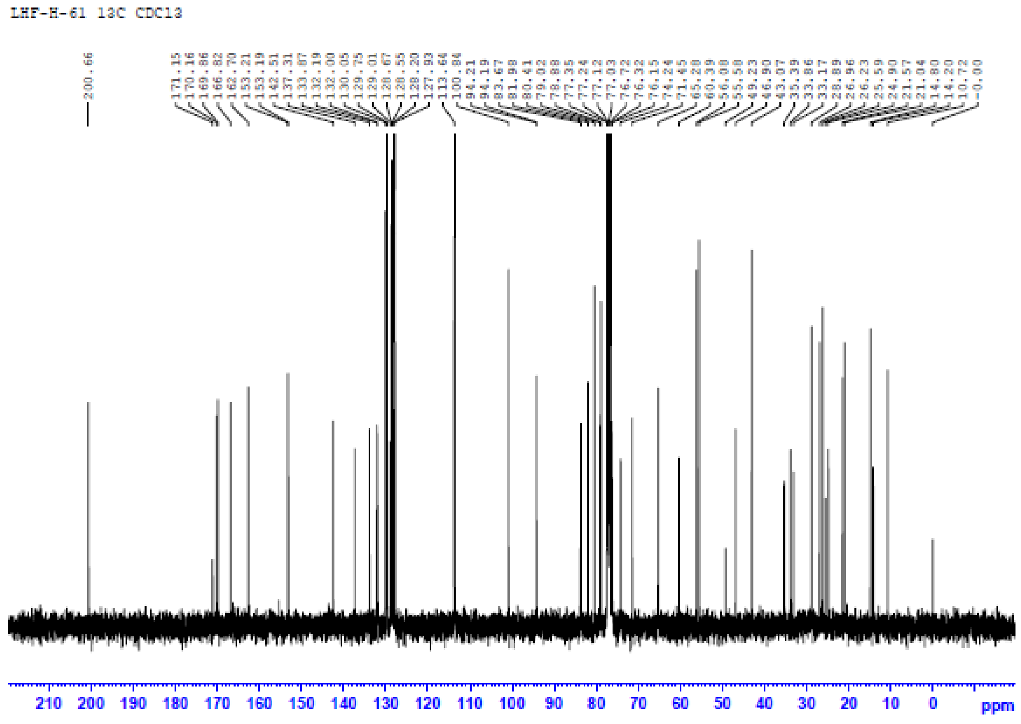

^{13}C-NMR of 13d

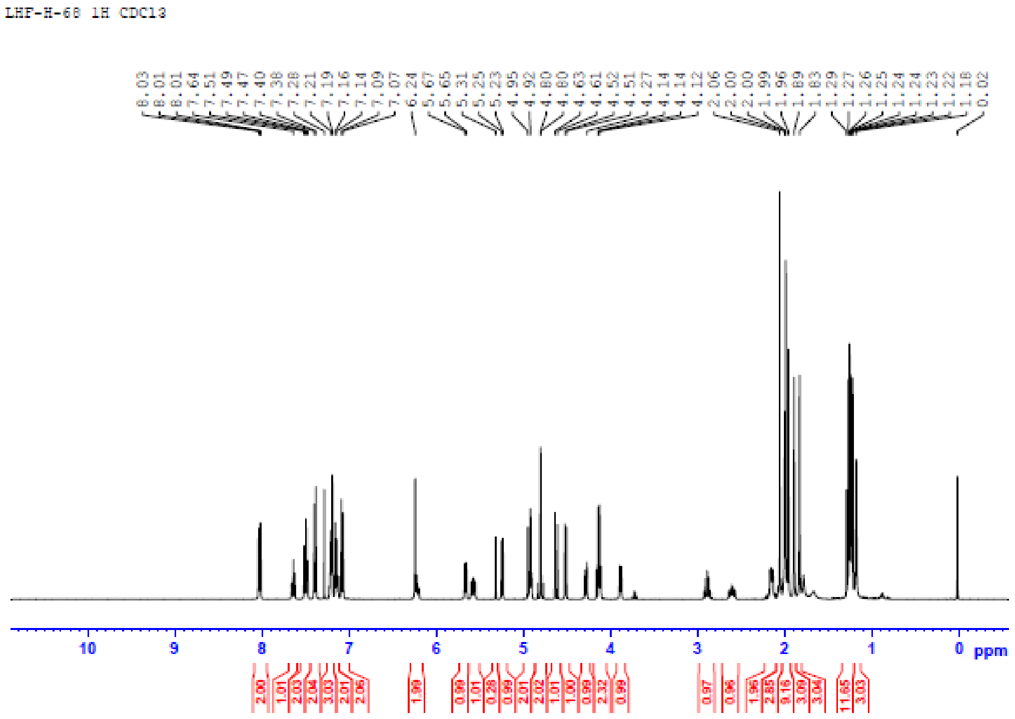

^{1}H-NMR of 13e

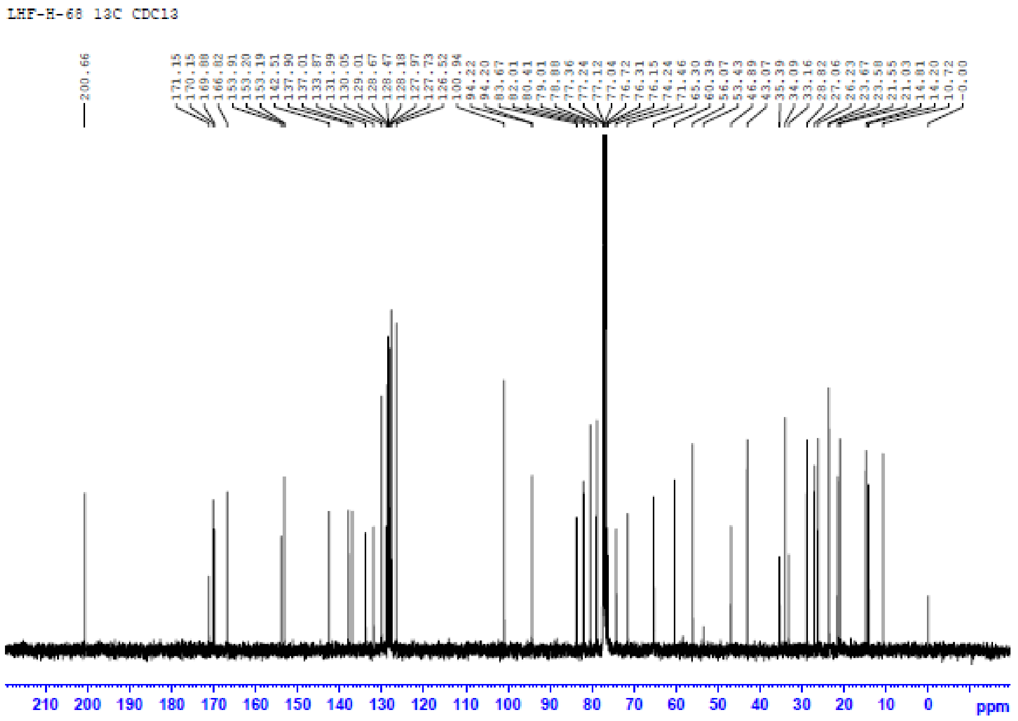

^{13}C-NMR of 13e

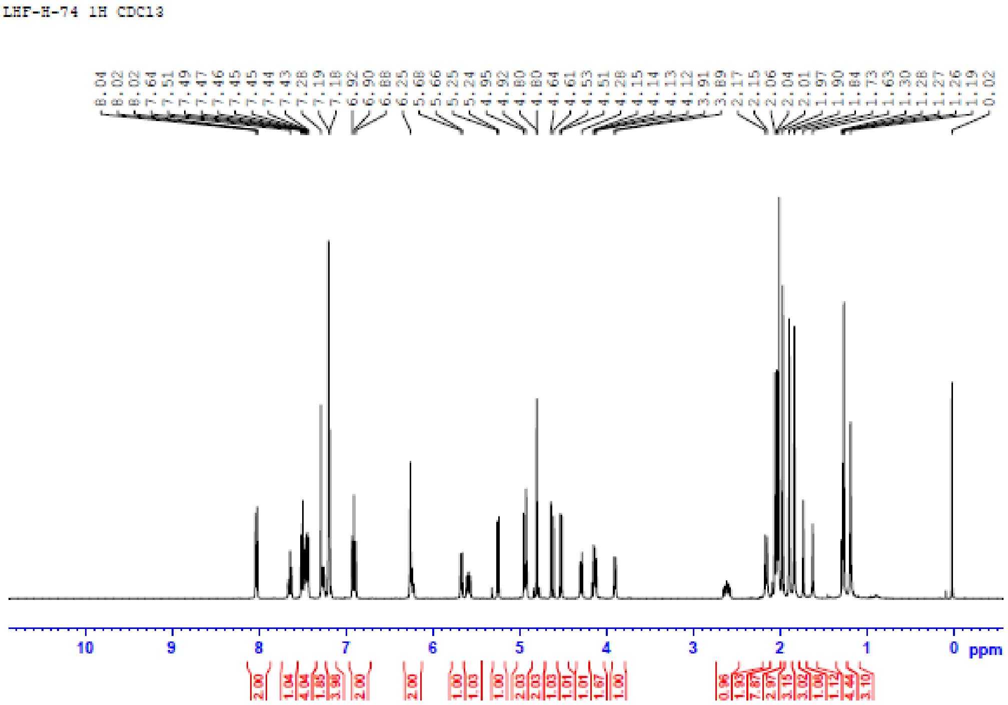

^{1}H-NMR of 13f

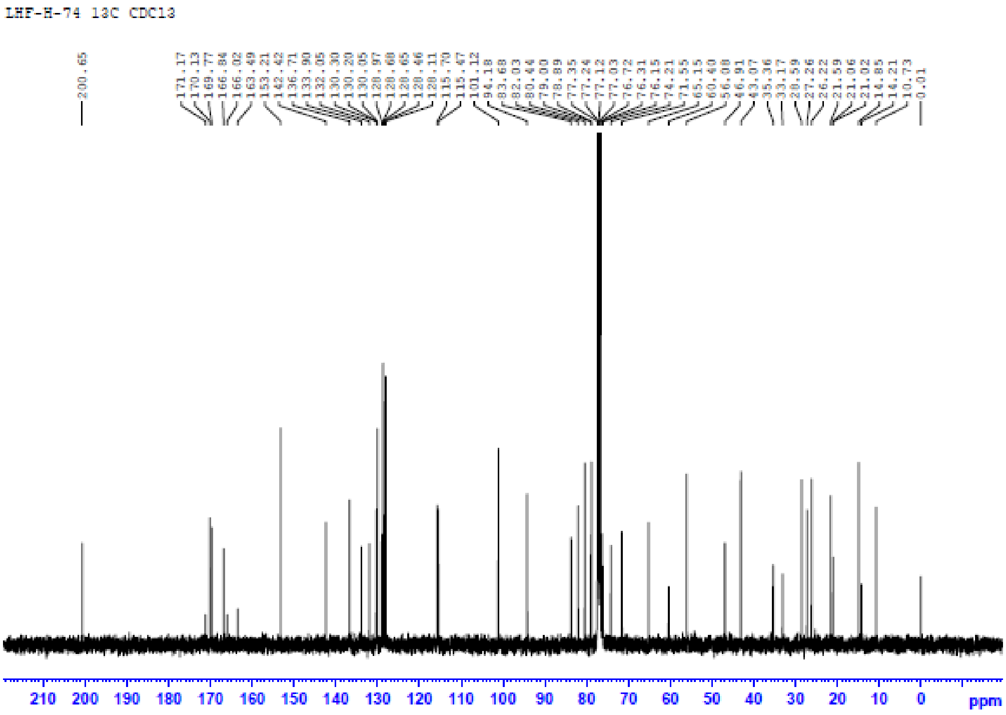

^{13}C-NMR of 13f

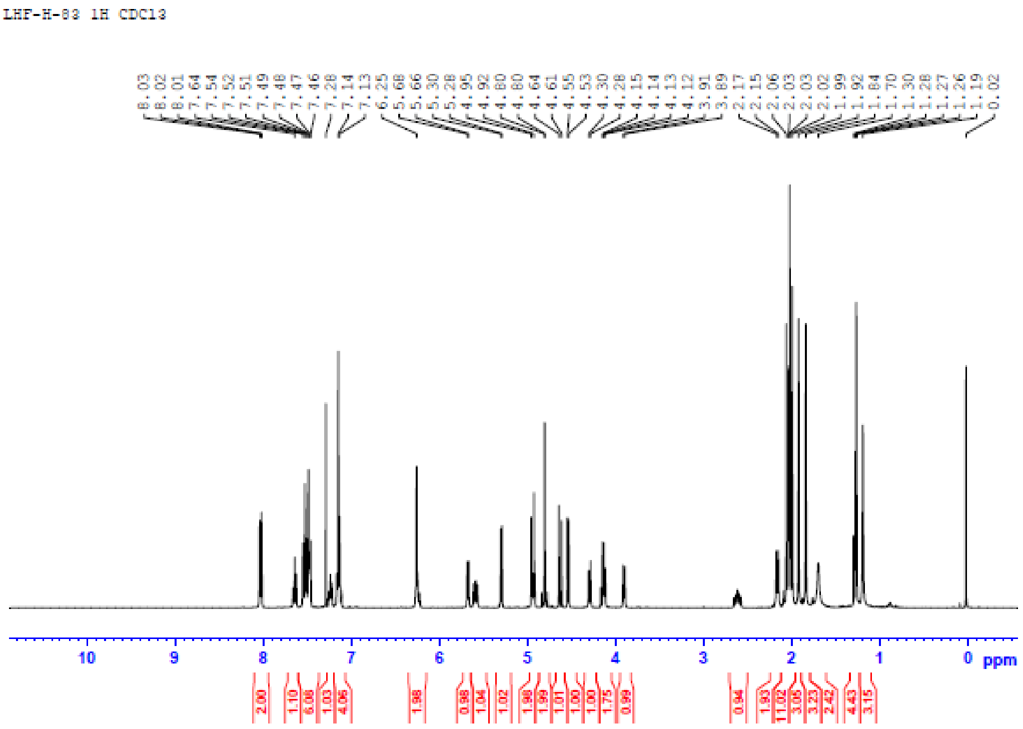

^{1}H-NMR of 13g

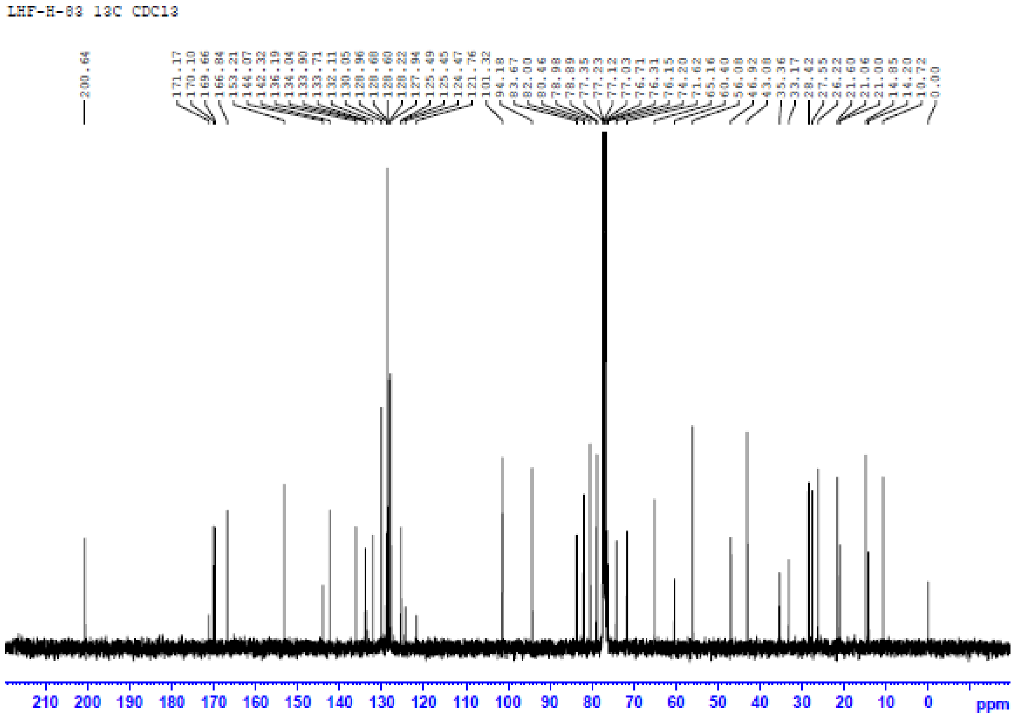

^{13}C-NMR of 13g

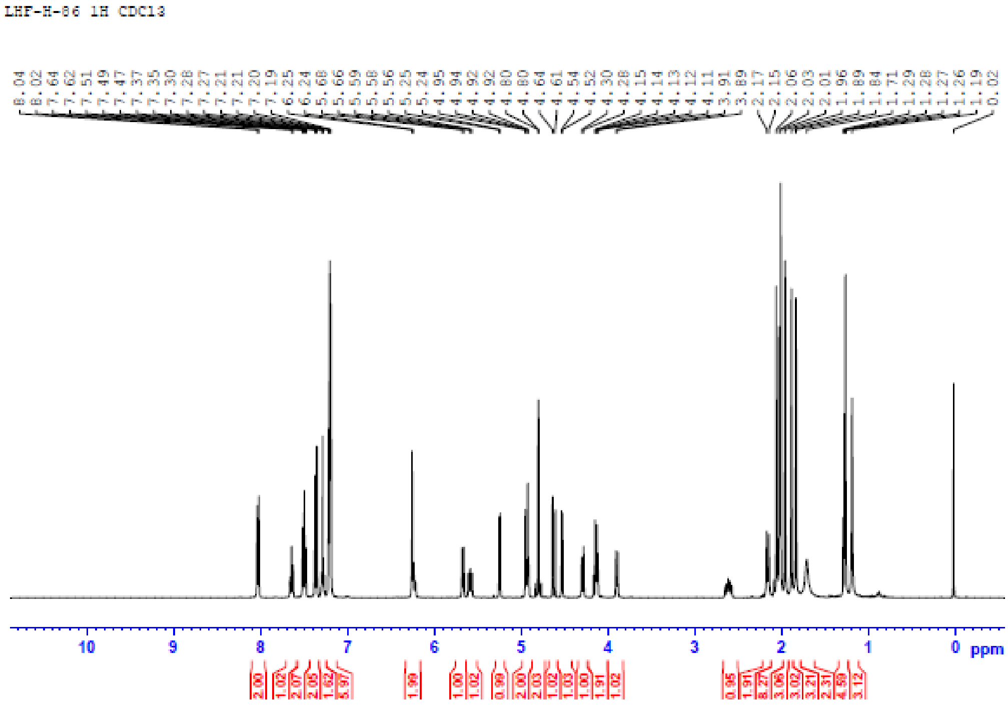

^{1}H-NMR of 13h

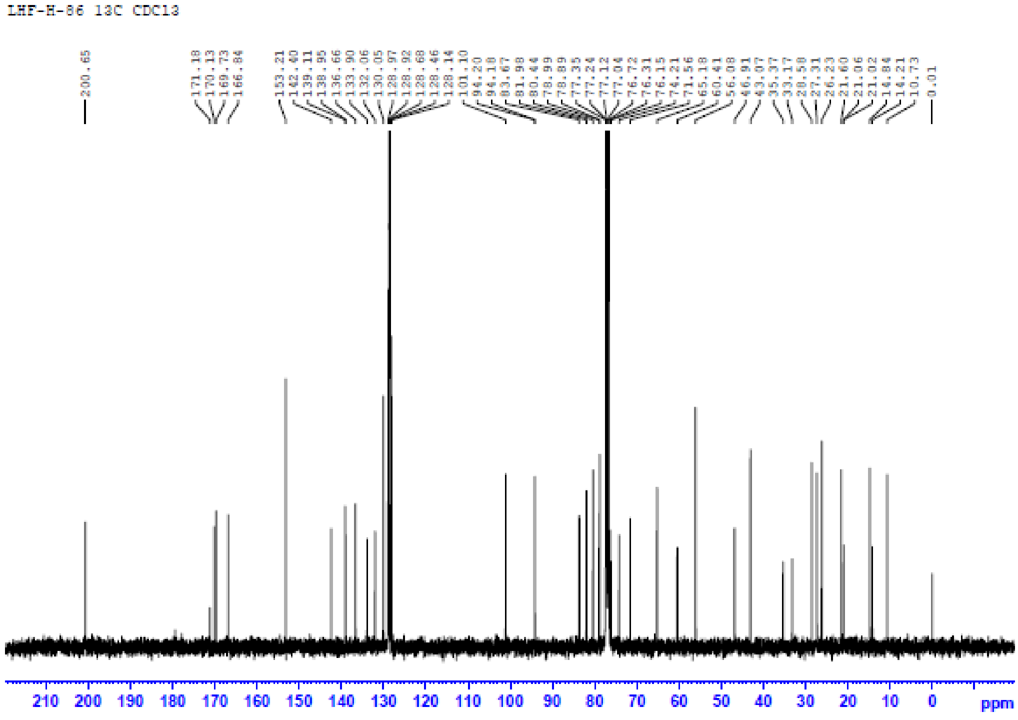

^{13}C-NMR of 13h

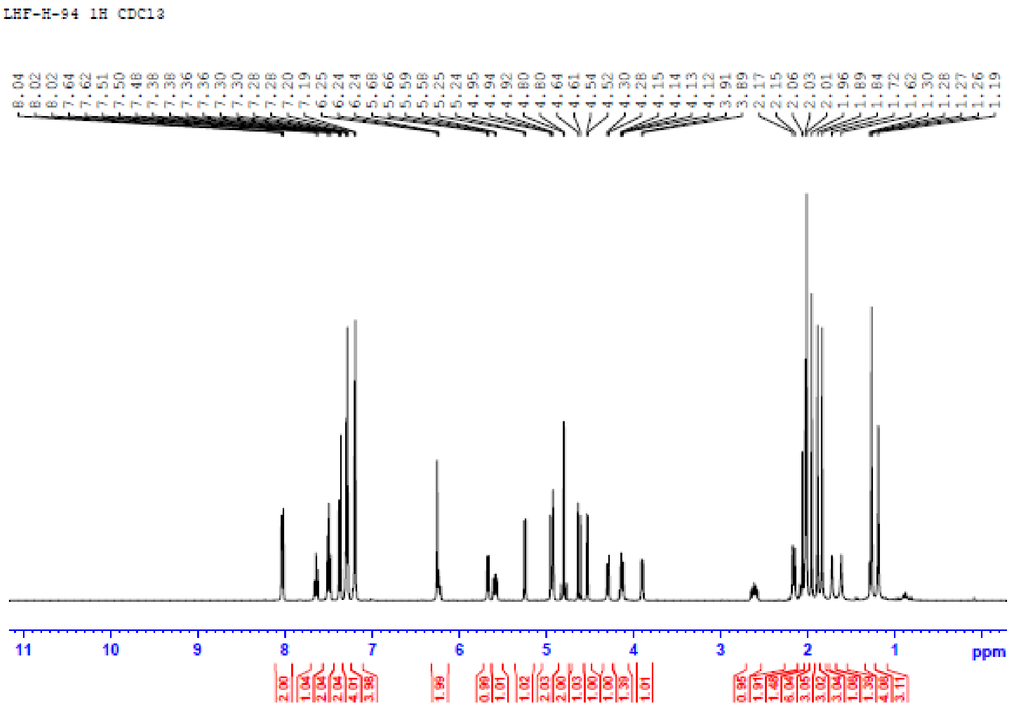

^{1}H-NMR of 13i

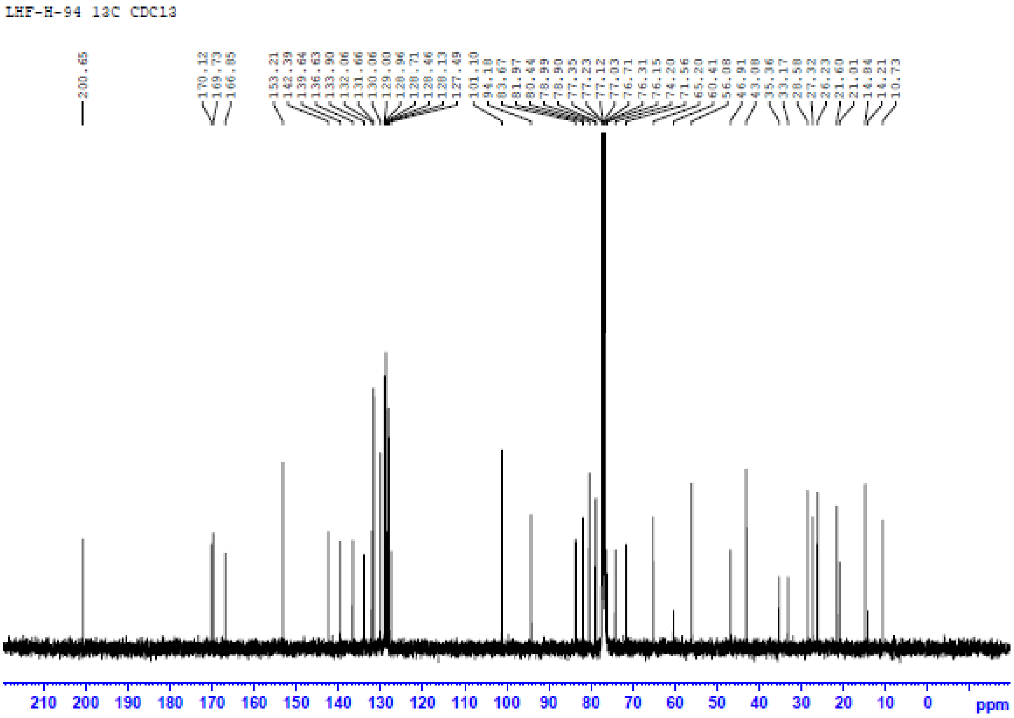

^{13}C-NMR of 13i

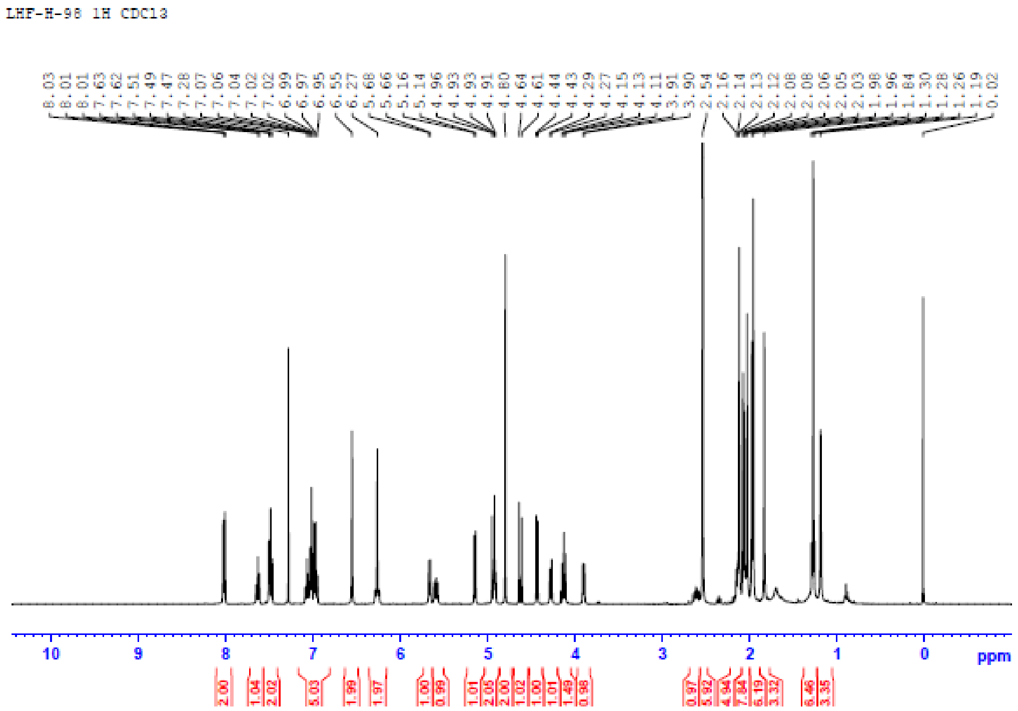

^{1}H-NMR of 13j

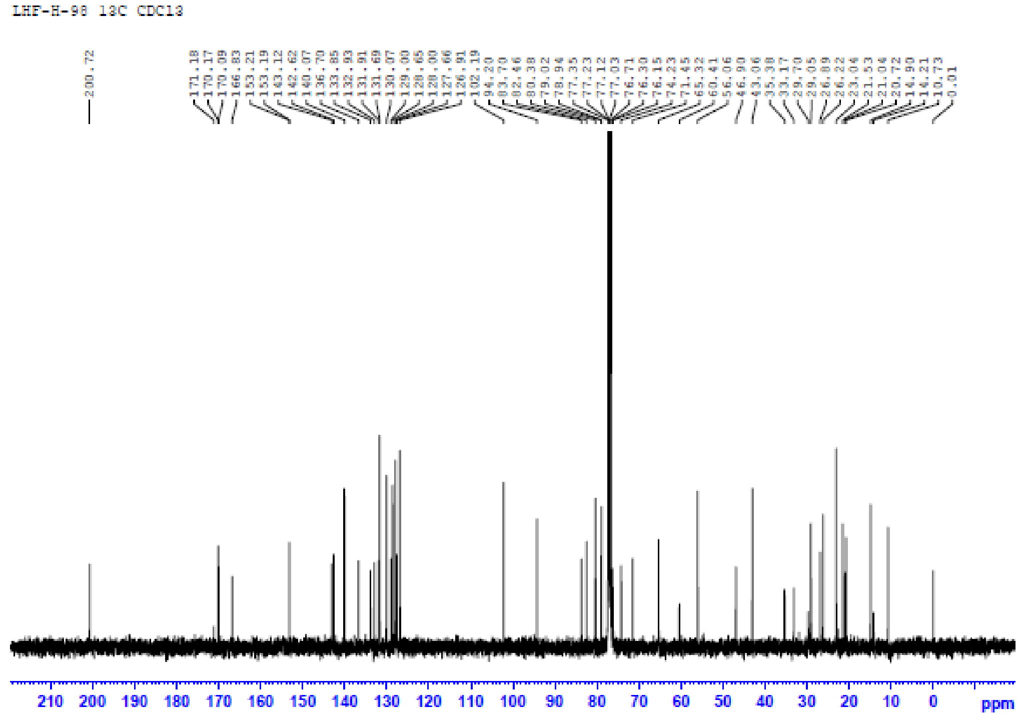

^{13}C-NMR of 13j

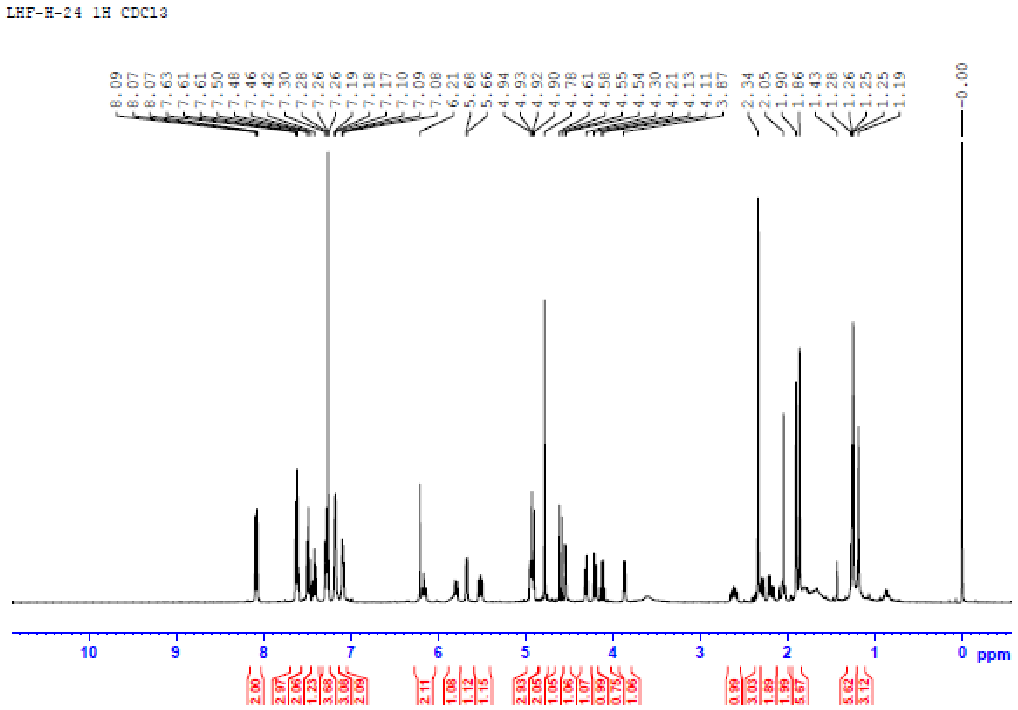

^{1}H-NMR of 5a

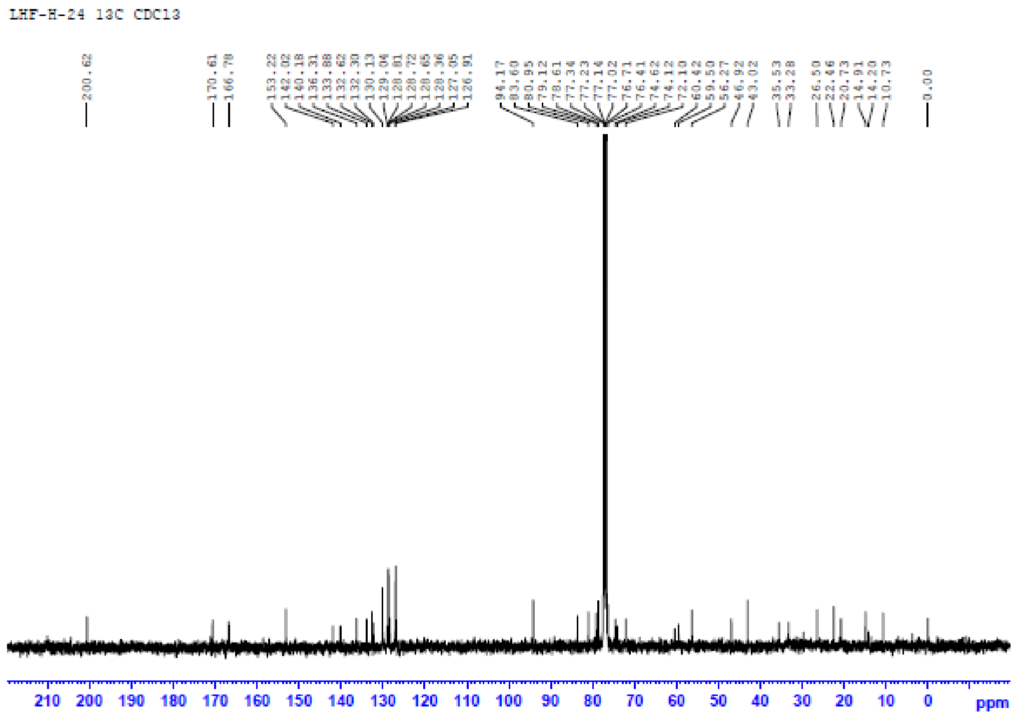

^{13}C-NMR of 5a

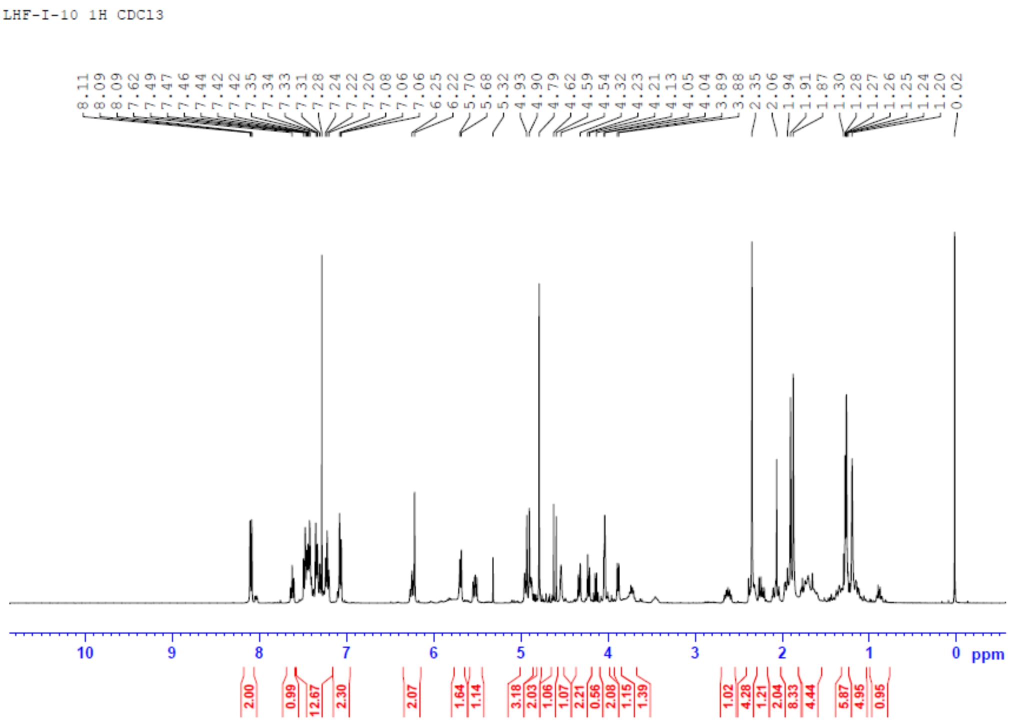

^{1}H-NMR of 5b

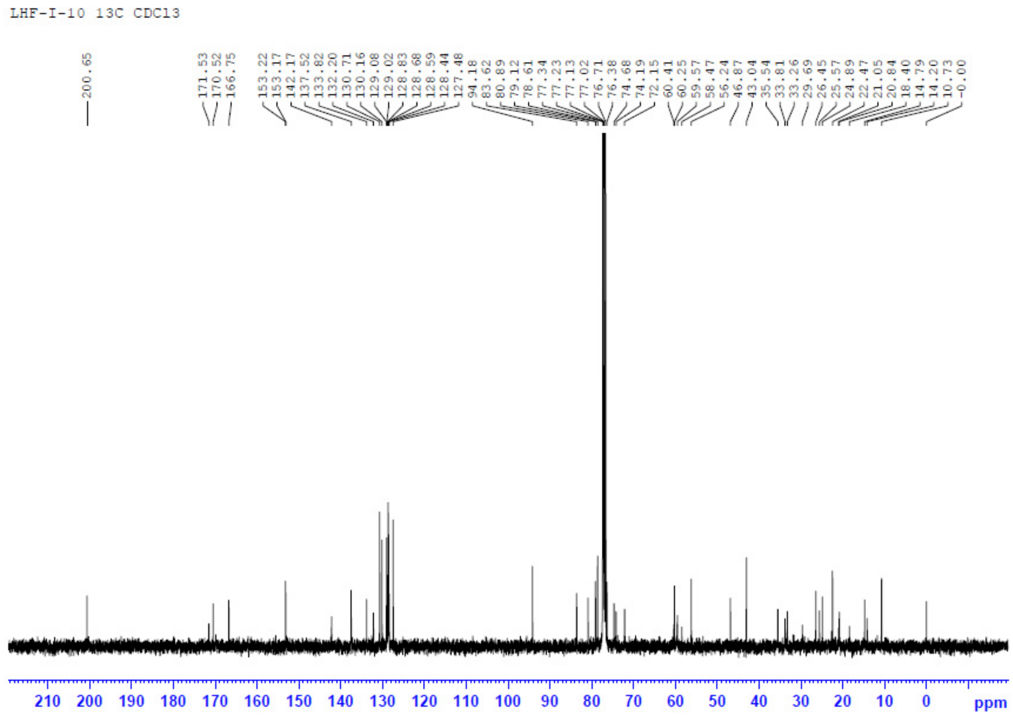

^{13}C-NMR of 5b

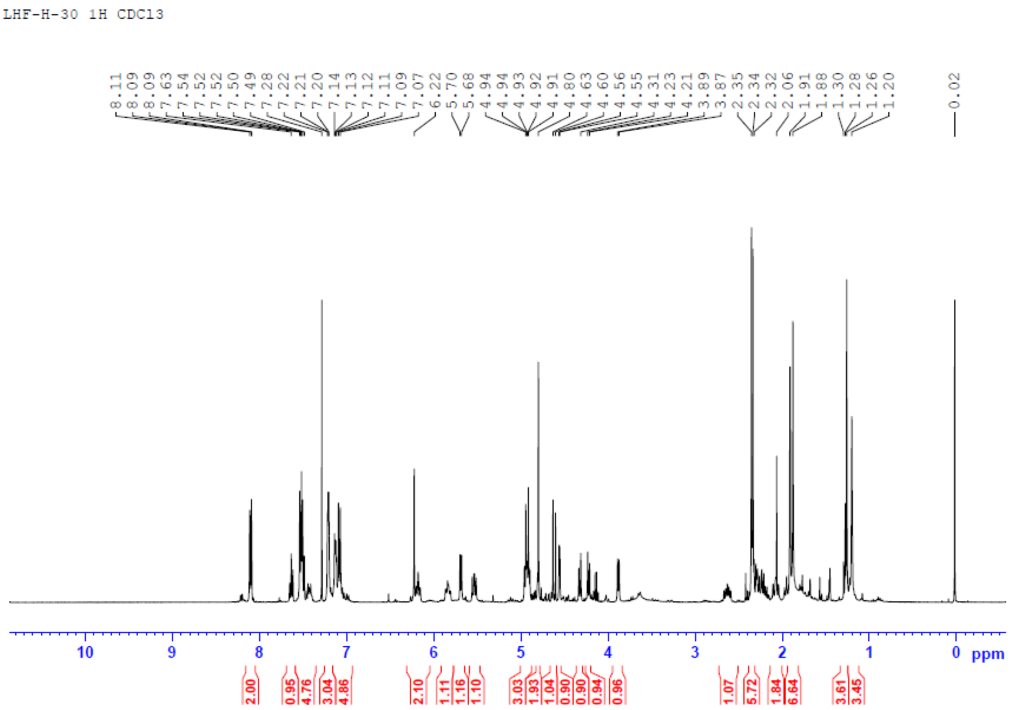

^{1}H-NMR of 5c

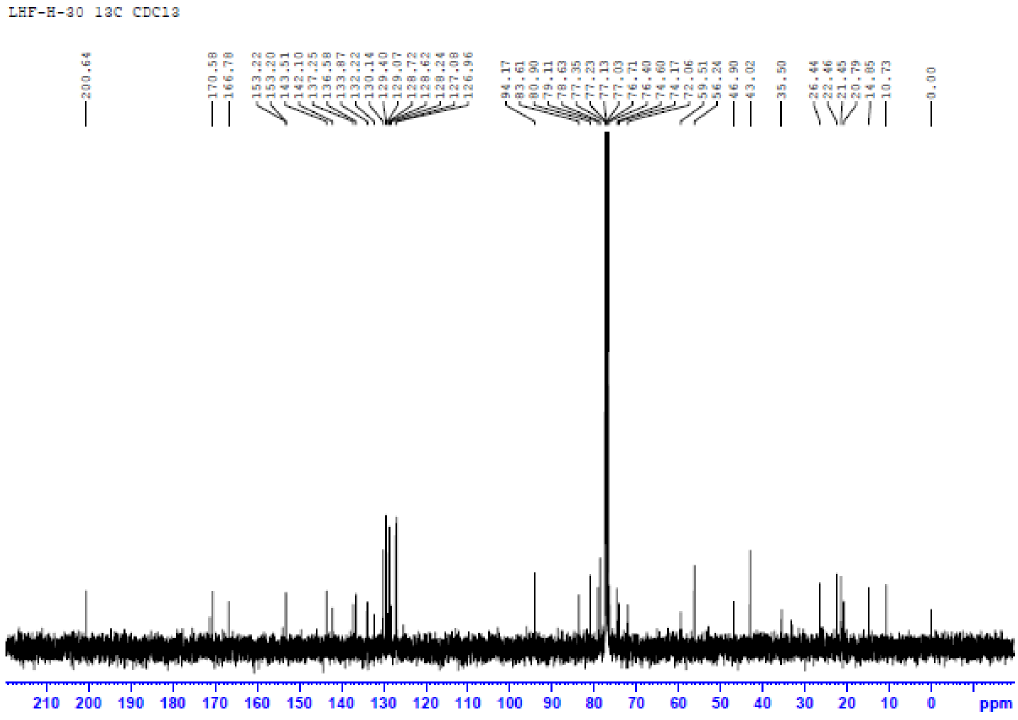

^{13}C-NMR of 5c

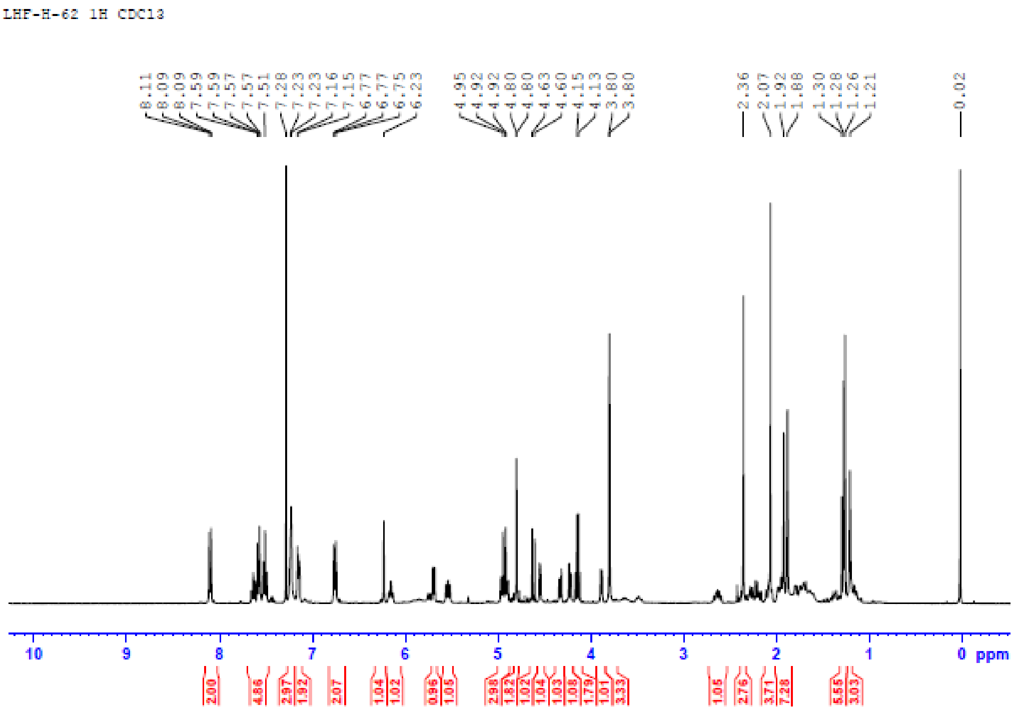

^{1}H-NMR of 5d

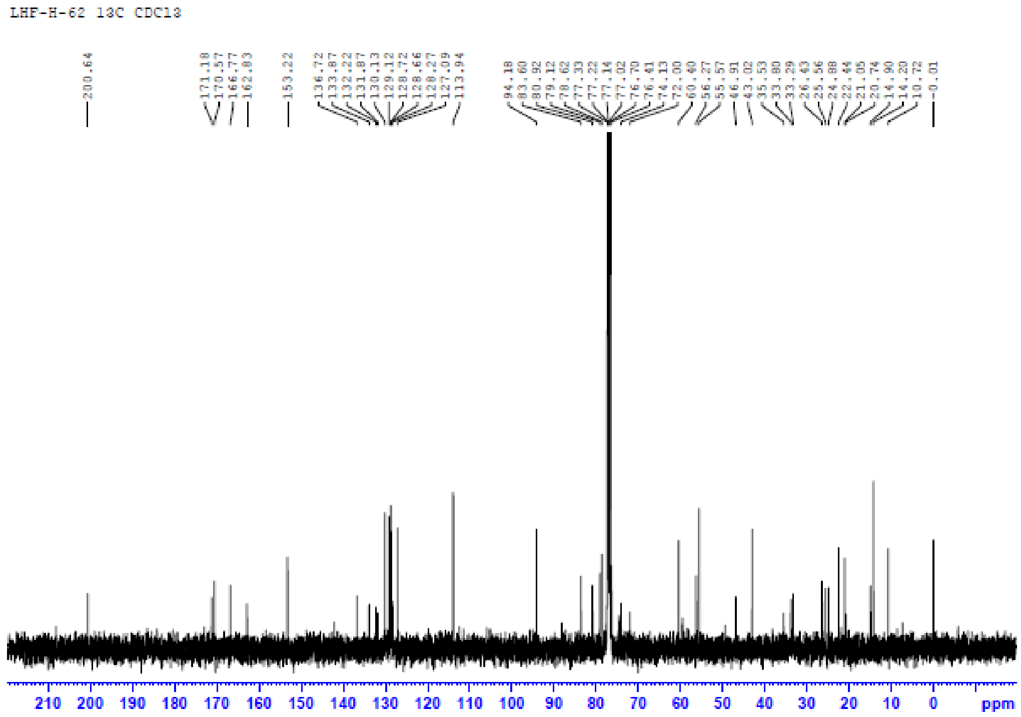

^{13}C-NMR of 5d

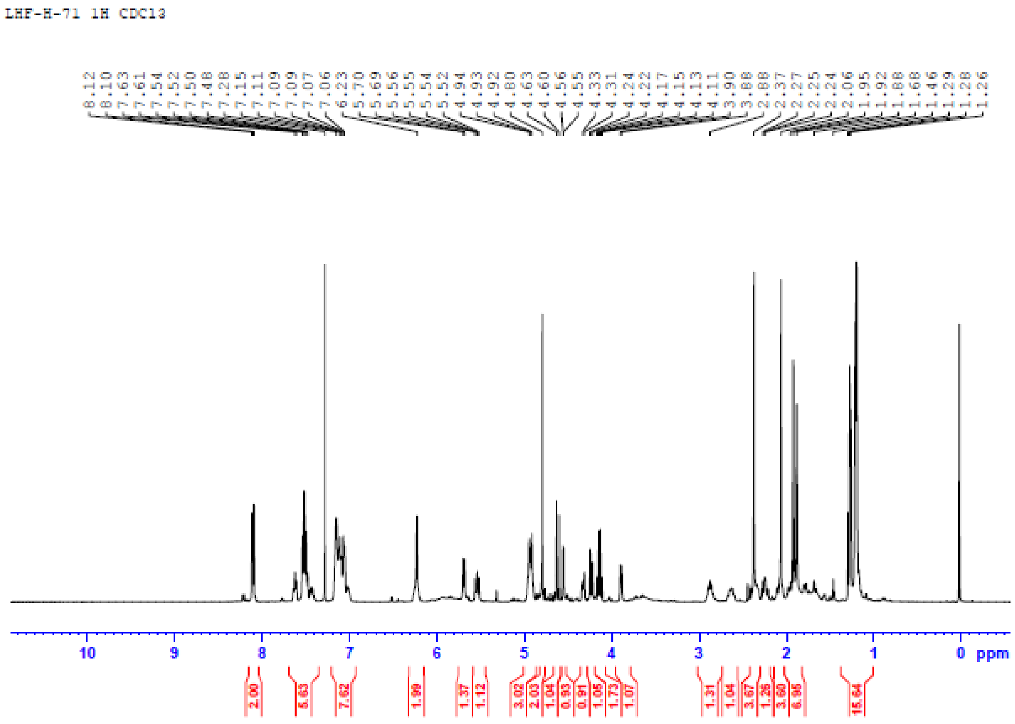

^{1}H-NMR of 5e

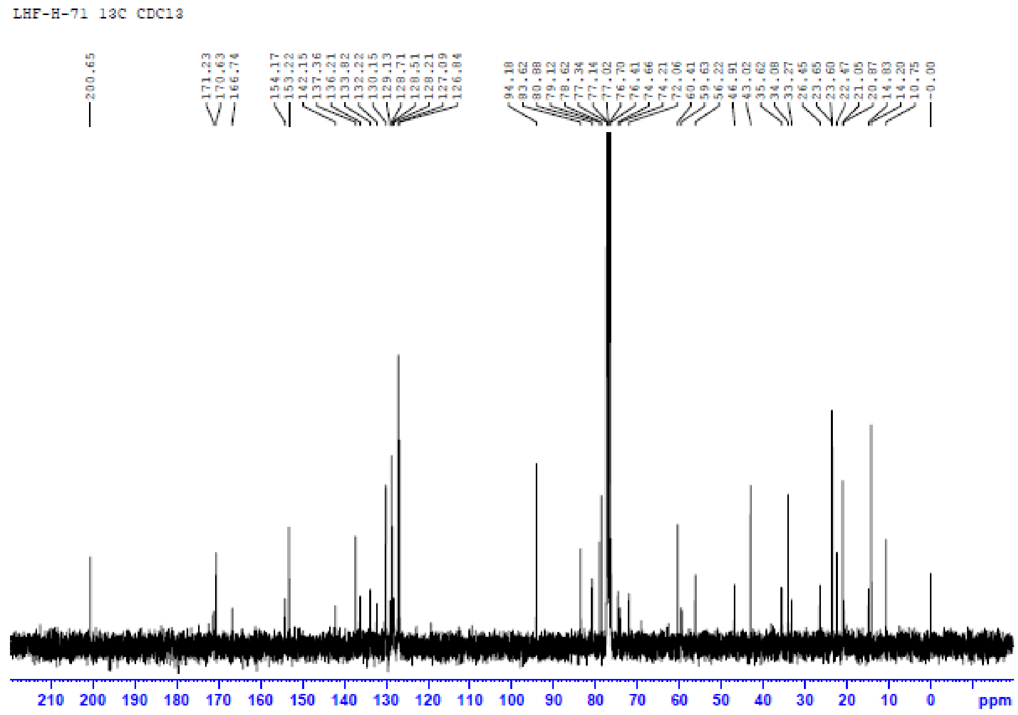

^{13}C-NMR of 5e

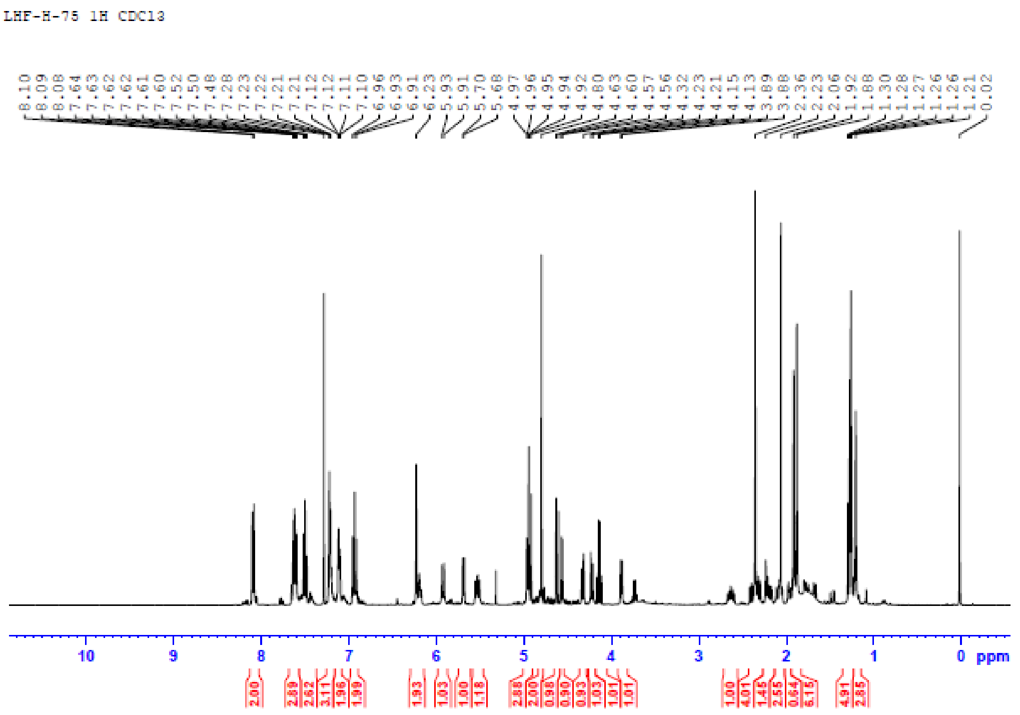

^{1}H-NMR of 5f

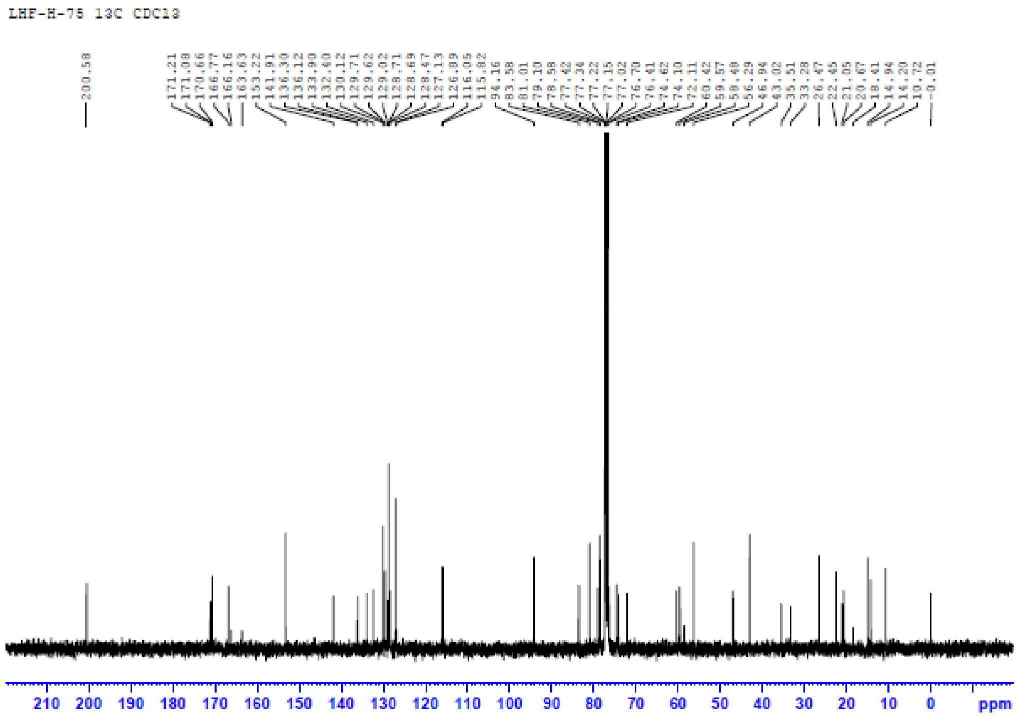

^{13}C-NMR of 5f

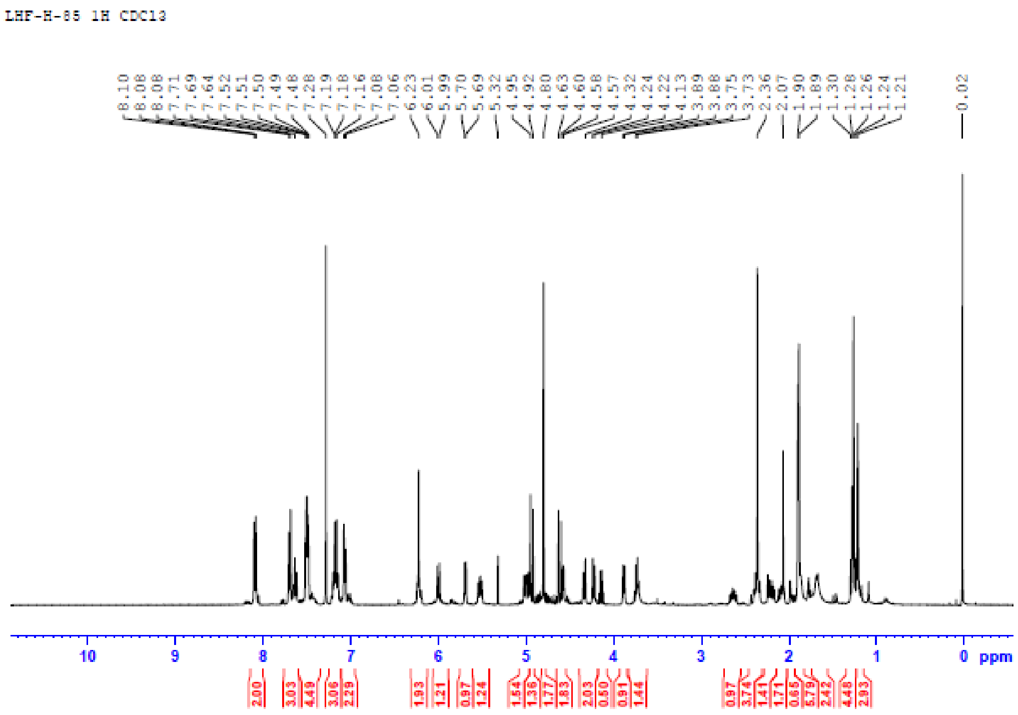

^{1}H-NMR of 5g

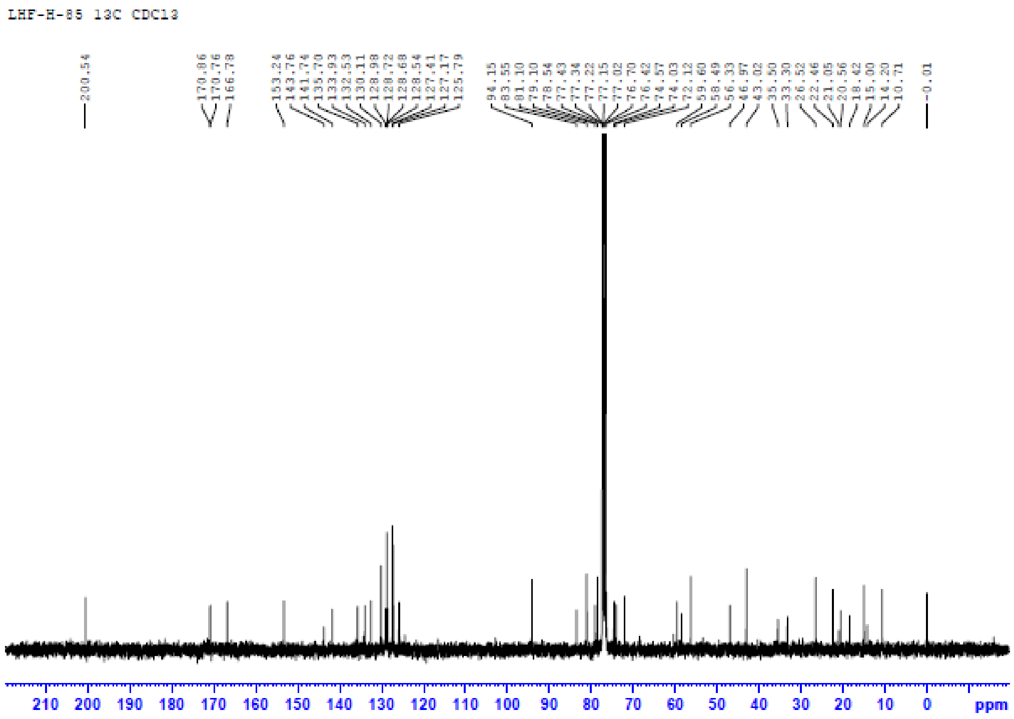

^{13}C-NMR of 5g

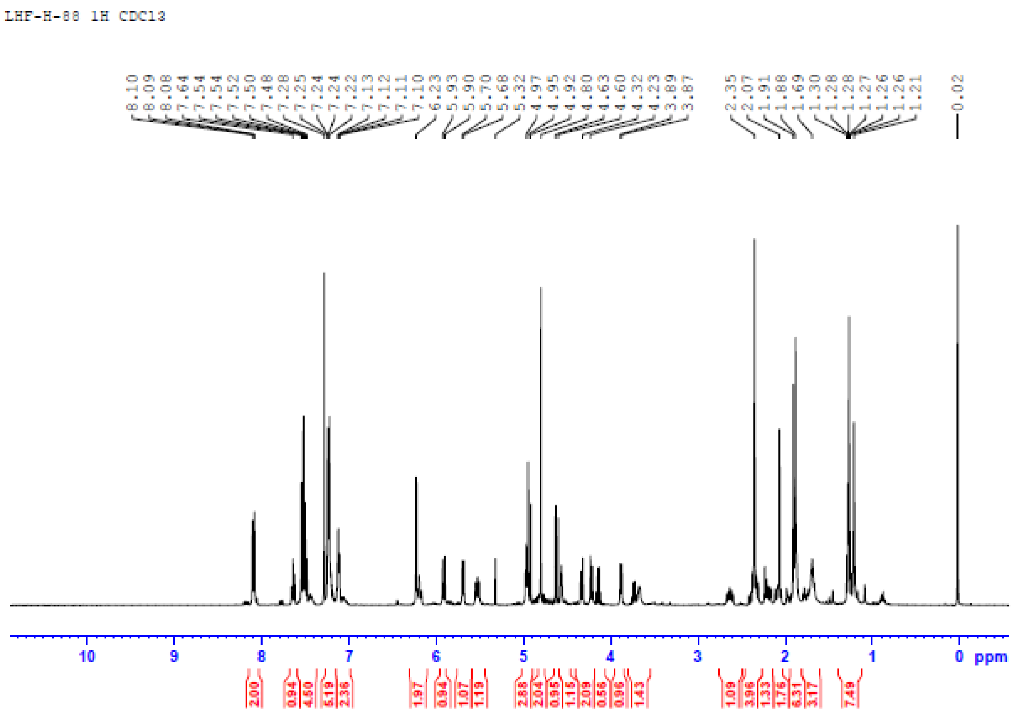

^{1}H-NMR of 5h

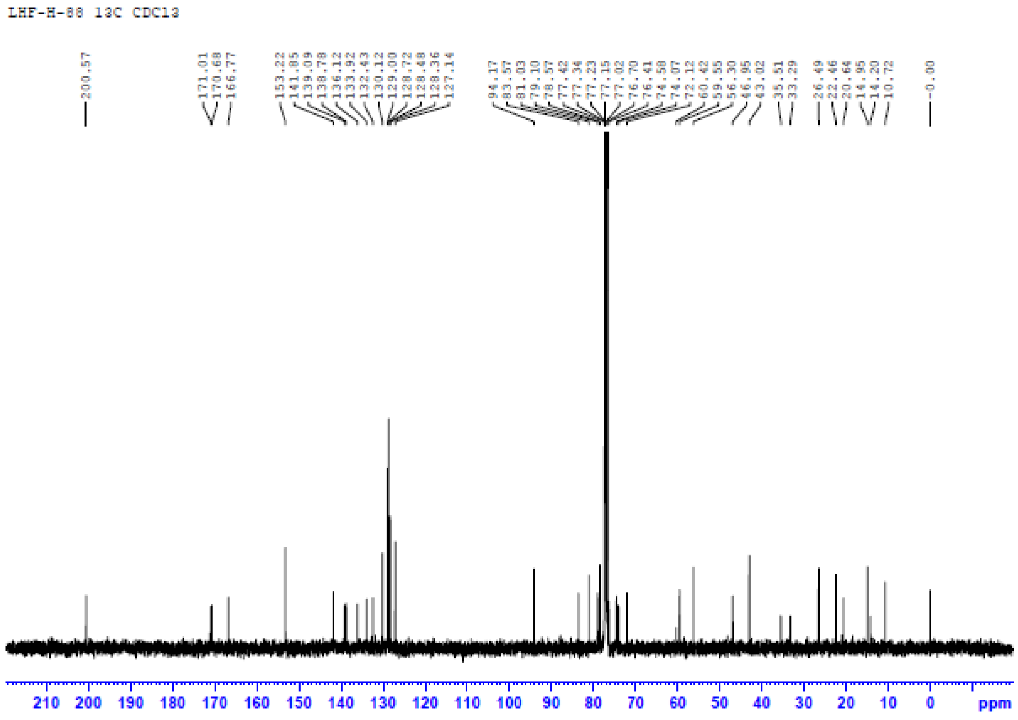

^{13}C-NMR of 5h

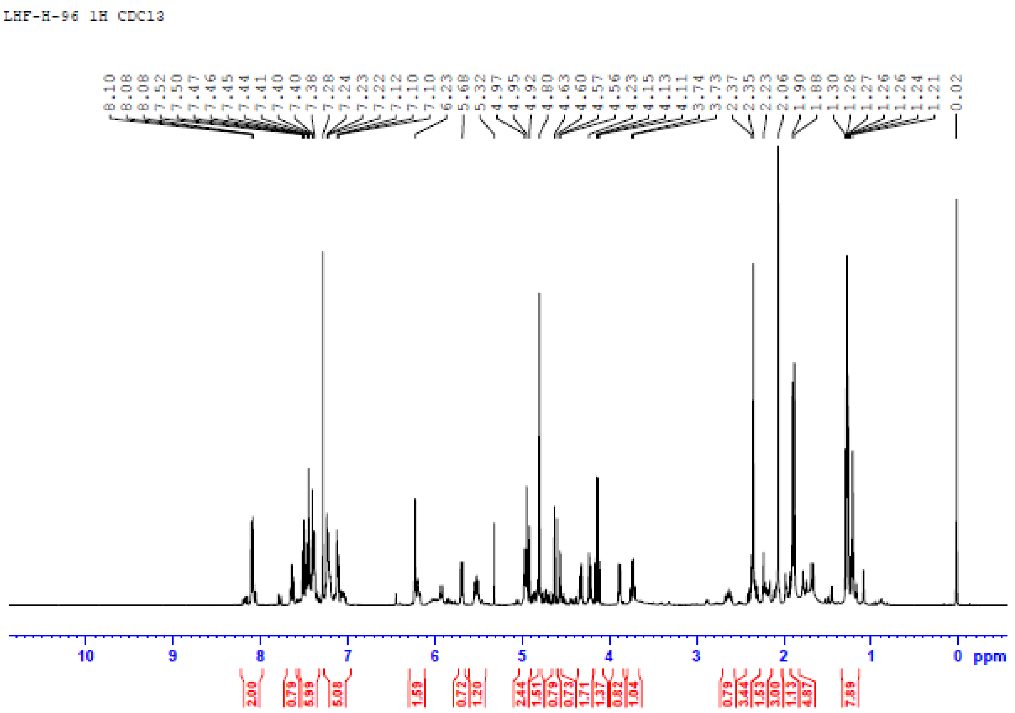

^{1}H-NMR of 5i

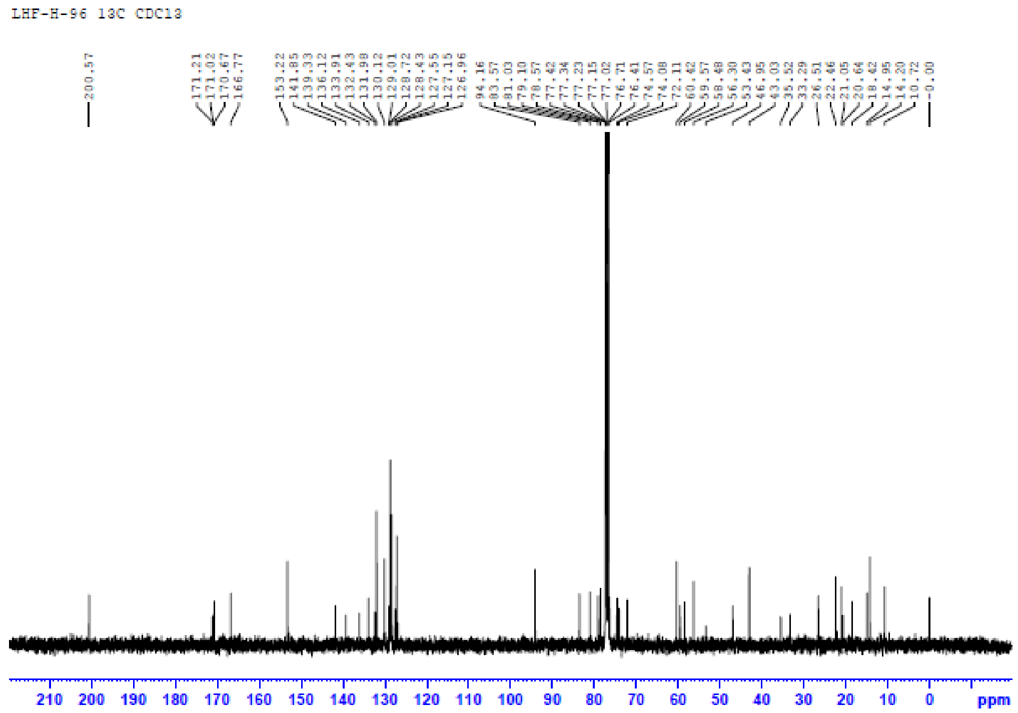

^{13}C-NMR of 5i

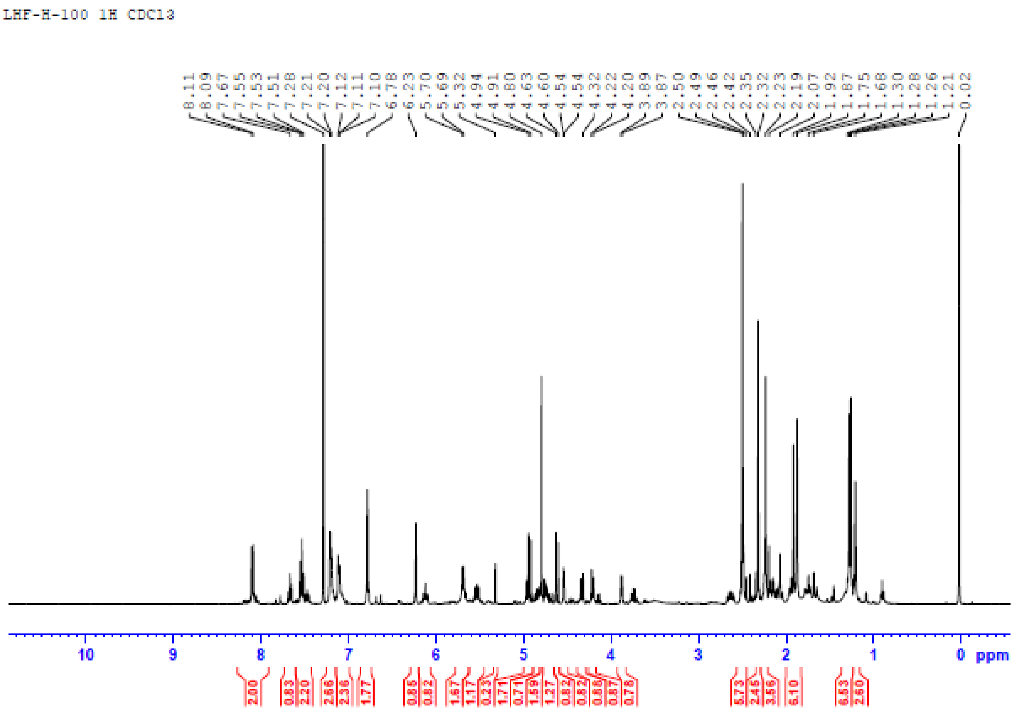

^{1}H-NMR of 5j

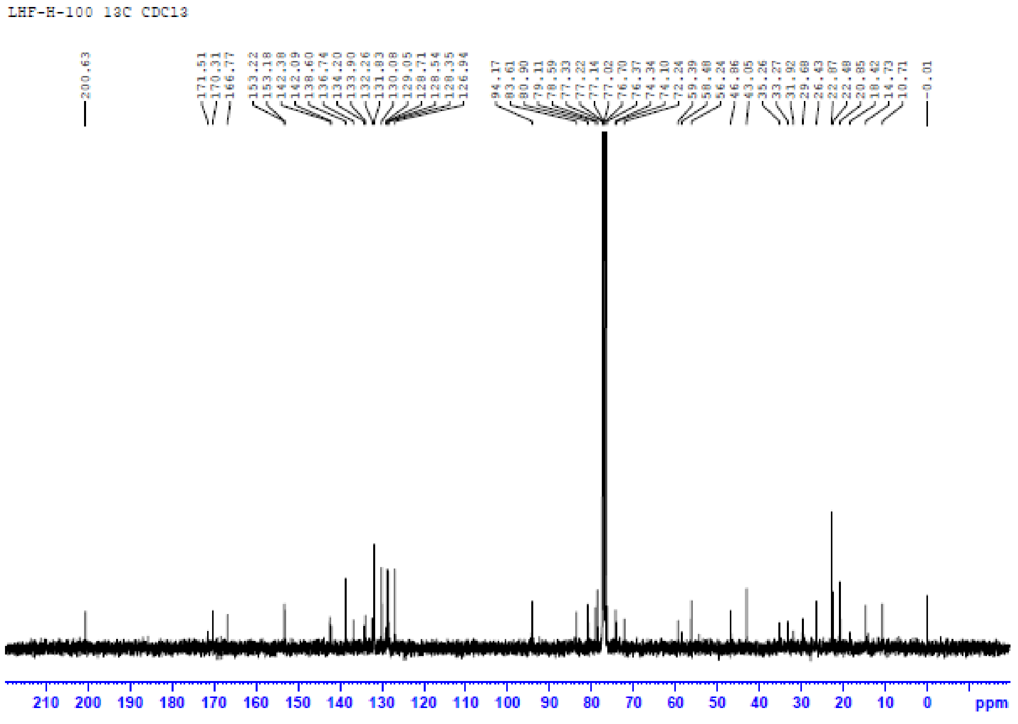

^{13}C-NMR of 5j

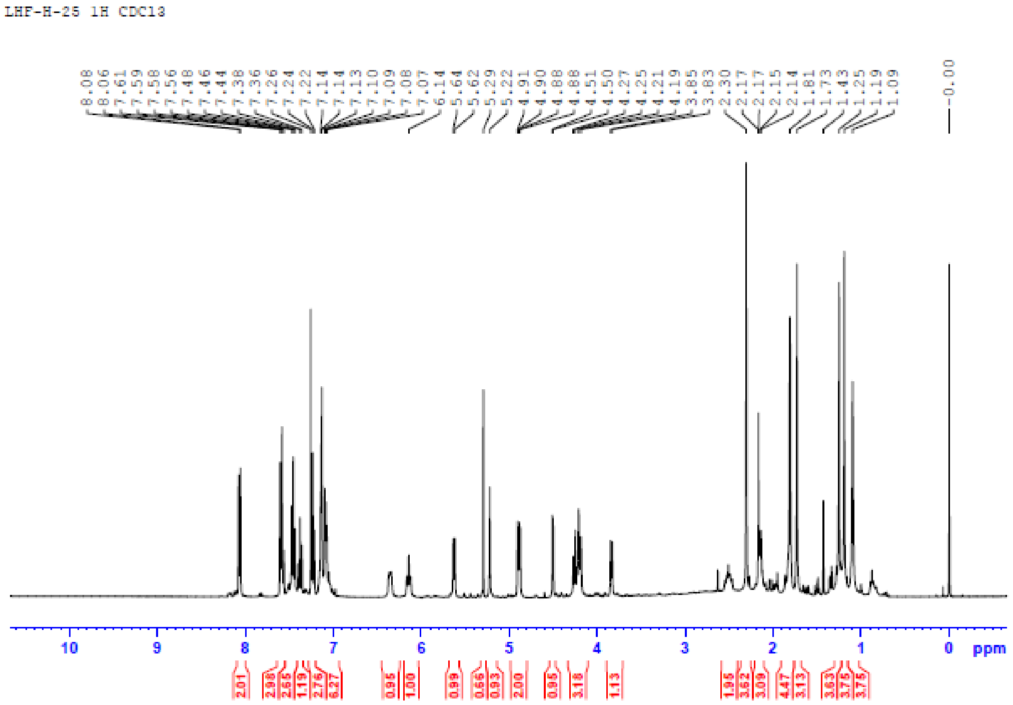

^{1}H-NMR of 3a

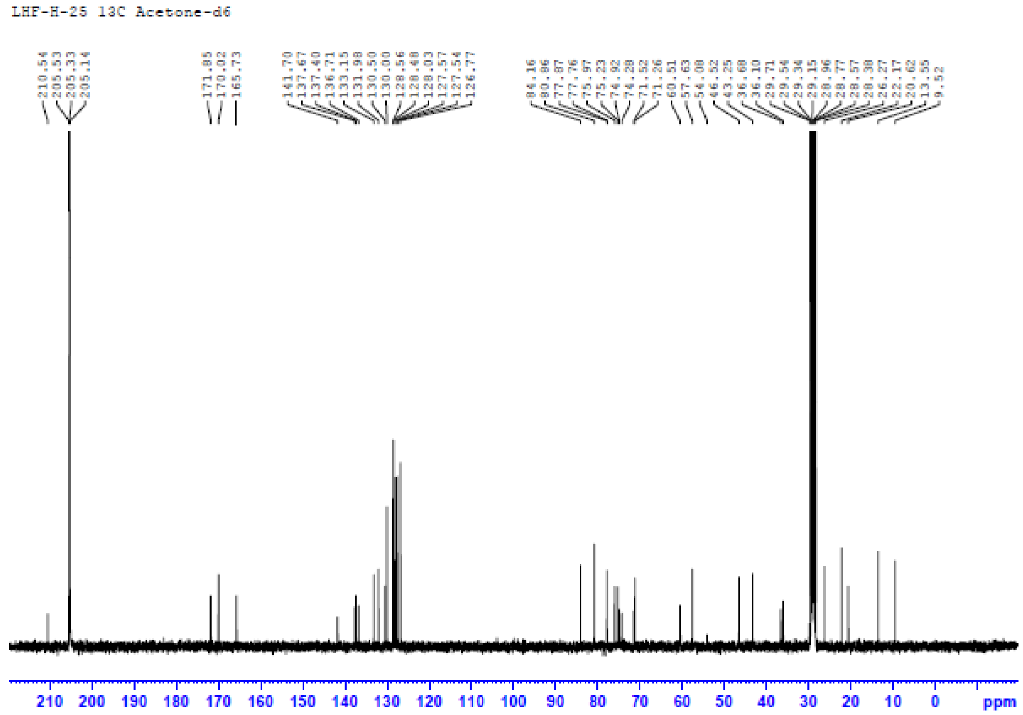

^{13}C-NMR of 3a

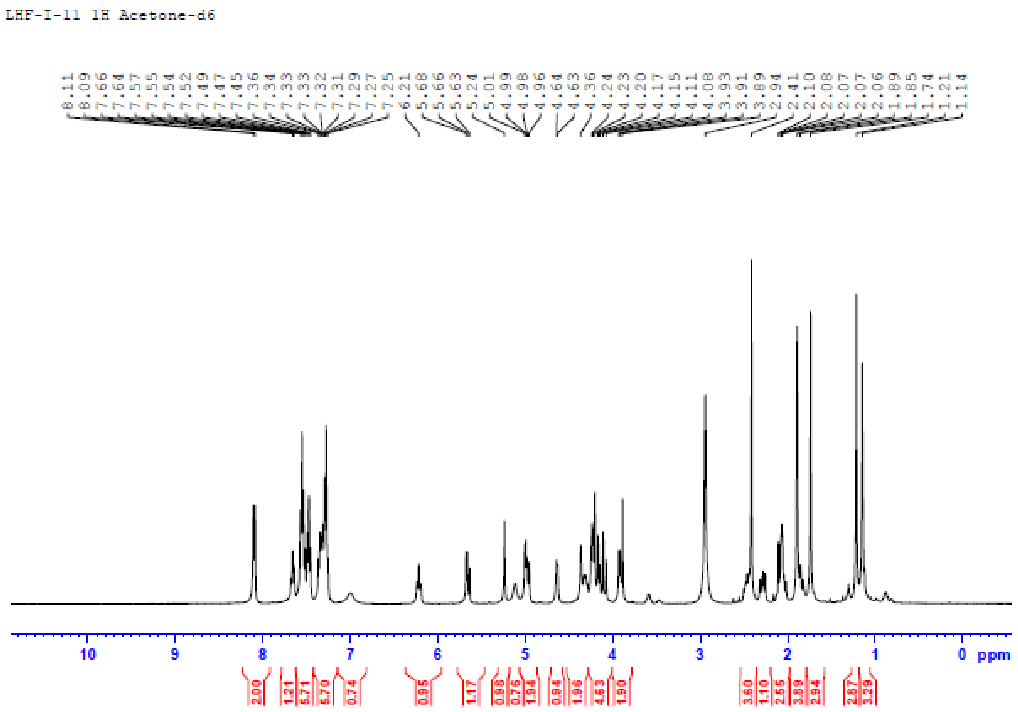

^{1}H-NMR of 3b

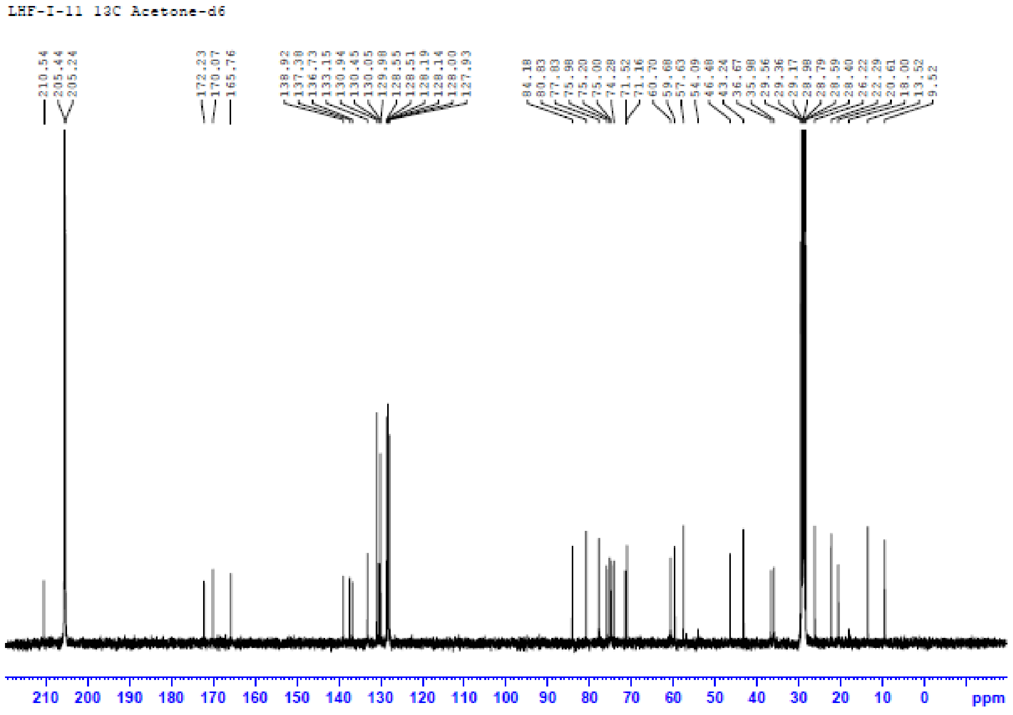

^{13}C-NMR of 3b

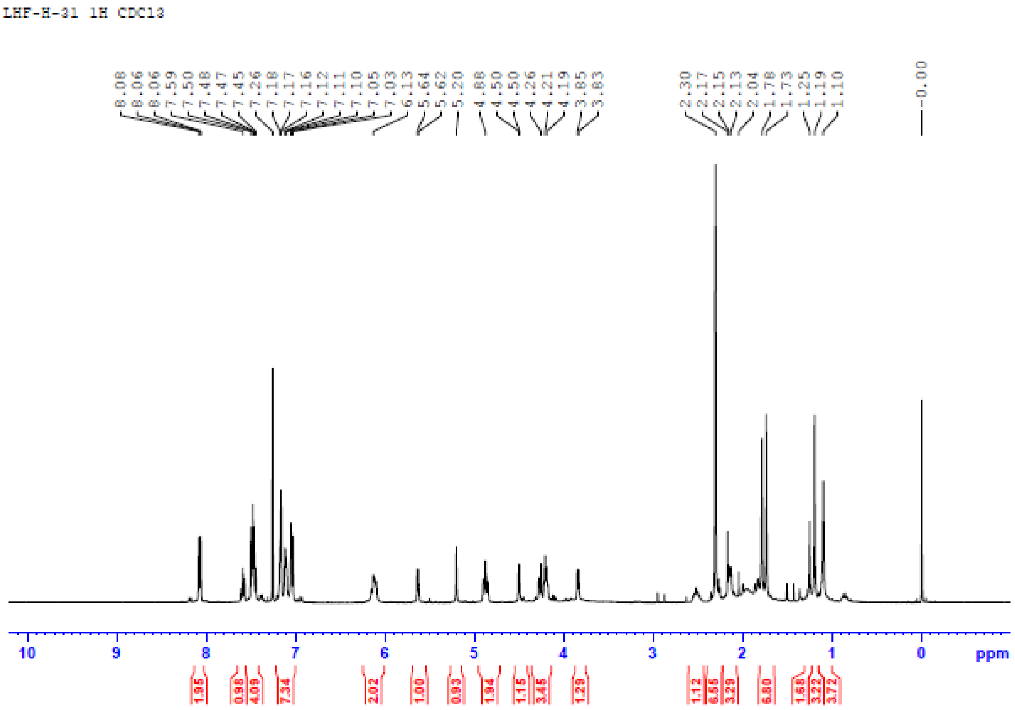

^{1}H-NMR of 3c

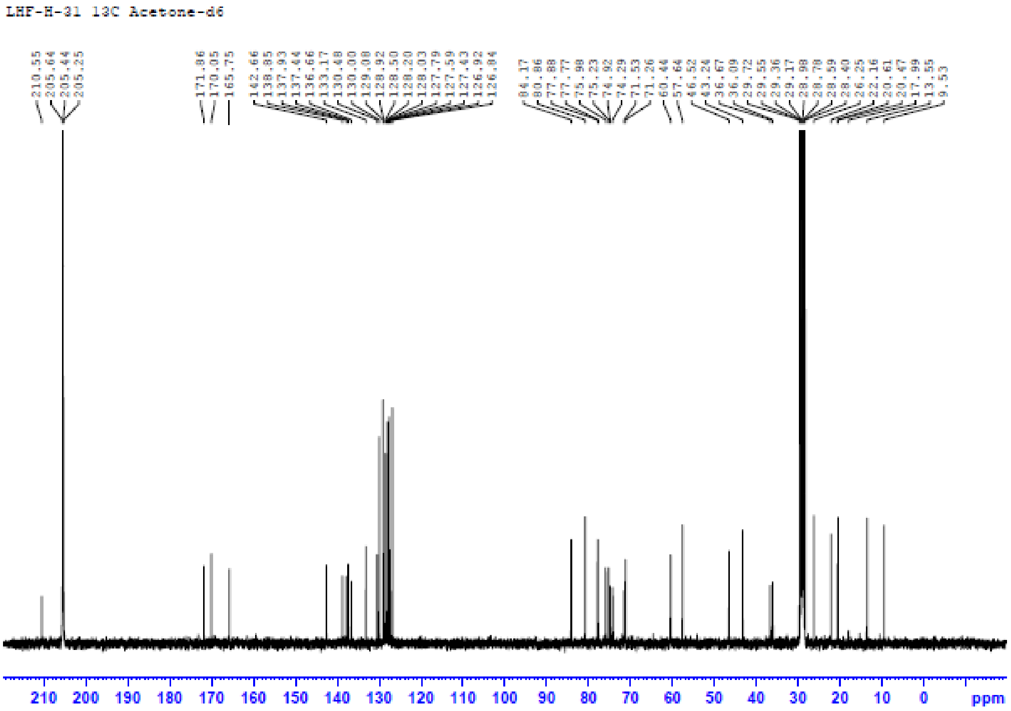

^{13}C-NMR of 3c

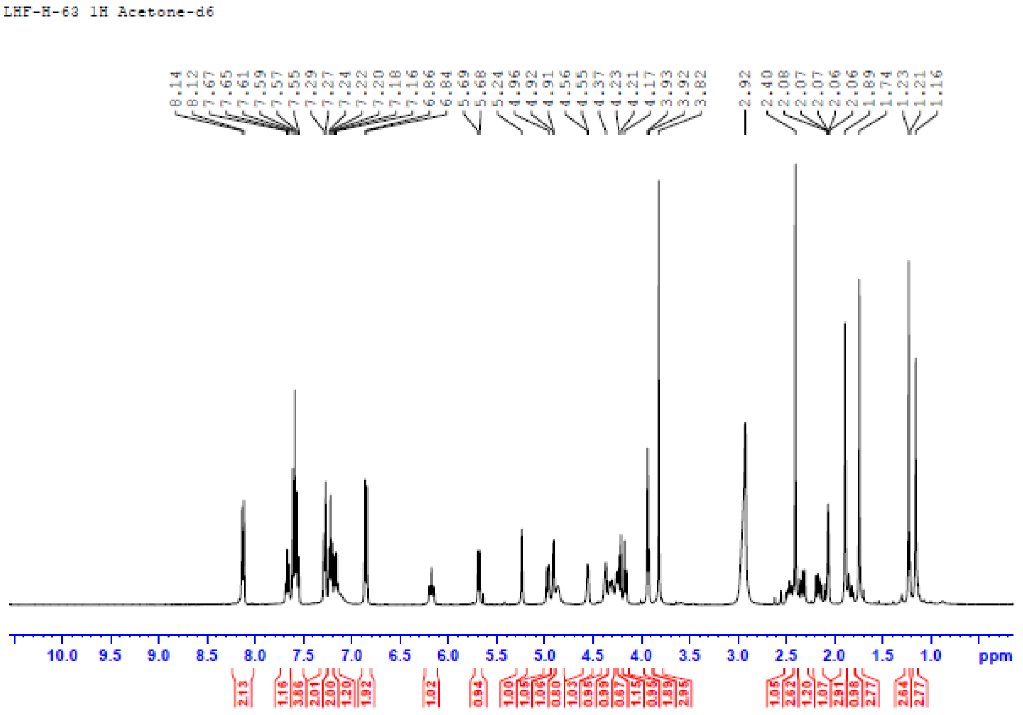

^{1}H-NMR of 3d

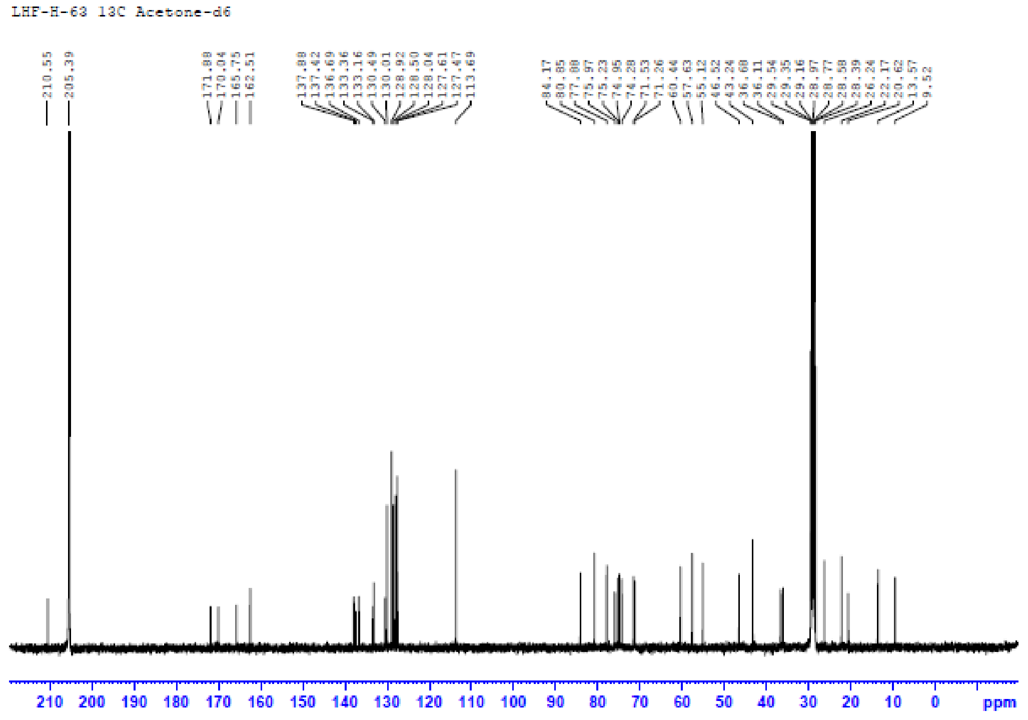

^{13}C-NMR of 3d

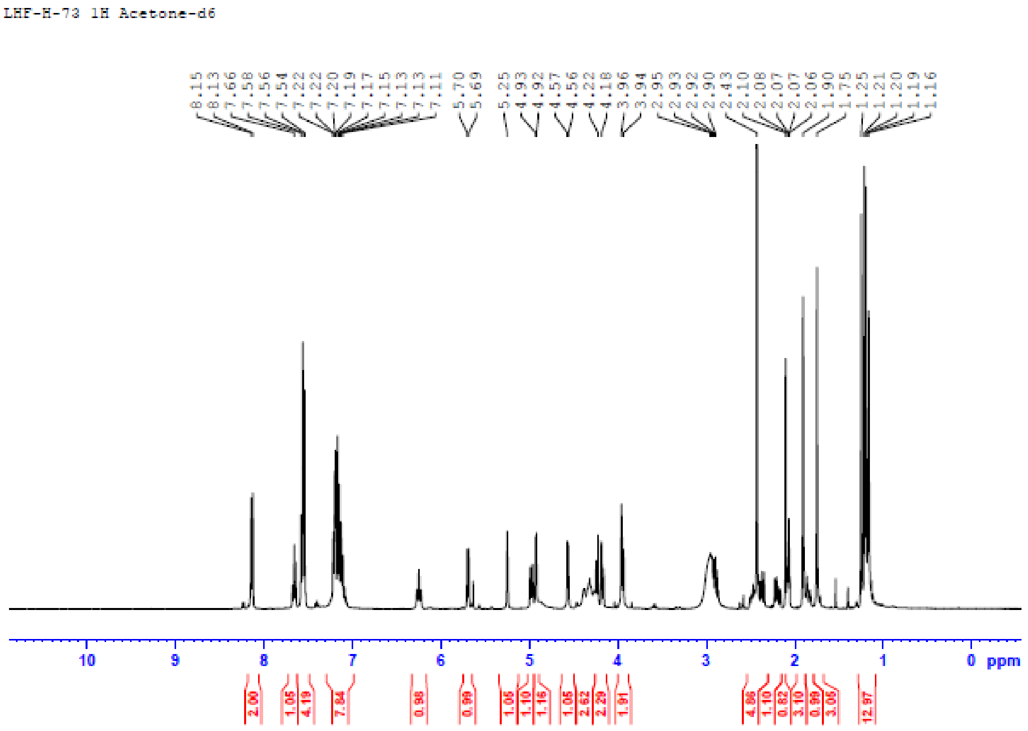

^{1}H-NMR of 3e

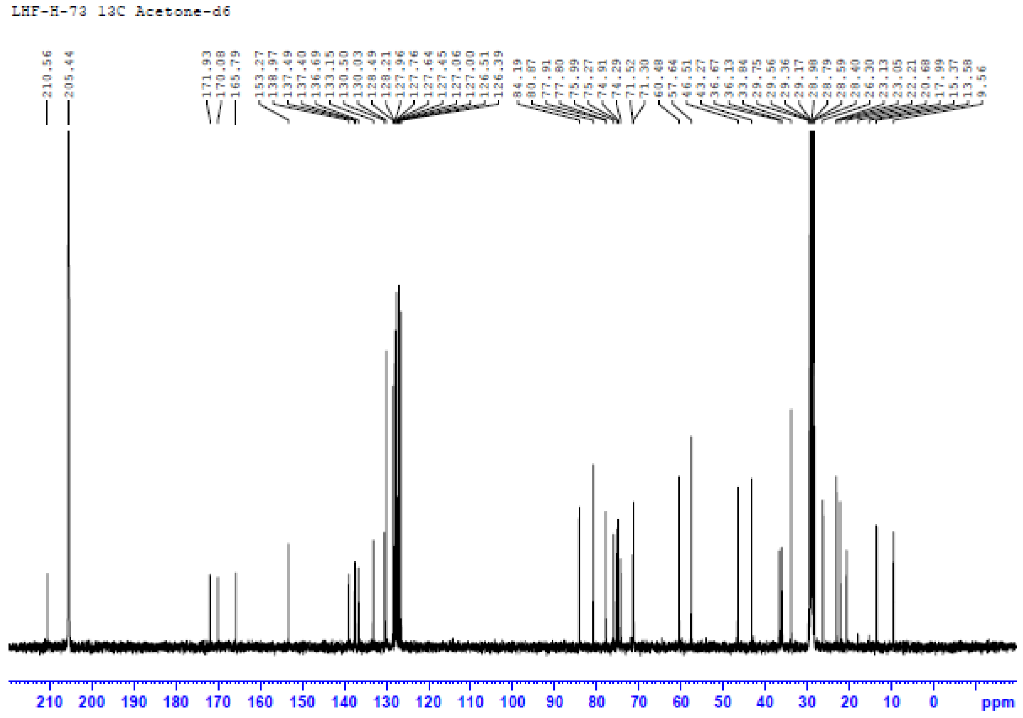

^{13}C-NMR of 3e

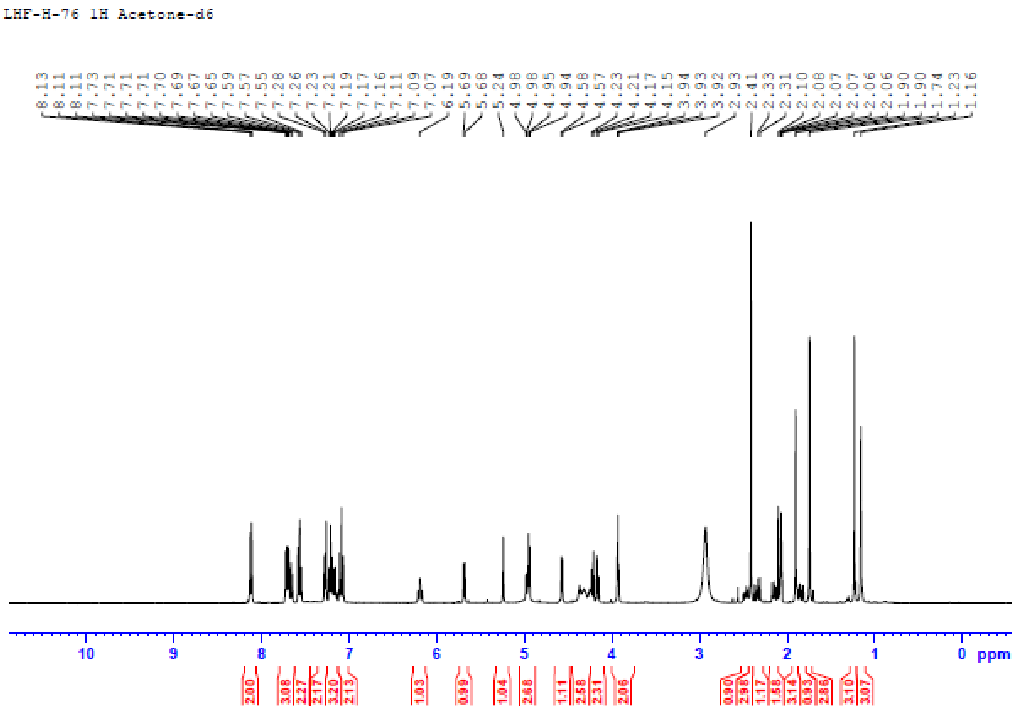

^{1}H-NMR of 3f

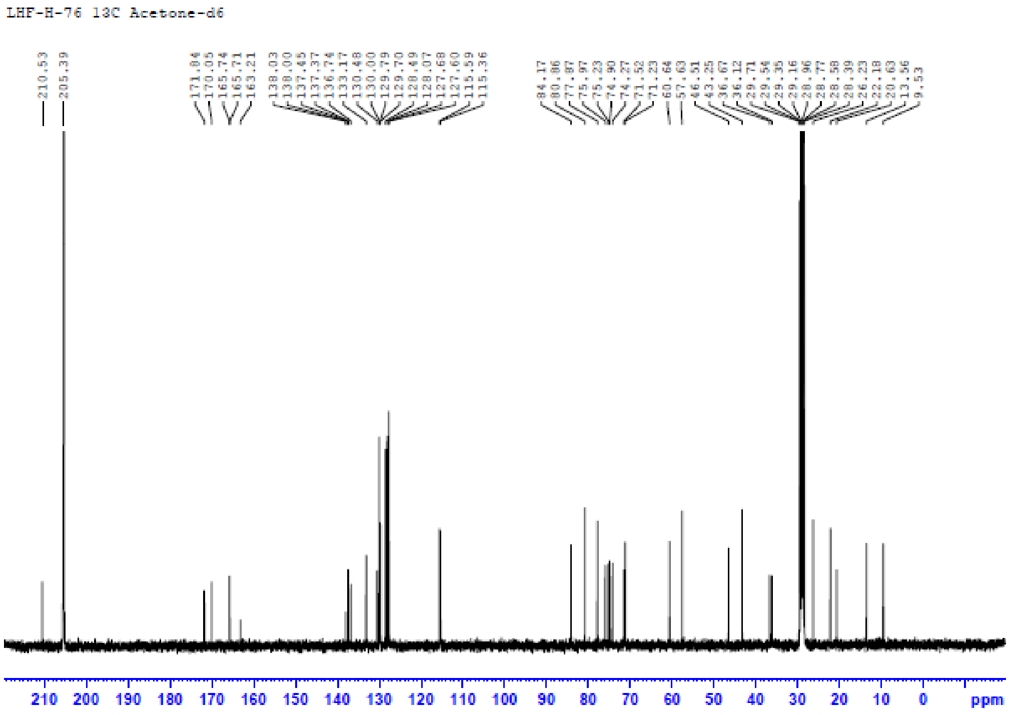

^{13}C-NMR of 3f

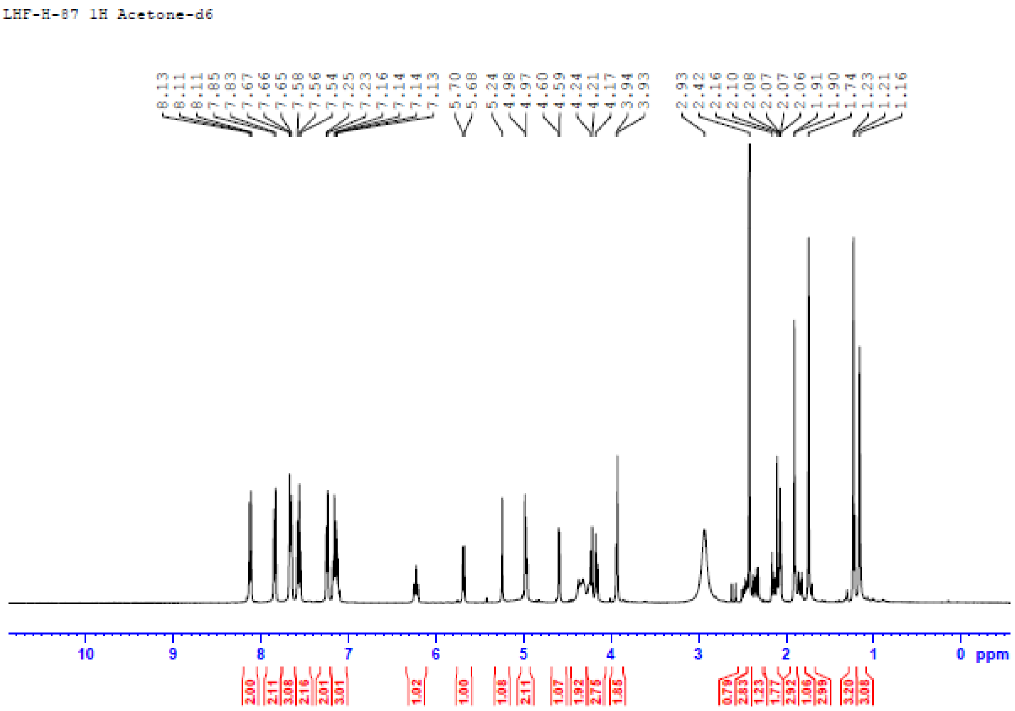

^{1}H-NMR of 3g

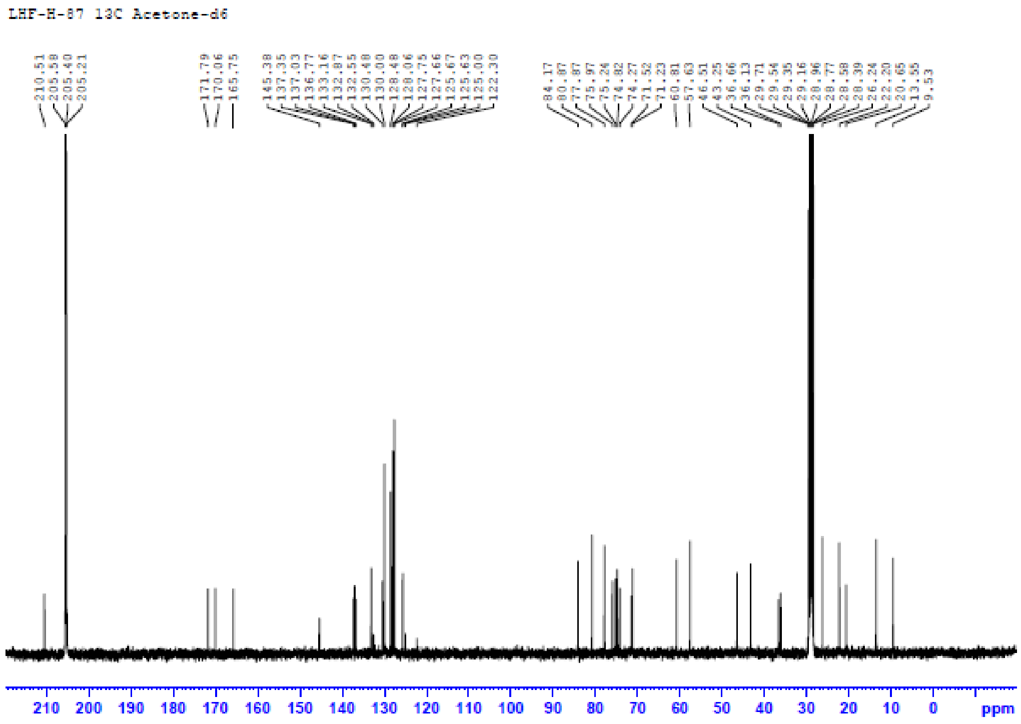

^{13}C-NMR of 3g

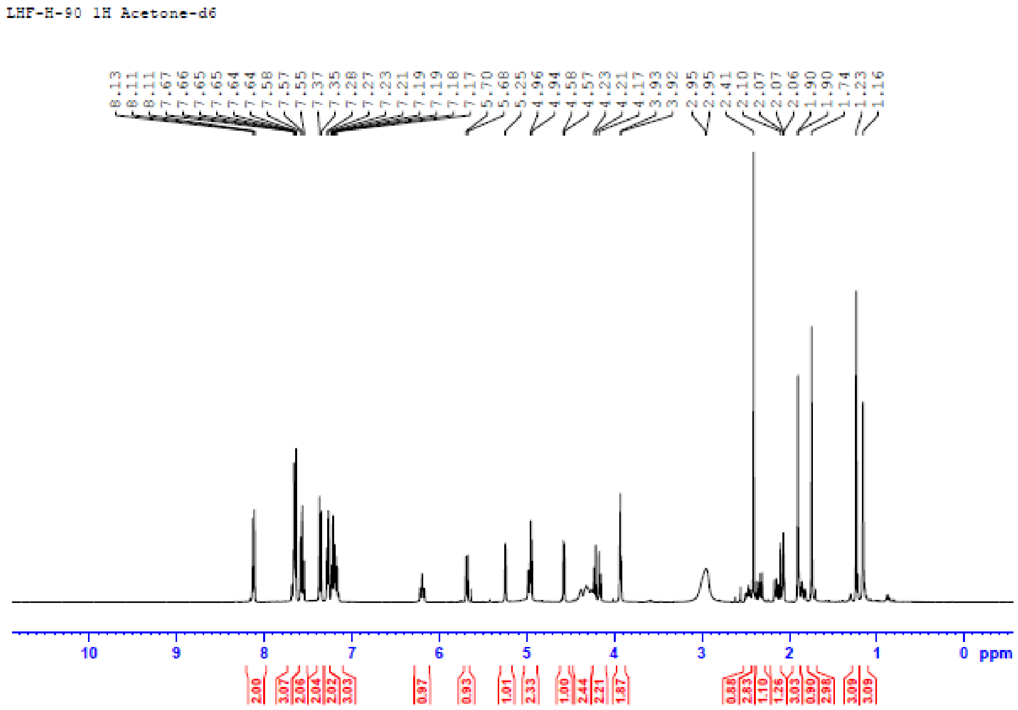

^{1}H-NMR of 3h

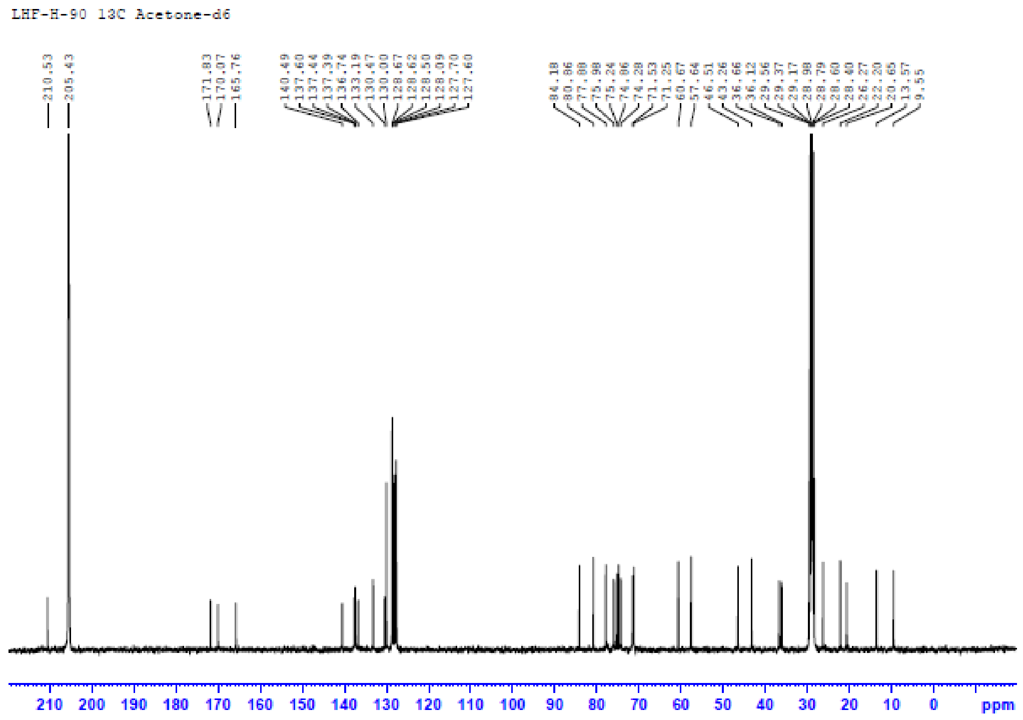

^{13}C-NMR of 3h

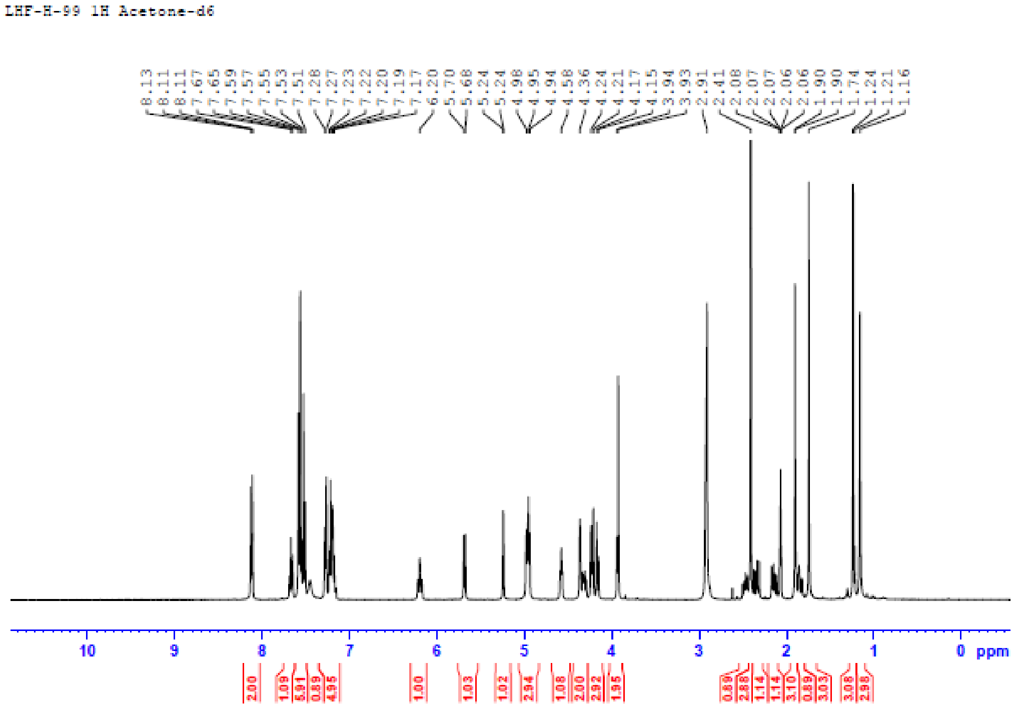

^{1}H-NMR of 3i

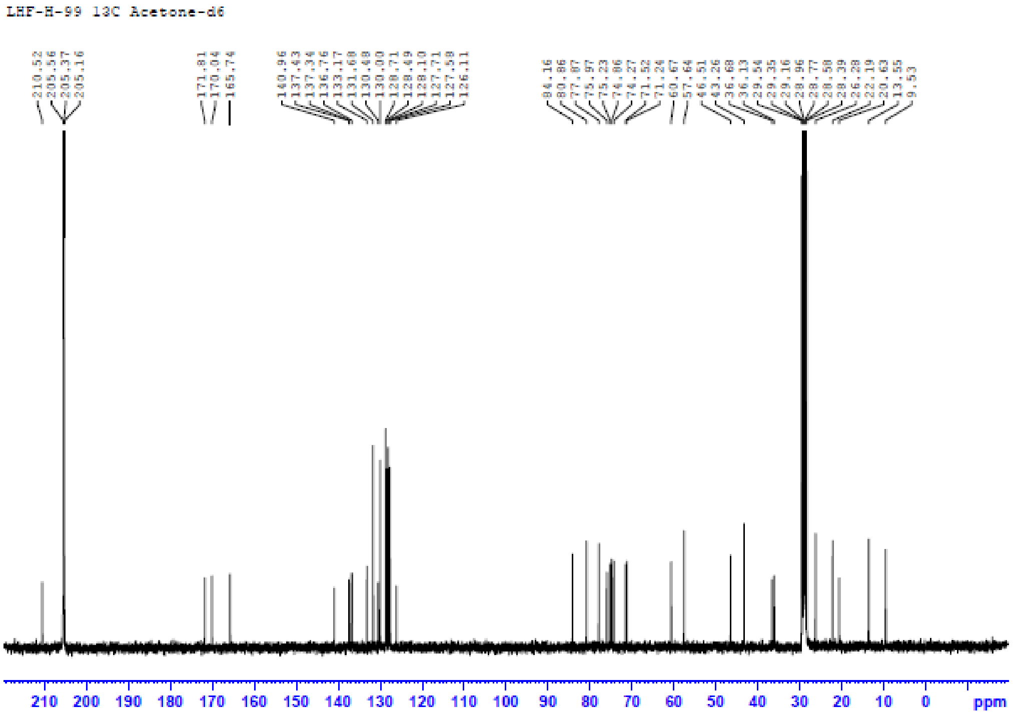

^{13}C-NMR of 3i

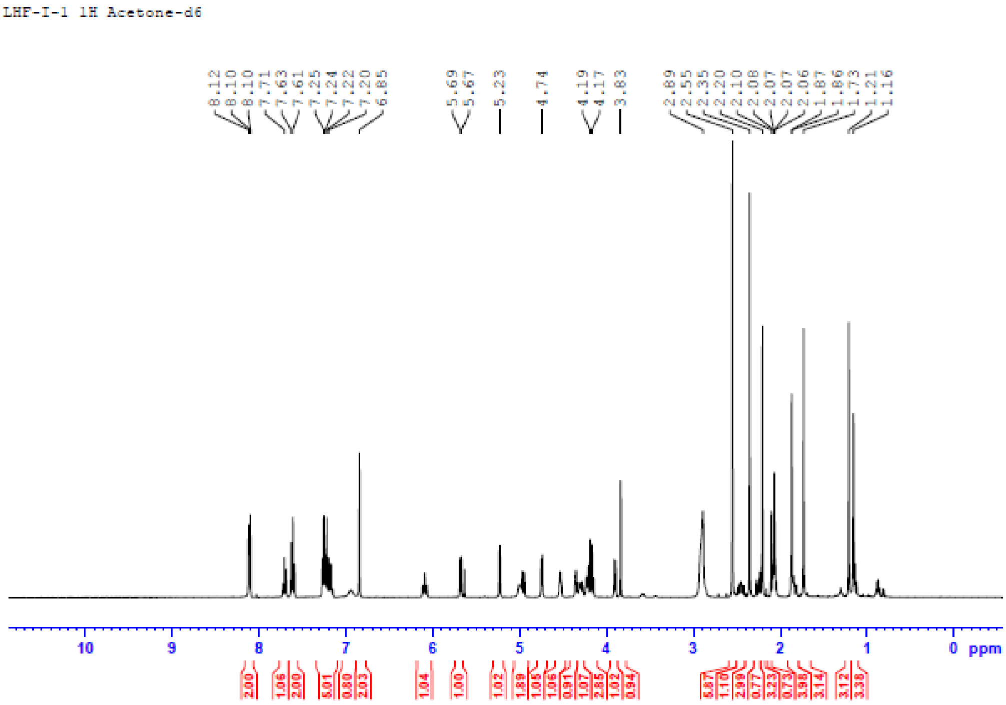

^{1}H-NMR of 3j

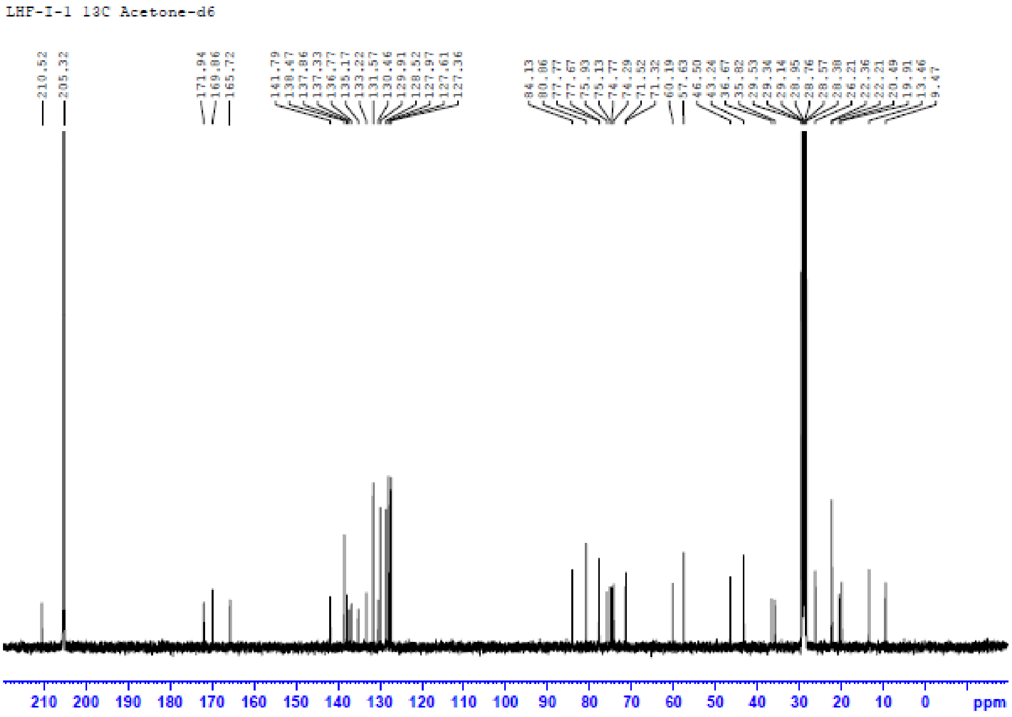

^{13}C-NMR of 3j

Shanghai Mass Spectrometry Center

Shanghai Institute of Organic Chemistry

Chinese Academy of Sciences

High Resolution MS Data Report

Instrument

Bruker Daltonics, Inc. APEXIII 7.0 TESLA FTMS

Card Serial Number	F130792
Analysis Name	D:\Data\zfj2013\2013704_000008.d
Sample Name	LHF-H-25
Acquisition Date	4/21/2013 10:02:33 AM
Operator:	zfj
Ionization Mode	ESI-Positive
Ion Mass (Measured)	870.2802

Sum Formula	Sigma	m/z	Err [ppm]	Mean Err [ppm]	Err [mDa]	rdb	N Rule	e^-
C 40 H 49 N 1 Na 1 O 19	0.071	870.2791	-1.29	-0.06	-1.12	16.50	ok	even
C 43 H 47 N 2 Na 1 O 16	0.077	870.2818	1.79	2.99	1.56	21.00	ok	odd
C 37 H 53 N 1 Na 1 O 19 S 1	0.082	870.2825	2.58	3.82	2.25	11.50	ok	even
C 44 H 49 N 1 Na 1 O 14 S 1	0.093	870.2766	-4.17	-2.92	-3.63	20.50	ok	even
C 47 H 47 N 2 Na 1 O 11 S 1	0.102	870.2793	-1.09	0.12	-0.95	25.00	ok	odd

HR-ESI of 3a

Shanghai Mass Spectrometry Center

Shanghai Institute of Organic Chemistry

Chinese Academy of Sciences

High Resolution MS Data Report

Instrument

Bruker Daltonics, Inc. APEXIII 7.0 TESLA FTMS

Card Serial Number	F130799
Analysis Name	D:\Data\zfj2013\2013704_000009.d
Sample Name	LHF-I-11
Acquisition Date	4/21/2013 10:03:31 AM
Operator:	zfj
Ionization Mode	ESI-Positive
Ion Mass (Measured)	884.2944

Sum Formula	Sigma	m/z	Err [ppm]	Mean Err [ppm]	Err [mDa]	rdb	N Rule	e^-
C 44 H 49 N 2 Na 1 O 16	0.080	884.2974	3.37	3.47	2.98	21.00	ok	odd
C 45 H 51 N 1 Na 1 O 14 S 1	0.164	884.2922	-2.49	2.81	-2.20	20.50	ok	even
C 48 H 49 N 2 Na 1 O 11 S 1	0.167	884.2949	0.54	6.13	0.48	25.00	ok	odd

HR-ESI of 3b

Shanghai Mass Spectrometry Center

Shanghai Institute of Organic Chemistry

Chinese Academy of Sciences

High Resolution MS Data Report

Instrument

Bruker Daltonics, Inc. APEXIII 7.0 TESLA FTMS

Card Serial Number	F130795
Analysis Name	D:\Data\zfj2013\2013704_000008.d
Sample Name	LHF-H-31
Acquisition Date	4/21/2013 10:02:33 AM
Operator:	zfj
Ionization Mode	ESI-Positive
Ion Mass (Measured)	884.2942

Sum Formula	Sigma	m/z	Err [ppm]	Mean Err [ppm]	Err [mDa]	rdb	N Rule	e^-
C 44 H 49 N 2 Na 1 O 16	0.024	884.2974	3.67	2.62	3.24	21.00	ok	odd
C 41 H 51 N 1 Na 1 O 19	0.034	884.2947	0.64	-0.42	0.56	16.50	ok	even
C 45 H 51 N 1 Na 1 O 14 S 1	0.135	884.2922	-2.19	1.35	-1.94	20.50	ok	even
C 48 H 49 N 2 Na 1 O 11 S 1	0.137	884.2949	0.84	4.54	0.74	25.00	ok	odd
C 38 H 55 N 1 Na 1 O 19 S 1	0.138	884.2981	4.45	8.15	3.93	11.50	ok	even

HR-ESI of 3c

Shanghai Mass Spectrometry Center

Shanghai Institute of Organic Chemistry

Chinese Academy of Sciences

High Resolution MS Data Report

Instrument

Bruker Daltonics, Inc. APEXIII 7.0 TESLA FTMS

Card Serial Number	F130794
Analysis Name	D:\Data\zfj2013\2013704_000008.d
Sample Name	LHF-H-63
Acquisition Date	4/21/2013 10:02:33 AM
Operator:	zfj
Ionization Mode	ESI-Positive
Ion Mass (Measured)	900.2889

Sum Formula	Sigma	m/z	Err [ppm]	Mean Err [ppm]	Err [mDa]	rdb	N Rule	e^-
C 44 H 49 N 2 Na 1 O 17	0.099	900.2923	3.80	2.34	3.42	21.00	ok	odd
C 41 H 51 N 1 Na 1 O 20	0.107	900.2897	0.82	-0.64	0.74	16.50	ok	even
C 48 H 49 N 2 Na 1 O 12 S 1	0.127	900.2898	1.02	7.23	0.92	25.00	ok	odd
C 45 H 51 N 1 Na 1 O 15 S 1	0.129	900.2872	-1.96	4.23	-1.76	20.50	ok	even
C 42 H 53 Na 1 O 18 S 1	0.133	900.2845	-4.93	1.24	-4.44	16.00	ok	odd
C 38 H 55 N 1 Na 1 O 20 S 1	0.139	900.2930	4.57	10.60	4.11	11.50	ok	even

HR-ESI of 3d

Shanghai Mass Spectrometry Center

Shanghai Institute of Organic Chemistry

Chinese Academy of Sciences

High Resolution MS Data Report

Instrument

Bruker Daltonics, Inc. APEXIII 7.0 TESLA FTMS

Card Serial Number	F130794
Analysis Name	D:\Data\zfj2013\2013704_000008.d
Sample Name	LHF-H-73
Acquisition Date	4/21/2013 10:02:33 AM
Operator:	zfj
Ionization Mode	ESI-Positive
Ion Mass (Measured)	912.3239

Sum Formula	Sigma	m/z	Err [ppm]	Mean Err [ppm]	Err [mDa]	rdb	N Rule	e^-
C 47 H 55 N 1 Na 1 O 14 S 1	0.034	912.3235	-0.42	-0.36	-0.39	20.50	ok	even
C 44 H 57 Na 1 O 17 S 1	0.039	912.3209	-3.36	-3.29	-3.07	16.00	ok	odd
C 43 H 55 N 1 Na 1 O 19	0.044	912.3260	2.32	2.71	2.12	16.50	ok	even

HR-ESI of 3e

Shanghai Mass Spectrometry Center

Shanghai Institute of Organic Chemistry

Chinese Academy of Sciences

High Resolution MS Data Report

Instrument

Bruker Daltonics, Inc. APEXIII 7.0 TESLA FTMS

Card Serial Number	F130800
Analysis Name	D:\Data\zfj2013\2013704_000007.d
Sample Name	LHF-H-76
Acquisition Date	4/21/2013 10:01:59 AM
Operator:	zfj
Ionization Mode	ESI-Positive
Ion Mass (Measured)	888.2700

Sum Formula	Sigma	m/z	Err [ppm]	Mean Err [ppm]	Err [mDa]	rdb	N Rule	e^-
C 46 H 45 N 2 Na 1 O 15	0.088	888.2712	1.39	-0.74	1.24	25.00	ok	odd
C 44 H 48 F 1 N 1 Na 1 O 14 S 1	0.098	888.2672	-3.15	-5.37	-2.80	20.50	ok	even
C 47 H 46 F 1 N 2 Na 1 O 11 S 1	0.099	888.2699	-0.14	-2.31	-0.12	25.00	ok	odd
C 47 H 47 N 1 Na 1 O 13 S 1	0.100	888.2660	-4.44	-6.64	-3.94	24.50	ok	even

HR-ESI of 3f

Shanghai Mass Spectrometry Center

Shanghai Institute of Organic Chemistry

Chinese Academy of Sciences

High Resolution MS Data Report

Instrument

BRUKER Bruker Daltonics, Inc. APEXIII 7.0 TESLA FTMS

Card Serial Number	F130795
Analysis Name	D:\Data\zfj2013\2013704_000008.d
Sample Name	LHF-H-87
Acquisition Date	4/21/2013 10:02:33 AM
Operator:	zfj
Ionization Mode	ESI-Positive
Ion Mass (Measured)	938.2651

Sum Formula	Sigma	m/z	Err [ppm]	Mean Err [ppm]	Err [mDa]	rdb	N Rule	e^-
C 44 H 46 F 3 N 2 Na 1 O 16	0.081	938.2692	4.35	4.27	4.08	21.00	ok	odd
C 45 H 48 F 3 N 1 Na 1 O 14 S 1	0.094	938.2640	-1.17	-1.30	-1.10	20.50	ok	even

HR-ESI of 3g

Shanghai Mass Spectrometry Center

Shanghai Institute of Organic Chemistry

Chinese Academy of Sciences

High Resolution MS Data Report

Instrument

BRUKER Bruker Daltonics, Inc. APEXIII 7.0 TESLA FTMS

Card Serial Number	F130801
Analysis Name	D:\Data\zfj2013\2013704_000007.d
Sample Name	LHF-H-90
Acquisition Date	4/21/2013 10:01:59 AM
Operator:	zfj
Ionization Mode	ESI-Positive
Ion Mass (Measured)	904.2359

Sum Formula	Sigma	m/z	Err [ppm]	Mean Err [ppm]	Err [mDa]	rdb	N Rule	e^-
C 47 H 45 Na 1 O 15 S 1	0.256	904.2371	1.38	1.38	1.25	25.00	ok	odd
C 47 H 44 Cl 1 N 1 Na 1 O 14	0.303	904.2343	-1.81	-1.81	-1.63	25.50	ok	even
C 44 H 48 Cl 1 N 1 Na 1 O 14 S 1	0.307	904.2376	1.92	1.92	1.74	20.50	ok	even
C 47 H 46 Cl 1 N 2 Na 1 O 11 S 1	0.322	904.2403	4.88	4.88	4.42	25.00	ok	odd

HR-ESI of 3h

Shanghai Mass Spectrometry Center

Shanghai Institute of Organic Chemistry

Chinese Academy of Sciences

High Resolution MS Data Report

Instrument

Bruker Daltonics, Inc. APEXIII 7.0 TESLA FTMS

Card Serial Number	F130800
Analysis Name	D:\Data\zfj2013\2013704_000011.d
Sample Name	LHF-H-99
Acquisition Date	4/21/2013 10:37:04 AM
Operator:	zfj
Ionization Mode	ESI-Positive
Ion Mass (Measured)	948.1870

Sum Formula	Sigma	m/z	Err [ppm]	Mean Err [ppm]	Err [mDa]	rdb	N Rule	e^-
C 44 H 48 Br 1 N 1 Na 1 O 14 S 1	0.007	948.1871	0.13	-0.24	0.12	20.50	ok	even
C 47 H 46 Br 1 N 2 Na 1 O 11 S 1	0.013	948.1898	2.96	2.58	2.80	25.00	ok	odd
C 47 H 44 Br 1 N 1 Na 1 O 14	0.021	948.1837	-3.42	-3.44	-3.25	25.50	ok	even

HR-ESI of 3i

Shanghai Mass Spectrometry Center

Shanghai Institute of Organic Chemistry

Chinese Academy of Sciences

High Resolution MS Data Report

Instrument

Bruker Daltonics, Inc. APEXIII 7.0 TESLA FTMS

Card Serial Number	F130797
Analysis Name	D:\Data\zfj2013\2013704_000009.d
Sample Name	LHF-I-1
Acquisition Date	4/21/2013 10:03:31 AM
Operator:	zfj
Ionization Mode	ESI-Positive
Ion Mass (Measured)	912.3264

Sum Formula	Sigma	m/z	Err [ppm]	Mean Err [ppm]	Err [mDa]	rdb	N Rule	e^-
C 46 H 53 N 2 Na 1 O 16	0.085	912.3287	2.61	3.10	2.38	21.00	ok	odd
C 47 H 55 N 1 Na 1 O 14 S 1	0.099	912.3235	-3.07	-2.60	-2.80	20.50	ok	even

HR-ESI of 3j

HPLC (Waters e2695; Kromasil C18 column (5 µm, 4.6 mm×250 mm); 0-20min: 5%-100%

MeCN, 20-23min: 100% MeCN; 240 nm)

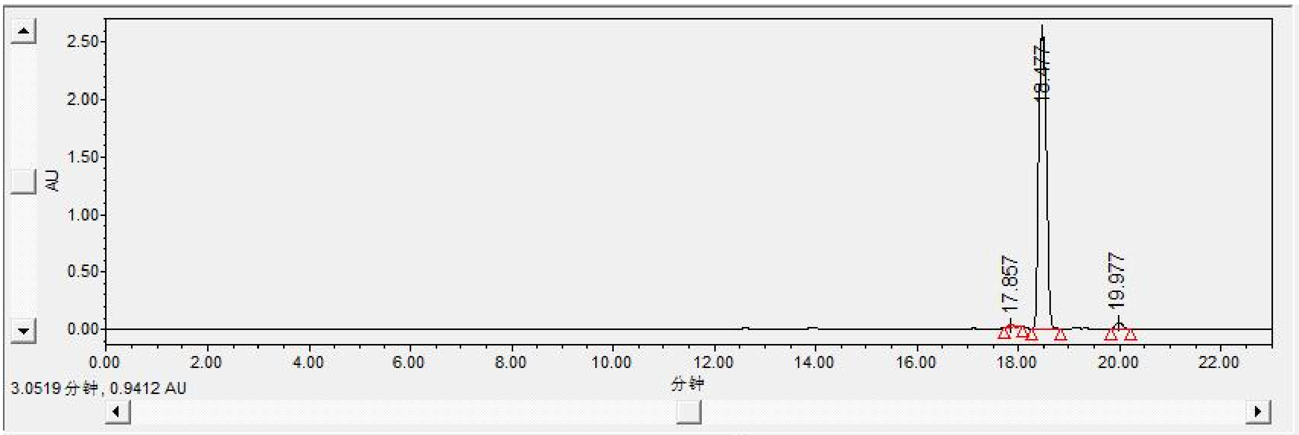

E	名称	保留时间 (分钟)	面积 (微伏*秒)	% 面积	高度 (微伏)	积分类型	含量	单位	峰类型	峰代码
1		17.857	193087	0.72	23757	bb			未知	
2		18.477	26180106	97.20	2569060	bb			未知	
3		19.977	561228	2.08	57043	bb			未知	

HPLC data of 3a

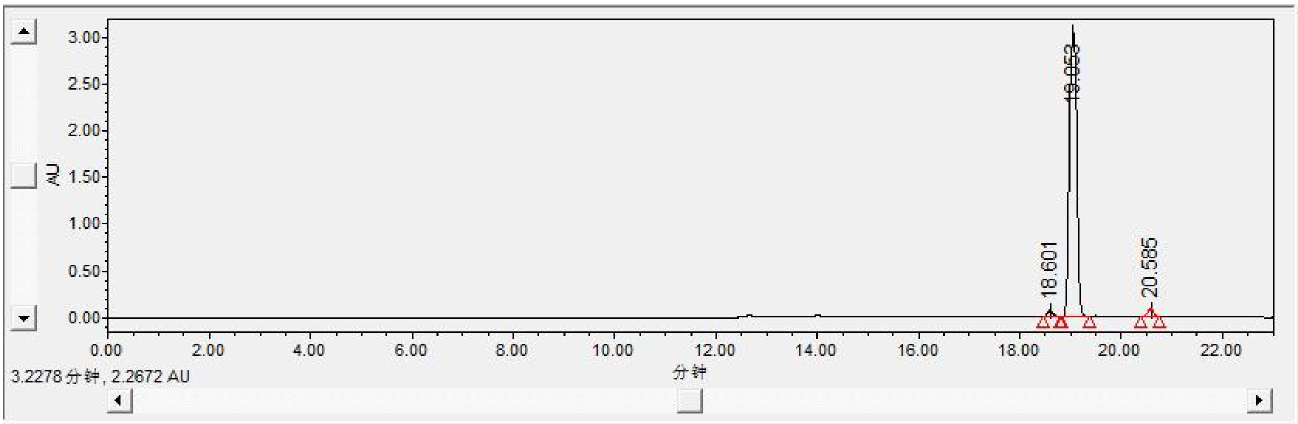

E	名称	保留时间 (分钟)	面积 (微伏*秒)	% 面积	高度 (微伏)	积分类型	含量	单位	峰类型	峰代码
1		18.601	546977	1.70	71448	bb			未知	
2		19.053	31005296	96.15	3038088	bb			未知	
3		20.585	696168	2.16	83270	bb			未知	

HPLC data of 3b

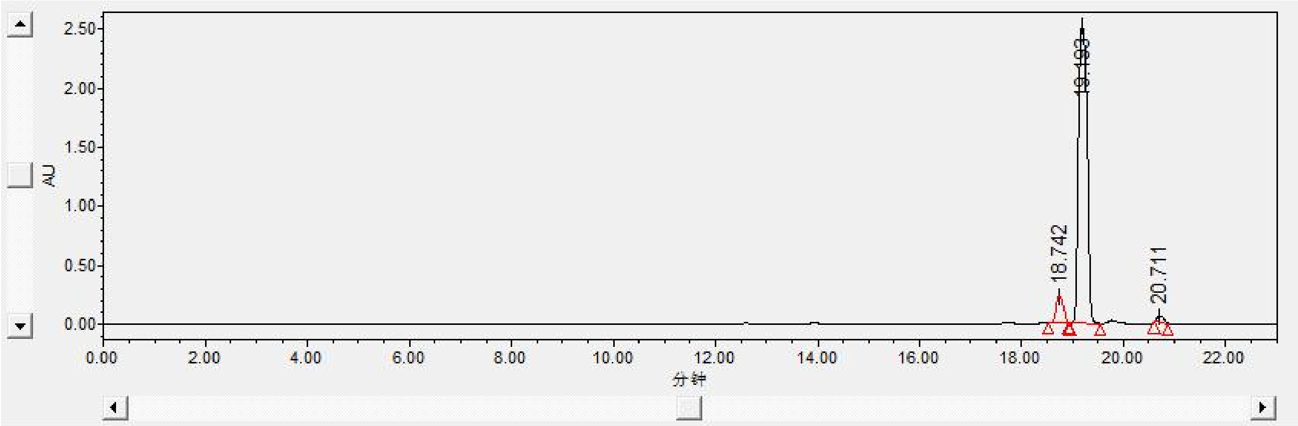

E	名称	保留时间 (分钟)	面积 (微伏*秒)	% 面积	高度 (微伏)	积分类型	含量	单位	峰类型	峰代码
1		18.742	2273804	7.12	226640	bb			未知	
2		19.193	29114857	91.23	2512288	bb			未知	
3		20.711	525752	1.65	57239	bb			未知	

HPLC data of 3c

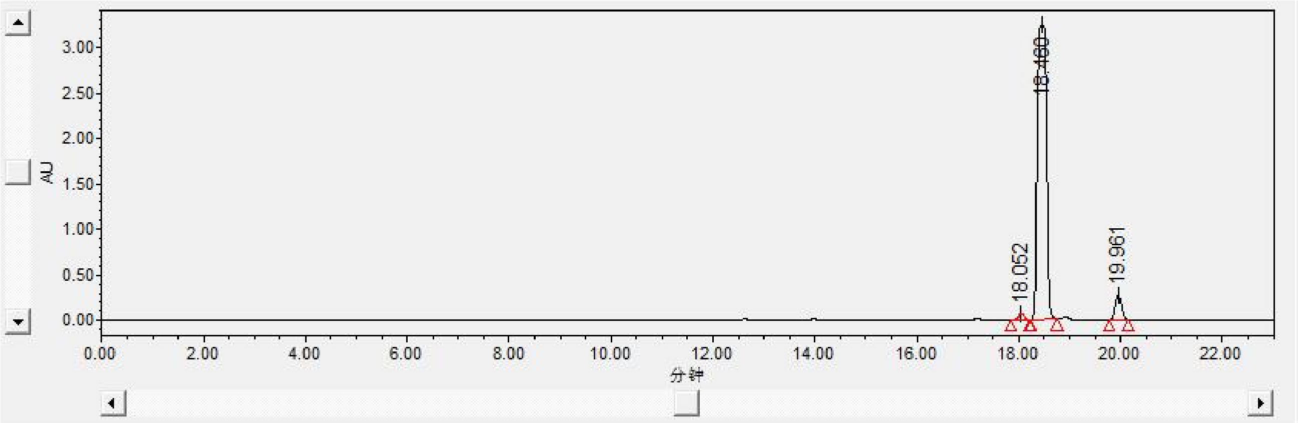

E	名称	保留时间 (分钟)	面积 (微伏*秒)	% 面积	高度 (微伏)	积分类型	含量	单位	峰类型	峰代码
1		18.052	595703	1.36	72707	bb			未知	
2		18.460	40825979	93.36	3238087	bb			未知	
3		19.961	2308970	5.28	270794	bb			未知	

HPLC data of 3d

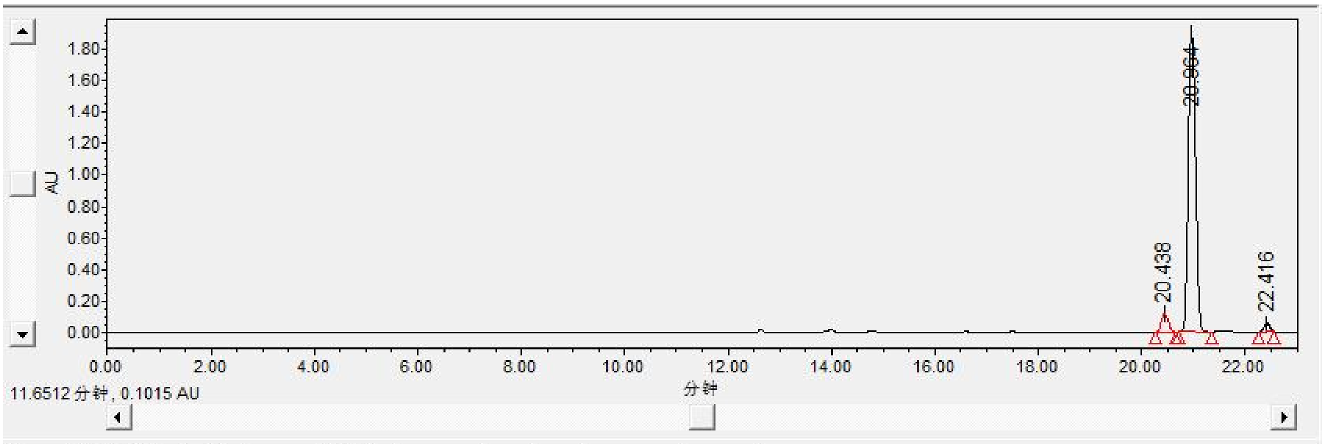

E	名称	保留时间 (分钟)	面积 (微伏*秒)	% 面积	高度 (微伏)	积分类型	含量	单位	峰类型	峰代码
1		20.438	1097474	5.52	117786	bb			未知	
2		20.964	18344342	92.25	1884100	bb			未知	
3		22.416	443936	2.23	54185	bb			未知	

HPLC data of 3e

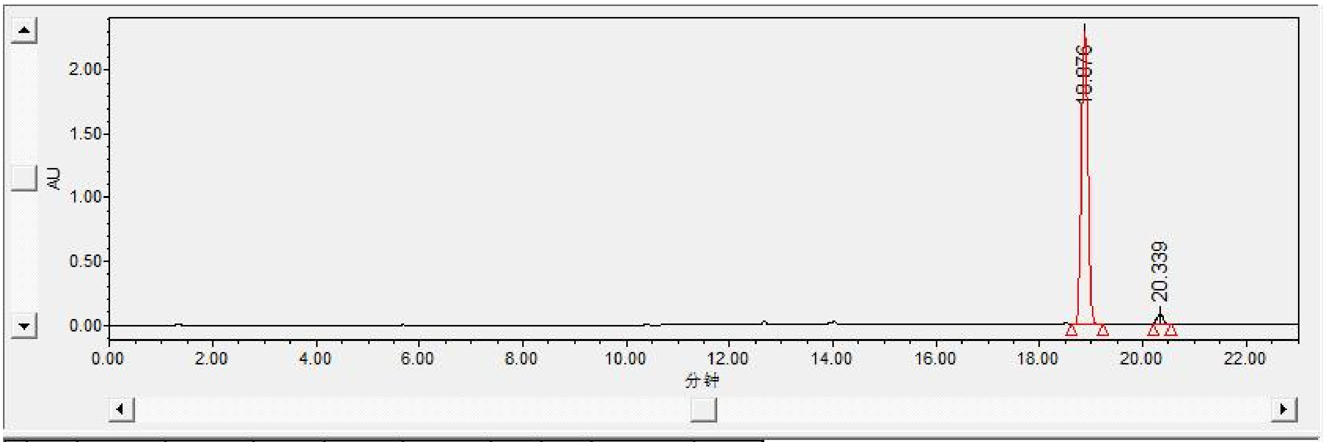

E	名称	保留时间 (分钟)	面积 (微伏*秒)	% 面积	高度 (微伏)	积分类型	含量	单位	峰类型	峰代码
1		18.876	20138377	96.81	2294533	bb			未知	
2		20.339	663889	3.19	79023	bb			未知	

HPLC data of 3f

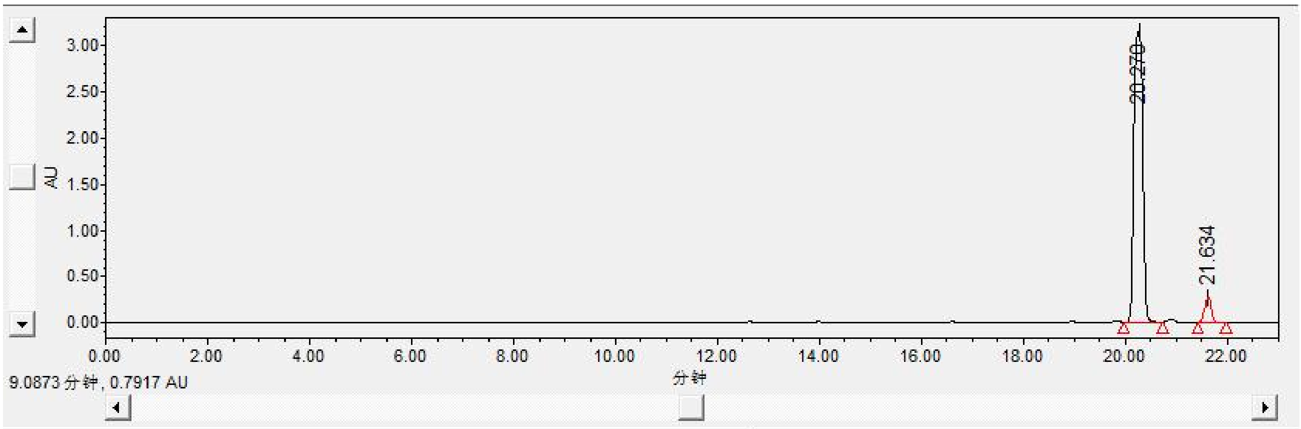

	名称	保留时间 (分钟)	面积 (微伏*秒)	% 面积	高度 (微伏)	积分类型	含量	单位	峰类型	峰代码
1		20.270	37286703	93.97	3130358	bb			未知	
2		21.634	2391315	6.03	273403	bb			未知	

HPLC data of 3g

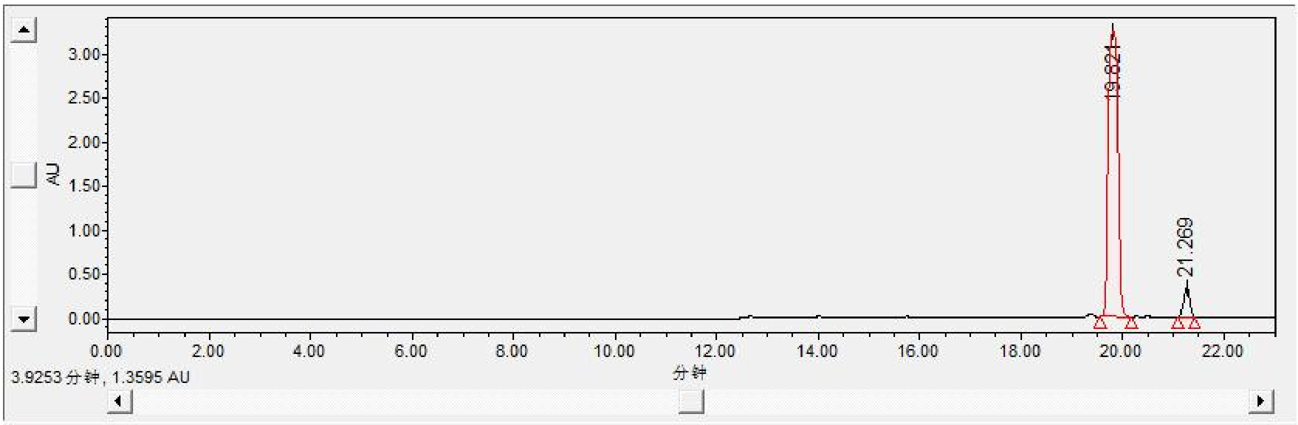

	名称	保留时间 (分钟)	面积 (微伏*秒)	% 面积	高度 (微伏)	积分类型	含量	单位	峰类型	峰代码
1		19.821	43459309	93.67	3232543	bb			未知	
2		21.269	2937510	6.33	336861	bb			未知	

HPLC data of 3h

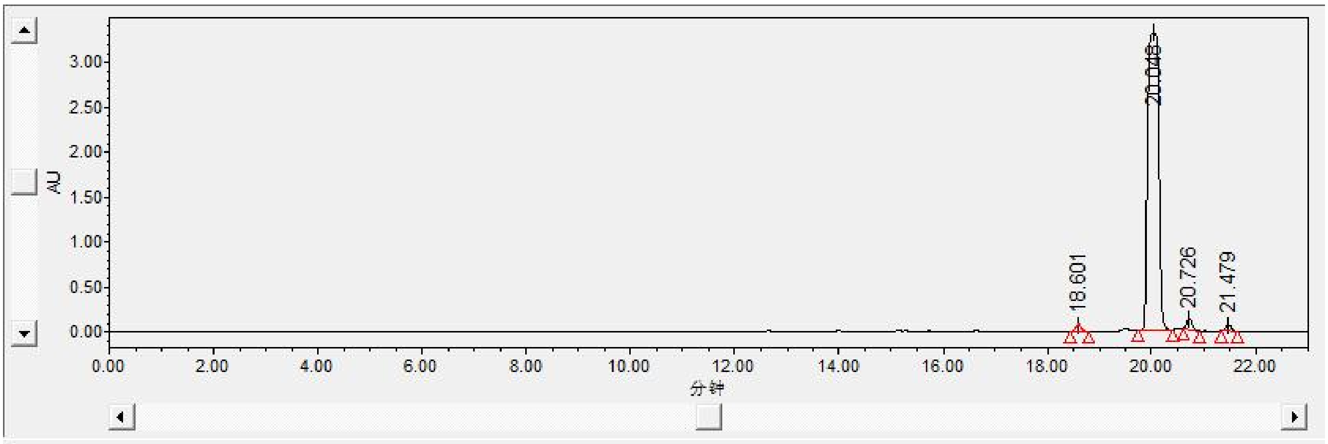

E	名称	保留时间(分钟)	面积(微伏*秒)	% 面积	高度(微伏)	积分类型	含量	单位	峰类型	峰代码
1		18.601	673404	1.34	82007	bb			未知	
2		20.048	48168798	95.75	3306880	bb			未知	
3		20.726	898346	1.79	117424	bb			未知	
4		21.479	565168	1.12	74040	bb			未知	

HPLC data of 3i

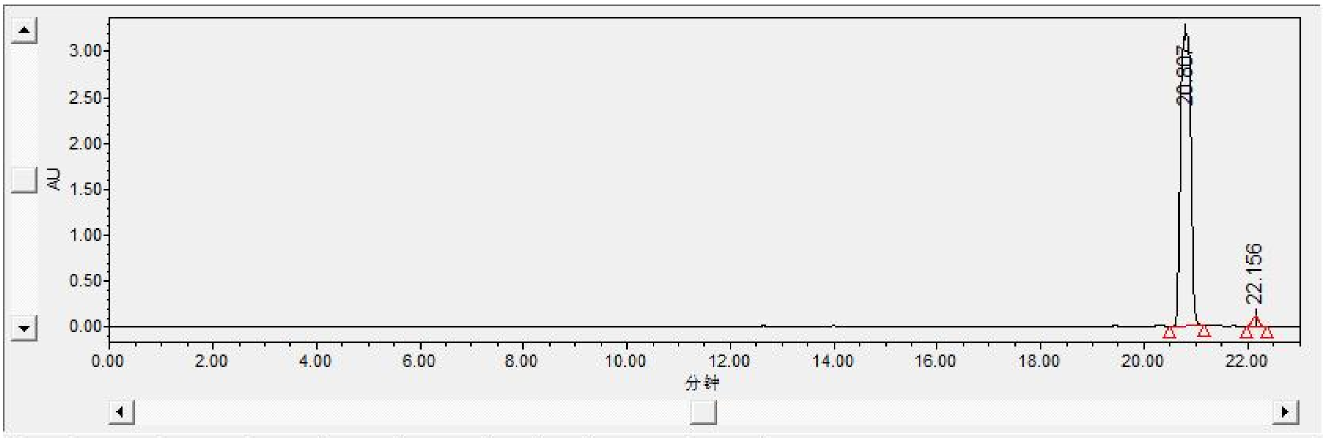

E	名称	保留时间(分钟)	面积(微伏*秒)	% 面积	高度(微伏)	积分类型	含量	单位	峰类型	峰代码
1		20.807	43614793	97.92	3192182	bb			未知	
2		22.156	927996	2.08	106859	bb			未知	

HPLC data of 3j
